# Application of Targeted Y-Chromosomal Capture Enrichment to Increase the Resolution of Native American Haplogroup Q

**DOI:** 10.1155/2024/3046495

**Published:** 2024-07-29

**Authors:** Zehra Köksal, Claus Børsting, Graciela Bailliet, Germán Burgos, Elizeu Carvalho, Andrea Casas-Vargas, Adriana Castillo, Marilia Brito Gomes, Beatriz Martínez, Humberto Ossa, María Laura Parolin, Alfredo Quiroz, Ulises Toscanini, William Usaquén, Irina F. Velázquez, Carlos Vullo, Leonor Gusmão, Vania Pereira

**Affiliations:** ^1^ Section of Forensic Genetics Department of Forensic Medicine Faculty of Health and Medical Sciences University of Copenhagen, Copenhagen, Denmark; ^2^ Instituto Multidisciplinario de Biología Celular Universidad Nacional de La Plata CCT-CONICET-La Plata CIC, La Plata, Argentina; ^3^ One Health Global Research Group Facultad de Medicina Universidad de Las Américas (UDLA), Quito, Ecuador; ^4^ Grupo de Medicina Xenómica Universidad de Santiago de Compostela, Santiago de Compostela, Spain; ^5^ DNA Diagnostic Laboratory (LDD) State University of Rio de Janeiro (UERJ), Rio de Janeiro, Brazil; ^6^ Grupo de Genética de Poblaciones e Identificación Instituto de Genética Universidad Nacional de Colombia, Bogotá, Colombia; ^7^ Department of Basic Sciences Universidad Industrial de Santander (UIS), Bucaramanga, Colombia; ^8^ Department of Internal Medicine Diabetes Unit State University of Rio de Janeiro (UERJ), Rio de Janeiro, Brazil; ^9^ Instituto de Investigaciones Inmunológicas Universidad de Cartagena, Cartagena, Colombia; ^10^ Department of Hematology Instituto de Previsión Laboratório de Genética y Biología Molecular, Asunción, Paraguay; ^11^ Facultad de Ciencias Pontificia Universidad Javeriana, Bogotá, Colombia; ^12^ Instituto de Diversidad y Evolución Austral (IDEAus) Centro Nacional Patagónico CONICET, Puerto Madryn, Argentina; ^13^ Instituto de Previsión Social, Asunción, Paraguay; ^14^ Primer Centro Argentino de Inmunogenética (PRICAI) Fundación Favaloro, Buenos Aires, Argentina; ^15^ DNA Forensic Laboratory Equipo Argentino de Antropología Forense (EAAF), Córdoba, Argentina

## Abstract

Y-chromosomal haplogroups and the Y-SNPs defining them are relevant for the exploration of male lineages, inference of paternal ancestry, and reconstruction of migration pathways, to name a few. Currently, over 300,000 Y-SNPs have been reported, defining 20 main haplogroups. However, ascertainment bias in the investigations has led to some haplogroups being overlooked, which hinders a representative depiction of certain populations and their migration events. For migration pattern analyses of the first settlers of the Americas, the Native American main founding lineage Q-M3 needs to be further investigated to allow clear genetic differentiation of individuals of different ethnogeographic origins. To increase the resolution within this haplogroup, a total of 7.45 Mb of the Y chromosome of 59 admixed South Americans of haplogroup Q was targeted for sequencing using hybridization capture enrichment. Data were combined with 218 publicly available sequences of Central and South Americans of haplogroup Q. After rigorous data processing, variants not meeting the quality criteria were excluded and 4128 reliable Y-SNPs were reported. A total of 2224 Y-SNPs had previously unknown positions in the phylogenetic tree, and 1291 of these are novel. The phylogenetic relationships between the Y-SNPs were established using the software SNPtotree in order to report a redesigned phylogenetic tree containing 300 branches, defined by 3400 Y-SNPs. The new tree introduces 117 previously undescribed branches and is the most comprehensive phylogenetic tree of the Native American haplogroup Q lineages to date. The 214 sequences were assigned to 135 different low- to high-resolution branches, while in the previous phylogenetic tree, only 195 sequences could be sorted into 14 low-resolution branches with the same quality criteria. The improved genetic differentiation of subhaplogroup Q-M3 has a great potential to resolve migration patterns of Native Americans.

## 1. Introduction

The Y chromosome plays a big role in evolutionary, population, and forensic genetics. The importance of the Y chromosome in these fields lies in its unique combination of paternal inheritance, haploidy and lack of recombination (in the majority of chromosome), and its large size compared to mitochondrial DNA, which is the maternally inherited counterpart among the haploid markers.

The ability to look back into human history is based on the identification of Y-chromosomal SNPs (Y-SNPs) that are used to define Y-chromosomal haplogroups. Y-SNPs can be applied for paternal ancestry inference, reconstruction of migration patterns, and (in association with Y-STRs) estimation of the ages of pedigrees and time to most recent common ancestors (TMRCAs). This is possible, because related males share nearly identical Y chromosomes [1, 2]. Only when a mutation in the Y chromosome is introduced into the germline of a man will this mutation be inherited to the respective son and all male descendants of the son. Thus, haplogroups and characteristic Y-SNPs of founding ancestors are shared by modern men belonging to the same paternal lineage. More recent mutation events further diversified the Y chromosomes and formed additional branches that are characteristic for certain geographic regions, populations, and time ranges [2].

The human Y chromosome has been systematically neglected in genome-wide association studies, because it has half of the copies of the autosomes and a highly complex structure [3]. It varies considerably in length (from 45.2 to 84.9 Mb) [4] and consists of large portions of repetitive sequences, which are mostly pseudoautosomal, heterochromatic, ampliconic, and X-transposed regions, where it is difficult to identify true variants [5]. There are nine unique regions spanning 8.97 Mb [6] that can be investigated with massively parallel sequencing (MPS). MPS enables the efficient identification of large numbers of SNPs and continuously replaces methods targeting limited numbers of Y-SNPs, like the single base extension (SBE) method, which combines multiplex PCRs, SBE reaction(s), and capillary electrophoresis (CE) [7–10].

High-throughput targeted MPS can be applied to sequence Y-SNPs [11–13] and autosomal SNPs [14, 15] using PCR enrichment. Another option is hybridization capture enrichment, which uses oligonucleotides (baits) to capture the regions of interest. This method is widely used in ancient DNA analysis and enables sequencing of most regions of interest within the Y chromosome [16–21].

After three decades of research on Y-chromosomal haplogroups, more than 300,000 Y-SNPs in 20 haplogroups with nearly 50,000 subclades have been reported to date according to the Y-SNP database ISOGG Y-DNA Haplogroup Tree 2019–2020 and the YFull Ytree v10.07.00 (last accessed February 2023). The most thoroughly researched haplogroup R is represented by around 22% of all Y-SNPs reported in databases. Other haplogroups, like haplogroup Q that is widely distributed in Central and North Asia and the Americas [22], lack further resolution in the subhaplogroups. Currently, only 3%–5% of the total Y-SNPs reported in databases represent sublineages within haplogroup Q.

The low resolution is particularly evident for the lineage Q1b1a1a-M3 that is the most frequent among the founding lineages of the first settlers of the Americas [23–26], followed by Q1b1a1a1-M848 [27, 28], and the much less prevalent Native American lineage C3-M217∗ [25, 29, 30]. Despite the high genetic variability within many descendants of Native Americans belonging to lineage Q1b1a1a-M3, the lack of high-resolution Y-SNPs inside this lineage causes most Native American Y chromosomes (61%–87%) to be characterized as Q1b1a1a-M3 [11, 25, 31–33], preventing the identification and geographic allocation of high-resolution haplogroups, which are the basis for interpreting migration patterns.

In this study, we aimed to further resolve the most diffused Native American founding lineage Q1b1a1a-M3 by reporting (novel) SNPs within this lineage and their phylogenetic relationships. Variant information was obtained by sequencing targeted regions representing 7.45 Mb of the Y chromosome in males from different regions in South America and complementing this dataset with published sequencing data of other Central and South American Y chromosomes belonging to haplogroup Q.

## 2. Material and Methods

### 2.1. Ethics Statement

All samples were collected under written informed consent and approved to be used in connection to this study by the following Ethics Committees: Comité de Ética de la Investigación en Seres Humanos (CEISH-UDLA) de la Universidad de Las Américas, Ecuador (2022-ENM-001); Comité de Ética do Hospital Universitário Pedro Ernesto/Universidade do Estado do Rio de Janeiro, Brazil (CAAE: 0067.0.228.000-09 and CAAE: 53563115.2.1001.5259); Comité de Ética da Universidade Federal do Pará, Brazil (43199815.9.0000.0018); Comité de Ética en Investigaciones Biomédicas del Instituto Multidisciplinario de Biología Celular (IMBICE), Argentina (CE00023); Comité de Ética de la Fundación Favaloro, Argentina; Comité de Ética de la Universidad Nacional de Colombia; and Comité de Ética en investigación Clínica del Instituto de Previsión Social, Paraguay (2015-03-25).

### 2.2. Samples, DNA Extraction, and Quantification

Initially, a total of 2755 blood samples were collected on FTA cards (Whatman Inc., Clifton, NJ, United States) with informed consent in connection with previous studies [29, 34–38]. The DNA extraction was conducted using the BioRobot EZ1 Workstation (Qiagen, Hilden, Germany), phenol-chloroform, or standard Chelex extraction methods following the manufacturer's recommendations. DNA concentrations were determined using the Qubit™ dsDNA High Sensitivity Assay and the Qubit® 3.0 Fluorometer (Invitrogen, Carlsbad, CA, United States) according to the manufacturer's protocols. The study was conducted according to the guidelines of the Declaration of Helsinki [39].

### 2.3. Sample Selection

Y-STR profiles of unrelated admixed men from different South American regions were typed in connection to other studies [29, 34–38, 40] using standard STR kits. These covered partly or completely the following 27 Y-STRs: DYS19, DYS385, DYS389I, DYS389II, DYS390, DYS391, DYS392, DYS393, DYS437, DYS438, DYS439, DYS448, DYS449, DYS456, DYS458, DYS460, DYS481, DYS518, DYS533, DYS549, DYS570, DYS576, DYS627, DYS635, DYS643, YGATAH4, and DYF387S1. The Y-DNA Haplogroup Predictor NevGen (Nevgen.org) was applied on the Y-STR profiles to determine the most likely haplogroup. For haplogroup predictions with low confidence, up to 59 Y-SNPs were investigated using PCR-SBE-CE as previously described [37, 41–45]. Ten of the targeted SNPs belonged to haplogroup Q: M242, P36.2, M346, M3, M19, Z19319, SA01, Z19483, M557, and SA05 [46].

Among the 2755 samples of individuals from South America, 405 individuals were identified as admixed individuals with Y chromosomes that belonged to the Native American haplogroup Q. Of the 405 individuals, 59 unrelated individuals were selected for this study due to their diverse Y-STR profiles and geographic origins. Admixed individuals with a native Y chromosome were selected in an effort to encompass most of the original genetic variation that over time has been lost in Native South Americans. The individuals originated from different regions in Argentina (*N* = 24), Bolivia (*N* = 2), Brazil (*N* = 4), Chile (*N* = 2), Colombia (*N* = 17), Ecuador (*N* = 8), and Paraguay (*N* = 2). The sample selection for MPS analysis was intended to have a broad distribution of samples from different geographic regions and a high diversity of Y-STR profiles within haplogroup Q to encompass a large variation. An overview of the collection sites is included in Figure S1. The sample distributions in scatter maps (Figure S1) were generated using the scattergeo function in the package plotly v4.14.3 in Python v3.6.8.

### 2.4. Publicly Available WGS Data

Y chromosome sequences of publicly available (whole) genome sequencing data of 218 samples from Central and South American men belonging to haplogroup Q were accessed [28, 47–54] (shown in Figure S1 and Table S1). The 218 samples can be sorted in one of three categories of (i) modern admixed (ModAdmix) (*N* = 71), (ii) ancient indigenous (AncNAM) (*N* = 104), and (iii) modern indigenous (ModNAM) individuals (*N* = 43). The ancient samples are 11,000–400 years old, while the modern samples are taken from modern-day individuals.

For the data analysis, sequencing data in CRAM (or BAM) or FASTQ format was downloaded. CRAM files were decompressed to BAM files, chromosome labels were adjusted for consistency among all datasets, BAM files were indexed, and variants were called and annotated. In sequencing data of FASTQ format, adapter sequences were trimmed, and the reads were aligned to the reference genome GRCh37.p13. In the resulting BAM file, the reads were sorted, and duplicate reads were removed. After base quality recalibration, variants were called and annotated as before. More detailed information on the toolkits and commands is given in Figure S2.

### 2.5. Hybridization Capture–Based Target Enrichment

#### 2.5.1. Probe Design

Custom RNA capture probes were designed with Agilent SureDesign's SureSelect DNA software (Agilent Technologies Inc., California, United States) to cover nine unique regions within the Y chromosome (Table S2) [6] to sequence the positions that potentially include phylogenetically relevant SNPs.

The final probe set comprised two groups of probes to cover the majority of the targeted regions: Both probe sets were designed with the 90 min hybridization option for the SureSelect XT HS2 protocol and optimized performance boosting, balancing the number of probes in GC-rich regions. The first set of probes was generated with default options in the remaining parameters, while the second set had a 1× tiling, determining the probe density or coverage of target nucleotides, and the least stringent masking of repetitive regions. The second set was designed to target regions that the first set failed to cover. Details on both probe sets can be viewed in Table S3. Both sets were combined and resulted in a total of 359,954 probes with an individual probe length of 120 bp and representing a total probe size of 7.84 Mb. Using these probes, approximately 7.45 Mb of the Y chromosome was targeted for sequencing (Figure S3).

#### 2.5.2. Library Preparation and Sequencing

Libraries were prepared with a DNA input of 8.7–15 ng using Agilent's SureSelect XT HS2 Target Enrichment for Illumina Paired-End Sequencing Library kit (Agilent Technologies, California, United States) for the 59 samples (ModAdmix) following the manufacturer's protocol. The DNA was mechanically sheared using the Covaris S220 Focused Ultrasonicator (Covaris, LLC., Massachusetts, United States) using 10% duty factor, 175 W peak incident power, 200 cycles per burst, 2 × 60 s treatment time, and bath temperature of 2°C–8°C. The resulting DNA fragments were end repaired, dA-tailed, and ligated with molecular barcodes (MBCs) for distinguishing PCR duplicates. After purification, the libraries were amplified with sample-specific index primer pairs. Purified libraries were then hybridized with the RNA probes, and complementary library molecules were captured, purified, and amplified.

After pooling 16 or 24 libraries to a concentration of 1.7–2 nM, the pools were paired-end sequenced on a flow cell of type SP using the NovaSeq 6000 SP Reagent Kit v1.5 (300 cycles) and the Illumina NovaSeq 6000 System (Illumina, California, United States).

#### 2.5.3. Bioinformatic Processing of Targeted Sequencing MPS Data

Data analysis was done with a customized bioinformatic pipeline. Detailed information on the toolkits, commands, and settings is shown in Figure S4.

In this pipeline, the adapter and barcode sequences were removed, and MBCs were annotated in the FASTQ files. Reads were mapped to the reference genome GRCh37. After sorting the reads in the BAM files by coordinates and adding read group information, the BAM files from different sequencing lanes per sample were merged into one BAM file per sample. PCR deduplication was performed for a read group that shares a pair of MBCs, if there were at least two read pairs with this MBC pair (parameter “m2” in Figure S4). Two complementary single-strand consensus sequences were used to generate duplex consensus sequences. Single-strand consensus reads without a complementary partner and PCR duplicates were filtered out. Thereafter, the base quality scores were recalibrated.

For estimation of the capture specificity, on-target to off-target sequenced base ratios were calculated based on output data of the picard CollectHsMetrics tool. To smooth out fluctuations in the ratios, moving averages and variations were calculated using windows of five samples upon sorting them by increasing DNA concentration for the library preparation. The average DNA concentration for the window with the highest average variation was used to determine the concentration threshold for stable capture specificity.

For coverage analyses of the processed BAM files, the repositories samtools-1.17 [55] and bedtools-2.30.0 [56] were applied using the tools samtools depth (applying Mapping Quality 25) and bedtools genomecov.

### 2.6. Variant Calling and Reporting in All 277 Profiles

Variants were called first for the subset of 59 samples sequenced in this study and then for all 277 samples. Haplotype likelihoods for each position were calculated using bcftools mpileup, and variants within the whole Y chromosome were called in VCF files using the tool bcftools call. The minimum base quality considered was 25, read depth of one, and ploidy of one. Already known variants of the gnomAD v.3.1 database, dbSNP database (accessed February 2022), the ISOGG Y-DNA Haplogroup Tree 2019–2020 and further publications [11, 47, 57] were annotated according to the following rules: SNPs taken from ISOGG or Yfull databases were annotated with the respective SNP names. SNPs present in the dbSNP database, the gnomAD database, or other publications were labelled with the location of the variation relative to the reference genome GRCh37 and the rs-number, the comment “gnomAD,” or the SNP names given in the respective publications. Lastly, novel variants were simply annotated using the locations in the Y chromosome (GRCh37) and were assigned with unique identifiers (ZK1 to ZK1291) in Table S4. For more details on the bioinformatic pipeline, please see Figure S4.

The base positions of all variants called in the 59 profiles of this study and additional positions of haplogroup Q SNPs from the database ISOGG Y-DNA Haplogroup Tree 2019–2020 were combined and targeted in all 277 profiles using the software Yleaf v.2.2 [12].

The Yleaf position file, which states the location and possible alleles of all targeted SNPs, was changed to include the respective set of variants. Here, base calling thresholds (minimum of five reads for each base, quality threshold of 25 for each read, and base majority threshold 95%) were applied on the 59 previously investigated and 218 publicly available sequencing data. A total of 4128 variable positions were reported in all 277 samples.

To ascertain the extent of resolution increased by the 4128 variants, the haplogroups of the 277 sequences were additionally inferred using Yleaf v.3.1, which targets 129,151 SNPs from the most resolved phylogenetic tree publicly available (Yfull YTree v11.01.00).

#### 2.6.1. Determining the Phylogenic Hierarchy of Variants Using SNPtotree

Due to the rather high rates of missing data, the software SNPtotree v.1.0 [58] was applied for an initial sorting of all 4128 variants into a phylogenetic tree using default settings. The input file contained the allelic status (ancestral or derived allele) of all 4128 variable sites in all 277 samples. The software filters out variants with contradictory phylogenetic relationships based on pairwise comparisons and variants with ambiguous positions in the phylogenetic tree.

Since the software does not take into account prior knowledge on the relationships of the SNPs, the generated data was supplemented with information from the Y-DNA Haplogroup Tree 2019–2020 from ISOGG and the Yfull YTree v11.01.00 to establish the most parsimonious variant hierarchy.

In this work, we chose to use a lineage-based nomenclature, introduced in 2002 by the Y Chromosome Consortium [59] and used in the construction of the ISOGG Y-DNA Haplogroup Tree 2019–2020. Although the more recently proposed nomenclature [60] based on mutation is simpler, it does not allow inferring relationships between subhaplogroups within a lineage. For example, for the Q-M346 and Q-L53 lineages named according to the mutation-based nomenclature, it is not possible to infer whether there is a close relationship between them. However, when using the adopted nomenclature, it is possible to see that Q1b1-L53 is a sublineage of Q1b-M346. Lineage-based nomenclature includes all the information that mutation-based nomenclature provides.

## 3. Results and Discussion

A total of 277 samples were investigated in this work, comprising 59 newly sequenced individuals and publicly available sequencing data from 218 individuals. The overall 277 South American samples of haplogroup Q originated from one of three categories of (i) ModAdmix (*N* = 130), (ii) AncNAM (*N* = 104), and (iii) ModNAM individuals (*N* = 43). All newly sequenced samples were of ModAdmix origin and were selected based on a broad distribution of different geographic regions and high diversity of Y-STR profiles. For these samples, 7.45 Mb of informative regions within the Y chromosome was sequenced in a targeted sequencing approach.

### 3.1. Sequencing Data Quality of the 59 Targeted Capture Samples

For the 59 samples sequenced in this study, MBCs were used during library preparation and utilized during the bioinformatic analysis to generate duplex consensus sequences (Figure S4). Since all reads of one orientation with the same MBC (and thus copies from the same original DNA fragment molecule) were compiled to one consensus sequence, the unit “read” of the hybridization capture–based target enrichment corresponds to an unknown, but usually higher number of actually sequenced reads. In the following, we refer to the term “read” for both MBC and non-MBC-generated reads for simplicity, even though this represents an underestimation for the MBC-based reads.

For each sample, the number of typed bases that successfully aligned to the reference genome GRCh37 ranged from 14 to 217 Mb (average: 115 Mb). The fraction of aligned bases that were assigned to the Y chromosome relative to the total number of sequenced bases was 0.72 (ranging from 0.21 to 0.83) when averaged over all 59 samples. The ratio of the number of on-target to off-target bases ranged from 0.11 to 1.3 per sample, with an average of 1.00.

**Figure 1:**
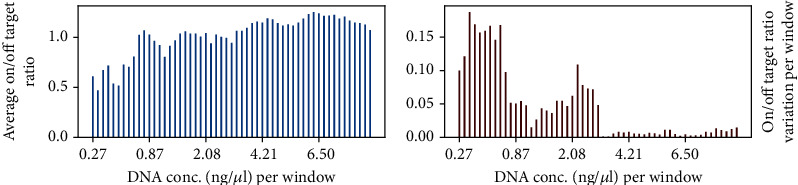
On-target to off-target ratios of sequenced bases for the 59 samples, which were sorted by increasing DNA concentrations for library preparation (abscissa, (a)), were averaged over windows of five samples for smoothing out fluctuations. Variations of on-target to off-target ratios among samples in each window (b) were highest for DNA concentrations below 0.59 ng/*μ*L.

Figure 1 presents the capture specificity based on on-target to off-target base ratios using moving averages for sample batches of five, when sorting samples according to DNA concentrations (0.2–9.2 ng/µL) on the abscissa. The input DNA concentration and capture specificity (on-target to off-target ratio) showed a positive linear correlation (correlation coefficient = 0.48, *p* value = 0.0001) when applying the Pearson correlation analysis. For lower DNA concentrations (< 0.59 ng/*μ*L), the capture specificity was highly variable, whereas it was more uniform for samples with higher concentrations.

The nucleotide positions that were sequenced with read depth ≥ 1 and with base quality ≥ 25 had median read depths of 2× to 18× per sample and 10× considering all samples. The median read depth per targeted base position ranged from 0× to 389×, with the highest median sequencing depths found in the sequencing region from 28,457,993 to 28,806,758 (Figure S5).

### 3.2. Comparison of Variants Reported in the 59 Sequenced Samples and the Whole Dataset of 277 Sequences

**Figure 2:**
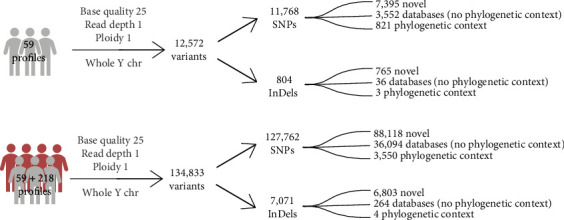
(a) Number of variants reported for the 59 samples sequenced in this study and (b) a combination of the 59 samples and 218 publicly available sequences using the same thresholds for variant calling.

When investigating the 59 samples sequenced in this study for Y-chromosomal variants, a total of 12,572 variants were observed in at least one of the samples with a minimum base quality of 25, ploidy of one, and minimum read depth of one. The majority of the variants, 11,768, were SNPs, and 804 variants were insertions and deletions (InDels) (Figure 2). Applying the same quality thresholds, 134,833 Y-chromosomal variants were reported when complementing the dataset of 59 sequences with 218 publicly available sequences. These 134,833 variants were composed of 127,762 SNPs and 7071 InDels.

**Figure 3:**
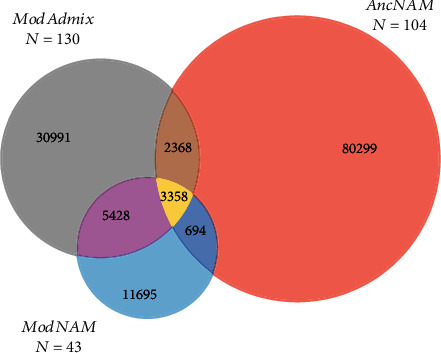
Venn diagram of the 134,833 different variants ascertained in the 277 samples subdivided into three major categories (ModAdmix [modern and admixed], AncNAM [ancient and indigenous], and ModNAM [modern and indigenous]). The numbers represent unique and shared variants among the three groups.

The 5-fold increase in the sample number from 59 to 277 surprisingly led to an 11-fold increase in reported variants. The variants were sorted according to the group of samples they were reported in ModAdmix, AncNAM, ModNAM, or (iv) any combination of the three groups (Figure 3). The subset of 59 sequences represented only ModAdmix samples, whereas the 218 publicly available sequences covered all three groups.

While most variants were exclusively found in one of the three sample groups, the biggest overlap of shared variants was reported between ModAdmix and ModNAM (7% of total variants). This is caused by the more similar evolutionary past, or a more recent common ancestor of current day admixed and indigenous individuals compared to the most recent common ancestor of the 11,000–400-year-old AncNAM individuals. In other words, the lineage of the AncNAM samples splits off earlier from the branch leading to the ModAdmix and ModNAM individuals. These two groups separated less than 400 years ago, and gene flow events between the groups may still be partially ongoing.

Generally, most of the variants (60%) were exclusively reported in AncNAM samples. This group was represented by only 38% of all analyzed samples and second in numbers to the ModAdmix (47%). It had a slightly wider geographic distribution from the Caribbean islands down to Tierra del Fuego in South Argentina, compared to the ModAdmix samples (Figure S1).

While a larger number of genetically diverse individuals highly impact the total number of reported variants, so does the number of sequenced bases. The sequences from the AncNAMs covered on average 1,188,675 bp (range 1878–13,382,470 bp) of the whole Y chromosome (with a mapping quality of 25). As expected, the sequences from modern samples covered more regions due to better sample conditions: The sequencing of ModAdmix samples covered on average 9,936,870 bp (range 2,764,105–17,631,605 bp), and the sequencing of ModNAM samples covered on average 13,158,176 bp (range 8,238,675–17,109,801 bp). Even though the AncNAM samples covered around ten times fewer bases than the modern samples, more variants were reported. The exclusively ancient SNPs were mostly (99%) without phylogenetic information, and the residual SNPs belonged to haplogroup Q or parallel haplogroups. These observations hinted towards sequencing errors fabricating a large portion of the called variants in the ancient samples. For this reason, we decided to use variants identified among the 59 sequenced samples of this study combined with well-characterized haplogroup Q SNPs from databases to refine the phylogenetic structure of haplogroup Q lineages.

### 3.3. Selection of 4128 Variants From 59 Targeted Sequencing Samples and Databases

**Figure 4:**
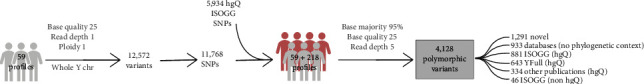
Selection of variants for phylogenetic investigations. The final set of 4128 positions was reported in all 277 sequences.

The set of 11,768 SNPs identified in the 59 samples (Figure 2) was supplemented with more than 5000 SNPs from haplogroup Q (Y-DNA Haplogroup Tree 2019–2020 from ISOGG), which were already reported and validated in other studies (Figure 4). These positions were investigated in the 59 admixed samples and in the 218 publicly available sequences using stricter variant calling criteria. The required minimum read depth was set to five, and variants were called only when they were represented by at least 95% of the reads (base majority). These settings helped to adjust for possible sequencing errors, particularly in the 218 publicly available sequences without MBCs. A total of 4128 polymorphic variants were ascertained in all 277 sequences after application of the more stringent criteria (Table S4). A total of 2267 out of 4128 variants (55%) were found in only one individual (singletons).

After cross-referencing the 4128 variants, 31% of the reported variants were, to our knowledge, not previously observed, whereas 23% were reported in databases without a phylogenetic context (only dbSNP and/or gnomAD databases), 45% were published with a phylogenetic context within haplogroup Q (Y-DNA Haplogroup Tree 2019–2020 from ISOGG, the Yfull YTree v11.01.00, and further publications [11, 28, 46, 47, 57]), and 1% were outside haplogroup Q (Figure 4). The latter were found in haplogroups parallel to lineage Q and thus characterized as recurrent. This is in accordance with a previous study that applied a similar sequence capture protocol targeting a smaller region of 3.7 Mb, where 0.92% (123 in 13,261 SNPs) of the SNPs were reported to be recurrent [19]. Studies with whole genome sequencing data resulted in a higher SNP recurrence rate (172/5865, 2.9%) [6], which may be explained by the sequencing of repetitive regions within the Y chromosome where the risk of recurrent mutations is higher [19].

### 3.4. Resolution of Haplogroup Q Phylogeny

Currently, one of the best resolved and updated phylogenetic trees of Y-SNPs can be found in the Yfull YTree v11.01.00, which includes more than 300,000 SNPs and 50,000 haplogroups (last accessed February 2023). An initial haplogroup inference of the 277 samples was based on 129,151 SNPs taken from the Yfull tree. Despite the high resolution, not all samples could be distinguished based on the defined haplogroups. For 82 sequences, haplogroup predictions failed due to data qualities below the selected thresholds: read depth ≥ 5, base quality ≥ 25, and base majority ≥ 95. For the remaining sequences, the inference of 14 different haplogroups was possible. The most frequently represented haplogroups were the very upstream branches Q1-L472 (38 samples) and Q1b1-L53 (128 samples). When the 4128 variants reported in this study were used, 214 samples were allocated to 135 different and more downstream lineages as represented in Table S5.

After combination with known phylogenetic relationships of Y-SNPs (Y-DNA Haplogroup Tree 2019–2020 from ISOGG and Yfull YTree v11.01.00), a comprehensive phylogenetic tree of the lineages represented by the 277 analyzed individuals was deducted using the software SNPtotree (available via https://github.com/ZehraKoksal/SNPtotree). The haplogroup Q tree is presented in Table S5, with 2236 out of 4128 variants incorporated into the tree. The residual variants were filtered out, due to contradictory relationships to other markers and ambiguous tree locations. Of the 2236 variants in the tree, 883 variants were novel.

The phylogenetic tree in Table S5 is to be read from the top left to the bottom right, where subbranches are presented in the cells on the lower right cell of the respective branch. The phylogenetic tree was built on the foundation of the already established haplogroup Q SNPs and additional SNPs reported in the 59 admixed samples typed in this study. Depending on where the branch-defining SNPs have been retrieved from, the branches were highlighted in different colors (see Table S5).

Out of all 104 AncNAM samples, 38 were assigned to nine mostly upstream branches in the phylogenetic tree, which is based on SNPs taken from Y-DNA Haplogroup Tree 2019–2020 from ISOGG and ModAdmix. Although ModAdmix and AncNAM share 5726 SNPs (Figure 3), only six branches are represented by samples from both groups. A portion of the 5726 SNPs may have been lost while building the phylogenetic tree due to the increased sequencing quality thresholds and contradictory information regarding relationships between SNPs. Further, the SNPs in the phylogenetic tree, which were taken from databases, seem to not include many ancient SNPs.

Three of the nine branches are characterized by SNPs exclusively found in eight of the ancient samples. The exclusively ancient SNPs may have been lost in modern individuals over time, but this demands further research. Some SNPs found in ancient samples are located at branch tips downstream of variants found in ModAdmix samples. Thus, the upstream variants may characterize very old lineages of common ancestors of the ancient and modern samples.

Variants found in ModNAM exclusively or together with ModAdmix were presented in more intertwined tree branches. This observation is assured by a more similar evolution or more recent separation between the branches leading to current day admixed and indigenous individuals, as opposed to modern and AncNAM samples. While the ancient samples are 11,000–400 years old, the ModNAM and ModAdmix split off less than 400 years ago and parts of both modern groups may still experience gene flow events.

Here, we were able to reconstruct and refine the phylogenetic structure of around 300 branches composed of 3400 variants within haplogroup Q lineages, in particular, the Q-M3 lineage. Furthermore, it was possible to identify 117 previously undescribed subhaplogroups. However, for 17 of these branches, the local tree topology was not entirely resolved, due to an incomplete overlap of genetic information retrieved from different sources. Three main branches directly downstream of lineage Q1 and 15 branches directly downstream of lineage Q1b were reported. This counteracts the resolution of polytomies and may be the result of unexplored variants and missing data. Thus, further analysis of the given variants in a new set of samples may help to refine the tree structure and resolve polytomies.

## 4. Conclusion

The application of targeted capture MPS enabled the targeted sequencing of 7.45 Mb within the Y chromosome. An initial screening for variants in all 277 samples resulted in 134,833 different variants. The majority of these were reported in indigenous and ancient samples possibly due to loss of ancient SNPs and sequencing errors of the ancient samples. Furthermore, modern-day indigenous and admixed individuals had the biggest overlap of shared variants, most likely due to a more recent evolutionary past. The samples of ancient origin diverged earlier from the branch leading to both groups of modern age. Applying strict genotyping criteria, 4128 variants were reported in the 277 samples. With the help of the SNP sorting software SNPtotree and previously established phylogenetic relationships of SNPs, 3400 variants and 300 branches were compiled into a comprehensive phylogenetic tree. The established phylogenetic tree of the Native American haplogroup Q lineage provides the highest resolution reported so far. In the future, the reported variants should be investigated in more individuals, which may help resolve some branches even further and may reflect characteristic geographic patterns of the reported variants.

## Data Availability

All relevant variant data are included in the published article and the supporting information.

## Supporting Information

Supporting Information Additional supporting information can be found online in the Supporting Information section. Figure S1. Map of the origin of all 277 haplogroup Q samples from Central and South America included in the study. Modern admixed (ModAdmix, black), ancient indigenous (AncNAM, red), and modern indigenous (ModNAM, blue) samples are presented at the place of their origin. The point size is proportional to the number of samples. Square markers indicate 59 samples genotyped in this study; round markers indicate 218 samples from public databases. Figure S2. Linux command-based bioinformatic pipeline for retrieval, preparation, and variant calling of publicly available sequencing data in the CRAM (or BAM) or FASTQ format. Figure S3. (a) Nine unique regions within the Y chromosome [6]; (b) targeted regions in this work using RNA baits. Figure S4. Bioinformatic pipeline for targeted capture data from SureSelect XT HS2 with the commands on the left side and visual representation of the commands' effects of the respective file on the right side. Figure S5. Median and cumulative read depth of the 59 samples within the nine targeted regions (orange bins). Table S1. References of the 277 samples of ModAdmix, AncNAM, or ModNAM origin. Table S2. Nine unique regions within the Y chromosome targeted in this work. Table S3. Region sizes, number of probes, and total probe size of the two designed probe groups. Table S4. Detailed information on the 4128 variants reported and targeted in this study. Table S5. Phylogenetic hierarchy of Y-chromosomal variants from the Native American haplogroup Q linages.
