# Supplementary material for: Application of Targeted Y-Chromosomal Capture Enrichment to Increase the Resolution of Native American Haplogroup Q
Source: Hum Mutat. 2024 Jul 29;2024:3046495. doi: 10.1155/2024/3046495 (PMC11918922; doi:10.1155/2024/3046495)
Supplement: Supporting Information — Additional supporting information can be found online in the Supporting Information section. Figure S1. Map of the origin of all 277 haplogroup Q samples from Central and South America included in the study. Modern admixed (ModAdmix, black), ancient indigenous (AncNAM, red), and modern indigenous (ModNAM, blue) samples are presented at the place of their origin. The point size is proportional to the number of samples. Square markers indicate 59 samples genotyped in this study; round markers indicate 218 samples from public databases. Figure S2. Linux command-based bioinformatic pipeline for retrieval, preparation, and variant calling of publicly available sequencing data in the CRAM (or BAM) or FASTQ format. Figure S3. (a) Nine unique regions within the Y chromosome [6]; (b) targeted regions in this work using RNA baits. Figure S4. Bioinformatic pipeline for targeted capture data from SureSelect XT HS2 with the commands on the left side and visual representation of the commands' effects of the respective file on the right side. Figure S5. Median and cumulative read depth of the 59 samples within the nine targeted regions (orange bins). Table S1. References of the 277 samples of ModAdmix, AncNAM, or ModNAM origin. Table S2. Nine unique regions within the Y chromosome targeted in this work. Table S3. Region sizes, number of probes, and total probe size of the two designed probe groups. Table S4. Detailed information on the 4128 variants reported and targeted in this study. Table S5. Phylogenetic hierarchy of Y-chromosomal variants from the Native American haplogroup Q linages. [file 3046495.f1.zip › Supplementary_TableS4_Variant_information_Revised.pdf]

Supplementary Table S4: Detailed information on the 4,128 variants reported and targeted in this study.

Sequence Variant Nomenclature	position in Y chr (GRCh37.p13)	Variant label	Annotation	Substitution	Ancestral allele	Derived allele
NC_000024.9:g.28804165C>A	28804165	28804165+A13718.2^^,G2a2b2a1a1b1a1a2a1a1b1a1~	A13718.2^^,G2a2b2a1a1b1a1a2a1a1b1a1~	C>A	C	A
NC_000024.9:g.14612382T>C	14612382	14612382+A4862,A00	A4862,A00	T>C	T	C
NC_000024.9:g.18952224G>C	18952224	18952224+A8557,BY1476,R1b1a1b1a1a1c2b2a1b6b	A8557,BY1476,R1b1a1b1a1a1c2b2a1b6b	G>C	G	C
NC_000024.9:g.18952225A>T	18952225	18952225+A8558,R1b1a1b1a1a1c2b2a1b6b	A8558,R1b1a1b1a1a1c2b2a1b6b	A>T	A	T
NC_000024.9:g.2839125C>A	2839125	2839125+B35,Z19439,Q1b1a1a1h1	B35,Z19439,Q1b1a1a1h1	C>A	C	A
NC_000024.9:g.2752041A>T	2752041	2752041+B46,Q1b1a1a1k2~	B46,Q1b1a1a1k2~	A>T	A	T
NC_000024.9:g.17789935G>A	17789935	17789935+BY117387,G2a2b2a1a1b1a1b2b1a~	BY117387,G2a2b2a1a1b1a1b2b1a~	G>A	G	A
NC_000024.9:g.22933398G>A	22933398	22933398+BY177,Z18036,R1b1a1b1a1a2c1a1d3c	BY177,Z18036,R1b1a1b1a1a2c1a1d3c	G>A	G	A
NC_000024.9:g.7569247G>A	7569247	7569247+BY65986	BY65986	G>A	G	A
NC_000024.9:g.22803761G>A	22803761	22803761+BZ3317,Q2b1a1b1~	BZ3317,Q2b1a1b1~	G>A	G	A
NC_000024.9:g.7543125T>A	7543125	7543125+CO10	CO10	T>A	T	A
NC_000024.9:g.21839618G>T	21839618	21839618+CO14	CO14	G>T	G	T
NC_000024.9:g.19212447A>G	19212447	19212447+CTS10021,M1148,Q1b1~	CTS10021,M1148,Q1b1~	A>G	A	G
NC_000024.9:g.19388473G>A	19388473	19388473+CTS10310	CTS10310	G>A	G	A
NC_000024.9:g.19454740G>A	19454740	19454740+CTS10434,M1151,Q1b1~	CTS10434,M1151,Q1b1~	G>A	G	A
NC_000024.9:g.22793324C>T	22793324	22793324+CTS10828,M1171,Q1b	CTS10828,M1171,Q1b	C>T	C	T
NC_000024.9:g.22954328C>G	22954328	22954328+CTS11101,M1172,Q1b1a~	CTS11101,M1172,Q1b1a~	C>G	C	G
NC_000024.9:g.23060094G>A	23060094	23060094+CTS11295,M1054,Z795,Q1b1a2	CTS11295,M1054,Z795,Q1b1a2	G>A	G	A
NC_000024.9:g.23079306G>T	23079306	23079306+CTS11350,M1174,Q1b1a~	CTS11350,M1174,Q1b1a~	G>T	G	T
NC_000024.9:g.23081524G>A	23081524	23081524+CTS11357,M925,Q1b1a1a1e	CTS11357,M925,Q1b1a1a1e	G>A	G	A
NC_000024.9:g.23154651A>T	23154651	23154651+CTS11538,M1175,Q1b	CTS11538,M1175,Q1b	A>T	A	T
NC_000024.9:g.23199656C>T	23199656	23199656+CTS11634,Q1b	CTS11634,Q1b	C>T	C	T
NC_000024.9:g.23206196C>A	23206196	23206196+CTS11663,Q1b1a1a	CTS11663,Q1b1a1a	C>A	C	A
NC_000024.9:g.23357268A>G	23357268	23357268+CTS11920,M1176,Q1b1~	CTS11920,M1176,Q1b1~	A>G	A	G
NC_000024.9:g.23391298T>G	23391298	23391298+CTS11969,M930,Q1b1a1	CTS11969,M930,Q1b1a1	T>G	T	G
NC_000024.9:g.23391307G>C	23391307	23391307+CTS11970,M931,Q1b1a1	CTS11970,M931,Q1b1a1	G>C	G	C
NC_000024.9:g.7264950C>T	7264950	7264950+CTS1199,M5063,PF1521,E1b1b1~	CTS1199,M5063,PF1521,E1b1b1~	C>T	C	T
NC_000024.9:g.7269037C>A	7269037	7269037+CTS1206,M956,Q1b	CTS1206,M956,Q1b	C>A	C	A
NC_000024.9:g.28551555T>A	28551555	28551555+CTS12266,Q1b	CTS12266,Q1b	T>A	T	A
NC_000024.9:g.28554810C>T	28554810	28554810+CTS12274,Q1b1a1~	CTS12274,Q1b1a1~	C>T	C	T
NC_000024.9:g.28587459T>C	28587459	28587459+CTS12440,E1b1a1a1c1a1a3a1d1b1b1a2~	CTS12440,E1b1a1a1c1a1a3a1d1b1b1a2~	T>C	T	C
NC_000024.9:g.7325413T>C	7325413	7325413+CTS1309,M1069,Q1b	CTS1309,M1069,Q1b	T>C	T	C
NC_000024.9:g.7343574A>C	7343574	7343574+CTS1330,M1070,Q1b1~	CTS1330,M1070,Q1b1~	A>C	A	C
NC_000024.9:g.7377856A>G	7377856	7377856+CTS1391,M1071,Q1b	CTS1391,M1071,Q1b	A>G	A	G
NC_000024.9:g.14064827G>A	14064827	14064827+CTS1780,M981,Q1b1a2	CTS1780,M981,Q1b1a2	G>A	G	A
NC_000024.9:g.14151622C>T	14151622	14151622+CTS2006,M1088,Q1	CTS2006,M1088,Q1	C>T	C	T
NC_000024.9:g.14171639C>A	14171639	14171639+CTS2068,M1089,Q1b1~	CTS2068,M1089,Q1b1~	C>A	C	A
NC_000024.9:g.14189325G>A	14189325	14189325+CTS2122,M1090,Z799,Q1b1~	CTS2122,M1090,Z799,Q1b1~	G>A	G	A
NC_000024.9:g.14198406C>T	14198406	14198406+CTS2146,M1091,Q1b	CTS2146,M1091,Q1b	C>T	C	T
NC_000024.9:g.14229525C>T	14229525	14229525+CTS2243,R1a1a1b1a3a2a1	CTS2243,R1a1a1b1a3a2a1	C>T	C	T
NC_000024.9:g.14284953G>A	14284953	14284953+CTS2385,M1093,Q1b	CTS2385,M1093,Q1b	G>A	G	A
NC_000024.9:g.14310345G>A	14310345	14310345+CTS2450,M1095,Q1b	CTS2450,M1095,Q1b	G>A	G	A
NC_000024.9:g.14370796T>C	14370796	14370796+CTS2577,M1097,Q1b1~	CTS2577,M1097,Q1b1~	T>C	T	C
NC_000024.9:g.14389497C>T	14389497	14389497+CTS2610,M848,Q1b1a1a1	CTS2610,M848,Q1b1a1a1	C>T	C	T
NC_000024.9:g.14407664C>T	14407664	14407664+CTS2656,L892,M1098,Q1b	CTS2656,L892,M1098,Q1b	C>T	C	T
NC_000024.9:g.14449243T>C	14449243	14449243+CTS2730,M988,Q1b1a2	CTS2730,M988,Q1b1a2	T>C	T	C
NC_000024.9:g.14534885C>A	14534885	14534885+CTS2869,M1099,Z800,Q1b	CTS2869,M1099,Z800,Q1b	C>A	C	A
NC_000024.9:g.14750385C>T	14750385	14750385+CTS3227,M1101,V2572,Q1b	CTS3227,M1101,V2572,Q1b	C>T	C	T
NC_000024.9:g.14767644C>G	14767644	14767644+CTS3248,M1102,V2582,Q1b	CTS3248,M1102,V2582,Q1b	C>G	C	G
NC_000024.9:g.15135698G>A	15135698	15135698+CTS3730	CTS3730	G>A	G	A
NC_000024.9:g.15201423C>T	15201423	15201423+CTS3812,M1106,Q1b	CTS3812,M1106,Q1b	C>T	C	T
NC_000024.9:g.15202608G>C	15202608	15202608+CTS3813,M1215,PF5491,P1 or K2b2a	CTS3813,M1215,PF5491,P1 or K2b2a	G>C	G	C
NC_000024.9:g.15253734C>T	15253734	15253734+CTS3905,E2b	CTS3905,E2b	C>T	C	T
NC_000024.9:g.15326406C>T	15326406	15326406+CTS4000,Q1b1a1a1h1	CTS4000,Q1b1a1a1h1	C>T	C	T
NC_000024.9:g.15540502C>T	15540502	15540502+CTS4281,O2a2b1a1a1a2a1a	CTS4281,O2a2b1a1a1a2a1a	C>T	C	T
NC_000024.9:g.15715320C>T	15715320	15715320+CTS4564,M861,Q1b1a1~	CTS4564,M861,Q1b1a1~	C>T	C	T
NC_000024.9:g.15774531G>T	15774531	15774531+CTS4695,M996,Z787,Q1b1a2~	CTS4695,M996,Z787,Q1b1a2~	G>T	G	T
NC_000024.9:g.15781062C>G	15781062	15781062+CTS4715,M1110,Z802,Q1b	CTS4715,M1110,Z802,Q1b	C>G	C	G
NC_000024.9:g.15803635C>T	15803635	15803635+CTS4765	CTS4765	C>T	C	T
NC_000024.9:g.15920365G>A	15920365	15920365+CTS4949,M1112,Q1b1~	CTS4949,M1112,Q1b1~	G>A	G	A
NC_000024.9:g.15946610T>C	15946610	15946610+CTS4995,M1113,Q1b	CTS4995,M1113,Q1b	T>C	T	C
NC_000024.9:g.16003678T>C	16003678	16003678+CTS5077,M1114,Z803,Q1b	CTS5077,M1114,Z803,Q1b	T>C	T	C
NC_000024.9:g.16019432T>C	16019432	16019432+CTS5104,M1116,Q1b1~	CTS5104,M1116,Q1b1~	T>C	T	C
NC_000024.9:g.16064245C>G	16064245	16064245+CTS5203,Q1b1~	CTS5203,Q1b1~	C>G	C	G
NC_000024.9:g.16413286C>A	16413286	16413286+CTS5647,M1120,Q1b1~	CTS5647,M1120,Q1b1~	C>A	C	A
NC_000024.9:g.16487674G>A	16487674	16487674+CTS5804,M1122,Q1	CTS5804,M1122,Q1	G>A	G	A
NC_000024.9:g.16599197G>A	16599197	16599197+CTS5992,M1123,Q1b	CTS5992,M1123,Q1b	G>A	G	A
NC_000024.9:g.16676876T>A	16676876	16676876+CTS6106,M1124,Q1b1~	CTS6106,M1124,Q1b1~	T>A	T	A
NC_000024.9:g.16841334G>A	16841334	16841334+CTS6354,M1126,Q1	CTS6354,M1126,Q1	G>A	G	A
NC_000024.9:g.16842796G>C	16842796	16842796+CTS6357,M1127,Q1b1~	CTS6357,M1127,Q1b1~	G>C	G	C
NC_000024.9:g.17151242T>C	17151242	17151242+CTS6857,M1129,Q1b1~	CTS6857,M1129,Q1b1~	T>C	T	C
NC_000024.9:g.6953167A>C	6953167	6953167+CTS686,M1066,Z797,Q1b	CTS686,M1066,Z797,Q1b	A>C	A	C
NC_000024.9:g.17159824C>T	17159824	17159824+CTS6869,M1130,Q1b1~	CTS6869,M1130,Q1b1~	C>T	C	T
NC_000024.9:g.17180544A>G	17180544	17180544+CTS6904	CTS6904	A>G	A	G
NC_000024.9:g.17317841A>T	17317841	17317841+CTS7143,M879,Z788,Q1b1a1a	CTS7143,M879,Z788,Q1b1a1a	A>T	A	T
NC_000024.9:g.17335310G>A	17335310	17335310+CTS7174,M1014,Z789,Q1b1a2	CTS7174,M1014,Z789,Q1b1a2	G>A	G	A
NC_000024.9:g.17560616A>T	17560616	17560616+CTS7611,M1134,Q1	CTS7611,M1134,Q1	A>T	A	T
NC_000024.9:g.17565152A>C	17565152	17565152+CTS7619,M1135,Q1b1a~	CTS7619,M1135,Q1b1a~	A>C	A	C
NC_000024.9:g.17625916T>G	17625916	17625916+CTS7714,M1137,Q1b	CTS7714,M1137,Q1b	T>G	T	G
NC_000024.9:g.17657530A>T	17657530	17657530+CTS7779,M881,Q1b1a1a	CTS7779,M881,Q1b1a1a	A>T	A	T
NC_000024.9:g.17673959G>A	17673959	17673959+CTS7799,M884,Q1b1a1a	CTS7799,M884,Q1b1a1a	G>A	G	A
NC_000024.9:g.17739031G>T	17739031	17739031+CTS7915,M1017,Q1b1a2~	CTS7915,M1017,Q1b1a2~	G>T	G	T
NC_000024.9:g.17795068A>C	17795068	17795068+CTS8010,M1138,Q1b	CTS8010,M1138,Q1b	A>C	A	C
NC_000024.9:g.17845925G>A	17845925	17845925+CTS8129,M885,Q1b1a1~	CTS8129,M885,Q1b1a1~	G>A	G	A
NC_000024.9:g.17935153C>T	17935153	17935153+CTS8328,M1139,Q1b	CTS8328,M1139,Q1b	C>T	C	T
NC_000024.9:g.17979120G>T	17979120	17979120+CTS8405,Q1b	CTS8405,Q1b	G>T	G	T
NC_000024.9:g.18021716G>A	18021716	18021716+CTS8430,Q1b1a1a	CTS8430,Q1b1a1a	G>A	G	A
NC_000024.9:g.18043582G>A	18043582	18043582+CTS8475,M886,Q1b1a1a	CTS8475,M886,Q1b1a1a	G>A	G	A
NC_000024.9:g.7049495G>A	7049495	7049495+CTS856,M826,D1a2a1c1c~	CTS856,M826,D1a2a1c1c~	G>A	G	A
NC_000024.9:g.18611597T>A	18611597	18611597+CTS9004,M1143,Q1b1~	CTS9004,M1143,Q1b1~	T>A	T	A
NC_000024.9:g.18928670G>A	18928670	18928670+CTS9489,M1146,E1a2a1b1	CTS9489,M1146,E1a2a1b1	G>A	G	A
NC_000024.9:g.7101931G>A	7101931	7101931+CTS950,M1068,Q1b	CTS950,M1068,Q1b	G>A	G	A
NC_000024.9:g.18952261T>C	18952261	18952261+CTS9539,M7348,R1b1a1b1a1a1c2b2a1b6b	CTS9539,M7348,R1b1a1b1a1a1c2b2a1b6b	T>C	T	C
NC_000024.9:g.2727311T>A	2727311	2727311+CTS97,M1062,V1032,Q1	CTS97,M1062,V1032,Q1	T>A	T	A
NC_000024.9:g.19072634T>G	19072634	19072634+CTS9796,M1147,Q1b	CTS9796,M1147,Q1b	T>G	T	G
NC_000024.9:g.19078157C>T	19078157	19078157+CTS9805,R1b1a1b1a1a2c1a4b7~	CTS9805,R1b1a1b1a1a2c1a4b7~	C>T	C	T
NC_000024.9:g.8039511G>A	8039511	8039511+F108	F108	G>A	G	A
NC_000024.9:g.6930969C>G	6930969	6930969+F15462,O2a2a1a2a1a1b1a	F15462,O2a2a1a2a1a1b1a	C>G	C	G
NC_000024.9:g.14776615A>T	14776615	14776615+F1836,Q2a	F1836,Q2a	A>T	A	T

NC_000024.9:g.14791432G>A	14791432	14791432+F1839,Q2a	F1839,Q2a	G>A	G	A
NC_000024.9:g.14899742G>A	14899742	14899742+F1858,Q2	F1858,Q2	G>A	G	A
NC_000024.9:g.15033183G>A	15033183	15033183+F1893,Q1b2b1b2a~	F1893,Q1b2b1b2a~	G>A	G	A
NC_000024.9:g.15265941A>T	15265941	15265941+F1924,Q2	F1924,Q2	A>T	A	T
NC_000024.9:g.15585403G>A	15585403	15585403+F1974,Q2	F1974,Q2	G>A	G	A
NC_000024.9:g.15880878T>A	15880878	15880878+F2023,Q2	F2023,Q2	T>A	T	A
NC_000024.9:g.16305155T>C	16305155	16305155+F2122,L942,M1118,Q1b	F2122,L942,M1118,Q1b	T>C	T	C
NC_000024.9:g.17305863T>G	17305863	17305863+F2440,Q2	F2440,Q2	T>G	T	G
NC_000024.9:g.17359287A>T	17359287	17359287+F2458,M1132,Q1b	F2458,M1132,Q1b	A>T	A	T
NC_000024.9:g.17972321T>A	17972321	17972321+F2657,Q2	F2657,Q2	T>A	T	A
NC_000024.9:g.18553194C>T	18553194	18553194+F2771	F2771	C>T	C	T
NC_000024.9:g.18575570C>T	18575570	18575570+F2777,Q2	F2777,Q2	C>T	C	T
NC_000024.9:g.18610642A>T	18610642	18610642+F2790,M1142,V3324,Q1	F2790,M1142,V3324,Q1	A>T	A	T
NC_000024.9:g.18775212T>A	18775212	18775212+F2840,M1144,V3486,Q1	F2840,M1144,V3486,Q1	T>A	T	A
NC_000024.9:g.18809307C>T	18809307	18809307+F2851,Q2	F2851,Q2	C>T	C	T
NC_000024.9:g.18858950A>G	18858950	18858950+F2877,Q2a	F2877,Q2a	A>G	A	G
NC_000024.9:g.18912103A>C	18912103	18912103+F2894,Q2	F2894,Q2	A>C	A	C
NC_000024.9:g.19089909G>T	19089909	19089909+F2934,Q2	F2934,Q2	G>T	G	T
NC_000024.9:g.19566131A>C	19566131	19566131+F3084,Q2	F3084,Q2	A>C	A	C
NC_000024.9:g.21113757C>T	21113757	21113757+F3121,Q2	F3121,Q2	C>T	C	T
NC_000024.9:g.21390848C>T	21390848	21390848+F3193,Q2	F3193,Q2	C>T	C	T
NC_000024.9:g.21441448C>T	21441448	21441448+F3207,Q2	F3207,Q2	C>T	C	T
NC_000024.9:g.23021064C>A	23021064	23021064+F3389,Q2	F3389,Q2	C>A	C	A
NC_000024.9:g.15033712C>T	15033712	15033712+F3773,L474,Q1b	F3773,L474,Q1b	C>T	C	T
NC_000024.9:g.2692142T>C	2692142	2692142+F711,L612,Q2	F711,L612,Q2	T>C	T	C
NC_000024.9:g.2713850A>G	2713850	2713850+F713,Q2	F713,Q2	A>G	A	G
NC_000024.9:g.6679787A>C	6679787	6679787+F803,Q2	F803,Q2	A>C	A	C
NC_000024.9:g.6718686T>C	6718686	6718686+F815,Q2	F815,Q2	T>C	T	C
NC_000024.9:g.7713719C>A	7713719	7713719+FGC12206	FGC12206	C>A	C	A
NC_000024.9:g.2806676A>G	2806676	2806676+FGC1770,Y2012,Q2a	FGC1770,Y2012,Q2a	A>G	A	G
NC_000024.9:g.6746675T>C	6746675	6746675+FGC1792,Y2035,Q2a	FGC1792,Y2035,Q2a	T>C	T	C
NC_000024.9:g.6986250T>C	6986250	6986250+FGC1794,Y2037,Q2a	FGC1794,Y2037,Q2a	T>C	T	C
NC_000024.9:g.7219594T>C	7219594	7219594+FGC1800,Y2121,Q2a1a	FGC1800,Y2121,Q2a1a	T>C	T	C
NC_000024.9:g.7408851C>A	7408851	7408851+FGC1802,Y2122,Q2a1a	FGC1802,Y2122,Q2a1a	C>A	C	A
NC_000024.9:g.7437821C>G	7437821	7437821+FGC1804,Y2045,Q2a	FGC1804,Y2045,Q2a	C>G	C	G
NC_000024.9:g.14045736T>C	14045736	14045736+FGC1826,Y2129,Q2a1a	FGC1826,Y2129,Q2a1a	T>C	T	C
NC_000024.9:g.14202870A>G	14202870	14202870+FGC1828,Y2130,Q2a1a	FGC1828,Y2130,Q2a1a	A>G	A	G
NC_000024.9:g.14285880C>G	14285880	14285880+FGC1829,Y2131,Q2a1a	FGC1829,Y2131,Q2a1a	C>G	C	G
NC_000024.9:g.14293849T>A	14293849	14293849+FGC1830,Y2061,Q2a	FGC1830,Y2061,Q2a	T>A	T	A
NC_000024.9:g.15614105C>G	15614105	15614105+FGC1840,Y2134,Q2a1a	FGC1840,Y2134,Q2a1a	C>G	C	G
NC_000024.9:g.15699493C>T	15699493	15699493+FGC1841,Y2068,Q2a	FGC1841,Y2068,Q2a	C>T	C	T
NC_000024.9:g.17686883A>G	17686883	17686883+FGC1847,Y2137,Q2a1a	FGC1847,Y2137,Q2a1a	A>G	A	G
NC_000024.9:g.18575106G>A	18575106	18575106+FGC1853,Y2141,Q2a1a	FGC1853,Y2141,Q2a1a	G>A	G	A
NC_000024.9:g.18714407C>A	18714407	18714407+FGC1854,Y2076,Q2a	FGC1854,Y2076,Q2a	C>A	C	A
NC_000024.9:g.18803642T>G	18803642	18803642+FGC1855,Y2080,Q2a	FGC1855,Y2080,Q2a	T>G	T	G
NC_000024.9:g.21479863G>A	21479863	21479863+FGC1862,Y2085,Q2a	FGC1862,Y2085,Q2a	G>A	G	A
NC_000024.9:g.22022365A>G	22022365	22022365+FGC1865,Y2088,Q2a	FGC1865,Y2088,Q2a	A>G	A	G
NC_000024.9:g.22931328T>A	22931328	22931328+FGC1869,Y2091,Q2a1	FGC1869,Y2091,Q2a1	T>A	T	A
NC_000024.9:g.23042385C>A	23042385	23042385+FGC1871,Y2146,Q2a1a	FGC1871,Y2146,Q2a1a	C>A	C	A
NC_000024.9:g.23053626A>G	23053626	23053626+FGC1872,Y2092,Q2a	FGC1872,Y2092,Q2a	A>G	A	G
NC_000024.9:g.23166596T>C	23166596	23166596+FGC1874,Y2094,Q2a1	FGC1874,Y2094,Q2a1	T>C	T	C
NC_000024.9:g.18043999G>A	18043999	18043999+FGC1915,Y2211,Q2a1a1a1	FGC1915,Y2211,Q2a1a1a1	G>A	G	A
NC_000024.9:g.18046486T>C	18046486	18046486+FGC1916,Y2210,Q2a1a1a1	FGC1916,Y2210,Q2a1a1a1	T>C	T	C
NC_000024.9:g.18051798A>C	18051798	18051798+FGC1917,Y2209,Q2a1a1a	FGC1917,Y2209,Q2a1a1a	A>C	A	C
NC_000024.9:g.22588598C>T	22588598	22588598+FGC1929,Y2200,Q2a1a1a1a	FGC1929,Y2200,Q2a1a1a1a	C>T	C	T
NC_000024.9:g.9850515T>C	9850515	9850515+FGC22000,M8378,Z7235,ZS3800,C1a1~	FGC22000,M8378,Z7235,ZS3800,C1a1~	T>C	T	C
NC_000024.9:g.16338625A>G	16338625	16338625+FGC26636,YP3066,A00	FGC26636,YP3066,A00	A>G	A	G
NC_000024.9:g.16645948G>A	16645948	16645948+FGC28906,Y10429,C2a1a1b1	FGC28906,Y10429,C2a1a1b1	G>A	G	A
NC_000024.9:g.19301608C>T	19301608	19301608+FGC4458	FGC4458	C>T	C	T
NC_000024.9:g.16071882T>C	16071882	16071882+FGC47532,Q1b1a2b~	FGC47532,Q1b1a2b~	T>C	T	C
NC_000024.9:g.15540398G>A	15540398	15540398+FGC4884,Y2988,Q2a1c1a	FGC4884,Y2988,Q2a1c1a	G>A	G	A
NC_000024.9:g.19448045C>A	19448045	19448045+FGC7652,Y1992,Q2	FGC7652,Y1992,Q2	C>A	C	A
NC_000024.9:g.22816809G>A	22816809	22816809+FGC7659,Y1996,Q2	FGC7659,Y1996,Q2	G>A	G	A
NC_000024.9:g.21524591G>A	21524591	21524591+FGC8302^,Z19501^,Q1b1a1a1h1	FGC8302^,Z19501^,Q1b1a1a1h1	G>A	G	A
NC_000024.9:g.15755640G>A	15755640	15755640+FGC8456,Y4303,Q1b1a1a2	FGC8456,Y4303,Q1b1a1a2	G>A	G	A
NC_000024.9:g.16802315T>C	16802315	16802315+FGC8461,Y4308,Q1b1a1a2	FGC8461,Y4308,Q1b1a1a2	T>C	T	C
NC_000024.9:g.17615150A>G	17615150	17615150+FGC8469,Y4276,Q1b1a1a2	FGC8469,Y4276,Q1b1a1a2	A>G	A	G
NC_000024.9:g.17946696C>T	17946696	17946696+FGC8472,Y6163,Q1b1a1a2	FGC8472,Y6163,Q1b1a1a2	C>T	C	T
NC_000024.9:g.14000379G>A	14000379	14000379+FT149106,G2a2b1b1a1b1d2~	FT149106,G2a2b1b1a1b1d2~	G>A	G	A
NC_000024.9:g.14472977C>T	14472977	14472977+FT192969.2,MF136585.2,S1a2a	FT192969.2,MF136585.2,S1a2a	C>T	C	T
NC_000024.9:g.8098860A>G	8098860	8098860+GG169	GG169	A>G	A	G
NC_000024.9:g.14198967T>C	14198967	14198967+GMP26	GMP26	T>C	T	C
NC_000024.9:g.7267390G>T	7267390	7267390+GMP35	GMP35	G>T	G	T
NC_000024.9:g.14886685T>C	14886685	14886685+GMP37	GMP37	T>C	T	C
NC_000024.9:g.6631920A>T	6631920	6631920+GMP41	GMP41	A>T	A	T
NC_000024.9:g.6965772G>T	6965772	6965772+GMP46	GMP46	G>T	G	T
NC_000024.9:g.7419588T>G	7419588	7419588+GMP47	GMP47	T>G	T	G
NC_000024.9:g.14044033C>T	14044033	14044033+GMP50	GMP50	C>T	C	T
NC_000024.9:g.14587968A>G	14587968	14587968+GMP51	GMP51	A>G	A	G
NC_000024.9:g.6678425T>A	6678425	6678425+GMP52	GMP52	T>A	T	A
NC_000024.9:g.7218975A>G	7218975	7218975+GMP65	GMP65	A>G	A	G
NC_000024.9:g.14754418C>G	14754418	14754418+GMP66	GMP66	C>G	C	G
NC_000024.9:g.16835476T>C	16835476	16835476+GMP67	GMP67	T>C	T	C
NC_000024.9:g.14196672A>T	14196672	14196672+GMP71	GMP71	A>T	A	T
NC_000024.9:g.13595103G>A	13595103	13595103+gnomad	gnomad	G>A	G	A
NC_000024.9:g.14163771T>A	14163771	14163771+gnomad	gnomad	T>A	T	A
NC_000024.9:g.14163825C>G	14163825	14163825+gnomad	gnomad	C>G	C	G
NC_000024.9:g.14169337A>G	14169337	14169337+gnomad	gnomad	A>G	A	G
NC_000024.9:g.14174143A>G	14174143	14174143+gnomad	gnomad	A>G	A	G
NC_000024.9:g.14177954G>A	14177954	14177954+gnomad	gnomad	G>A	G	A
NC_000024.9:g.14194788T>C	14194788	14194788+gnomad	gnomad	T>C	T	C
NC_000024.9:g.14234790A>T	14234790	14234790+gnomad	gnomad	A>T	A	T
NC_000024.9:g.14275986G>A	14275986	14275986+gnomad	gnomad	G>A	G	A
NC_000024.9:g.14281438A>G	14281438	14281438+gnomad	gnomad	A>G	A	G
NC_000024.9:g.14300327G>A	14300327	14300327+gnomad	gnomad	G>A	G	A
NC_000024.9:g.14305590A>C	14305590	14305590+gnomad	gnomad	A>C	A	C
NC_000024.9:g.14342917C>T	14342917	14342917+gnomad	gnomad	C>T	C	T
NC_000024.9:g.14352018C>T	14352018	14352018+gnomad	gnomad	C>T	C	T
NC_000024.9:g.14370715G>A	14370715	14370715+gnomad	gnomad	G>A	G	A
NC_000024.9:g.14371755G>A	14371755	14371755+gnomad	gnomad	G>A	G	A
NC_000024.9:g.14418467G>A	14418467	14418467+gnomad	gnomad	G>A	G	A
NC_000024.9:g.14424794C>A	14424794	14424794+gnomad	gnomad	C>A	C	A
NC_000024.9:g.14426596C>T	14426596	14426596+gnomad	gnomad	C>T	C	T
NC_000024.9:g.14483506T>C	14483506	14483506+gnomad	gnomad	T>C	T	C

NC_000024.9:g.14483848G>A	14483848	14483848+gnomad	gnomad		G>A	G	A
NC_000024.9:g.14534530C>T	14534530	14534530+gnomad	gnomad		C>T	C	T
NC_000024.9:g.14542409C>T	14542409	14542409+gnomad	gnomad		C>T	C	T
NC_000024.9:g.14582984G>A	14582984	14582984+gnomad	gnomad		G>A	G	A
NC_000024.9:g.14596302A>G	14596302	14596302+gnomad	gnomad		A>G	A	G
NC_000024.9:g.14598021C>T	14598021	14598021+gnomad	gnomad		C>T	C	T
NC_000024.9:g.14600040G>A	14600040	14600040+gnomad	gnomad		G>A	G	A
NC_000024.9:g.14628951T>C	14628951	14628951+gnomad	gnomad		T>C	T	C
NC_000024.9:g.14709384G>A	14709384	14709384+gnomad	gnomad		G>A	G	A
NC_000024.9:g.14710855C>A	14710855	14710855+gnomad	gnomad		C>A	C	A
NC_000024.9:g.14734946G>A	14734946	14734946+gnomad	gnomad		G>A	G	A
NC_000024.9:g.14741992G>A	14741992	14741992+gnomad	gnomad		G>A	G	A
NC_000024.9:g.14757678C>T	14757678	14757678+gnomad	gnomad		C>T	C	T
NC_000024.9:g.14773942C>T	14773942	14773942+gnomad	gnomad		C>T	C	T
NC_000024.9:g.14788374C>T	14788374	14788374+gnomad	gnomad		C>T	C	T
NC_000024.9:g.14812907G>A	14812907	14812907+gnomad	gnomad		G>A	G	A
NC_000024.9:g.14905267A>G	14905267	14905267+gnomad	gnomad		A>G	A	G
NC_000024.9:g.14961436C>T	14961436	14961436+gnomad	gnomad		C>T	C	T
NC_000024.9:g.15097144C>G	15097144	15097144+gnomad	gnomad		C>G	C	G
NC_000024.9:g.15125539G>T	15125539	15125539+gnomad	gnomad		G>T	G	T
NC_000024.9:g.15128969G>A	15128969	15128969+gnomad	gnomad		G>A	G	A
NC_000024.9:g.15165439G>A	15165439	15165439+gnomad	gnomad		G>A	G	A
NC_000024.9:g.15165935A>C	15165935	15165935+gnomad	gnomad		A>C	A	C
NC_000024.9:g.15181120C>G	15181120	15181120+gnomad	gnomad		C>G	C	G
NC_000024.9:g.15232425C>T	15232425	15232425+gnomad	gnomad		C>T	C	T
NC_000024.9:g.15259627C>G	15259627	15259627+gnomad	gnomad		C>G	C	G
NC_000024.9:g.15287276G>T	15287276	15287276+gnomad	gnomad		G>T	G	T
NC_000024.9:g.15300018A>G	15300018	15300018+gnomad	gnomad		A>G	A	G
NC_000024.9:g.15314620C>T	15314620	15314620+gnomad	gnomad		C>T	C	T
NC_000024.9:g.15366830C>T	15366830	15366830+gnomad	gnomad		C>T	C	T
NC_000024.9:g.15378790G>A	15378790	15378790+gnomad	gnomad		G>A	G	A
NC_000024.9:g.15436000T>C	15436000	15436000+gnomad	gnomad		T>C	T	C
NC_000024.9:g.15455055G>A	15455055	15455055+gnomad	gnomad		G>A	G	A
NC_000024.9:g.15461745A>G	15461745	15461745+gnomad	gnomad		A>G	A	G
NC_000024.9:g.15517586G>A	15517586	15517586+gnomad	gnomad		G>A	G	A
NC_000024.9:g.15541106T>C	15541106	15541106+gnomad	gnomad		T>C	T	C
NC_000024.9:g.15563985A>C	15563985	15563985+gnomad	gnomad		A>C	A	C
NC_000024.9:g.15575320C>T	15575320	15575320+gnomad	gnomad		C>T	C	T
NC_000024.9:g.15581903A>C	15581903	15581903+gnomad	gnomad		A>C	A	C
NC_000024.9:g.15594686T>G	15594686	15594686+gnomad	gnomad		T>G	T	G
NC_000024.9:g.15611177G>A	15611177	15611177+gnomad	gnomad		G>A	G	A
NC_000024.9:g.15617382G>A	15617382	15617382+gnomad	gnomad		G>A	G	A
NC_000024.9:g.15618111G>T	15618111	15618111+gnomad	gnomad		G>T	G	T
NC_000024.9:g.15655756G>T	15655756	15655756+gnomad	gnomad		G>T	G	T
NC_000024.9:g.15668335C>A	15668335	15668335+gnomad	gnomad		C>A	C	A
NC_000024.9:g.15682590G>A	15682590	15682590+gnomad	gnomad		G>A	G	A
NC_000024.9:g.15684428C>T	15684428	15684428+gnomad	gnomad		C>T	C	T
NC_000024.9:g.15703917T>G	15703917	15703917+gnomad	gnomad		T>G	T	G
NC_000024.9:g.15731956G>A	15731956	15731956+gnomad	gnomad		G>A	G	A
NC_000024.9:g.15738919C>T	15738919	15738919+gnomad	gnomad		C>T	C	T
NC_000024.9:g.15740089G>A	15740089	15740089+gnomad	gnomad		G>A	G	A
NC_000024.9:g.15788539T>C	15788539	15788539+gnomad	gnomad		T>C	T	C
NC_000024.9:g.15822014G>A	15822014	15822014+gnomad	gnomad		G>A	G	A
NC_000024.9:g.15896497A>G	15896497	15896497+gnomad	gnomad		A>G	A	G
NC_000024.9:g.15939394C>T	15939394	15939394+gnomad	gnomad		C>T	C	T
NC_000024.9:g.15981102A>G	15981102	15981102+gnomad	gnomad		A>G	A	G
NC_000024.9:g.15992003T>C	15992003	15992003+gnomad	gnomad		T>C	T	C
NC_000024.9:g.15997660C>T	15997660	15997660+gnomad	gnomad		C>T	C	T
NC_000024.9:g.16076128G>T	16076128	16076128+gnomad	gnomad		G>T	G	T
NC_000024.9:g.16171559C>T	16171559	16171559+gnomad	gnomad		C>T	C	T
NC_000024.9:g.16183818C>A	16183818	16183818+gnomad	gnomad		C>A	C	A
NC_000024.9:g.16229002G>A	16229002	16229002+gnomad	gnomad		G>A	G	A
NC_000024.9:g.16240950C>T	16240950	16240950+gnomad	gnomad		C>T	C	T
NC_000024.9:g.16262608C>T	16262608	16262608+gnomad	gnomad		C>T	C	T
NC_000024.9:g.16299022G>A	16299022	16299022+gnomad	gnomad		G>A	G	A
NC_000024.9:g.16317648A>T	16317648	16317648+gnomad	gnomad		A>T	A	T
NC_000024.9:g.16345922C>A	16345922	16345922+gnomad	gnomad		C>A	C	A
NC_000024.9:g.16375565G>A	16375565	16375565+gnomad	gnomad		G>A	G	A
NC_000024.9:g.16385437G>A	16385437	16385437+gnomad	gnomad		G>A	G	A
NC_000024.9:g.16412520C>A	16412520	16412520+gnomad	gnomad		C>A	C	A
NC_000024.9:g.16416952T>C	16416952	16416952+gnomad	gnomad		T>C	T	C
NC_000024.9:g.16423917C>T	16423917	16423917+gnomad	gnomad		C>T	C	T
NC_000024.9:g.16459507A>G	16459507	16459507+gnomad	gnomad		A>G	A	G
NC_000024.9:g.16475328G>T	16475328	16475328+gnomad	gnomad		G>T	G	T
NC_000024.9:g.16489336G>C	16489336	16489336+gnomad	gnomad		G>C	G	C
NC_000024.9:g.16510518A>G	16510518	16510518+gnomad	gnomad		A>G	A	G
NC_000024.9:g.16522552A>G	16522552	16522552+gnomad	gnomad		A>G	A	G
NC_000024.9:g.16627316G>A	16627316	16627316+gnomad	gnomad		G>A	G	A
NC_000024.9:g.16628087T>C	16628087	16628087+gnomad	gnomad		T>C	T	C
NC_000024.9:g.16630859G>A	16630859	16630859+gnomad	gnomad		G>A	G	A
NC_000024.9:g.16630883G>A	16630883	16630883+gnomad	gnomad		G>A	G	A
NC_000024.9:g.16632888G>A	16632888	16632888+gnomad	gnomad		G>A	G	A
NC_000024.9:g.16648378C>T	16648378	16648378+gnomad	gnomad		C>T	C	T
NC_000024.9:g.16713792G>A	16713792	16713792+gnomad	gnomad		G>A	G	A
NC_000024.9:g.16757197G>A	16757197	16757197+gnomad	gnomad		G>A	G	A
NC_000024.9:g.16781610A>G	16781610	16781610+gnomad	gnomad		A>G	A	G
NC_000024.9:g.16792979C>T	16792979	16792979+gnomad	gnomad		C>T	C	T
NC_000024.9:g.16819433G>A	16819433	16819433+gnomad	gnomad		G>A	G	A
NC_000024.9:g.16864576C>T	16864576	16864576+gnomad	gnomad		C>T	C	T
NC_000024.9:g.16923455C>T	16923455	16923455+gnomad	gnomad		C>T	C	T
NC_000024.9:g.16927159G>A	16927159	16927159+gnomad	gnomad		G>A	G	A
NC_000024.9:g.17010630T>C	17010630	17010630+gnomad	gnomad		T>C	T	C
NC_000024.9:g.17049279C>T	17049279	17049279+gnomad	gnomad		C>T	C	T
NC_000024.9:g.17053136A>T	17053136	17053136+gnomad	gnomad		A>T	A	T
NC_000024.9:g.17074115C>T	17074115	17074115+gnomad	gnomad		C>T	C	T
NC_000024.9:g.17097777A>T	17097777	17097777+gnomad	gnomad		A>T	A	T
NC_000024.9:g.17131623A>T	17131623	17131623+gnomad	gnomad		A>T	A	T
NC_000024.9:g.17137687A>G	17137687	17137687+gnomad	gnomad		A>G	A	G
NC_000024.9:g.17138238T>C	17138238	17138238+gnomad	gnomad		T>C	T	C
NC_000024.9:g.17167060T>C	17167060	17167060+gnomad	gnomad		T>C	T	C
NC_000024.9:g.17193728G>A	17193728	17193728+gnomad	gnomad		G>A	G	A
NC_000024.9:g.17252589C>G	17252589	17252589+gnomad	gnomad		C>G	C	G
NC_000024.9:g.17271552A>G	17271552	17271552+gnomad	gnomad		A>G	A	G
NC_000024.9:g.17327945T>G	17327945	17327945+gnomad	gnomad		T>G	T	G

NC_000024.9:g.17345520T>C	17345520	17345520+gnomad	gnomad	T>C	T	C
NC_000024.9:g.17348565G>A	17348565	17348565+gnomad	gnomad	G>A	G	A
NC_000024.9:g.17352306G>A	17352306	17352306+gnomad	gnomad	G>A	G	A
NC_000024.9:g.17355090C>G	17355090	17355090+gnomad	gnomad	C>G	C	G
NC_000024.9:g.17369401G>A	17369401	17369401+gnomad	gnomad	G>A	G	A
NC_000024.9:g.17375351T>C	17375351	17375351+gnomad	gnomad	T>C	T	C
NC_000024.9:g.17389466A>C	17389466	17389466+gnomad	gnomad	A>C	A	C
NC_000024.9:g.17411518C>T	17411518	17411518+gnomad	gnomad	C>T	C	T
NC_000024.9:g.17412959G>T	17412959	17412959+gnomad	gnomad	G>T	G	T
NC_000024.9:g.17418633A>G	17418633	17418633+gnomad	gnomad	A>G	A	G
NC_000024.9:g.17436462A>G	17436462	17436462+gnomad	gnomad	A>G	A	G
NC_000024.9:g.17744437C>T	17744437	17744437+gnomad	gnomad	C>T	C	T
NC_000024.9:g.17754573C>T	17754573	17754573+gnomad	gnomad	C>T	C	T
NC_000024.9:g.17781168C>A	17781168	17781168+gnomad	gnomad	C>A	C	A
NC_000024.9:g.17801672T>C	17801672	17801672+gnomad	gnomad	T>C	T	C
NC_000024.9:g.17840414C>T	17840414	17840414+gnomad	gnomad	C>T	C	T
NC_000024.9:g.17877230G>C	17877230	17877230+gnomad	gnomad	G>C	G	C
NC_000024.9:g.17879297C>T	17879297	17879297+gnomad	gnomad	C>T	C	T
NC_000024.9:g.17964789A>G	17964789	17964789+gnomad	gnomad	A>G	A	G
NC_000024.9:g.17976674C>A	17976674	17976674+gnomad	gnomad	C>A	C	A
NC_000024.9:g.18025414C>T	18025414	18025414+gnomad	gnomad	C>T	C	T
NC_000024.9:g.18045244C>G	18045244	18045244+gnomad	gnomad	C>G	C	G
NC_000024.9:g.18070823C>A	18070823	18070823+gnomad	gnomad	C>A	C	A
NC_000024.9:g.18076781G>A	18076781	18076781+gnomad	gnomad	G>A	G	A
NC_000024.9:g.18082237T>C	18082237	18082237+gnomad	gnomad	T>C	T	C
NC_000024.9:g.18094967T>C	18094967	18094967+gnomad	gnomad	T>C	T	C
NC_000024.9:g.18128892A>G	18128892	18128892+gnomad	gnomad	A>G	A	G
NC_000024.9:g.18210274T>A	18210274	18210274+gnomad	gnomad	T>A	T	A
NC_000024.9:g.18222521G>C	18222521	18222521+gnomad	gnomad	G>C	G	C
NC_000024.9:g.18256990C>T	18256990	18256990+gnomad	gnomad	C>T	C	T
NC_000024.9:g.18258631C>T	18258631	18258631+gnomad	gnomad	C>T	C	T
NC_000024.9:g.18538536C>T	18538536	18538536+gnomad	gnomad	C>T	C	T
NC_000024.9:g.18616994A>G	18616994	18616994+gnomad	gnomad	A>G	A	G
NC_000024.9:g.18640233C>T	18640233	18640233+gnomad	gnomad	C>T	C	T
NC_000024.9:g.18662343C>A	18662343	18662343+gnomad	gnomad	C>A	C	A
NC_000024.9:g.18693423C>T	18693423	18693423+gnomad	gnomad	C>T	C	T
NC_000024.9:g.18722508G>A	18722508	18722508+gnomad	gnomad	G>A	G	A
NC_000024.9:g.18743512G>C	18743512	18743512+gnomad	gnomad	G>C	G	C
NC_000024.9:g.18816349T>G	18816349	18816349+gnomad	gnomad	T>G	T	G
NC_000024.9:g.18831725A>G	18831725	18831725+gnomad	gnomad	A>G	A	G
NC_000024.9:g.18859043G>A	18859043	18859043+gnomad	gnomad	G>A	G	A
NC_000024.9:g.18859866A>G	18859866	18859866+gnomad	gnomad	A>G	A	G
NC_000024.9:g.18866755A>G	18866755	18866755+gnomad	gnomad	A>G	A	G
NC_000024.9:g.18889910A>G	18889910	18889910+gnomad	gnomad	A>G	A	G
NC_000024.9:g.18894580A>G	18894580	18894580+gnomad	gnomad	A>G	A	G
NC_000024.9:g.18909272G>A	18909272	18909272+gnomad	gnomad	G>A	G	A
NC_000024.9:g.18989224C>A	18989224	18989224+gnomad	gnomad	C>A	C	A
NC_000024.9:g.19001403C>G	19001403	19001403+gnomad	gnomad	C>G	C	G
NC_000024.9:g.19005156A>C	19005156	19005156+gnomad	gnomad	A>C	A	C
NC_000024.9:g.19008994T>G	19008994	19008994+gnomad	gnomad	T>G	T	G
NC_000024.9:g.19024373C>G	19024373	19024373+gnomad	gnomad	C>G	C	G
NC_000024.9:g.19034247G>T	19034247	19034247+gnomad	gnomad	G>T	G	T
NC_000024.9:g.19050691G>A	19050691	19050691+gnomad	gnomad	G>A	G	A
NC_000024.9:g.19078939G>A	19078939	19078939+gnomad	gnomad	G>A	G	A
NC_000024.9:g.19096513T>A	19096513	19096513+gnomad	gnomad	T>A	T	A
NC_000024.9:g.19150663T>A	19150663	19150663+gnomad	gnomad	T>A	T	A
NC_000024.9:g.19156119G>A	19156119	19156119+gnomad	gnomad	G>A	G	A
NC_000024.9:g.19170039T>C	19170039	19170039+gnomad	gnomad	T>C	T	C
NC_000024.9:g.19207786T>C	19207786	19207786+gnomad	gnomad	T>C	T	C
NC_000024.9:g.19269359G>A	19269359	19269359+gnomad	gnomad	G>A	G	A
NC_000024.9:g.19279512T>C	19279512	19279512+gnomad	gnomad	T>C	T	C
NC_000024.9:g.19289913C>T	19289913	19289913+gnomad	gnomad	C>T	C	T
NC_000024.9:g.19290781C>G	19290781	19290781+gnomad	gnomad	C>G	C	G
NC_000024.9:g.19313194T>A	19313194	19313194+gnomad	gnomad	T>A	T	A
NC_000024.9:g.19354030A>T	19354030	19354030+gnomad	gnomad	A>T	A	T
NC_000024.9:g.19359306A>G	19359306	19359306+gnomad	gnomad	A>G	A	G
NC_000024.9:g.19364458G>A	19364458	19364458+gnomad	gnomad	G>A	G	A
NC_000024.9:g.19382504G>A	19382504	19382504+gnomad	gnomad	G>A	G	A
NC_000024.9:g.19389593G>A	19389593	19389593+gnomad	gnomad	G>A	G	A
NC_000024.9:g.19414602A>G	19414602	19414602+gnomad	gnomad	A>G	A	G
NC_000024.9:g.19422212T>C	19422212	19422212+gnomad	gnomad	T>C	T	C
NC_000024.9:g.19433021G>A	19433021	19433021+gnomad	gnomad	G>A	G	A
NC_000024.9:g.19433286T>C	19433286	19433286+gnomad	gnomad	T>C	T	C
NC_000024.9:g.19438720C>T	19438720	19438720+gnomad	gnomad	C>T	C	T
NC_000024.9:g.19449074G>A	19449074	19449074+gnomad	gnomad	G>A	G	A
NC_000024.9:g.19462356G>A	19462356	19462356+gnomad	gnomad	G>A	G	A
NC_000024.9:g.19466283C>T	19466283	19466283+gnomad	gnomad	C>T	C	T
NC_000024.9:g.19467283A>C	19467283	19467283+gnomad	gnomad	A>C	A	C
NC_000024.9:g.19467337A>G	19467337	19467337+gnomad	gnomad	A>G	A	G
NC_000024.9:g.19491393C>T	19491393	19491393+gnomad	gnomad	C>T	C	T
NC_000024.9:g.19523582A>G	19523582	19523582+gnomad	gnomad	A>G	A	G
NC_000024.9:g.19555147G>A	19555147	19555147+gnomad	gnomad	G>A	G	A
NC_000024.9:g.21042268G>A	21042268	21042268+gnomad	gnomad	G>A	G	A
NC_000024.9:g.21101762G>A	21101762	21101762+gnomad	gnomad	G>A	G	A
NC_000024.9:g.21102034T>C	21102034	21102034+gnomad	gnomad	T>C	T	C
NC_000024.9:g.21127294T>A	21127294	21127294+gnomad	gnomad	T>A	T	A
NC_000024.9:g.21203934G>T	21203934	21203934+gnomad	gnomad	G>T	G	T
NC_000024.9:g.21266499G>A	21266499	21266499+gnomad	gnomad	G>A	G	A
NC_000024.9:g.21268937C>T	21268937	21268937+gnomad	gnomad	C>T	C	T
NC_000024.9:g.21277067G>A	21277067	21277067+gnomad	gnomad	G>A	G	A
NC_000024.9:g.21297027T>G	21297027	21297027+gnomad	gnomad	T>G	T	G
NC_000024.9:g.21305902A>G	21305902	21305902+gnomad	gnomad	A>G	A	G
NC_000024.9:g.21316364C>T	21316364	21316364+gnomad	gnomad	C>T	C	T
NC_000024.9:g.21363746C>T	21363746	21363746+gnomad	gnomad	C>T	C	T
NC_000024.9:g.21368217G>A	21368217	21368217+gnomad	gnomad	G>A	G	A
NC_000024.9:g.21384253C>T	21384253	21384253+gnomad	gnomad	C>T	C	T
NC_000024.9:g.21390792A>C	21390792	21390792+gnomad	gnomad	A>C	A	C
NC_000024.9:g.21439036A>G	21439036	21439036+gnomad	gnomad	A>G	A	G
NC_000024.9:g.21441434C>T	21441434	21441434+gnomad	gnomad	C>T	C	T
NC_000024.9:g.21444631C>T	21444631	21444631+gnomad	gnomad	C>T	C	T
NC_000024.9:g.21445856G>A	21445856	21445856+gnomad	gnomad	G>A	G	A
NC_000024.9:g.21482093C>G	21482093	21482093+gnomad	gnomad	C>G	C	G
NC_000024.9:g.21487601G>A	21487601	21487601+gnomad	gnomad	G>A	G	A
NC_000024.9:g.21489951A>G	21489951	21489951+gnomad	gnomad	A>G	A	G

NC_000024.9:g.21492315C>A	21492315	21492315+gnomad	gnomad	C>A	C	A
NC_000024.9:g.21532654A>G	21532654	21532654+gnomad	gnomad	A>G	A	G
NC_000024.9:g.21544314T>A	21544314	21544314+gnomad	gnomad	T>A	T	A
NC_000024.9:g.21568921G>A	21568921	21568921+gnomad	gnomad	G>A	G	A
NC_000024.9:g.21584488G>A	21584488	21584488+gnomad	gnomad	G>A	G	A
NC_000024.9:g.21590250C>A	21590250	21590250+gnomad	gnomad	C>A	C	A
NC_000024.9:g.21599084C>G	21599084	21599084+gnomad	gnomad	C>G	C	G
NC_000024.9:g.21610289C>T	21610289	21610289+gnomad	gnomad	C>T	C	T
NC_000024.9:g.21614588C>T	21614588	21614588+gnomad	gnomad	C>T	C	T
NC_000024.9:g.21624532G>A	21624532	21624532+gnomad	gnomad	G>A	G	A
NC_000024.9:g.21643465C>T	21643465	21643465+gnomad	gnomad	C>T	C	T
NC_000024.9:g.21663352T>C	21663352	21663352+gnomad	gnomad	T>C	T	C
NC_000024.9:g.21678097A>G	21678097	21678097+gnomad	gnomad	A>G	A	G
NC_000024.9:g.21735046A>G	21735046	21735046+gnomad	gnomad	A>G	A	G
NC_000024.9:g.21747057T>C	21747057	21747057+gnomad	gnomad	T>C	T	C
NC_000024.9:g.21769234T>C	21769234	21769234+gnomad	gnomad	T>C	T	C
NC_000024.9:g.21807104G>A	21807104	21807104+gnomad	gnomad	G>A	G	A
NC_000024.9:g.21825608C>T	21825608	21825608+gnomad	gnomad	C>T	C	T
NC_000024.9:g.21845487A>G	21845487	21845487+gnomad	gnomad	A>G	A	G
NC_000024.9:g.21906621A>G	21906621	21906621+gnomad	gnomad	A>G	A	G
NC_000024.9:g.21935629T>A	21935629	21935629+gnomad	gnomad	T>A	T	A
NC_000024.9:g.21972561G>T	21972561	21972561+gnomad	gnomad	G>T	G	T
NC_000024.9:g.22614591C>A	22614591	22614591+gnomad	gnomad	C>A	C	A
NC_000024.9:g.22631408G>A	22631408	22631408+gnomad	gnomad	G>A	G	A
NC_000024.9:g.22639039C>T	22639039	22639039+gnomad	gnomad	C>T	C	T
NC_000024.9:g.22725139C>T	22725139	22725139+gnomad	gnomad	C>T	C	T
NC_000024.9:g.22783759C>A	22783759	22783759+gnomad	gnomad	C>A	C	A
NC_000024.9:g.22802630G>A	22802630	22802630+gnomad	gnomad	G>A	G	A
NC_000024.9:g.22807170G>T	22807170	22807170+gnomad	gnomad	G>T	G	T
NC_000024.9:g.22812618G>A	22812618	22812618+gnomad	gnomad	G>A	G	A
NC_000024.9:g.22813293A>G	22813293	22813293+gnomad	gnomad	A>G	A	G
NC_000024.9:g.22820351G>A	22820351	22820351+gnomad	gnomad	G>A	G	A
NC_000024.9:g.2686207T>C	2686207	2686207+gnomad	gnomad	T>C	T	C
NC_000024.9:g.2711453T>C	2711453	2711453+gnomad	gnomad	T>C	T	C
NC_000024.9:g.2722244G>A	2722244	2722244+gnomad	gnomad	G>A	G	A
NC_000024.9:g.2766663T>A	2766663	2766663+gnomad	gnomad	T>A	T	A
NC_000024.9:g.2794516C>G	2794516	2794516+gnomad	gnomad	C>G	C	G
NC_000024.9:g.2823665A>G	2823665	2823665+gnomad	gnomad	A>G	A	G
NC_000024.9:g.2858363T>C	2858363	2858363+gnomad	gnomad	T>C	T	C
NC_000024.9:g.2867481A>T	2867481	2867481+gnomad	gnomad	A>T	A	T
NC_000024.9:g.6734211C>G	6734211	6734211+gnomad	gnomad	C>G	C	G
NC_000024.9:g.6769235A>T	6769235	6769235+gnomad	gnomad	A>T	A	T
NC_000024.9:g.6773931G>A	6773931	6773931+gnomad	gnomad	G>A	G	A
NC_000024.9:g.6776946C>T	6776946	6776946+gnomad	gnomad	C>T	C	T
NC_000024.9:g.6832380A>T	6832380	6832380+gnomad	gnomad	A>T	A	T
NC_000024.9:g.6833047A>G	6833047	6833047+gnomad	gnomad	A>G	A	G
NC_000024.9:g.6833276T>A	6833276	6833276+gnomad	gnomad	T>A	T	A
NC_000024.9:g.6852329C>T	6852329	6852329+gnomad	gnomad	C>T	C	T
NC_000024.9:g.6917905A>T	6917905	6917905+gnomad	gnomad	A>T	A	T
NC_000024.9:g.6927863G>A	6927863	6927863+gnomad	gnomad	G>A	G	A
NC_000024.9:g.6932307C>G	6932307	6932307+gnomad	gnomad	C>G	C	G
NC_000024.9:g.6950439A>G	6950439	6950439+gnomad	gnomad	A>G	A	G
NC_000024.9:g.6975145G>A	6975145	6975145+gnomad	gnomad	G>A	G	A
NC_000024.9:g.7021078C>T	7021078	7021078+gnomad	gnomad	C>T	C	T
NC_000024.9:g.7047905G>T	7047905	7047905+gnomad	gnomad	G>T	G	T
NC_000024.9:g.7069159G>A	7069159	7069159+gnomad	gnomad	G>A	G	A
NC_000024.9:g.7070982C>G	7070982	7070982+gnomad	gnomad	C>G	C	G
NC_000024.9:g.7094117G>T	7094117	7094117+gnomad	gnomad	G>T	G	T
NC_000024.9:g.7147629C>T	7147629	7147629+gnomad	gnomad	C>T	C	T
NC_000024.9:g.7165792C>G	7165792	7165792+gnomad	gnomad	C>G	C	G
NC_000024.9:g.7170410C>T	7170410	7170410+gnomad	gnomad	C>T	C	T
NC_000024.9:g.7174314G>T	7174314	7174314+gnomad	gnomad	G>T	G	T
NC_000024.9:g.7214725G>A	7214725	7214725+gnomad	gnomad	G>A	G	A
NC_000024.9:g.7228865G>A	7228865	7228865+gnomad	gnomad	G>A	G	A
NC_000024.9:g.7241780G>A	7241780	7241780+gnomad	gnomad	G>A	G	A
NC_000024.9:g.7252589G>A	7252589	7252589+gnomad	gnomad	G>A	G	A
NC_000024.9:g.7356973C>T	7356973	7356973+gnomad	gnomad	C>T	C	T
NC_000024.9:g.7419454T>A	7419454	7419454+gnomad	gnomad	T>A	T	A
NC_000024.9:g.8028789C>T	8028789	8028789+gnomad	gnomad	C>T	C	T
NC_000024.9:g.8032482C>T	8032482	8032482+gnomad	gnomad	C>T	C	T
NC_000024.9:g.14084421G>A	14084421	14084421+6QHWE_private	6QHWE_private	G>A	G	A
NC_000024.9:g.14194651G>A	14194651	14194651+6QHWE_private	6QHWE_private	G>A	G	A
NC_000024.9:g.14206138C>A	14206138	14206138+6QHWE_private	6QHWE_private	C>A	C	A
NC_000024.9:g.14341232C>A	14341232	14341232+6QHWE_private	6QHWE_private	C>A	C	A
NC_000024.9:g.14398682C>G	14398682	14398682+6QHWE_private	6QHWE_private	C>G	C	G
NC_000024.9:g.14498073G>T	14498073	14498073+6QHWE_private	6QHWE_private	G>T	G	T
NC_000024.9:g.14878757C>T	14878757	14878757+6QHWE_private	6QHWE_private	C>T	C	T
NC_000024.9:g.15073754G>A	15073754	15073754+6QHWE_private	6QHWE_private	G>A	G	A
NC_000024.9:g.15204044C>T	15204044	15204044+6QHWE_private	6QHWE_private	C>T	C	T
NC_000024.9:g.15867240C>T	15867240	15867240+6QHWE_private	6QHWE_private	C>T	C	T
NC_000024.9:g.15980062C>T	15980062	15980062+6QHWE_private	6QHWE_private	C>T	C	T
NC_000024.9:g.16529488C>A	16529488	16529488+6QHWE_private	6QHWE_private	C>A	C	A
NC_000024.9:g.16532424T>A	16532424	16532424+6QHWE_private	6QHWE_private	T>A	T	A
NC_000024.9:g.16755998T>A	16755998	16755998+6QHWE_private	6QHWE_private	T>A	T	A
NC_000024.9:g.16824962A>G	16824962	16824962+6QHWE_private	6QHWE_private	A>G	A	G
NC_000024.9:g.16921575C>A	16921575	16921575+6QHWE_private	6QHWE_private	C>A	C	A
NC_000024.9:g.17032882A>G	17032882	17032882+6QHWE_private	6QHWE_private	A>G	A	G
NC_000024.9:g.17140952A>C	17140952	17140952+6QHWE_private	6QHWE_private	A>C	A	C
NC_000024.9:g.17507997C>T	17507997	17507997+6QHWE_private	6QHWE_private	C>T	C	T
NC_000024.9:g.17528373C>G	17528373	17528373+6QHWE_private	6QHWE_private	C>G	C	G
NC_000024.9:g.17546863G>A	17546863	17546863+6QHWE_private	6QHWE_private	G>A	G	A
NC_000024.9:g.17648849T>C	17648849	17648849+6QHWE_private	6QHWE_private	T>C	T	C
NC_000024.9:g.17685415C>T	17685415	17685415+6QHWE_private	6QHWE_private	C>T	C	T
NC_000024.9:g.17781297A>G	17781297	17781297+6QHWE_private	6QHWE_private	A>G	A	G
NC_000024.9:g.18111388C>T	18111388	18111388+6QHWE_private	6QHWE_private	C>T	C	T
NC_000024.9:g.18112160A>C	18112160	18112160+6QHWE_private	6QHWE_private	A>C	A	C
NC_000024.9:g.18761277C>T	18761277	18761277+6QHWE_private	6QHWE_private	C>T	C	T
NC_000024.9:g.18803573T>C	18803573	18803573+6QHWE_private	6QHWE_private	T>C	T	C
NC_000024.9:g.19105109G>C	19105109	19105109+6QHWE_private	6QHWE_private	G>C	G	C
NC_000024.9:g.19119680C>A	19119680	19119680+6QHWE_private	6QHWE_private	C>A	C	A
NC_000024.9:g.19188650G>A	19188650	19188650+6QHWE_private	6QHWE_private	G>A	G	A
NC_000024.9:g.19264133T>A	19264133	19264133+6QHWE_private	6QHWE_private	T>A	T	A
NC_000024.9:g.21176720G>A	21176720	21176720+6QHWE_private	6QHWE_private	G>A	G	A
NC_000024.9:g.21428685C>T	21428685	21428685+6QHWE_private	6QHWE_private	C>T	C	T

NC_000024.9:g.21452743T>C	21452743	21452743+6QHWE_private	6QHWE_private	T>C	T	C
NC_000024.9:g.21623946C>T	21623946	21623946+6QHWE_private	6QHWE_private	C>T	C	T
NC_000024.9:g.21882303T>C	21882303	21882303+6QHWE_private	6QHWE_private	T>C	T	C
NC_000024.9:g.21993571C>T	21993571	21993571+6QHWE_private	6QHWE_private	C>T	C	T
NC_000024.9:g.22205606A>C	22205606	22205606+6QHWE_private	6QHWE_private	A>C	A	C
NC_000024.9:g.22753675A>T	22753675	22753675+6QHWE_private	6QHWE_private	A>T	A	T
NC_000024.9:g.22889063G>A	22889063	22889063+6QHWE_private	6QHWE_private	G>A	G	A
NC_000024.9:g.22889067G>A	22889067	22889067+6QHWE_private	6QHWE_private	G>A	G	A
NC_000024.9:g.22926293G>A	22926293	22926293+6QHWE_private	6QHWE_private	G>A	G	A
NC_000024.9:g.23013140G>C	23013140	23013140+6QHWE_private	6QHWE_private	G>C	G	C
NC_000024.9:g.23145708C>T	23145708	23145708+6QHWE_private	6QHWE_private	C>T	C	T
NC_000024.9:g.2854108C>T	2854108	2854108+6QHWE_private	6QHWE_private	C>T	C	T
NC_000024.9:g.6884667C>T	6884667	6884667+6QHWE_private	6QHWE_private	C>T	C	T
NC_000024.9:g.6912299G>A	6912299	6912299+6QHWE_private	6QHWE_private	G>A	G	A
NC_000024.9:g.18850966A>G	18850966	18850966+B35_eq	B35_eq	A>G	A	G
NC_000024.9:g.18907452C>G	18907452	18907452+B35_eq	B35_eq	C>G	C	G
NC_000024.9:g.6793301T>C	6793301	6793301+B35_eq	B35_eq	T>C	T	C
NC_000024.9:g.19219857C>T	19219857	19219857+L275_eq	L275_eq	C>T	C	T
NC_000024.9:g.28496780G>A	28496780	28496780+L275_eq	L275_eq	G>A	G	A
NC_000024.9:g.21481526G>C	21481526	21481526+LD4PC_private	LD4PC_private	G>C	G	C
NC_000024.9:g.14018503T>A	14018503	14018503+M39DJ_private	M39DJ_private	T>A	T	A
NC_000024.9:g.14286380C>G	14286380	14286380+M39DJ_private	M39DJ_private	C>G	C	G
NC_000024.9:g.14543192G>A	14543192	14543192+M39DJ_private	M39DJ_private	G>A	G	A
NC_000024.9:g.14764319T>C	14764319	14764319+M39DJ_private	M39DJ_private	T>C	T	C
NC_000024.9:g.15036632C>T	15036632	15036632+M39DJ_private	M39DJ_private	C>T	C	T
NC_000024.9:g.15042856C>T	15042856	15042856+M39DJ_private	M39DJ_private	C>T	C	T
NC_000024.9:g.15058005C>G	15058005	15058005+M39DJ_private	M39DJ_private	C>G	C	G
NC_000024.9:g.15058491A>G	15058491	15058491+M39DJ_private	M39DJ_private	A>G	A	G
NC_000024.9:g.15164108G>A	15164108	15164108+M39DJ_private	M39DJ_private	G>A	G	A
NC_000024.9:g.15510815T>C	15510815	15510815+M39DJ_private	M39DJ_private	T>C	T	C
NC_000024.9:g.16188494T>C	16188494	16188494+M39DJ_private	M39DJ_private	T>C	T	C
NC_000024.9:g.16222818G>T	16222818	16222818+M39DJ_private	M39DJ_private	G>T	G	T
NC_000024.9:g.16229766C>G	16229766	16229766+M39DJ_private	M39DJ_private	C>G	C	G
NC_000024.9:g.16548093G>A	16548093	16548093+M39DJ_private	M39DJ_private	G>A	G	A
NC_000024.9:g.16604432T>C	16604432	16604432+M39DJ_private	M39DJ_private	T>C	T	C
NC_000024.9:g.16851206G>C	16851206	16851206+M39DJ_private	M39DJ_private	G>C	G	C
NC_000024.9:g.16959760G>A	16959760	16959760+M39DJ_private	M39DJ_private	G>A	G	A
NC_000024.9:g.17134474A>G	17134474	17134474+M39DJ_private	M39DJ_private	A>G	A	G
NC_000024.9:g.17507140C>T	17507140	17507140+M39DJ_private	M39DJ_private	C>T	C	T
NC_000024.9:g.17557250A>G	17557250	17557250+M39DJ_private	M39DJ_private	A>G	A	G
NC_000024.9:g.17591130T>A	17591130	17591130+M39DJ_private	M39DJ_private	T>A	T	A
NC_000024.9:g.17717806A>C	17717806	17717806+M39DJ_private	M39DJ_private	A>C	A	C
NC_000024.9:g.17742771C>T	17742771	17742771+M39DJ_private	M39DJ_private	C>T	C	T
NC_000024.9:g.17864545T>C	17864545	17864545+M39DJ_private	M39DJ_private	T>C	T	C
NC_000024.9:g.17892245C>T	17892245	17892245+M39DJ_private	M39DJ_private	C>T	C	T
NC_000024.9:g.18103414A>G	18103414	18103414+M39DJ_private	M39DJ_private	A>G	A	G
NC_000024.9:g.18117462A>C	18117462	18117462+M39DJ_private	M39DJ_private	A>C	A	C
NC_000024.9:g.18151009C>T	18151009	18151009+M39DJ_private	M39DJ_private	C>T	C	T
NC_000024.9:g.18159925G>T	18159925	18159925+M39DJ_private	M39DJ_private	G>T	G	T
NC_000024.9:g.18221618A>G	18221618	18221618+M39DJ_private	M39DJ_private	A>G	A	G
NC_000024.9:g.18620869C>T	18620869	18620869+M39DJ_private	M39DJ_private	C>T	C	T
NC_000024.9:g.18775024C>G	18775024	18775024+M39DJ_private	M39DJ_private	C>G	C	G
NC_000024.9:g.18780840C>T	18780840	18780840+M39DJ_private	M39DJ_private	C>T	C	T
NC_000024.9:g.19088593G>A	19088593	19088593+M39DJ_private	M39DJ_private	G>A	G	A
NC_000024.9:g.19349198G>A	19349198	19349198+M39DJ_private	M39DJ_private	G>A	G	A
NC_000024.9:g.19406301A>T	19406301	19406301+M39DJ_private	M39DJ_private	A>T	A	T
NC_000024.9:g.21413643T>C	21413643	21413643+M39DJ_private	M39DJ_private	T>C	T	C
NC_000024.9:g.21541899C>A	21541899	21541899+M39DJ_private	M39DJ_private	C>A	C	A
NC_000024.9:g.21653531G>T	21653531	21653531+M39DJ_private	M39DJ_private	G>T	G	T
NC_000024.9:g.21682273C>T	21682273	21682273+M39DJ_private	M39DJ_private	C>T	C	T
NC_000024.9:g.21738880C>A	21738880	21738880+M39DJ_private	M39DJ_private	C>A	C	A
NC_000024.9:g.21798822A>G	21798822	21798822+M39DJ_private	M39DJ_private	A>G	A	G
NC_000024.9:g.21864672C>T	21864672	21864672+M39DJ_private	M39DJ_private	C>T	C	T
NC_000024.9:g.22177050A>T	22177050	22177050+M39DJ_private	M39DJ_private	A>T	A	T
NC_000024.9:g.22566260T>C	22566260	22566260+M39DJ_private	M39DJ_private	T>C	T	C
NC_000024.9:g.22625314C>G	22625314	22625314+M39DJ_private	M39DJ_private	C>G	C	G
NC_000024.9:g.22723179C>G	22723179	22723179+M39DJ_private	M39DJ_private	C>G	C	G
NC_000024.9:g.22753117T>C	22753117	22753117+M39DJ_private	M39DJ_private	T>C	T	C
NC_000024.9:g.22762736A>G	22762736	22762736+M39DJ_private	M39DJ_private	A>G	A	G
NC_000024.9:g.22977722C>A	22977722	22977722+M39DJ_private	M39DJ_private	C>A	C	A
NC_000024.9:g.23298883A>C	23298883	23298883+M39DJ_private	M39DJ_private	A>C	A	C
NC_000024.9:g.23477319C>T	23477319	23477319+M39DJ_private	M39DJ_private	C>T	C	T
NC_000024.9:g.6740037A>C	6740037	6740037+M39DJ_private	M39DJ_private	A>C	A	C
NC_000024.9:g.21661290C>G	21661290	21661290+MPB016_eq	MPB016_eq	C>G	C	G
NC_000024.9:g.6793738T>A	6793738	6793738+MPB016_eq	MPB016_eq	T>A	T	A
NC_000024.9:g.7136193C>A	7136193	7136193+MPB016_eq	MPB016_eq	C>A	C	A
NC_000024.9:g.18108468T>A	18108468	18108468+MPB118_eq	MPB118_eq	T>A	T	A
NC_000024.9:g.21239957C>G	21239957	21239957+MPB118_eq	MPB118_eq	C>G	C	G
NC_000024.9:g.16517006A>T	16517006	16517006+N8A2QN_private	N8A2QN_private	A>T	A	T
NC_000024.9:g.17906514A>G	17906514	17906514+N8A2QN_private	N8A2QN_private	A>G	A	G
NC_000024.9:g.17263815G>C	17263815	17263815+private_N87FK8	private_N87FK8	G>C	G	C
NC_000024.9:g.14141137T>C	14141137	14141137+private_TYEQC	private_TYEQC	T>C	T	C
NC_000024.9:g.15789285A>G	15789285	15789285+private_TYEQC	private_TYEQC	A>G	A	G
NC_000024.9:g.15879887A>G	15879887	15879887+private_TYEQC	private_TYEQC	A>G	A	G
NC_000024.9:g.15996147A>T	15996147	15996147+private_TYEQC	private_TYEQC	A>T	A	T
NC_000024.9:g.16570463A>G	16570463	16570463+private_TYEQC	private_TYEQC	A>G	A	G
NC_000024.9:g.16661845G>A	16661845	16661845+private_TYEQC	private_TYEQC	G>A	G	A
NC_000024.9:g.16798270T>A	16798270	16798270+private_TYEQC	private_TYEQC	T>A	T	A
NC_000024.9:g.17362149C>T	17362149	17362149+private_TYEQC	private_TYEQC	C>T	C	T
NC_000024.9:g.17405847C>T	17405847	17405847+private_TYEQC	private_TYEQC	C>T	C	T
NC_000024.9:g.17800135A>T	17800135	17800135+private_TYEQC	private_TYEQC	A>T	A	T
NC_000024.9:g.18690769G>A	18690769	18690769+private_TYEQC	private_TYEQC	G>A	G	A
NC_000024.9:g.18787905G>C	18787905	18787905+private_TYEQC	private_TYEQC	G>C	G	C
NC_000024.9:g.19126591C>T	19126591	19126591+private_TYEQC	private_TYEQC	C>T	C	T
NC_000024.9:g.19257518A>T	19257518	19257518+private_TYEQC	private_TYEQC	A>T	A	T
NC_000024.9:g.21226007C>T	21226007	21226007+private_TYEQC	private_TYEQC	C>T	C	T
NC_000024.9:g.21304756A>G	21304756	21304756+private_TYEQC	private_TYEQC	A>G	A	G
NC_000024.9:g.21404372G>A	21404372	21404372+private_TYEQC	private_TYEQC	G>A	G	A
NC_000024.9:g.21561881G>A	21561881	21561881+private_TYEQC	private_TYEQC	G>A	G	A
NC_000024.9:g.21589945A>T	21589945	21589945+private_TYEQC	private_TYEQC	A>T	A	T
NC_000024.9:g.21715705A>T	21715705	21715705+private_TYEQC	private_TYEQC	A>T	A	T
NC_000024.9:g.22138450G>A	22138450	22138450+private_TYEQC	private_TYEQC	G>A	G	A
NC_000024.9:g.22140339C>T	22140339	22140339+private_TYEQC	private_TYEQC	C>T	C	T
NC_000024.9:g.22601538A>T	22601538	22601538+private_TYEQC	private_TYEQC	A>T	A	T

NC_000024.9:g.23291826A>G	23291826	23291826+private_TYEQC	private_TYEQC	A>G	A	G
NC_000024.9:g.23296971A>G	23296971	23296971+private_TYEQC	private_TYEQC	A>G	A	G
NC_000024.9:g.23354813A>C	23354813	23354813+private_TYEQC	private_TYEQC	A>C	A	C
NC_000024.9:g.23491604C>G	23491604	23491604+private_TYEQC	private_TYEQC	C>G	C	G
NC_000024.9:g.28632123A>G	28632123	28632123+private_TYEQC	private_TYEQC	A>G	A	G
NC_000024.9:g.7112836A>G	7112836	7112836+private_TYEQC	private_TYEQC	A>G	A	G
NC_000024.9:g.14804262G>T	14804262	14804262+T4WQV_private	T4WQV_private	G>T	G	T
NC_000024.9:g.15727399C>T	15727399	15727399+T4WQV_private	T4WQV_private	C>T	C	T
NC_000024.9:g.16594190A>C	16594190	16594190+T4WQV_private	T4WQV_private	A>C	A	C
NC_000024.9:g.16907588G>A	16907588	16907588+T4WQV_private	T4WQV_private	G>A	G	A
NC_000024.9:g.17203842T>G	17203842	17203842+T4WQV_private	T4WQV_private	T>G	T	G
NC_000024.9:g.17421079G>A	17421079	17421079+T4WQV_private	T4WQV_private	G>A	G	A
NC_000024.9:g.17806174A>T	17806174	17806174+T4WQV_private	T4WQV_private	A>T	A	T
NC_000024.9:g.18196822C>T	18196822	18196822+T4WQV_private	T4WQV_private	C>T	C	T
NC_000024.9:g.18664342C>A	18664342	18664342+T4WQV_private	T4WQV_private	C>A	C	A
NC_000024.9:g.19217204T>A	19217204	19217204+T4WQV_private	T4WQV_private	T>A	T	A
NC_000024.9:g.19349622A>G	19349622	19349622+T4WQV_private	T4WQV_private	A>G	A	G
NC_000024.9:g.21062438T>C	21062438	21062438+T4WQV_private	T4WQV_private	T>C	T	C
NC_000024.9:g.22137132C>A	22137132	22137132+T4WQV_private	T4WQV_private	C>A	C	A
NC_000024.9:g.14019278G>T	14019278	14019278+UCNEN_private	UCNEN_private	G>T	G	T
NC_000024.9:g.14186161T>A	14186161	14186161+UCNEN_private	UCNEN_private	T>A	T	A
NC_000024.9:g.15027317A>C	15027317	15027317+UCNEN_private	UCNEN_private	A>C	A	C
NC_000024.9:g.15318793C>A	15318793	15318793+UCNEN_private	UCNEN_private	C>A	C	A
NC_000024.9:g.15355766T>C	15355766	15355766+UCNEN_private	UCNEN_private	T>C	T	C
NC_000024.9:g.15356961T>C	15356961	15356961+UCNEN_private	UCNEN_private	T>C	T	C
NC_000024.9:g.15447970A>G	15447970	15447970+UCNEN_private	UCNEN_private	A>G	A	G
NC_000024.9:g.15569473C>T	15569473	15569473+UCNEN_private	UCNEN_private	C>T	C	T
NC_000024.9:g.15576335T>C	15576335	15576335+UCNEN_private	UCNEN_private	T>C	T	C
NC_000024.9:g.15685181A>G	15685181	15685181+UCNEN_private	UCNEN_private	A>G	A	G
NC_000024.9:g.15846296G>C	15846296	15846296+UCNEN_private	UCNEN_private	G>C	G	C
NC_000024.9:g.15926369C>T	15926369	15926369+UCNEN_private	UCNEN_private	C>T	C	T
NC_000024.9:g.15926490C>G	15926490	15926490+UCNEN_private	UCNEN_private	C>G	C	G
NC_000024.9:g.15951681T>A	15951681	15951681+UCNEN_private	UCNEN_private	T>A	T	A
NC_000024.9:g.15956791G>T	15956791	15956791+UCNEN_private	UCNEN_private	G>T	G	T
NC_000024.9:g.16303474A>G	16303474	16303474+UCNEN_private	UCNEN_private	A>G	A	G
NC_000024.9:g.16524207A>G	16524207	16524207+UCNEN_private	UCNEN_private	A>G	A	G
NC_000024.9:g.16596119G>A	16596119	16596119+UCNEN_private	UCNEN_private	G>A	G	A
NC_000024.9:g.17076462A>G	17076462	17076462+UCNEN_private	UCNEN_private	A>G	A	G
NC_000024.9:g.17174292C>T	17174292	17174292+UCNEN_private	UCNEN_private	C>T	C	T
NC_000024.9:g.17347823T>C	17347823	17347823+UCNEN_private	UCNEN_private	T>C	T	C
NC_000024.9:g.17411085G>A	17411085	17411085+UCNEN_private	UCNEN_private	G>A	G	A
NC_000024.9:g.17956798A>G	17956798	17956798+UCNEN_private	UCNEN_private	A>G	A	G
NC_000024.9:g.18067616G>A	18067616	18067616+UCNEN_private	UCNEN_private	G>A	G	A
NC_000024.9:g.18083626T>C	18083626	18083626+UCNEN_private	UCNEN_private	T>C	T	C
NC_000024.9:g.18122506G>C	18122506	18122506+UCNEN_private	UCNEN_private	G>C	G	C
NC_000024.9:g.18241542G>A	18241542	18241542+UCNEN_private	UCNEN_private	G>A	G	A
NC_000024.9:g.18633047G>A	18633047	18633047+UCNEN_private	UCNEN_private	G>A	G	A
NC_000024.9:g.18832804A>C	18832804	18832804+UCNEN_private	UCNEN_private	A>C	A	C
NC_000024.9:g.19033422A>G	19033422	19033422+UCNEN_private	UCNEN_private	A>G	A	G
NC_000024.9:g.19209618C>A	19209618	19209618+UCNEN_private	UCNEN_private	C>A	C	A
NC_000024.9:g.21070636C>T	21070636	21070636+UCNEN_private	UCNEN_private	C>T	C	T
NC_000024.9:g.21239939C>G	21239939	21239939+UCNEN_private	UCNEN_private	C>G	C	G
NC_000024.9:g.21843423T>A	21843423	21843423+UCNEN_private	UCNEN_private	T>A	T	A
NC_000024.9:g.22117043C>T	22117043	22117043+UCNEN_private	UCNEN_private	C>T	C	T
NC_000024.9:g.22130922T>C	22130922	22130922+UCNEN_private	UCNEN_private	T>C	T	C
NC_000024.9:g.22190115A>G	22190115	22190115+UCNEN_private	UCNEN_private	A>G	A	G
NC_000024.9:g.22591954C>T	22591954	22591954+UCNEN_private	UCNEN_private	C>T	C	T
NC_000024.9:g.22892915G>A	22892915	22892915+UCNEN_private	UCNEN_private	G>A	G	A
NC_000024.9:g.22991328A>G	22991328	22991328+UCNEN_private	UCNEN_private	A>G	A	G
NC_000024.9:g.23259013G>T	23259013	23259013+UCNEN_private	UCNEN_private	G>T	G	T
NC_000024.9:g.6743056T>C	6743056	6743056+UCNEN_private	UCNEN_private	T>C	T	C
NC_000024.9:g.6793820G>A	6793820	6793820+UCNEN_private	UCNEN_private	G>A	G	A
NC_000024.9:g.7252911T>G	7252911	7252911+UCNEN_private	UCNEN_private	T>G	T	G
NC_000024.9:g.16212604G>T	16212604	16212604+Z5910_eq	Z5910_eq	G>T	G	T
NC_000024.9:g.22162360C>T	22162360	22162360+Z5917_eq	Z5917_eq	C>T	C	T
NC_000024.9:g.22657015C>T	22657015	22657015+Z780_eq	Z780_eq	C>T	C	T
NC_000024.9:g.28731705C>A	28731705	28731705+Z780_eq	Z780_eq	C>A	C	A
NC_000024.9:g.6753291G>T	6753291	6753291+L1242,I1a3a1a2b~	L1242,I1a3a1a2b~	G>T	G	T
NC_000024.9:g.14491835C>T	14491835	14491835+L1425,Q1b1a1e2	L1425,Q1b1a1e2	C>T	C	T
NC_000024.9:g.6753258T>C	6753258	6753258+L147.1^^,PF4883.1^^,J1a2a1a2b	L147.1^^,PF4883.1^^,J1a2a1a2b	T>C	T	C
NC_000024.9:g.14889974C>T	14889974	14889974+L215,Page82,S325,Q2a	L215,Page82,S325,Q2a	C>T	C	T
NC_000024.9:g.17516095G>A	17516095	17516095+L232,S432,Q	L232,S432,Q	G>A	G	A
NC_000024.9:g.19136888G>A	19136888	19136888+L275,Q2	L275,Q2	G>A	G	A
NC_000024.9:g.17661226G>C	17661226	17661226+L331,Q1b1	L331,Q1b1	G>C	G	C
NC_000024.9:g.18146921G>A	18146921	18146921+L475,Q1b1	L475,Q1b1	G>A	G	A
NC_000024.9:g.19304761G>A	19304761	19304761+L476,Q1b1	L476,Q1b1	G>A	G	A
NC_000024.9:g.21642296G>A	21642296	21642296+L53,S326,Q1b1	L53,S326,Q1b1	G>A	G	A
NC_000024.9:g.23292782G>A	23292782	23292782+L54,Q1b1a	L54,Q1b1a	G>A	G	A
NC_000024.9:g.19413335G>A	19413335	19413335+L55,Q1b1	L55,Q1b1	G>A	G	A
NC_000024.9:g.15574102G>A	15574102	15574102+L57,Q1b	L57,Q1b	G>A	G	A
NC_000024.9:g.6753287A>G	6753287	6753287+L684,H1a1a1b	L684,H1a1a1b	A>G	A	G
NC_000024.9:g.17913419G>A	17913419	17913419+M1022,Q1b1a2	M1022,Q1b1a2	G>A	G	A
NC_000024.9:g.18898848A>G	18898848	18898848+M1027,Q1b1a2	M1027,Q1b1a2	A>G	A	G
NC_000024.9:g.21573011G>A	21573011	21573011+M1039,Z791,Q1b1a2	M1039,Z791,Q1b1a2	G>A	G	A
NC_000024.9:g.22594593G>C	22594593	22594593+M1049,Z793,Q1b1a2~	M1049,Z793,Q1b1a2~	G>C	G	C
NC_000024.9:g.28645581T>A	28645581	28645581+M1060,E1b1a1a1a2a1b2~	M1060,E1b1a1a1a2a1b2~	T>A	T	A
NC_000024.9:g.13952379C>T	13952379	13952379+M1086,Q1b1~	M1086,Q1b1~	C>T	C	T
NC_000024.9:g.18101626C>T	18101626	18101626+M1141,Q1b1~	M1141,Q1b1~	C>T	C	T
NC_000024.9:g.21079551T>C	21079551	21079551+M1152,Y767,Q1b	M1152,Y767,Q1b	T>C	T	C
NC_000024.9:g.21079955T>C	21079955	21079955+M1153,Q1b	M1153,Q1b	T>C	T	C
NC_000024.9:g.21254696G>A	21254696	21254696+M1155,Q1	M1155,Q1	G>A	G	A
NC_000024.9:g.21330428A>G	21330428	21330428+M1156,Y744,Q1b	M1156,Y744,Q1b	A>G	A	G
NC_000024.9:g.21352775C>T	21352775	21352775+M1157,Q1b	M1157,Q1b	C>T	C	T
NC_000024.9:g.21890333C>T	21890333	21890333+M1161,Y768,Q1b1~	M1161,Y768,Q1b1~	C>T	C	T
NC_000024.9:g.21951098A>G	21951098	21951098+M1162,Q1b1~	M1162,Q1b1~	A>G	A	G
NC_000024.9:g.21963247A>G	21963247	21963247+M1164,Y760,Q1b1~	M1164,Y760,Q1b1~	A>G	A	G
NC_000024.9:g.22059040A>G	22059040	22059040+M1167,Q1b1~	M1167,Q1b1~	A>G	A	G
NC_000024.9:g.23489071C>T	23489071	23489071+M1177,Y761,Q1b1~	M1177,Y761,Q1b1~	C>T	C	T
NC_000024.9:g.6929806C>T	6929806	6929806+M12335,R1a1a1b1a2b3a4a2b~	M12335,R1a1a1b1a2b3a4a2b~	C>T	C	T
NC_000024.9:g.14339808C>G	14339808	14339808+M2470,L	M2470,L	C>G	C	G
NC_000024.9:g.21878708G>A	21878708	21878708+M289	M289	G>A	G	A
NC_000024.9:g.19096363G>A	19096363	19096363+M3,Q1b1a1a	M3,Q1b1a1a	G>A	G	A
NC_000024.9:g.2887156C>G	2887156	2887156+M346,Q1b	M346,Q1b	C>G	C	G
NC_000024.9:g.15027507A>G	15027507	15027507+M378,Page100,Q2a1	M378,Page100,Q2a1	A>G	A	G

NC_000024.9:g.17007374C>T	17007374	17007374+M7658,B2b1a2b~	M7658,B2b1a2b~	C>T	C	T
NC_000024.9:g.6740034C>A	6740034	6740034+M8963.2,I2a1b1a2b1a2a3a1~	M8963.2,I2a1b1a2b1a2a3a1~	C>A	C	A
NC_000024.9:g.21149835T>A	21149835	21149835+M902,Q1b1a1a1	M902,Q1b1a1a1	T>A	T	A
NC_000024.9:g.6631686C>T	6631686	6631686+M944,Q1b1a2	M944,Q1b1a2	C>T	C	T
NC_000024.9:g.14149428C>T	14149428	14149428+M982,Q1b1a2	M982,Q1b1a2	C>T	C	T
NC_000024.9:g.8622146T>A	8622146	8622146+MPB002	MPB002	T>A	T	A
NC_000024.9:g.15853695C>T	15853695	15853695+MPB003	MPB003	C>T	C	T
NC_000024.9:g.21786976A>G	21786976	21786976+MPB008	MPB008	A>G	A	G
NC_000024.9:g.21789685G>T	21789685	21789685+MPB009	MPB009	G>T	G	T
NC_000024.9:g.14111656G>T	14111656	14111656+MPB010	MPB010	G>T	G	T
NC_000024.9:g.28537340G>A	28537340	28537340+MPB011	MPB011	G>A	G	A
NC_000024.9:g.2887944A>C	2887944	2887944+MPB012	MPB012	A>C	A	C
NC_000024.9:g.8115275G>A	8115275	8115275+MPB013	MPB013	G>A	G	A
NC_000024.9:g.14831006A>G	14831006	14831006+MPB014	MPB014	A>G	A	G
NC_000024.9:g.23881858T>C	23881858	23881858+MPB015	MPB015	T>C	T	C
NC_000024.9:g.8025278A>G	8025278	8025278+MPB016	MPB016	A>G	A	G
NC_000024.9:g.22774282G>T	22774282	22774282+MPB024	MPB024	G>T	G	T
NC_000024.9:g.8259828C>T	8259828	8259828+MPB034	MPB034	C>T	C	T
NC_000024.9:g.17100544G>A	17100544	17100544+MPB047	MPB047	G>A	G	A
NC_000024.9:g.17466912A>T	17466912	17466912+MPB049	MPB049	A>T	A	T
NC_000024.9:g.14376670G>A	14376670	14376670+MPB069.2,Z19477.2,O2a1b1a1a1b1a1c1a1	MPB069.2,Z19477.2,O2a1b1a1a1b1a1c1a1	G>A	G	A
NC_000024.9:g.7730471G>C	7730471	7730471+MPB073	MPB073	G>C	G	C
NC_000024.9:g.2904411T>C	2904411	2904411+MPB074	MPB074	T>C	T	C
NC_000024.9:g.6793855T>C	6793855	6793855+MPB075	MPB075	T>C	T	C
NC_000024.9:g.8041202A>G	8041202	8041202+MPB076	MPB076	A>G	A	G
NC_000024.9:g.8416938G>A	8416938	8416938+MPB077	MPB077	G>A	G	A
NC_000024.9:g.8783917A>T	8783917	8783917+MPB078	MPB078	A>T	A	T
NC_000024.9:g.8853387T>C	8853387	8853387+MPB079	MPB079	T>C	T	C
NC_000024.9:g.9141584C>G	9141584	9141584+MPB080	MPB080	C>G	C	G
NC_000024.9:g.9142200C>T	9142200	9142200+MPB081	MPB081	C>T	C	T
NC_000024.9:g.14387871G>T	14387871	14387871+MPB082	MPB082	G>T	G	T
NC_000024.9:g.14536746T>G	14536746	14536746+MPB083	MPB083	T>G	T	G
NC_000024.9:g.14588713G>A	14588713	14588713+MPB084	MPB084	G>A	G	A
NC_000024.9:g.14768064G>A	14768064	14768064+MPB085	MPB085	G>A	G	A
NC_000024.9:g.16063187C>G	16063187	16063187+MPB086	MPB086	C>G	C	G
NC_000024.9:g.16312932G>A	16312932	16312932+MPB087	MPB087	G>A	G	A
NC_000024.9:g.16361049C>G	16361049	16361049+MPB088	MPB088	C>G	C	G
NC_000024.9:g.16541267G>A	16541267	16541267+MPB089	MPB089	G>A	G	A
NC_000024.9:g.16541273T>C	16541273	16541273+MPB090	MPB090	T>C	T	C
NC_000024.9:g.16691849C>T	16691849	16691849+MPB091	MPB091	C>T	C	T
NC_000024.9:g.17046880T>C	17046880	17046880+MPB092	MPB092	T>C	T	C
NC_000024.9:g.18075139C>T	18075139	18075139+MPB093	MPB093	C>T	C	T
NC_000024.9:g.18566385T>C	18566385	18566385+MPB094	MPB094	T>C	T	C
NC_000024.9:g.18703360A>G	18703360	18703360+MPB095	MPB095	A>G	A	G
NC_000024.9:g.19093267T>C	19093267	19093267+MPB096	MPB096	T>C	T	C
NC_000024.9:g.19412415G>A	19412415	19412415+MPB097	MPB097	G>A	G	A
NC_000024.9:g.19443704C>G	19443704	19443704+MPB098	MPB098	C>G	C	G
NC_000024.9:g.19535405A>G	19535405	19535405+MPB099	MPB099	A>G	A	G
NC_000024.9:g.21068023A>C	21068023	21068023+MPB100	MPB100	A>C	A	C
NC_000024.9:g.21074311T>C	21074311	21074311+MPB101	MPB101	T>C	T	C
NC_000024.9:g.21080509G>T	21080509	21080509+MPB102	MPB102	G>T	G	T
NC_000024.9:g.21212879T>C	21212879	21212879+MPB103	MPB103	T>C	T	C
NC_000024.9:g.21476510G>A	21476510	21476510+MPB104	MPB104	G>A	G	A
NC_000024.9:g.21593105A>G	21593105	21593105+MPB105	MPB105	A>G	A	G
NC_000024.9:g.21627192T>C	21627192	21627192+MPB106	MPB106	T>C	T	C
NC_000024.9:g.21758691T>G	21758691	21758691+MPB107	MPB107	T>G	T	G
NC_000024.9:g.22560182C>T	22560182	22560182+MPB108	MPB108	C>T	C	T
NC_000024.9:g.22845730T>C	22845730	22845730+MPB109	MPB109	T>C	T	C
NC_000024.9:g.23479934A>T	23479934	23479934+MPB110	MPB110	A>T	A	T
NC_000024.9:g.23558384T>C	23558384	23558384+MPB111	MPB111	T>C	T	C
NC_000024.9:g.23762766C>T	23762766	23762766+MPB112	MPB112	C>T	C	T
NC_000024.9:g.23885353G>A	23885353	23885353+MPB113	MPB113	G>A	G	A
NC_000024.9:g.24430702A>G	24430702	24430702+MPB114	MPB114	A>G	A	G
NC_000024.9:g.9399070G>C	9399070	9399070+MPB115	MPB115	G>C	G	C
NC_000024.9:g.8234285A>G	8234285	8234285+MPB120	MPB120	A>G	A	G
NC_000024.9:g.8771442C>T	8771442	8771442+MPB122	MPB122	C>T	C	T
NC_000024.9:g.9445826A>T	9445826	9445826+MPB123	MPB123	A>T	A	T
NC_000024.9:g.15820739A>G	15820739	15820739+MPB127	MPB127	A>G	A	G
NC_000024.9:g.17709056A>T	17709056	17709056+MPB128	MPB128	A>T	A	T
NC_000024.9:g.18179405A>C	18179405	18179405+MPB129	MPB129	A>C	A	C
NC_000024.9:g.18232613G>T	18232613	18232613+MPB130	MPB130	G>T	G	T
NC_000024.9:g.19034296G>A	19034296	19034296+MPB131	MPB131	G>A	G	A
NC_000024.9:g.21907743T>C	21907743	21907743+MPB136	MPB136	T>C	T	C
NC_000024.9:g.23292630C>G	23292630	23292630+MPB137	MPB137	C>G	C	G
NC_000024.9:g.6752837T>C	6752837	6752837+MPB139	MPB139	T>C	T	C
NC_000024.9:g.8287550G>A	8287550	8287550+MPB140	MPB140	G>A	G	A
NC_000024.9:g.14530849T>A	14530849	14530849+MPB143	MPB143	T>A	T	A
NC_000024.9:g.18975159G>A	18975159	18975159+MPB145	MPB145	G>A	G	A
NC_000024.9:g.19099657C>T	19099657	19099657+MPB146	MPB146	C>T	C	T
NC_000024.9:g.28683931G>A	28683931	28683931+MPB148	MPB148	G>A	G	A
NC_000024.9:g.7568683C>T	7568683	7568683+MPB149	MPB149	C>T	C	T
NC_000024.9:g.13895226C>T	13895226	13895226+MPB151	MPB151	C>T	C	T
NC_000024.9:g.15780131T>C	15780131	15780131+MPB154	MPB154	T>C	T	C
NC_000024.9:g.15918456C>T	15918456	15918456+MPB156	MPB156	C>T	C	T
NC_000024.9:g.23113735G>A	23113735	23113735+MPB159	MPB159	G>A	G	A
NC_000024.9:g.17485418G>T	17485418	17485418+MPB160	MPB160	G>T	G	T
NC_000024.9:g.13918077G>C	13918077	13918077+MPB162	MPB162	G>C	G	C
NC_000024.9:g.22662786C>T	22662786	22662786+MPB163	MPB163	C>T	C	T
NC_000024.9:g.23857014G>T	23857014	23857014+MPB164	MPB164	G>T	G	T
NC_000024.9:g.7327684C>T	7327684	7327684+MPB165	MPB165	C>T	C	T
NC_000024.9:g.8055626C>A	8055626	8055626+MPB166	MPB166	C>A	C	A
NC_000024.9:g.16687733A>C	16687733	16687733+MPB167	MPB167	A>C	A	C
NC_000024.9:g.18223025T>A	18223025	18223025+MPB168	MPB168	T>A	T	A
NC_000024.9:g.28622574T>A	28622574	28622574+MPB169	MPB169	T>A	T	A
NC_000024.9:g.14067678T>C	14067678	14067678+MPB170	MPB170	T>C	T	C
NC_000024.9:g.22590797C>A	22590797	22590797+MPB174	MPB174	C>A	C	A
NC_000024.9:g.2752579A>G	2752579	2752579+MPB426	MPB426	A>G	A	G
NC_000024.9:g.8631247A>G	8631247	8631247+MPB428	MPB428	A>G	A	G
NC_000024.9:g.14496441G>T	14496441	14496441+P36.1,A1b1a1	P36.1,A1b1a1	G>T	G	T
NC_000024.9:g.14491649T>C	14491649	14491649+P53.2,D1a2a1a2a~	P53.2,D1a2a1a2a~	T>C	T	C
NC_000024.9:g.21834571G>A	21834571	21834571+PF1076.2,Q1b1a1a1p	PF1076.2,Q1b1a1a1p	G>A	G	A
NC_000024.9:g.18943495T>A	18943495	18943495+PF2542,E1b1b1b1a1g~	PF2542,E1b1b1b1a1g~	T>A	T	A
NC_000024.9:g.7018117C>T	7018117	7018117+PF4276	PF4276	C>T	C	T
NC_000024.9:g.17905568C>T	17905568	17905568+PF4974	PF4974	C>T	C	T

NC_000024.9:g.21051456G>A	21051456	21051456+PF5657,T	PF5657,T	G>A	G	A
NC_000024.9:g.15881322G>A	15881322	15881322+PF5766,L2~	PF5766,L2~	G>A	G	A
NC_000024.9:g.8087573A>T	8087573	8087573+Q1a,F1096	Q1a,F1096	A>T	A	T
NC_000024.9:g.8454150A>C	8454150	8454150+Q1a,F1215	Q1a,F1215	A>C	A	C
NC_000024.9:g.8491335T>G	8491335	8491335+Q1a,F1251	Q1a,F1251	T>G	T	G
NC_000024.9:g.14299613C>T	14299613	14299613+Q1a,F1755	Q1a,F1755	C>T	C	T
NC_000024.9:g.14325979A>T	14325979	14325979+Q1a,F1762	Q1a,F1762	A>T	A	T
NC_000024.9:g.15634135C>T	15634135	15634135+Q1a,F1979	Q1a,F1979	C>T	C	T
NC_000024.9:g.15976684T>G	15976684	15976684+Q1a,F2054	Q1a,F2054	T>G	T	G
NC_000024.9:g.16569510G>T	16569510	16569510+Q1a,F2203	Q1a,F2203	G>T	G	T
NC_000024.9:g.18234309A>C	18234309	18234309+Q1a,F2743	Q1a,F2743	A>C	A	C
NC_000024.9:g.18401339A>T	18401339	18401339+Q1a,F2753	Q1a,F2753	A>T	A	T
NC_000024.9:g.18599094C>T	18599094	18599094+Q1a,F2786	Q1a,F2786	C>T	C	T
NC_000024.9:g.18642600C>T	18642600	18642600+Q1a,F2798	Q1a,F2798	C>T	C	T
NC_000024.9:g.21723218T>C	21723218	21723218+Q1a,F3243	Q1a,F3243	T>C	T	C
NC_000024.9:g.6871854G>T	6871854	6871854+Q1a,F844	Q1a,F844	G>T	G	T
NC_000024.9:g.14275583G>A	14275583	14275583+Q1a,Y627	Q1a,Y627	G>A	G	A
NC_000024.9:g.16201957C>T	16201957	16201957+Q1a,Y638	Q1a,Y638	C>T	C	T
NC_000024.9:g.6764055A>C	6764055	6764055+Q1a,Y663	Q1a,Y663	A>C	A	C
NC_000024.9:g.19302992C>T	19302992	19302992+Q1a,Y664	Q1a,Y664	C>T	C	T
NC_000024.9:g.18410781G>A	18410781	18410781+Q1a,Y692	Q1a,Y692	G>A	G	A
NC_000024.9:g.21738511T>C	21738511	21738511+Q1a,Y706	Q1a,Y706	T>C	T	C
NC_000024.9:g.23606505C>G	23606505	23606505+Q1a,Y9312	Q1a,Y9312	C>G	C	G
NC_000024.9:g.7932797G>A	7932797	7932797+Q1a,Y9313	Q1a,Y9313	G>A	G	A
NC_000024.9:g.17341480C>A	17341480	17341480+Q1a,Y9314	Q1a,Y9314	C>A	C	A
NC_000024.9:g.18104889C>T	18104889	18104889+Q1a,Z19188	Q1a,Z19188	C>T	C	T
NC_000024.9:g.16520179T>G	16520179	16520179+Q1a,Z19222	Q1a,Z19222	T>G	T	G
NC_000024.9:g.8438421A>T	8438421	8438421+Q1a1,F1202	Q1a1,F1202	A>T	A	T
NC_000024.9:g.8500133G>A	8500133	8500133+Q1a1,F1261	Q1a1,F1261	G>A	G	A
NC_000024.9:g.8547406A>C	8547406	8547406+Q1a1,F1298	Q1a1,F1298	A>C	A	C
NC_000024.9:g.8554290A>G	8554290	8554290+Q1a1,F1303	Q1a1,F1303	A>G	A	G
NC_000024.9:g.9163911G>T	9163911	9163911+Q1a1,F1505	Q1a1,F1505	G>T	G	T
NC_000024.9:g.14285881C>T	14285881	14285881+Q1a1,F1748	Q1a1,F1748	C>T	C	T
NC_000024.9:g.22904616G>A	22904616	22904616+Q1a1,F3366	Q1a1,F3366	G>A	G	A
NC_000024.9:g.14208130C>T	14208130	14208130+Q1a1,F4164	Q1a1,F4164	C>T	C	T
NC_000024.9:g.13216723T>A	13216723	13216723+Q1a1,F4853	Q1a1,F4853	T>A	T	A
NC_000024.9:g.13821459G>A	13821459	13821459+Q1a1,F4902	Q1a1,F4902	G>A	G	A
NC_000024.9:g.14812749G>A	14812749	14812749+Q1a1,F4972	Q1a1,F4972	G>A	G	A
NC_000024.9:g.16295379G>A	16295379	16295379+Q1a1,F5039	Q1a1,F5039	G>A	G	A
NC_000024.9:g.16490831T>C	16490831	16490831+Q1a1,F5048	Q1a1,F5048	T>C	T	C
NC_000024.9:g.16863096G>T	16863096	16863096+Q1a1,F5067	Q1a1,F5067	G>T	G	T
NC_000024.9:g.18991911C>T	18991911	18991911+Q1a1,F5175	Q1a1,F5175	C>T	C	T
NC_000024.9:g.21560759C>T	21560759	21560759+Q1a1,F5246	Q1a1,F5246	C>T	C	T
NC_000024.9:g.2888083C>G	2888083	2888083+Q1a1,F746	Q1a1,F746	C>G	C	G
NC_000024.9:g.6629389C>T	6629389	6629389+Q1a1,F790	Q1a1,F790	C>T	C	T
NC_000024.9:g.6735509G>C	6735509	6735509+Q1a1,F818	Q1a1,F818	G>C	G	C
NC_000024.9:g.6980331A>T	6980331	6980331+Q1a1,F896	Q1a1,F896	A>T	A	T
NC_000024.9:g.21301192C>T	21301192	21301192+Q1a1,M7361	Q1a1,M7361	C>T	C	T
NC_000024.9:g.15744594C>T	15744594	15744594+Q1a1,SK1918	Q1a1,SK1918	C>T	C	T
NC_000024.9:g.19001689A>T	19001689	19001689+Q1a1,SK1921	Q1a1,SK1921	A>T	A	T
NC_000024.9:g.16727098C>T	16727098	16727098+Q1a1,Y571	Q1a1,Y571	C>T	C	T
NC_000024.9:g.17787018C>T	17787018	17787018+Q1a1,Y574	Q1a1,Y574	C>T	C	T
NC_000024.9:g.7342387C>T	7342387	7342387+Q1a1,Y577	Q1a1,Y577	C>T	C	T
NC_000024.9:g.9852688T>C	9852688	9852688+Q1a1,Y619	Q1a1,Y619	T>C	T	C
NC_000024.9:g.10068956C>A	10068956	10068956+Q1a1,Y620	Q1a1,Y620	C>A	C	A
NC_000024.9:g.14296376A>G	14296376	14296376+Q1a1,Y629	Q1a1,Y629	A>G	A	G
NC_000024.9:g.14908821T>C	14908821	14908821+Q1a1,Y633	Q1a1,Y633	T>C	T	C
NC_000024.9:g.15440068T>C	15440068	15440068+Q1a1,Y634	Q1a1,Y634	T>C	T	C
NC_000024.9:g.16425791C>A	16425791	16425791+Q1a1,Y641	Q1a1,Y641	C>A	C	A
NC_000024.9:g.16848496C>T	16848496	16848496+Q1a1,Y642	Q1a1,Y642	C>T	C	T
NC_000024.9:g.21887261C>A	21887261	21887261+Q1a1,Y643	Q1a1,Y643	C>A	C	A
NC_000024.9:g.21908693T>C	21908693	21908693+Q1a1,Y644	Q1a1,Y644	T>C	T	C
NC_000024.9:g.22058653G>C	22058653	22058653+Q1a1,Y646	Q1a1,Y646	G>C	G	C
NC_000024.9:g.22566227A>G	22566227	22566227+Q1a1,Y653	Q1a1,Y653	A>G	A	G
NC_000024.9:g.21119463G>A	21119463	21119463+Q1a1,Y667	Q1a1,Y667	G>A	G	A
NC_000024.9:g.15669938C>T	15669938	15669938+Q1a1,Y676	Q1a1,Y676	C>T	C	T
NC_000024.9:g.19245067C>A	19245067	19245067+Q1a1,Y696	Q1a1,Y696	C>A	C	A
NC_000024.9:g.21349520G>T	21349520	21349520+Q1a1,Y699	Q1a1,Y699	G>T	G	T
NC_000024.9:g.21459335C>T	21459335	21459335+Q1a1,Y700	Q1a1,Y700	C>T	C	T
NC_000024.9:g.21484396C>G	21484396	21484396+Q1a1,Y702	Q1a1,Y702	C>G	C	G
NC_000024.9:g.21542665A>G	21542665	21542665+Q1a1,Y704	Q1a1,Y704	A>G	A	G
NC_000024.9:g.2691217C>T	2691217	2691217+Q1a1,Z19137	Q1a1,Z19137	C>T	C	T
NC_000024.9:g.7733229A>G	7733229	7733229+Q1a1,Z19146	Q1a1,Z19146	A>G	A	G
NC_000024.9:g.7946678A>C	7946678	7946678+Q1a1,Z19148	Q1a1,Z19148	A>C	A	C
NC_000024.9:g.8355780G>A	8355780	8355780+Q1a1,Z19149	Q1a1,Z19149	G>A	G	A
NC_000024.9:g.8432920A>C	8432920	8432920+Q1a1,Z19151	Q1a1,Z19151	A>C	A	C
NC_000024.9:g.14842870G>A	14842870	14842870+Q1a1,Z19169	Q1a1,Z19169	G>A	G	A
NC_000024.9:g.14963804A>G	14963804	14963804+Q1a1,Z19171	Q1a1,Z19171	A>G	A	G
NC_000024.9:g.15253735G>A	15253735	15253735+Q1a1,Z19172	Q1a1,Z19172	G>A	G	A
NC_000024.9:g.16547475C>G	16547475	16547475+Q1a1,Z19181	Q1a1,Z19181	C>G	C	G
NC_000024.9:g.17817072T>C	17817072	17817072+Q1a1,Z19186	Q1a1,Z19186	T>C	T	C
NC_000024.9:g.21215167A>G	21215167	21215167+Q1a1,Z19195	Q1a1,Z19195	A>G	A	G
NC_000024.9:g.21797894C>T	21797894	21797894+Q1a1,Z19197	Q1a1,Z19197	C>T	C	T
NC_000024.9:g.22943663T>A	22943663	22943663+Q1a1,Z19209	Q1a1,Z19209	T>A	T	A
NC_000024.9:g.24399072A>C	24399072	24399072+Q1a1,Z19212	Q1a1,Z19212	A>C	A	C
NC_000024.9:g.24475994C>T	24475994	24475994+Q1a1,Z19214	Q1a1,Z19214	C>T	C	T
NC_000024.9:g.22662391G>A	22662391	22662391+Q1a1,Z27417.2	Q1a1,Z27417.2	G>A	G	A
NC_000024.9:g.13658040A>T	13658040	13658040+Q1a1~,Z19160	Q1a1~,Z19160	A>T	A	T
NC_000024.9:g.8129592T>C	8129592	8129592+Q1a1a,F1111	Q1a1a,F1111	T>C	T	C
NC_000024.9:g.8601548G>A	8601548	8601548+Q1a1a,F1340	Q1a1a,F1340	G>A	G	A
NC_000024.9:g.14185141G>T	14185141	14185141+Q1a1a,F1718	Q1a1a,F1718	G>T	G	T
NC_000024.9:g.14770511A>G	14770511	14770511+Q1a1a,F1830	Q1a1a,F1830	A>G	A	G
NC_000024.9:g.14830200G>A	14830200	14830200+Q1a1a,F1843	Q1a1a,F1843	G>A	G	A
NC_000024.9:g.15062719G>C	15062719	15062719+Q1a1a,F1907	Q1a1a,F1907	G>C	G	C
NC_000024.9:g.15077751T>C	15077751	15077751+Q1a1a,F1908	Q1a1a,F1908	T>C	T	C
NC_000024.9:g.15520934C>G	15520934	15520934+Q1a1a,F1966	Q1a1a,F1966	C>G	C	G
NC_000024.9:g.15941917C>T	15941917	15941917+Q1a1a,F2043	Q1a1a,F2043	C>T	C	T
NC_000024.9:g.15945395G>C	15945395	15945395+Q1a1a,F2045	Q1a1a,F2045	G>C	G	C
NC_000024.9:g.15983949A>C	15983949	15983949+Q1a1a,F2060	Q1a1a,F2060	A>C	A	C
NC_000024.9:g.16202425T>C	16202425	16202425+Q1a1a,F2086	Q1a1a,F2086	T>C	T	C
NC_000024.9:g.16211687C>T	16211687	16211687+Q1a1a,F2094	Q1a1a,F2094	C>T	C	T
NC_000024.9:g.16243471A>G	16243471	16243471+Q1a1a,F2103	Q1a1a,F2103	A>G	A	G
NC_000024.9:g.16802392A>C	16802392	16802392+Q1a1a,F2273	Q1a1a,F2273	A>C	A	C
NC_000024.9:g.18840168G>A	18840168	18840168+Q1a1a,F2864	Q1a1a,F2864	G>A	G	A

NC_000024.9:g.18912023G>T	18912023	18912023+Q1a1a,F2893	Q1a1a,F2893	G>T	G	T
NC_000024.9:g.19268036A>T	19268036	19268036+Q1a1a,F2998	Q1a1a,F2998	A>T	A	T
NC_000024.9:g.19332304C>G	19332304	19332304+Q1a1a,F3019	Q1a1a,F3019	C>G	C	G
NC_000024.9:g.19344295G>C	19344295	19344295+Q1a1a,F3022	Q1a1a,F3022	G>C	G	C
NC_000024.9:g.24417419C>G	24417419	24417419+Q1a1a,F3625	Q1a1a,F3625	C>G	C	G
NC_000024.9:g.21104416C>T	21104416	21104416+Q1a1a,F4295	Q1a1a,F4295	C>T	C	T
NC_000024.9:g.7557577C>T	7557577	7557577+Q1a1a,F4710	Q1a1a,F4710	C>T	C	T
NC_000024.9:g.7880184G>A	7880184	7880184+Q1a1a,F4734	Q1a1a,F4734	G>A	G	A
NC_000024.9:g.8733537A>G	8733537	8733537+Q1a1a,F4791	Q1a1a,F4791	A>G	A	G
NC_000024.9:g.9818001G>C	9818001	9818001+Q1a1a,F4831	Q1a1a,F4831	G>C	G	C
NC_000024.9:g.10082411A>G	10082411	10082411+Q1a1a,F4843	Q1a1a,F4843	A>G	A	G
NC_000024.9:g.13684761C>T	13684761	13684761+Q1a1a,F4891	Q1a1a,F4891	C>T	C	T
NC_000024.9:g.14879893G>A	14879893	14879893+Q1a1a,F4977	Q1a1a,F4977	G>A	G	A
NC_000024.9:g.17011560G>T	17011560	17011560+Q1a1a,F5074	Q1a1a,F5074	G>T	G	T
NC_000024.9:g.17484443A>G	17484443	17484443+Q1a1a,F5100	Q1a1a,F5100	A>G	A	G
NC_000024.9:g.18024026G>A	18024026	18024026+Q1a1a,F5129	Q1a1a,F5129	G>A	G	A
NC_000024.9:g.19341911C>A	19341911	19341911+Q1a1a,F5197	Q1a1a,F5197	C>A	C	A
NC_000024.9:g.22031296G>A	22031296	22031296+Q1a1a,F5272	Q1a1a,F5272	G>A	G	A
NC_000024.9:g.22628226T>C	22628226	22628226+Q1a1a,F5350	Q1a1a,F5350	T>C	T	C
NC_000024.9:g.22675027G>A	22675027	22675027+Q1a1a,F5355	Q1a1a,F5355	G>A	G	A
NC_000024.9:g.2886502T>C	2886502	2886502+Q1a1a,F745	Q1a1a,F745	T>C	T	C
NC_000024.9:g.2889760C>T	2889760	2889760+Q1a1a,F750	Q1a1a,F750	C>T	C	T
NC_000024.9:g.7193234T>A	7193234	7193234+Q1a1a,F924	Q1a1a,F924	T>A	T	A
NC_000024.9:g.2888663C>T	2888663	2888663+Q1a1a,L415.2	Q1a1a,L415.2	C>T	C	T
NC_000024.9:g.21907394T>C	21907394	21907394+Q1a1a,M120	Q1a1a,M120	T>C	T	C
NC_000024.9:g.15030650C>A	15030650	15030650+Q1a1a,M265	Q1a1a,M265	C>A	C	A
NC_000024.9:g.17161359G>T	17161359	17161359+Q1a1a,Y572	Q1a1a,Y572	G>T	G	T
NC_000024.9:g.22621254G>T	22621254	22621254+Q1a1a,Y575	Q1a1a,Y575	G>T	G	T
NC_000024.9:g.7137166C>T	7137166	7137166+Q1a1a,Y610	Q1a1a,Y610	C>T	C	T
NC_000024.9:g.7432639C>T	7432639	7432639+Q1a1a,Y611	Q1a1a,Y611	C>T	C	T
NC_000024.9:g.7904858C>T	7904858	7904858+Q1a1a,Y613	Q1a1a,Y613	C>T	C	T
NC_000024.9:g.14537145C>A	14537145	14537145+Q1a1a,Y631	Q1a1a,Y631	C>A	C	A
NC_000024.9:g.15995283G>T	15995283	15995283+Q1a1a,Y637	Q1a1a,Y637	G>T	G	T
NC_000024.9:g.21955751T>C	21955751	21955751+Q1a1a,Y645	Q1a1a,Y645	T>C	T	C
NC_000024.9:g.22121079G>T	22121079	22121079+Q1a1a,Y647	Q1a1a,Y647	G>T	G	T
NC_000024.9:g.23345363T>C	23345363	23345363+Q1a1a,Y656	Q1a1a,Y656	T>C	T	C
NC_000024.9:g.24434213T>C	24434213	24434213+Q1a1a,Y659	Q1a1a,Y659	T>C	T	C
NC_000024.9:g.19308981G>A	19308981	19308981+Q1a1a,Y665	Q1a1a,Y665	G>A	G	A
NC_000024.9:g.23831612G>A	23831612	23831612+Q1a1a,Y668	Q1a1a,Y668	G>A	G	A
NC_000024.9:g.16863801G>T	16863801	16863801+Q1a1a,Y677	Q1a1a,Y677	G>T	G	T
NC_000024.9:g.2865795C>T	2865795	2865795+Q1a1a,Y683	Q1a1a,Y683	C>T	C	T
NC_000024.9:g.17617938G>A	17617938	17617938+Q1a1a,Y691	Q1a1a,Y691	G>A	G	A
NC_000024.9:g.18820049C>G	18820049	18820049+Q1a1a,Y693	Q1a1a,Y693	C>G	C	G
NC_000024.9:g.19243339G>A	19243339	19243339+Q1a1a,Y695	Q1a1a,Y695	G>A	G	A
NC_000024.9:g.19431584G>C	19431584	19431584+Q1a1a,Y697	Q1a1a,Y697	G>C	G	C
NC_000024.9:g.21186974C>T	21186974	21186974+Q1a1a,Y698	Q1a1a,Y698	C>T	C	T
NC_000024.9:g.21473854G>T	21473854	21473854+Q1a1a,Y701	Q1a1a,Y701	G>T	G	T
NC_000024.9:g.21496346A>G	21496346	21496346+Q1a1a,Y703	Q1a1a,Y703	A>G	A	G
NC_000024.9:g.21709263G>A	21709263	21709263+Q1a1a,Y705	Q1a1a,Y705	G>A	G	A
NC_000024.9:g.8410250C>T	8410250	8410250+Q1a1a,Z19150	Q1a1a,Z19150	C>T	C	T
NC_000024.9:g.14157955G>A	14157955	14157955+Q1a1a,Z19167	Q1a1a,Z19167	G>A	G	A
NC_000024.9:g.16318016C>A	16318016	16318016+Q1a1a,Z19177	Q1a1a,Z19177	C>A	C	A
NC_000024.9:g.16447622T>G	16447622	16447622+Q1a1a,Z19178	Q1a1a,Z19178	T>G	T	G
NC_000024.9:g.16523297A>G	16523297	16523297+Q1a1a,Z19180	Q1a1a,Z19180	A>G	A	G
NC_000024.9:g.17175799A>G	17175799	17175799+Q1a1a,Z19184	Q1a1a,Z19184	A>G	A	G
NC_000024.9:g.19128440T>C	19128440	19128440+Q1a1a,Z19191	Q1a1a,Z19191	T>C	T	C
NC_000024.9:g.22020659G>C	22020659	22020659+Q1a1a,Z19198	Q1a1a,Z19198	G>C	G	C
NC_000024.9:g.22054395G>A	22054395	22054395+Q1a1a,Z19200	Q1a1a,Z19200	G>A	G	A
NC_000024.9:g.22912856G>T	22912856	22912856+Q1a1a,Z19208	Q1a1a,Z19208	G>T	G	T
NC_000024.9:g.23404344G>A	23404344	23404344+Q1a1a,Z19210	Q1a1a,Z19210	G>A	G	A
NC_000024.9:g.24417504G>T	24417504	24417504+Q1a1a,Z19213	Q1a1a,Z19213	G>T	G	T
NC_000024.9:g.22643901A>T	22643901	22643901+Q1a1a~,Y654	Q1a1a~,Y654	A>T	A	T
NC_000024.9:g.7001509C>T	7001509	7001509+Q1a1a2,M7417	Q1a1a2,M7417	C>T	C	T
NC_000024.9:g.7834763G>A	7834763	7834763+Q1a1a2,Z35919	Q1a1a2,Z35919	G>A	G	A
NC_000024.9:g.21973806G>A	21973806	21973806+Q1a1b,Z36031	Q1a1b,Z36031	G>A	G	A
NC_000024.9:g.15026112G>A	15026112	15026112+Q1a2a1b~,BZ648	Q1a2a1b~,BZ648	G>A	G	A
NC_000024.9:g.16868573C>T	16868573	16868573+Q1a2a2b~,Y22391	Q1a2a2b~,Y22391	C>T	C	T
NC_000024.9:g.8558847G>C	8558847	8558847+Q1a2a2b1a~,BZ1021	Q1a2a2b1a~,BZ1021	G>C	G	C
NC_000024.9:g.8483035G>C	8483035	8483035+Q1b,F1242	Q1b,F1242	G>C	G	C
NC_000024.9:g.10053142C>T	10053142	10053142+Q1b,FGC6791	Q1b,FGC6791	C>T	C	T
NC_000024.9:g.24470083A>C	24470083	24470083+Q1b,M1057	Q1b,M1057	A>C	A	C
NC_000024.9:g.7688075T>C	7688075	7688075+Q1b,M1072	Q1b,M1072	T>C	T	C
NC_000024.9:g.8131242C>T	8131242	8131242+Q1b,M966	Q1b,M966	C>T	C	T
NC_000024.9:g.8148869G>A	8148869	8148869+Q1b,S324	Q1b,S324	G>A	G	A
NC_000024.9:g.8493022G>A	8493022	8493022+Q1b,V2131	Q1b,V2131	G>A	G	A
NC_000024.9:g.23771830A>G	23771830	23771830+Q1b,Y713	Q1b,Y713	A>G	A	G
NC_000024.9:g.8130510G>A	8130510	8130510+Q1b,Y728	Q1b,Y728	G>A	G	A
NC_000024.9:g.9831756C>T	9831756	9831756+Q1b,Y732	Q1b,Y732	C>T	C	T
NC_000024.9:g.10002452A>G	10002452	10002452+Q1b,Y733	Q1b,Y733	A>G	A	G
NC_000024.9:g.13607474C>T	13607474	13607474+Q1b,Y737	Q1b,Y737	C>T	C	T
NC_000024.9:g.8235033C>G	8235033	8235033+Q1b1,L213	Q1b1,L213	C>G	C	G
NC_000024.9:g.16396131G>A	16396131	16396131+Q1b1~,CTS5619	Q1b1~,CTS5619	G>A	G	A
NC_000024.9:g.7903145C>T	7903145	7903145+Q1b1~,M1073	Q1b1~,M1073	C>T	C	T
NC_000024.9:g.17996448A>G	17996448	17996448+Q1b1~,Y712	Q1b1~,Y712	A>G	A	G
NC_000024.9:g.21624818G>C	21624818	21624818+Q1b1~,Y747	Q1b1~,Y747	G>C	G	C
NC_000024.9:g.23557435C>T	23557435	23557435+Q1b1~,Y762	Q1b1~,Y762	C>T	C	T
NC_000024.9:g.23567021G>C	23567021	23567021+Q1b1~,Y763	Q1b1~,Y763	G>C	G	C
NC_000024.9:g.9937276C>T	9937276	9937276+Q1b1a1a,FGC8414	Q1b1a1a,FGC8414	C>T	C	T
NC_000024.9:g.7955584G>A	7955584	7955584+Q1b1a1a,M836	Q1b1a1a,M836	G>A	G	A
NC_000024.9:g.23073760C>A	23073760	23073760+Q1b1a1a1e1,CTS11330	Q1b1a1a1e1,CTS11330	C>A	C	A
NC_000024.9:g.7136391T>G	7136391	7136391+Q1b1a1a1e1a,CTS1002	Q1b1a1a1e1a,CTS1002	T>G	T	G
NC_000024.9:g.19420238G>T	19420238	19420238+Q1b1a1a1e1a,CTS10359	Q1b1a1a1e1a,CTS10359	G>T	G	T
NC_000024.9:g.22773363A>G	22773363	22773363+Q1b1a1a1e1a,CTS10779	Q1b1a1a1e1a,CTS10779	A>G	A	G
NC_000024.9:g.22894819G>T	22894819	22894819+Q1b1a1a1e1a,CTS11007	Q1b1a1a1e1a,CTS11007	G>T	G	T
NC_000024.9:g.23051019C>T	23051019	23051019+Q1b1a1a1e1a,CTS11283^	Q1b1a1a1e1a,CTS11283^	C>T	C	T
NC_000024.9:g.23186289T>G	23186289	23186289+Q1b1a1a1e1a,CTS11601	Q1b1a1a1e1a,CTS11601	T>G	T	G
NC_000024.9:g.14129152T>A	14129152	14129152+Q1b1a1a1e1a,CTS1941	Q1b1a1a1e1a,CTS1941	T>A	T	A
NC_000024.9:g.2678179T>G	2678179	2678179+Q1b1a1a1e1a,CTS30	Q1b1a1a1e1a,CTS30	T>G	T	G
NC_000024.9:g.14836804A>G	14836804	14836804+Q1b1a1a1e1a,CTS3328	Q1b1a1a1e1a,CTS3328	A>G	A	G
NC_000024.9:g.16003907G>T	16003907	16003907+Q1b1a1a1e1a,CTS5079	Q1b1a1a1e1a,CTS5079	G>T	G	T
NC_000024.9:g.17203137C>A	17203137	17203137+Q1b1a1a1e1a,CTS6940	Q1b1a1a1e1a,CTS6940	C>A	C	A
NC_000024.9:g.17322314G>A	17322314	17322314+Q1b1a1a1e1a,CTS7148	Q1b1a1a1e1a,CTS7148	G>A	G	A
NC_000024.9:g.17729335G>A	17729335	17729335+Q1b1a1a1e1a,CTS7901	Q1b1a1a1e1a,CTS7901	G>A	G	A
NC_000024.9:g.18086946A>G	18086946	18086946+Q1b1a1a1e1a,CTS8567	Q1b1a1a1e1a,CTS8567	A>G	A	G

NC_000024.9:g.18755424A>G	18755424	18755424+Q1b1a1a1e1a,CTS9220	Q1b1a1a1e1a,CTS9220	A>G	A	G
NC_000024.9:g.18962747C>G	18962747	18962747+Q1b1a1a1e1a,CTS9559	Q1b1a1a1e1a,CTS9559	C>G	C	G
NC_000024.9:g.15519861A>G	15519861	15519861+Q1b1a1a1e1a,M859	Q1b1a1a1e1a,M859	A>G	A	G
NC_000024.9:g.14454319C>T	14454319	14454319+Q1b1a1a1e1a,M859_eq	Q1b1a1a1e1a,M859_eq	C>T	C	T
NC_000024.9:g.14216495A>G	14216495	14216495+Q1b1a1a1e1a,Z771	Q1b1a1a1e1a,Z771	A>G	A	G
NC_000024.9:g.22001909C>T	22001909	22001909+Q1b1a1a1e1a,Z775	Q1b1a1a1e1a,Z775	C>T	C	T
NC_000024.9:g.15825550G>T	15825550	15825550+Q1b1a1a1e1a~,CTS4795	Q1b1a1a1e1a~,CTS4795	G>T	G	T
NC_000024.9:g.24507766T>A	24507766	24507766+Q1b1a1a1e1a~,Z768	Q1b1a1a1e1a~,Z768	T>A	T	A
NC_000024.9:g.6777980G>C	6777980	6777980+Q1b1a1a1e1a1,CTS479	Q1b1a1a1e1a1,CTS479	G>C	G	C
NC_000024.9:g.14315959C>T	14315959	14315959+Q1b1a1a1e1a1~,CTS2462	Q1b1a1a1e1a1~,CTS2462	C>T	C	T
NC_000024.9:g.22807063G>A	22807063	22807063+Q1b1a1a1e1b~,CTS10853	Q1b1a1a1e1b~,CTS10853	G>A	G	A
NC_000024.9:g.22905230G>A	22905230	22905230+Q1b1a1a1e1b~,CTS11027	Q1b1a1a1e1b~,CTS11027	G>A	G	A
NC_000024.9:g.22926178G>T	22926178	22926178+Q1b1a1a1e1b~,CTS11064	Q1b1a1a1e1b~,CTS11064	G>T	G	T
NC_000024.9:g.17403120G>A	17403120	17403120+Q1b1a1a1e1b~,CTS7291	Q1b1a1a1e1b~,CTS7291	G>A	G	A
NC_000024.9:g.18958625C>T	18958625	18958625+Q1b1a1a1e1b~,CTS9550	Q1b1a1a1e1b~,CTS9550	C>T	C	T
NC_000024.9:g.16644000C>T	16644000	16644000+Q1b1a1a1e1b1~,CTS6060	Q1b1a1a1e1b1~,CTS6060	C>T	C	T
NC_000024.9:g.17291624T>A	17291624	17291624+Q1b1a1a1e1b1~,CTS7101	Q1b1a1a1e1b1~,CTS7101	T>A	T	A
NC_000024.9:g.17680998T>A	17680998	17680998+Q1b1a1a1e1b1~,CTS7811	Q1b1a1a1e1b1~,CTS7811	T>A	T	A
NC_000024.9:g.19218783G>A	19218783	19218783+Q1b1a1a1e1b1a~,CTS10033	Q1b1a1a1e1b1a~,CTS10033	G>A	G	A
NC_000024.9:g.23420061C>A	23420061	23420061+Q1b1a1a1e1b1a~,CTS12013	Q1b1a1a1e1b1a~,CTS12013	C>A	C	A
NC_000024.9:g.15667681A>G	15667681	15667681+Q1b1a1a1e1b1a~,CTS4481	Q1b1a1a1e1b1a~,CTS4481	A>G	A	G
NC_000024.9:g.16390248C>T	16390248	16390248+Q1b1a1a1e1b1a~,CTS5603	Q1b1a1a1e1b1a~,CTS5603	C>T	C	T
NC_000024.9:g.22664280G>C	22664280	22664280+Q1b1a1a1e1c~,CTS10623	Q1b1a1a1e1c~,CTS10623	G>C	G	C
NC_000024.9:g.17470857C>T	17470857	17470857+Q1b1a1a1e1c~,CTS7417	Q1b1a1a1e1c~,CTS7417	C>T	C	T
NC_000024.9:g.14282049G>T	14282049	14282049+Q1b1a1a1e1c~,Y10782	Q1b1a1a1e1c~,Y10782	G>T	G	T
NC_000024.9:g.21103672T>C	21103672	21103672+Q1b1a1a1e1c~,Y10784	Q1b1a1a1e1c~,Y10784	T>C	T	C
NC_000024.9:g.22918083A>T	22918083	22918083+Q1b1a1a1e1c~,Y10785	Q1b1a1a1e1c~,Y10785	A>T	A	T
NC_000024.9:g.15311307T>G	15311307	15311307+Q1b1a1a1e1c~,Y11652	Q1b1a1a1e1c~,Y11652	T>G	T	G
NC_000024.9:g.17622880G>A	17622880	17622880+Q1b1a1a1e1c~,Y11653	Q1b1a1a1e1c~,Y11653	G>A	G	A
NC_000024.9:g.8212180C>A	8212180	8212180+Q1b1a1a1e1c~,Y11656	Q1b1a1a1e1c~,Y11656	C>A	C	A
NC_000024.9:g.17015428C>T	17015428	17015428+Q1b1a1a1e1c~,Y11657	Q1b1a1a1e1c~,Y11657	C>T	C	T
NC_000024.9:g.17325723A>T	17325723	17325723+Q1b1a1a1e1c~,Y11658	Q1b1a1a1e1c~,Y11658	A>T	A	T
NC_000024.9:g.19006520A>C	19006520	19006520+Q1b1a1a1e1c~,Y16654	Q1b1a1a1e1c~,Y16654	A>C	A	C
NC_000024.9:g.7008283A>G	7008283	7008283+Q1b1a1a1e2,Y12424	Q1b1a1a1e2,Y12424	A>G	A	G
NC_000024.9:g.9423277C>T	9423277	9423277+Q1b1a1a1e2,Y12428	Q1b1a1a1e2,Y12428	C>T	C	T
NC_000024.9:g.15418465G>A	15418465	15418465+Q1b1a1a1e2,Y12431	Q1b1a1a1e2,Y12431	G>A	G	A
NC_000024.9:g.2851828A>G	2851828	2851828+Q1b1a1a1e2,Z19548	Q1b1a1a1e2,Z19548	A>G	A	G
NC_000024.9:g.7579219G>A	7579219	7579219+Q1b1a1a1e2,Z19581	Q1b1a1a1e2,Z19581	G>A	G	A
NC_000024.9:g.7868237A>T	7868237	7868237+Q1b1a1a1e2,Z19583	Q1b1a1a1e2,Z19583	A>T	A	T
NC_000024.9:g.14473175C>T	14473175	14473175+Q1b1a1a1e2,Z19612	Q1b1a1a1e2,Z19612	C>T	C	T
NC_000024.9:g.16189725C>T	16189725	16189725+Q1b1a1a1e2,Z19628	Q1b1a1a1e2,Z19628	C>T	C	T
NC_000024.9:g.23332061G>A	23332061	23332061+Q1b1a1a1e2,Z19667	Q1b1a1a1e2,Z19667	G>A	G	A
NC_000024.9:g.24502838C>T	24502838	24502838+Q1b1a1a1e3~,BZ270	Q1b1a1a1e3~,BZ270	C>T	C	T
NC_000024.9:g.7548499A>G	7548499	7548499+Q1b1a1a1e3~,SK1973	Q1b1a1a1e3~,SK1973	A>G	A	G
NC_000024.9:g.7558554G>A	7558554	7558554+Q1b1a1a1e3~,SK1974	Q1b1a1a1e3~,SK1974	G>A	G	A
NC_000024.9:g.15979832A>G	15979832	15979832+Q1b1a1a1e3~,SK1976	Q1b1a1a1e3~,SK1976	A>G	A	G
NC_000024.9:g.7654363G>A	7654363	7654363+Q1b1a1a1e3~,Y26551	Q1b1a1a1e3~,Y26551	G>A	G	A
NC_000024.9:g.17374774G>C	17374774	17374774+Q1b1a1a1e3~,Y26591	Q1b1a1a1e3~,Y26591	G>C	G	C
NC_000024.9:g.7180234C>T	7180234	7180234+Q1b1a1a1h1,Z19461	Q1b1a1a1h1,Z19461	C>T	C	T
NC_000024.9:g.8298339G>C	8298339	8298339+Q1b1a1a1h1,Z19464	Q1b1a1a1h1,Z19464	G>C	G	C
NC_000024.9:g.8366399G>T	8366399	8366399+Q1b1a1a1h1,Z19465	Q1b1a1a1h1,Z19465	G>T	G	T
NC_000024.9:g.8591059A>G	8591059	8591059+Q1b1a1a1h1,Z19466	Q1b1a1a1h1,Z19466	A>G	A	G
NC_000024.9:g.13667499A>C	13667499	13667499+Q1b1a1a1h1,Z19475	Q1b1a1a1h1,Z19475	A>C	A	C
NC_000024.9:g.23783003T>C	23783003	23783003+Q1b1a1a1h1,Z19515	Q1b1a1a1h1,Z19515	T>C	T	C
NC_000024.9:g.23808064G>A	23808064	23808064+Q1b1a1a1h1,Z19517	Q1b1a1a1h1,Z19517	G>A	G	A
NC_000024.9:g.7510095T>C	7510095	7510095+Q1b1a1a1h1,Z35460	Q1b1a1a1h1,Z35460	T>C	T	C
NC_000024.9:g.8433027C>T	8433027	8433027+Q1b1a1a1i,B48	Q1b1a1a1i,B48	C>T	C	T
NC_000024.9:g.8871665C>G	8871665	8871665+Q1b1a1a1i,Z19295	Q1b1a1a1i,Z19295	C>G	C	G
NC_000024.9:g.7899234C>T	7899234	7899234+Q1b1a1a1i1a,Z19320	Q1b1a1a1i1a,Z19320	C>T	C	T
NC_000024.9:g.8592765A>G	8592765	8592765+Q1b1a1a1i1a,Z19321	Q1b1a1a1i1a,Z19321	A>G	A	G
NC_000024.9:g.14631239G>A	14631239	14631239+Q1b1a1a1i1a~,Z19322	Q1b1a1a1i1a~,Z19322	G>A	G	A
NC_000024.9:g.8483906G>C	8483906	8483906+Q1b1a1a1i1a1,Z19325	Q1b1a1a1i1a1,Z19325	G>C	G	C
NC_000024.9:g.8539880G>T	8539880	8539880+Q1b1a1a1i1a1,Z19326	Q1b1a1a1i1a1,Z19326	G>T	G	T
NC_000024.9:g.9908136C>T	9908136	9908136+Q1b1a1a1i1a1,Z19327	Q1b1a1a1i1a1,Z19327	C>T	C	T
NC_000024.9:g.8067992T>C	8067992	8067992+Q1b1a1a1i1a1a,Z19338	Q1b1a1a1i1a1a,Z19338	T>C	T	C
NC_000024.9:g.8445140G>T	8445140	8445140+Q1b1a1a1i1a1a,Z19339	Q1b1a1a1i1a1a,Z19339	G>T	G	T
NC_000024.9:g.8203194A>C	8203194	8203194+Q1b1a1a1i1a1a,Z35909	Q1b1a1a1i1a1a,Z35909	A>C	A	C
NC_000024.9:g.7698498T>C	7698498	7698498+Q1b1a1a1i1a2,Z35921	Q1b1a1a1i1a2,Z35921	T>C	T	C
NC_000024.9:g.14193916G>A	14193916	14193916+Q1b1a1a1j,Y782	Q1b1a1a1j,Y782	G>A	G	A
NC_000024.9:g.15257564A>G	15257564	15257564+Q1b1a1a1j,Y785	Q1b1a1a1j,Y785	A>G	A	G
NC_000024.9:g.16556737C>T	16556737	16556737+Q1b1a1a1j,Y786	Q1b1a1a1j,Y786	C>T	C	T
NC_000024.9:g.23348002C>A	23348002	23348002+Q1b1a1a1j,Y788	Q1b1a1a1j,Y788	C>A	C	A
NC_000024.9:g.8224305C>A	8224305	8224305+Q1b1a1a1j,Y792	Q1b1a1a1j,Y792	C>A	C	A
NC_000024.9:g.7819978A>C	7819978	7819978+Q1b1a1a1j,Z19354	Q1b1a1a1j,Z19354	A>C	A	C
NC_000024.9:g.7878556C>A	7878556	7878556+Q1b1a1a1j,Z19355	Q1b1a1a1j,Z19355	C>A	C	A
NC_000024.9:g.15096944G>C	15096944	15096944+Q1b1a1a1j,Z19363	Q1b1a1a1j,Z19363	G>C	G	C
NC_000024.9:g.15792662T>C	15792662	15792662+Q1b1a1a1j,Z19366	Q1b1a1a1j,Z19366	T>C	T	C
NC_000024.9:g.7164117C>T	7164117	7164117+Q1b1a1a1j,Z5913	Q1b1a1a1j,Z5913	C>T	C	T
NC_000024.9:g.21077916C>T	21077916	21077916+Q1b1a1a1j1,Y793	Q1b1a1a1j1,Y793	C>T	C	T
NC_000024.9:g.28542555C>T	28542555	28542555+Q1b1a1a1j1,Y795	Q1b1a1a1j1,Y795	C>T	C	T
NC_000024.9:g.7250331T>C	7250331	7250331+Q1b1a1a1j1,Y807	Q1b1a1a1j1,Y807	T>C	T	C
NC_000024.9:g.7250351C>T	7250351	7250351+Q1b1a1a1j1,Y808	Q1b1a1a1j1,Y808	C>T	C	T
NC_000024.9:g.7276422A>G	7276422	7276422+Q1b1a1a1j1,Y809	Q1b1a1a1j1,Y809	A>G	A	G
NC_000024.9:g.14023757C>T	14023757	14023757+Q1b1a1a1j1,Y816	Q1b1a1a1j1,Y816	C>T	C	T
NC_000024.9:g.14043472G>T	14043472	14043472+Q1b1a1a1j1,Y817	Q1b1a1a1j1,Y817	G>T	G	T
NC_000024.9:g.14442245G>T	14442245	14442245+Q1b1a1a1j1,Y819	Q1b1a1a1j1,Y819	G>T	G	T
NC_000024.9:g.14891157T>A	14891157	14891157+Q1b1a1a1j1,Y820	Q1b1a1a1j1,Y820	T>A	T	A
NC_000024.9:g.15733176C>T	15733176	15733176+Q1b1a1a1j1,Y821	Q1b1a1a1j1,Y821	C>T	C	T
NC_000024.9:g.15788323C>A	15788323	15788323+Q1b1a1a1j1,Y822	Q1b1a1a1j1,Y822	C>A	C	A
NC_000024.9:g.15943510T>C	15943510	15943510+Q1b1a1a1j1,Y823	Q1b1a1a1j1,Y823	T>C	T	C
NC_000024.9:g.16267326A>C	16267326	16267326+Q1b1a1a1j1,Y825	Q1b1a1a1j1,Y825	A>C	A	C
NC_000024.9:g.16476835T>C	16476835	16476835+Q1b1a1a1j1,Y827	Q1b1a1a1j1,Y827	T>C	T	C
NC_000024.9:g.17085420C>G	17085420	17085420+Q1b1a1a1j1,Y829	Q1b1a1a1j1,Y829	C>G	C	G
NC_000024.9:g.19342429G>A	19342429	19342429+Q1b1a1a1j1,Y833	Q1b1a1a1j1,Y833	G>A	G	A
NC_000024.9:g.21288111A>G	21288111	21288111+Q1b1a1a1j1,Y834	Q1b1a1a1j1,Y834	A>G	A	G
NC_000024.9:g.21496826C>A	21496826	21496826+Q1b1a1a1j1,Y835	Q1b1a1a1j1,Y835	C>A	C	A
NC_000024.9:g.21986005C>T	21986005	21986005+Q1b1a1a1j1,Y836	Q1b1a1a1j1,Y836	C>T	C	T
NC_000024.9:g.22990641C>G	22990641	22990641+Q1b1a1a1j1,Y845	Q1b1a1a1j1,Y845	C>G	C	G
NC_000024.9:g.23003075C>T	23003075	23003075+Q1b1a1a1j1,Y846	Q1b1a1a1j1,Y846	C>T	C	T
NC_000024.9:g.23098756G>A	23098756	23098756+Q1b1a1a1j1,Y847	Q1b1a1a1j1,Y847	G>A	G	A
NC_000024.9:g.23393585A>G	23393585	23393585+Q1b1a1a1j1,Y849	Q1b1a1a1j1,Y849	A>G	A	G
NC_000024.9:g.28615064A>G	28615064	28615064+Q1b1a1a1j1,Y851	Q1b1a1a1j1,Y851	A>G	A	G
NC_000024.9:g.18190832G>T	18190832	18190832+Q1b1a1a1j1,Y855	Q1b1a1a1j1,Y855	G>T	G	T
NC_000024.9:g.7877382T>C	7877382	7877382+Q1b1a1a1j1,Z19373	Q1b1a1a1j1,Z19373	T>C	T	C

NC_000024.9:g.8297991G>C	8297991	8297991+Q1b1a1a1j1,Z19375	Q1b1a1a1j1,Z19375	G>C	G	C
NC_000024.9:g.8875283G>A	8875283	8875283+Q1b1a1a1j1,Z19379	Q1b1a1a1j1,Z19379	G>A	G	A
NC_000024.9:g.9523019C>A	9523019	9523019+Q1b1a1a1j1,Z19381	Q1b1a1a1j1,Z19381	C>A	C	A
NC_000024.9:g.14899209G>A	14899209	14899209+Q1b1a1a1j1,Z19395	Q1b1a1a1j1,Z19395	G>A	G	A
NC_000024.9:g.15589260G>C	15589260	15589260+Q1b1a1a1j1,Z19397	Q1b1a1a1j1,Z19397	G>C	G	C
NC_000024.9:g.23523760T>C	23523760	23523760+Q1b1a1a1j1,Z19421	Q1b1a1a1j1,Z19421	T>C	T	C
NC_000024.9:g.7595831T>C	7595831	7595831+Q1b1a1a1j1,Z19427	Q1b1a1a1j1,Z19427	T>C	T	C
NC_000024.9:g.9107914A>G	9107914	9107914+Q1b1a1a1j1,Z35632	Q1b1a1a1j1,Z35632	A>G	A	G
NC_000024.9:g.2699718G>A	2699718	2699718+Q1b1a1a1j1,Z5914	Q1b1a1a1j1,Z5914	G>A	G	A
NC_000024.9:g.14052814T>A	14052814	14052814+Q1b1a1a1j1a,Y818	Q1b1a1a1j1a,Y818	T>A	T	A
NC_000024.9:g.17600962T>A	17600962	17600962+Q1b1a1a1j1a,Y830	Q1b1a1a1j1a,Y830	T>A	T	A
NC_000024.9:g.19071189C>T	19071189	19071189+Q1b1a1a1j1a,Y832	Q1b1a1a1j1a,Y832	C>T	C	T
NC_000024.9:g.8216604T>C	8216604	8216604+Q1b1a1a1k1,Z19429	Q1b1a1a1k1,Z19429	T>C	T	C
NC_000024.9:g.22177431G>A	22177431	22177431+Q1b1a1a1k1,Z19433	Q1b1a1a1k1,Z19433	G>A	G	A
NC_000024.9:g.14918316C>A	14918316	14918316+Q1b1a1a1k1,Z5915	Q1b1a1a1k1,Z5915	C>A	C	A
NC_000024.9:g.17733196G>A	17733196	17733196+Q1b1a1a1k1a,Z19435	Q1b1a1a1k1a,Z19435	G>A	G	A
NC_000024.9:g.22045347T>C	22045347	22045347+Q1b1a1a1k1a,Z19436	Q1b1a1a1k1a,Z19436	T>C	T	C
NC_000024.9:g.22693191A>C	22693191	22693191+Q1b1a1a1k1a,Z19437	Q1b1a1a1k1a,Z19437	A>C	A	C
NC_000024.9:g.16704121G>C	16704121	16704121+Q1b1a1a1k1a,Z5916	Q1b1a1a1k1a,Z5916	G>C	G	C
NC_000024.9:g.8482051G>C	8482051	8482051+Q1b1a1a1l,SK281	Q1b1a1a1l,SK281	G>C	G	C
NC_000024.9:g.6945309G>A	6945309	6945309+Q1b1a1a1l1~,Z35727	Q1b1a1a1l1~,Z35727	G>A	G	A
NC_000024.9:g.7035979A>G	7035979	7035979+Q1b1a1a1l1~,Z35728	Q1b1a1a1l1~,Z35728	A>G	A	G
NC_000024.9:g.7383110C>T	7383110	7383110+Q1b1a1a1l1~,Z35729	Q1b1a1a1l1~,Z35729	C>T	C	T
NC_000024.9:g.7880052G>A	7880052	7880052+Q1b1a1a1l1~,Z35731	Q1b1a1a1l1~,Z35731	G>A	G	A
NC_000024.9:g.14449543A>G	14449543	14449543+Q1b1a1a1m,CTS2731	Q1b1a1a1m,CTS2731	A>G	A	G
NC_000024.9:g.18088531C>T	18088531	18088531+Q1b1a1a1m2~,CTS8571	Q1b1a1a1m2~,CTS8571	C>T	C	T
NC_000024.9:g.17273121G>A	17273121	17273121+Q1b1a1a1m2~,Y26508	Q1b1a1a1m2~,Y26508	G>A	G	A
NC_000024.9:g.18771282A>G	18771282	18771282+Q1b1a1a1m2~,Y26522	Q1b1a1a1m2~,Y26522	A>G	A	G
NC_000024.9:g.21083582C>T	21083582	21083582+Q1b1a1a1m2~,Y26531	Q1b1a1a1m2~,Y26531	C>T	C	T
NC_000024.9:g.14834663G>T	14834663	14834663+Q1b1a1a1m2~,Y28017	Q1b1a1a1m2~,Y28017	G>T	G	T
NC_000024.9:g.2902697T>C	2902697	2902697+Q1b1a1a1m2b~,Y26467	Q1b1a1a1m2b~,Y26467	T>C	T	C
NC_000024.9:g.8071637C>T	8071637	8071637+Q1b1a1a1m2b~,Y26480	Q1b1a1a1m2b~,Y26480	C>T	C	T
NC_000024.9:g.9446993C>T	9446993	9446993+Q1b1a1a1m2b~,Y26485	Q1b1a1a1m2b~,Y26485	C>T	C	T
NC_000024.9:g.17246688C>A	17246688	17246688+Q1b1a1a1m2b~,Y26507	Q1b1a1a1m2b~,Y26507	C>A	C	A
NC_000024.9:g.24404756G>A	24404756	24404756+Q1b1a1a1m2b~,Y26545	Q1b1a1a1m2b~,Y26545	G>A	G	A
NC_000024.9:g.7625240T>A	7625240	7625240+Q1b1a1a1n~,Y27993	Q1b1a1a1n~,Y27993	T>A	T	A
NC_000024.9:g.2693291A>G	2693291	2693291+Q1b1a1a1p,Z35492	Q1b1a1a1p,Z35492	A>G	A	G
NC_000024.9:g.7774054C>T	7774054	7774054+Q1b1a1a1p,Z35500	Q1b1a1a1p,Z35500	C>T	C	T
NC_000024.9:g.8612578C>G	8612578	8612578+Q1b1a1a1p,Z35506	Q1b1a1a1p,Z35506	C>G	C	G
NC_000024.9:g.8841107C>T	8841107	8841107+Q1b1a1a1p,Z35507	Q1b1a1a1p,Z35507	C>T	C	T
NC_000024.9:g.8895749C>T	8895749	8895749+Q1b1a1a1p,Z35508	Q1b1a1a1p,Z35508	C>T	C	T
NC_000024.9:g.15253292G>A	15253292	15253292+Q1b1a1a1p,Z35522	Q1b1a1a1p,Z35522	G>A	G	A
NC_000024.9:g.8467139G>T	8467139	8467139+Q1b1a1a1v~,BZ3401	Q1b1a1a1v~,BZ3401	G>T	G	T
NC_000024.9:g.9108326G>T	9108326	9108326+Q1b1a1a1v~,BZ3407	Q1b1a1a1v~,BZ3407	G>T	G	T
NC_000024.9:g.19468776C>T	19468776	19468776+Q1b1a1a1v~,BZ3477	Q1b1a1a1v~,BZ3477	C>T	C	T
NC_000024.9:g.21842067C>A	21842067	21842067+Q1b1a1a1v~,SK1981	Q1b1a1a1v~,SK1981	C>A	C	A
NC_000024.9:g.8208661A>G	8208661	8208661+Q1b1a1a2b2~,FGC8427	Q1b1a1a2b2~,FGC8427	A>G	A	G
NC_000024.9:g.18687079T>C	18687079	18687079+Q1b1a1a2b2~,FGC8474	Q1b1a1a2b2~,FGC8474	T>C	T	C
NC_000024.9:g.19467575A>G	19467575	19467575+Q1b1a1a2b2~,FGC8480	Q1b1a1a2b2~,FGC8480	A>G	A	G
NC_000024.9:g.19008020G>A	19008020	19008020+Q1b1a1a2b2~,Y4280	Q1b1a1a2b2~,Y4280	G>A	G	A
NC_000024.9:g.19182500A>C	19182500	19182500+Q1b1a1a2b2~,Y4281	Q1b1a1a2b2~,Y4281	A>C	A	C
NC_000024.9:g.7841244G>T	7841244	7841244+Q1b1a1a2b2~,Y4287	Q1b1a1a2b2~,Y4287	G>T	G	T
NC_000024.9:g.7983379C>A	7983379	7983379+Q1b1a1a2b2~,Y4288	Q1b1a1a2b2~,Y4288	C>A	C	A
NC_000024.9:g.15174347C>T	15174347	15174347+Q1b1a1a2b2~,Y4298	Q1b1a1a2b2~,Y4298	C>T	C	T
NC_000024.9:g.15902939G>A	15902939	15902939+Q1b1a1b~,PH1888	Q1b1a1b~,PH1888	G>A	G	A
NC_000024.9:g.8510540C>T	8510540	8510540+Q1b1a2,M971	Q1b1a2,M971	C>T	C	T
NC_000024.9:g.24482276A>G	24482276	24482276+Q1b1a2,Y2804	Q1b1a2,Y2804	A>G	A	G
NC_000024.9:g.17688363T>A	17688363	17688363+Q1b1a2~,CTS7827	Q1b1a2~,CTS7827	T>A	T	A
NC_000024.9:g.13209278C>T	13209278	13209278+Q1b1a2~,Z19276	Q1b1a2~,Z19276	C>T	C	T
NC_000024.9:g.6589670C>T	6589670	6589670+Q1b1a2a~,Y2808	Q1b1a2a~,Y2808	C>T	C	T
NC_000024.9:g.23997774T>C	23997774	23997774+Q1b1a2a~,Y2831	Q1b1a2a~,Y2831	T>C	T	C
NC_000024.9:g.7412517A>T	7412517	7412517+Q1b1a2a~,Z19272	Q1b1a2a~,Z19272	A>T	A	T
NC_000024.9:g.8642911C>T	8642911	8642911+Q1b1a2a~,Z781	Q1b1a2a~,Z781	C>T	C	T
NC_000024.9:g.15072936T>C	15072936	15072936+Q1b1a2a~,Z786	Q1b1a2a~,Z786	T>C	T	C
NC_000024.9:g.9058440G>A	9058440	9058440+Q1b1a2a1,Y2816	Q1b1a2a1,Y2816	G>A	G	A
NC_000024.9:g.7796887T>A	7796887	7796887+Q1b1a2a1a1a1~,Z778	Q1b1a2a1a1a1~,Z778	T>A	T	A
NC_000024.9:g.19231917T>C	19231917	19231917+Q1b1a2a1b,CTS10056	Q1b1a2a1b,CTS10056	T>C	T	C
NC_000024.9:g.22803682A>G	22803682	22803682+Q1b1a2a1b,CTS10848	Q1b1a2a1b,CTS10848	A>G	A	G
NC_000024.9:g.22861526G>T	22861526	22861526+Q1b1a2a1b,CTS10970	Q1b1a2a1b,CTS10970	G>T	G	T
NC_000024.9:g.23054731C>T	23054731	23054731+Q1b1a2a1b,CTS11286	Q1b1a2a1b,CTS11286	C>T	C	T
NC_000024.9:g.15042067C>T	15042067	15042067+Q1b1a2a1b,CTS3582	Q1b1a2a1b,CTS3582	C>T	C	T
NC_000024.9:g.15665594A>G	15665594	15665594+Q1b1a2a1b,CTS4476	Q1b1a2a1b,CTS4476	A>G	A	G
NC_000024.9:g.6964852C>T	6964852	6964852+Q1b1a2a1b,CTS705	Q1b1a2a1b,CTS705	C>T	C	T
NC_000024.9:g.17413954G>A	17413954	17413954+Q1b1a2a1b,CTS7307	Q1b1a2a1b,CTS7307	G>A	G	A
NC_000024.9:g.17550593C>A	17550593	17550593+Q1b1a2a1b,CTS7595	Q1b1a2a1b,CTS7595	C>A	C	A
NC_000024.9:g.17651890T>C	17651890	17651890+Q1b1a2a1b,CTS7769	Q1b1a2a1b,CTS7769	T>C	T	C
NC_000024.9:g.17962817T>C	17962817	17962817+Q1b1a2a1b,CTS8371	Q1b1a2a1b,CTS8371	T>C	T	C
NC_000024.9:g.18068157G>A	18068157	18068157+Q1b1a2a1b,CTS8525	Q1b1a2a1b,CTS8525	G>A	G	A
NC_000024.9:g.18083322A>G	18083322	18083322+Q1b1a2a1b,CTS8561	Q1b1a2a1b,CTS8561	A>G	A	G
NC_000024.9:g.18808552C>T	18808552	18808552+Q1b1a2a1b,CTS9298	Q1b1a2a1b,CTS9298	C>T	C	T
NC_000024.9:g.7616820G>A	7616820	7616820+Q1b1a2a1b,Y2810	Q1b1a2a1b,Y2810	G>A	G	A
NC_000024.9:g.7696044T>A	7696044	7696044+Q1b1a2a1b,Y2811	Q1b1a2a1b,Y2811	T>A	T	A
NC_000024.9:g.7948057A>T	7948057	7948057+Q1b1a2a1b,Y2812	Q1b1a2a1b,Y2812	A>T	A	T
NC_000024.9:g.8429552G>T	8429552	8429552+Q1b1a2a1b,Y2813	Q1b1a2a1b,Y2813	G>T	G	T
NC_000024.9:g.8471309C>T	8471309	8471309+Q1b1a2a1b,Y2814	Q1b1a2a1b,Y2814	C>T	C	T
NC_000024.9:g.8521207T>C	8521207	8521207+Q1b1a2a1b,Y2815	Q1b1a2a1b,Y2815	T>C	T	C
NC_000024.9:g.9070037A>T	9070037	9070037+Q1b1a2a1b,Y2817	Q1b1a2a1b,Y2817	A>T	A	T
NC_000024.9:g.9760279G>A	9760279	9760279+Q1b1a2a1b,Y2818	Q1b1a2a1b,Y2818	G>A	G	A
NC_000024.9:g.19104439G>A	19104439	19104439+Q1b1a2a1b,Y2821	Q1b1a2a1b,Y2821	G>A	G	A
NC_000024.9:g.19316878T>C	19316878	19316878+Q1b1a2a1b,Y2822	Q1b1a2a1b,Y2822	T>C	T	C
NC_000024.9:g.21781511T>C	21781511	21781511+Q1b1a2a1b,Y2823	Q1b1a2a1b,Y2823	T>C	T	C
NC_000024.9:g.21825985C>T	21825985	21825985+Q1b1a2a1b,Y2824	Q1b1a2a1b,Y2824	C>T	C	T
NC_000024.9:g.21833372G>C	21833372	21833372+Q1b1a2a1b,Y2825	Q1b1a2a1b,Y2825	G>C	G	C
NC_000024.9:g.21978397T>G	21978397	21978397+Q1b1a2a1b,Y2826	Q1b1a2a1b,Y2826	T>G	T	G
NC_000024.9:g.23452026A>T	23452026	23452026+Q1b1a2a1b,Y2828	Q1b1a2a1b,Y2828	A>T	A	T
NC_000024.9:g.23966083G>A	23966083	23966083+Q1b1a2a1b,Y2830	Q1b1a2a1b,Y2830	G>A	G	A
NC_000024.9:g.7078739G>T	7078739	7078739+Q1b1a2a1b,Z19271	Q1b1a2a1b,Z19271	G>T	G	T
NC_000024.9:g.8227309T>C	8227309	8227309+Q1b1a2a1b,Z19273	Q1b1a2a1b,Z19273	T>C	T	C
NC_000024.9:g.20833715C>T	20833715	20833715+Q1b1a2a1b,Z19285	Q1b1a2a1b,Z19285	C>T	C	T
NC_000024.9:g.22516553A>G	22516553	22516553+Q1b1a2a1b,Z19290	Q1b1a2a1b,Z19290	A>G	A	G
NC_000024.9:g.8715235G>T	8715235	8715235+Q1b1a2a1b,Z782	Q1b1a2a1b,Z782	G>T	G	T
NC_000024.9:g.14864475C>T	14864475	14864475+Q1b1a2a1b,Z784	Q1b1a2a1b,Z784	C>T	C	T
NC_000024.9:g.23084064G>A	23084064	23084064+Q1b1a2a1b~,CTS11365	Q1b1a2a1b~,CTS11365	G>A	G	A
NC_000024.9:g.14303794C>T	14303794	14303794+Q1b1a2a1b~,CTS2433	Q1b1a2a1b~,CTS2433	C>T	C	T

NC_000024.9:g.14512533G>A	14512533	14512533+Q1b1a2a1b~,CTS2837	Q1b1a2a1b~,CTS2837	G>A	G	A
NC_000024.9:g.15890680C>A	15890680	15890680+Q1b1a2a1b~,CTS4894	Q1b1a2a1b~,CTS4894	C>A	C	A
NC_000024.9:g.17428822C>A	17428822	17428822+Q1b1a2a1b~,CTS7338	Q1b1a2a1b~,CTS7338	C>A	C	A
NC_000024.9:g.18990978G>A	18990978	18990978+Q1b1a2a1b~,CTS9614	Q1b1a2a1b~,CTS9614	G>A	G	A
NC_000024.9:g.14620204T>C	14620204	14620204+Q1b1a2a1b~,Y2819	Q1b1a2a1b~,Y2819	T>C	T	C
NC_000024.9:g.9989983G>T	9989983	9989983+Q1b1a2a1b~,Z19275	Q1b1a2a1b~,Z19275	G>T	G	T
NC_000024.9:g.16005445C>A	16005445	16005445+Q1b1a2a1b~,Z19280	Q1b1a2a1b~,Z19280	C>A	C	A
NC_000024.9:g.18412166G>A	18412166	18412166+Q1b1a2a1b~,Z19283	Q1b1a2a1b~,Z19283	G>A	G	A
NC_000024.9:g.22077123C>T	22077123	22077123+Q1b1a2a1b~,Z19287	Q1b1a2a1b~,Z19287	C>T	C	T
NC_000024.9:g.14172093G>A	14172093	14172093+Q1b1a2a1b1~,BZ1032	Q1b1a2a1b1~,BZ1032	G>A	G	A
NC_000024.9:g.23567486G>A	23567486	23567486+Q1b1a2a1b1~,BZ1033	Q1b1a2a1b1~,BZ1033	G>A	G	A
NC_000024.9:g.19502238T>C	19502238	19502238+Q1b1a2a1b1~,CTS10510	Q1b1a2a1b1~,CTS10510	T>C	T	C
NC_000024.9:g.14448152G>A	14448152	14448152+Q1b1a2a1b1~,CTS2726	Q1b1a2a1b1~,CTS2726	G>A	G	A
NC_000024.9:g.14856567C>A	14856567	14856567+Q1b1a2a1b1~,CTS3347	Q1b1a2a1b1~,CTS3347	C>A	C	A
NC_000024.9:g.15050465T>C	15050465	15050465+Q1b1a2a1b1~,CTS3591	Q1b1a2a1b1~,CTS3591	T>C	T	C
NC_000024.9:g.15825740A>T	15825740	15825740+Q1b1a2a1b1~,CTS4796	Q1b1a2a1b1~,CTS4796	A>T	A	T
NC_000024.9:g.18081554G>A	18081554	18081554+Q1b1a2a1b1~,CTS8553	Q1b1a2a1b1~,CTS8553	G>A	G	A
NC_000024.9:g.18396648A>T	18396648	18396648+Q1b1a2a1b1~,Y26465	Q1b1a2a1b1~,Y26465	A>T	A	T
NC_000024.9:g.2696130T>C	2696130	2696130+Q1b1a2a2,YP910	Q1b1a2a2,YP910	T>C	T	C
NC_000024.9:g.24516576T>C	24516576	24516576+Q1b1a2a2a3~,YP1111	Q1b1a2a2a3~,YP1111	T>C	T	C
NC_000024.9:g.23374754C>A	23374754	23374754+Q1b1a2a3~,YP937	Q1b1a2a3~,YP937	C>A	C	A
NC_000024.9:g.6866305A>G	6866305	6866305+Q1b1a2a3a~,FGC12203	Q1b1a2a3a~,FGC12203	A>G	A	G
NC_000024.9:g.15486346T>C	15486346	15486346+Q1b1a2a3a~,FGC12205	Q1b1a2a3a~,FGC12205	T>C	T	C
NC_000024.9:g.14009693A>T	14009693	14009693+Q1b1a2a3a~,FGC12226	Q1b1a2a3a~,FGC12226	A>T	A	T
NC_000024.9:g.16817218A>G	16817218	16817218+Q1b1a2a3a~,FGC12244	Q1b1a2a3a~,FGC12244	A>G	A	G
NC_000024.9:g.21181975G>C	21181975	21181975+Q1b1a2a3a~,FGC12264	Q1b1a2a3a~,FGC12264	G>C	G	C
NC_000024.9:g.8245734T>G	8245734	8245734+Q1b1a2a3a~,YP923	Q1b1a2a3a~,YP923	T>G	T	G
NC_000024.9:g.7391481T>C	7391481	7391481+Q1b1a2a3a~,YP926	Q1b1a2a3a~,YP926	T>C	T	C
NC_000024.9:g.7782768C>T	7782768	7782768+Q1b1a2a3a1~,FGC12208	Q1b1a2a3a1~,FGC12208	C>T	C	T
NC_000024.9:g.18945943T>A	18945943	18945943+Q1b1a2a3a1~,FGC12256	Q1b1a2a3a1~,FGC12256	T>A	T	A
NC_000024.9:g.18992317G>A	18992317	18992317+Q1b1a2a3a1~,FGC12258	Q1b1a2a3a1~,FGC12258	G>A	G	A
NC_000024.9:g.19076704A>T	19076704	19076704+Q1b1a2a3a1~,FGC12259	Q1b1a2a3a1~,FGC12259	A>T	A	T
NC_000024.9:g.23498649T>C	23498649	23498649+Q1b1a2a3a1~,FGC12277	Q1b1a2a3a1~,FGC12277	T>C	T	C
NC_000024.9:g.23539191A>G	23539191	23539191+Q1b1a2a3a1~,FGC12278	Q1b1a2a3a1~,FGC12278	A>G	A	G
NC_000024.9:g.7805128G>A	7805128	7805128+Q1b1a2a3a1~,YP922	Q1b1a2a3a1~,YP922	G>A	G	A
NC_000024.9:g.16984394G>C	16984394	16984394+Q1b1a2a3a1~,YP928	Q1b1a2a3a1~,YP928	G>C	G	C
NC_000024.9:g.18704719A>G	18704719	18704719+Q1b1a2a3a1~,YP930	Q1b1a2a3a1~,YP930	A>G	A	G
NC_000024.9:g.18944706G>C	18944706	18944706+Q1b1a2a3a1~,YP931	Q1b1a2a3a1~,YP931	G>C	G	C
NC_000024.9:g.21665814C>A	21665814	21665814+Q1b1a2a3a1~,YP936	Q1b1a2a3a1~,YP936	C>A	C	A
NC_000024.9:g.2808817A>T	2808817	2808817+Q1b1a2a3a1a~,BZ1670	Q1b1a2a3a1a~,BZ1670	A>T	A	T
NC_000024.9:g.8180048G>A	8180048	8180048+Q1b1a2a3a1a~,BZ1679	Q1b1a2a3a1a~,BZ1679	G>A	G	A
NC_000024.9:g.14603936T>C	14603936	14603936+Q1b1a2a3a1a~,BZ1688	Q1b1a2a3a1a~,BZ1688	T>C	T	C
NC_000024.9:g.15455626C>T	15455626	15455626+Q1b1a2a3a1a~,BZ1691	Q1b1a2a3a1a~,BZ1691	C>T	C	T
NC_000024.9:g.17356531G>C	17356531	17356531+Q1b1a2a3a1a~,BZ1697	Q1b1a2a3a1a~,BZ1697	G>C	G	C
NC_000024.9:g.21465856C>T	21465856	21465856+Q1b1a2a3a1a~,BZ1701	Q1b1a2a3a1a~,BZ1701	C>T	C	T
NC_000024.9:g.23492436C>T	23492436	23492436+Q1b1a2a3a1a~,BZ1706	Q1b1a2a3a1a~,BZ1706	C>T	C	T
NC_000024.9:g.23804949C>T	23804949	23804949+Q1b1a2a3a1a~,BZ1708	Q1b1a2a3a1a~,BZ1708	C>T	C	T
NC_000024.9:g.24517749C>A	24517749	24517749+Q1b1a2a3a1a~,BZ1710	Q1b1a2a3a1a~,BZ1710	C>A	C	A
NC_000024.9:g.8502476G>A	8502476	8502476+Q1b1a2a3a1a~,FGC12198	Q1b1a2a3a1a~,FGC12198	G>A	G	A
NC_000024.9:g.7871263G>A	7871263	7871263+Q1b1a2a3a1a~,FGC12210	Q1b1a2a3a1a~,FGC12210	G>A	G	A
NC_000024.9:g.8264064G>A	8264064	8264064+Q1b1a2a3a1a~,FGC12213	Q1b1a2a3a1a~,FGC12213	G>A	G	A
NC_000024.9:g.13667921G>A	13667921	13667921+Q1b1a2a3a1a~,FGC12224	Q1b1a2a3a1a~,FGC12224	G>A	G	A
NC_000024.9:g.14376801T>C	14376801	14376801+Q1b1a2a3a1a~,FGC12229	Q1b1a2a3a1a~,FGC12229	T>C	T	C
NC_000024.9:g.14458625T>C	14458625	14458625+Q1b1a2a3a1a~,FGC12230	Q1b1a2a3a1a~,FGC12230	T>C	T	C
NC_000024.9:g.15112667G>A	15112667	15112667+Q1b1a2a3a1a~,FGC12233	Q1b1a2a3a1a~,FGC12233	G>A	G	A
NC_000024.9:g.15588249T>A	15588249	15588249+Q1b1a2a3a1a~,FGC12237	Q1b1a2a3a1a~,FGC12237	T>A	T	A
NC_000024.9:g.16615806G>C	16615806	16615806+Q1b1a2a3a1a~,FGC12242	Q1b1a2a3a1a~,FGC12242	G>C	G	C
NC_000024.9:g.16982535G>A	16982535	16982535+Q1b1a2a3a1a~,FGC12246	Q1b1a2a3a1a~,FGC12246	G>A	G	A
NC_000024.9:g.17555536C>A	17555536	17555536+Q1b1a2a3a1a~,FGC12249	Q1b1a2a3a1a~,FGC12249	C>A	C	A
NC_000024.9:g.17590973A>G	17590973	17590973+Q1b1a2a3a1a~,FGC12250	Q1b1a2a3a1a~,FGC12250	A>G	A	G
NC_000024.9:g.17681023G>T	17681023	17681023+Q1b1a2a3a1a~,FGC12251	Q1b1a2a3a1a~,FGC12251	G>T	G	T
NC_000024.9:g.18839042T>C	18839042	18839042+Q1b1a2a3a1a~,FGC12254	Q1b1a2a3a1a~,FGC12254	T>C	T	C
NC_000024.9:g.18982672G>A	18982672	18982672+Q1b1a2a3a1a~,FGC12257	Q1b1a2a3a1a~,FGC12257	G>A	G	A
NC_000024.9:g.19148839C>A	19148839	19148839+Q1b1a2a3a1a~,FGC12260	Q1b1a2a3a1a~,FGC12260	C>A	C	A
NC_000024.9:g.19231205A>G	19231205	19231205+Q1b1a2a3a1a~,FGC12262	Q1b1a2a3a1a~,FGC12262	A>G	A	G
NC_000024.9:g.21092876C>A	21092876	21092876+Q1b1a2a3a1a~,FGC12263	Q1b1a2a3a1a~,FGC12263	C>A	C	A
NC_000024.9:g.21946048T>C	21946048	21946048+Q1b1a2a3a1a~,FGC12266	Q1b1a2a3a1a~,FGC12266	T>C	T	C
NC_000024.9:g.23160843G>T	23160843	23160843+Q1b1a2a3a1a~,FGC12273	Q1b1a2a3a1a~,FGC12273	G>T	G	T
NC_000024.9:g.22048297T>A	22048297	22048297+Q1b2~,B28	Q1b2~,B28	T>A	T	A
NC_000024.9:g.23157802G>T	23157802	23157802+Q1b2~,F5389	Q1b2~,F5389	G>T	G	T
NC_000024.9:g.22170858G>A	22170858	22170858+Q1b2~,FGC6802	Q1b2~,FGC6802	G>A	G	A
NC_000024.9:g.7974296A>G	7974296	7974296+Q1b2~,FGC6804	Q1b2~,FGC6804	A>G	A	G
NC_000024.9:g.17719324G>A	17719324	17719324+Q1b2~,Y2659	Q1b2~,Y2659	G>A	G	A
NC_000024.9:g.18986473G>A	18986473	18986473+Q1b2~,Y2708	Q1b2~,Y2708	G>A	G	A
NC_000024.9:g.6766774C>T	6766774	6766774+Q1b2a,F4674	Q1b2a,F4674	C>T	C	T
NC_000024.9:g.8617180C>T	8617180	8617180+Q1b2a,F4781	Q1b2a,F4781	C>T	C	T
NC_000024.9:g.15757656A>T	15757656	15757656+Q1b2a,F5015	Q1b2a,F5015	A>T	A	T
NC_000024.9:g.17677818C>T	17677818	17677818+Q1b2a,F5107	Q1b2a,F5107	C>T	C	T
NC_000024.9:g.23979427G>A	23979427	23979427+Q1b2a,F5416	Q1b2a,F5416	G>A	G	A
NC_000024.9:g.24436516T>C	24436516	24436516+Q1b2a,Y4801	Q1b2a,Y4801	T>C	T	C
NC_000024.9:g.15326466G>T	15326466	15326466+Q1b2a,Y6777	Q1b2a,Y6777	G>T	G	T
NC_000024.9:g.22814546C>T	22814546	22814546+Q1b2a,Y6785	Q1b2a,Y6785	C>T	C	T
NC_000024.9:g.23768342C>T	23768342	23768342+Q1b2a,Y6787	Q1b2a,Y6787	C>T	C	T
NC_000024.9:g.8435729C>T	8435729	8435729+Q1b2a,Z19233	Q1b2a,Z19233	C>T	C	T
NC_000024.9:g.15968545G>A	15968545	15968545+Q1b2a,Z19239	Q1b2a,Z19239	G>A	G	A
NC_000024.9:g.16041505C>T	16041505	16041505+Q1b2a,Z19240	Q1b2a,Z19240	C>T	C	T
NC_000024.9:g.17751098G>T	17751098	17751098+Q1b2a,Z19242	Q1b2a,Z19242	G>T	G	T
NC_000024.9:g.18726873G>A	18726873	18726873+Q1b2a,Z19243	Q1b2a,Z19243	G>A	G	A
NC_000024.9:g.19384343G>A	19384343	19384343+Q1b2a,Z19245	Q1b2a,Z19245	G>A	G	A
NC_000024.9:g.20815820G>T	20815820	20815820+Q1b2a,Z19247	Q1b2a,Z19247	G>T	G	T
NC_000024.9:g.21486149G>A	21486149	21486149+Q1b2a,Z19249	Q1b2a,Z19249	G>A	G	A
NC_000024.9:g.22861464C>T	22861464	22861464+Q1b2a,Z19255	Q1b2a,Z19255	C>T	C	T
NC_000024.9:g.9777360A>T	9777360	9777360+Q1b2b1a2e2a~,YP4799	Q1b2b1a2e2a~,YP4799	A>T	A	T
NC_000024.9:g.8025349G>T	8025349	8025349+Q2,F1082	Q2,F1082	G>T	G	T
NC_000024.9:g.8219098A>G	8219098	8219098+Q2,F1126	Q2,F1126	A>G	A	G
NC_000024.9:g.8394560C>T	8394560	8394560+Q2,F1169	Q2,F1169	C>T	C	T
NC_000024.9:g.8440399T>C	8440399	8440399+Q2,F1205	Q2,F1205	T>C	T	C
NC_000024.9:g.8530564C>A	8530564	8530564+Q2,F1280	Q2,F1280	C>A	C	A
NC_000024.9:g.8599321A>T	8599321	8599321+Q2,F1337	Q2,F1337	A>T	A	T
NC_000024.9:g.14966714C>G	14966714	14966714+Q2,F1875	Q2,F1875	C>G	C	G
NC_000024.9:g.7642841G>C	7642841	7642841+Q2,Y1043	Q2,Y1043	G>C	G	C
NC_000024.9:g.7751037A>G	7751037	7751037+Q2,Y1044	Q2,Y1044	A>G	A	G
NC_000024.9:g.7791804C>T	7791804	7791804+Q2,Y1047	Q2,Y1047	C>T	C	T
NC_000024.9:g.7840832T>C	7840832	7840832+Q2,Y1049	Q2,Y1049	T>C	T	C

NC_000024.9:g.7881856A>C	7881856	7881856+Q2,Y1050	Q2,Y1050	A>C	A	C
NC_000024.9:g.8332304C>A	8332304	8332304+Q2,Y1051	Q2,Y1051	C>A	C	A
NC_000024.9:g.8343767G>A	8343767	8343767+Q2,Y1052	Q2,Y1052	G>A	G	A
NC_000024.9:g.8776629T>G	8776629	8776629+Q2,Y1053	Q2,Y1053	T>G	T	G
NC_000024.9:g.19124330G>A	19124330	19124330+Q2,Y1101	Q2,Y1101	G>A	G	A
NC_000024.9:g.7751169C>T	7751169	7751169+Q2,Z10235	Q2,Z10235	C>T	C	T
NC_000024.9:g.8451013G>A	8451013	8451013+Q2a,F1213	Q2a,F1213	G>A	G	A
NC_000024.9:g.8611330T>A	8611330	8611330+Q2a,F1349	Q2a,F1349	T>A	T	A
NC_000024.9:g.8821295A>G	8821295	8821295+Q2a,FGC1814	Q2a,FGC1814	A>G	A	G
NC_000024.9:g.7652630G>A	7652630	7652630+Q2a,Y2047	Q2a,Y2047	G>A	G	A
NC_000024.9:g.8681004T>C	8681004	8681004+Q2a1,FGC1812	Q2a1,FGC1812	T>C	T	C
NC_000024.9:g.7952263C>T	7952263	7952263+Q2a1,Y2050	Q2a1,Y2050	C>T	C	T
NC_000024.9:g.8682184C>T	8682184	8682184+Q2a1,Y2053	Q2a1,Y2053	C>T	C	T
NC_000024.9:g.8705261C>A	8705261	8705261+Q2a1b,BZ386	Q2a1b,BZ386	C>A	C	A
NC_000024.9:g.15948632G>C	15948632	15948632+Q2a1b,BZ391	Q2a1b,BZ391	G>C	G	C
NC_000024.9:g.21343902G>A	21343902	21343902+rs1000165088	rs1000165088	G>A	G	A
NC_000024.9:g.28807336G>T	28807336	28807336+rs1000541638	rs1000541638	G>T	G	T
NC_000024.9:g.15327498T>C	15327498	15327498+rs1001870542	rs1001870542	T>C	T	C
NC_000024.9:g.16518293C>T	16518293	16518293+rs1003435123	rs1003435123	C>T	C	T
NC_000024.9:g.16659460G>A	16659460	16659460+rs1003644999	rs1003644999	G>A	G	A
NC_000024.9:g.2800143G>A	2800143	2800143+rs1007058453	rs1007058453	G>A	G	A
NC_000024.9:g.18865867G>A	18865867	18865867+rs1007141656	rs1007141656	G>A	G	A
NC_000024.9:g.21042051G>A	21042051	21042051+rs1007638889	rs1007638889	G>A	G	A
NC_000024.9:g.15722776G>A	15722776	15722776+rs1010292714	rs1010292714	G>A	G	A
NC_000024.9:g.14699558G>T	14699558	14699558+rs1013308194	rs1013308194	G>T	G	T
NC_000024.9:g.23237966C>A	23237966	23237966+rs1014398026	rs1014398026	C>A	C	A
NC_000024.9:g.22521507G>A	22521507	22521507+rs1015620533	rs1015620533	G>A	G	A
NC_000024.9:g.22563418C>T	22563418	22563418+rs1016141730	rs1016141730	C>T	C	T
NC_000024.9:g.18909494A>G	18909494	18909494+rs1016656449	rs1016656449	A>G	A	G
NC_000024.9:g.18723545T>C	18723545	18723545+rs1019089540	rs1019089540	T>C	T	C
NC_000024.9:g.21247423G>A	21247423	21247423+rs1019630879	rs1019630879	G>A	G	A
NC_000024.9:g.16981963C>T	16981963	16981963+rs1021669312	rs1021669312	C>T	C	T
NC_000024.9:g.21558121G>A	21558121	21558121+rs1024043871	rs1024043871	G>A	G	A
NC_000024.9:g.15647440G>T	15647440	15647440+rs1025909741	rs1025909741	G>T	G	T
NC_000024.9:g.18943473T>A	18943473	18943473+rs1027272333	rs1027272333	T>A	T	A
NC_000024.9:g.28786007C>A	28786007	28786007+rs1029978808	rs1029978808	C>A	C	A
NC_000024.9:g.18796405G>A	18796405	18796405+rs1032756430	rs1032756430	G>A	G	A
NC_000024.9:g.16634553A>G	16634553	16634553+rs1035792302	rs1035792302	A>G	A	G
NC_000024.9:g.19119763C>T	19119763	19119763+rs1036910919	rs1036910919	C>T	C	T
NC_000024.9:g.17270902C>G	17270902	17270902+rs1038073034	rs1038073034	C>G	C	G
NC_000024.9:g.15810356A>G	15810356	15810356+rs1040762501	rs1040762501	A>G	A	G
NC_000024.9:g.19249004G>A	19249004	19249004+rs1041098506	rs1041098506	G>A	G	A
NC_000024.9:g.18946275A>T	18946275	18946275+rs1041832476	rs1041832476	A>T	A	T
NC_000024.9:g.7058473G>T	7058473	7058473+rs1041942756	rs1041942756	G>T	G	T
NC_000024.9:g.58997988C>T	58997988	58997988+rs10436828	rs10436828	C>T	C	T
NC_000024.9:g.17119166A>G	17119166	17119166+rs1044704396	rs1044704396	A>G	A	G
NC_000024.9:g.58979740C>T	58979740	58979740+rs10449152	rs10449152	C>T	C	T
NC_000024.9:g.58979741A>G	58979741	58979741+rs10449153	rs10449153	A>G	A	G
NC_000024.9:g.16989718C>T	16989718	16989718+rs1045859884	rs1045859884	C>T	C	T
NC_000024.9:g.21544679C>T	21544679	21544679+rs1046487833	rs1046487833	C>T	C	T
NC_000024.9:g.16484750G>T	16484750	16484750+rs1048307851	rs1048307851	G>T	G	T
NC_000024.9:g.18176034G>A	18176034	18176034+rs1050541515	rs1050541515	G>A	G	A
NC_000024.9:g.21408027A>G	21408027	21408027+rs1052321859	rs1052321859	A>G	A	G
NC_000024.9:g.21822167C>T	21822167	21822167+rs1052588385	rs1052588385	C>T	C	T
NC_000024.9:g.22051930G>T	22051930	22051930+rs1054376541	rs1054376541	G>T	G	T
NC_000024.9:g.22852890T>C	22852890	22852890+rs1055333098	rs1055333098	T>C	T	C
NC_000024.9:g.21186828T>C	21186828	21186828+rs1056956667	rs1056956667	T>C	T	C
NC_000024.9:g.13448162T>C	13448162	13448162+rs111213763	rs111213763	T>C	T	C
NC_000024.9:g.13448183T>A	13448183	13448183+rs111213765	rs111213765	T>A	T	A
NC_000024.9:g.58976256A>G	58976256	58976256+rs111214018	rs111214018	A>G	A	G
NC_000024.9:g.58978549A>G	58978549	58978549+rs111250623	rs111250623	A>G	A	G
NC_000024.9:g.28791783A>C	28791783	28791783+rs111483144	rs111483144	A>C	A	C
NC_000024.9:g.13447852A>G	13447852	13447852+rs111628973	rs111628973	A>G	A	G
NC_000024.9:g.28812980T>A	28812980	28812980+rs111828982	rs111828982	T>A	T	A
NC_000024.9:g.13429757G>C	13429757	13429757+rs111931855	rs111931855	G>C	G	C
NC_000024.9:g.13903202A>G	13903202	13903202+rs112012054	rs112012054	A>G	A	G
NC_000024.9:g.19071385T>C	19071385	19071385+rs112104391	rs112104391	T>C	T	C
NC_000024.9:g.13427873T>G	13427873	13427873+rs112187769	rs112187769	T>G	T	G
NC_000024.9:g.28586722A>G	28586722	28586722+rs112364132	rs112364132	A>G	A	G
NC_000024.9:g.14490393A>G	14490393	14490393+rs112487081	rs112487081	A>G	A	G
NC_000024.9:g.28814844T>G	28814844	28814844+rs112545479	rs112545479	T>G	T	G
NC_000024.9:g.13449142A>C	13449142	13449142+rs112575652	rs112575652	A>C	A	C
NC_000024.9:g.28809882A>G	28809882	28809882+rs112637370	rs112637370	A>G	A	G
NC_000024.9:g.28789011T>G	28789011	28789011+rs112695985	rs112695985	T>G	T	G
NC_000024.9:g.13446654T>C	13446654	13446654+rs112760143	rs112760143	T>C	T	C
NC_000024.9:g.28810212G>T	28810212	28810212+rs112893987	rs112893987	G>T	G	T
NC_000024.9:g.28583736C>A	28583736	28583736+rs112941636	rs112941636	C>A	C	A
NC_000024.9:g.58979654A>T	58979654	58979654+rs112959580	rs112959580	A>T	A	T
NC_000024.9:g.16441971C>A	16441971	16441971+rs113001980	rs113001980	C>A	C	A
NC_000024.9:g.28776391G>A	28776391	28776391+rs113056285	rs113056285	G>A	G	A
NC_000024.9:g.28818284A>G	28818284	28818284+rs113075516	rs113075516	A>G	A	G
NC_000024.9:g.13450069T>C	13450069	13450069+rs113150365	rs113150365	T>C	T	C
NC_000024.9:g.28809845T>G	28809845	28809845+rs113195250	rs113195250	T>G	T	G
NC_000024.9:g.28807349A>T	28807349	28807349+rs113228027	rs113228027	A>T	A	T
NC_000024.9:g.28818295C>G	28818295	28818295+rs113314966	rs113314966	C>G	C	G
NC_000024.9:g.13448853A>C	13448853	13448853+rs113537379	rs113537379	A>C	A	C
NC_000024.9:g.28809864T>A	28809864	28809864+rs113552652	rs113552652	T>A	T	A
NC_000024.9:g.28582566C>G	28582566	28582566+rs113624297	rs113624297	C>G	C	G
NC_000024.9:g.9850614G>C	9850614	9850614+rs113733758	rs113733758	G>C	G	C
NC_000024.9:g.28810632A>G	28810632	28810632+rs113739300	rs113739300	A>G	A	G
NC_000024.9:g.2686555A>G	2686555	2686555+rs113881366	rs113881366	A>G	A	G
NC_000024.9:g.17731389C>T	17731389	17731389+rs113915181	rs113915181	C>T	C	T
NC_000024.9:g.9850573C>T	9850573	9850573+rs1171643949	rs1171643949	C>T	C	T
NC_000024.9:g.28808070G>A	28808070	28808070+rs1179904602	rs1179904602	G>A	G	A
NC_000024.9:g.58969124T>C	58969124	58969124+rs11799074	rs11799074	T>C	T	C
NC_000024.9:g.58969118A>G	58969118	58969118+rs11799270	rs11799270	A>G	A	G
NC_000024.9:g.28808976C>T	28808976	28808976+rs1206528507	rs1206528507	C>T	C	T
NC_000024.9:g.13592861T>C	13592861	13592861+rs1209877986	rs1209877986	T>C	T	C
NC_000024.9:g.28587859G>C	28587859	28587859+rs12116397	rs12116397	G>C	G	C
NC_000024.9:g.28588173C>G	28588173	28588173+rs12116410	rs12116410	C>G	C	G
NC_000024.9:g.7111075C>T	7111075	7111075+rs1214459974	rs1214459974	C>T	C	T
NC_000024.9:g.58979310T>C	58979310	58979310+rs12164445	rs12164445	T>C	T	C
NC_000024.9:g.4357890C>T	4357890	4357890+rs1225253028	rs1225253028	C>T	C	T
NC_000024.9:g.16494398G>T	16494398	16494398+rs1232431	rs1232431	G>T	G	T

NC_000024.9:g.14435779A>G	14435779	14435779+rs1247147948	rs1247147948	A>G	A	G
NC_000024.9:g.21869055C>T	21869055	21869055+rs1248622510	rs1248622510	C>T	C	T
NC_000024.9:g.16441969C>A	16441969	16441969+rs1267231975	rs1267231975	C>A	C	A
NC_000024.9:g.2759959C>T	2759959	2759959+rs1270129	rs1270129	C>T	C	T
NC_000024.9:g.14315955A>G	14315955	14315955+rs1281366472	rs1281366472	A>G	A	G
NC_000024.9:g.13395042C>T	13395042	13395042+rs1283536979	rs1283536979	C>T	C	T
NC_000024.9:g.4726333T>A	4726333	4726333+rs1289801536	rs1289801536	T>A	T	A
NC_000024.9:g.16870364G>A	16870364	16870364+rs1299346724	rs1299346724	G>A	G	A
NC_000024.9:g.9850581C>T	9850581	9850581+rs1310629473	rs1310629473	C>T	C	T
NC_000024.9:g.15226548A>G	15226548	15226548+rs13305177	rs13305177	A>G	A	G
NC_000024.9:g.28472208G>A	28472208	28472208+rs1335151320	rs1335151320	G>A	G	A
NC_000024.9:g.28552901C>T	28552901	28552901+rs1340143866	rs1340143866	C>T	C	T
NC_000024.9:g.13591168G>T	13591168	13591168+rs1341951773	rs1341951773	G>T	G	T
NC_000024.9:g.28470598C>A	28470598	28470598+rs1362567547	rs1362567547	C>A	C	A
NC_000024.9:g.17809221C>T	17809221	17809221+rs1381670889	rs1381670889	C>T	C	T
NC_000024.9:g.28812340G>A	28812340	28812340+rs138532430	rs138532430	G>A	G	A
NC_000024.9:g.28586903T>A	28586903	28586903+rs138749504	rs138749504	T>A	T	A
NC_000024.9:g.14000216C>T	14000216	14000216+rs1389101051	rs1389101051	C>T	C	T
NC_000024.9:g.4502558T>C	4502558	4502558+rs1394106562	rs1394106562	T>C	T	C
NC_000024.9:g.9947215C>G	9947215	9947215+rs139591669	rs139591669	C>G	C	G
NC_000024.9:g.28818407C>A	28818407	28818407+rs140208873	rs140208873	C>A	C	A
NC_000024.9:g.28687807A>G	28687807	28687807+rs1404319449	rs1404319449	A>G	A	G
NC_000024.9:g.9850578A>T	9850578	9850578+rs1411297067	rs1411297067	A>T	A	T
NC_000024.9:g.28809076G>C	28809076	28809076+rs1432487706	rs1432487706	G>C	G	C
NC_000024.9:g.28812759C>A	28812759	28812759+rs144102930	rs144102930	C>A	C	A
NC_000024.9:g.28797832G>T	28797832	28797832+rs144208007	rs144208007	G>T	G	T
NC_000024.9:g.13195624G>A	13195624	13195624+rs1451671785	rs1451671785	G>A	G	A
NC_000024.9:g.28817301C>T	28817301	28817301+rs145400344	rs145400344	C>T	C	T
NC_000024.9:g.28818362A>G	28818362	28818362+rs145802734	rs145802734	A>G	A	G
NC_000024.9:g.2818677A>G	2818677	2818677+rs1467994281	rs1467994281	A>G	A	G
NC_000024.9:g.13593583C>T	13593583	13593583+rs1470735685	rs1470735685	C>T	C	T
NC_000024.9:g.15558499T>C	15558499	15558499+rs147915010	rs147915010	T>C	T	C
NC_000024.9:g.3834391G>T	3834391	3834391+rs1483562125	rs1483562125	G>T	G	T
NC_000024.9:g.19411925A>G	19411925	19411925+rs1569416800	rs1569416800	A>G	A	G
NC_000024.9:g.6734763C>T	6734763	6734763+rs1603021133	rs1603021133	C>T	C	T
NC_000024.9:g.7379268C>T	7379268	7379268+rs1603074991	rs1603074991	C>T	C	T
NC_000024.9:g.7460176T>C	7460176	7460176+rs1603078550	rs1603078550	T>C	T	C
NC_000024.9:g.7460201T>C	7460201	7460201+rs1603078553	rs1603078553	T>C	T	C
NC_000024.9:g.14229664C>T	14229664	14229664+rs1603172164	rs1603172164	C>T	C	T
NC_000024.9:g.14370167C>T	14370167	14370167+rs1603179200	rs1603179200	C>T	C	T
NC_000024.9:g.14530425G>A	14530425	14530425+rs1603185723	rs1603185723	G>A	G	A
NC_000024.9:g.14615247C>T	14615247	14615247+rs1603188547	rs1603188547	C>T	C	T
NC_000024.9:g.14969016G>C	14969016	14969016+rs1603204499	rs1603204499	G>C	G	C
NC_000024.9:g.15071327G>A	15071327	15071327+rs1603209132	rs1603209132	G>A	G	A
NC_000024.9:g.16645476T>G	16645476	16645476+rs1603498988	rs1603498988	T>G	T	G
NC_000024.9:g.17082981T>C	17082981	17082981+rs1603506312	rs1603506312	T>C	T	C
NC_000024.9:g.18581960A>G	18581960	18581960+rs1603521280	rs1603521280	A>G	A	G
NC_000024.9:g.19058867T>A	19058867	19058867+rs1603526104	rs1603526104	T>A	T	A
NC_000024.9:g.19305382C>T	19305382	19305382+rs1603529080	rs1603529080	C>T	C	T
NC_000024.9:g.21336392G>A	21336392	21336392+rs1603539811	rs1603539811	G>A	G	A
NC_000024.9:g.21838922G>A	21838922	21838922+rs1603545315	rs1603545315	G>A	G	A
NC_000024.9:g.22293908G>T	22293908	22293908+rs1603557500	rs1603557500	G>T	G	T
NC_000024.9:g.7461424C>G	7461424	7461424+rs199498256	rs199498256	C>G	C	G
NC_000024.9:g.13447977A>T	13447977	13447977+rs199551994	rs199551994	A>T	A	T
NC_000024.9:g.58979661T>A	58979661	58979661+rs199761124	rs199761124	T>A	T	A
NC_000024.9:g.28584105G>A	28584105	28584105+rs199790585	rs199790585	G>A	G	A
NC_000024.9:g.28804131A>G	28804131	28804131+rs200189258	rs200189258	A>G	A	G
NC_000024.9:g.28787566A>T	28787566	28787566+rs200205529	rs200205529	A>T	A	T
NC_000024.9:g.13529496C>T	13529496	13529496+rs200273969	rs200273969	C>T	C	T
NC_000024.9:g.28798952C>G	28798952	28798952+rs200457936	rs200457936	C>G	C	G
NC_000024.9:g.28584134C>T	28584134	28584134+rs200578708	rs200578708	C>T	C	T
NC_000024.9:g.13450084C>T	13450084	13450084+rs200687341	rs200687341	C>T	C	T
NC_000024.9:g.15558495T>C	15558495	15558495+rs200737532	rs200737532	T>C	T	C
NC_000024.9:g.28814983G>T	28814983	28814983+rs200778010	rs200778010	G>T	G	T
NC_000024.9:g.28819013T>G	28819013	28819013+rs200795484	rs200795484	T>G	T	G
NC_000024.9:g.28810898C>T	28810898	28810898+rs200865928	rs200865928	C>T	C	T
NC_000024.9:g.18149590G>C	18149590	18149590+rs200906617	rs200906617	G>C	G	C
NC_000024.9:g.28814703C>T	28814703	28814703+rs201176076	rs201176076	C>T	C	T
NC_000024.9:g.28813244C>G	28813244	28813244+rs201342803	rs201342803	C>G	C	G
NC_000024.9:g.28810833C>T	28810833	28810833+rs201353086	rs201353086	C>T	C	T
NC_000024.9:g.28709252C>A	28709252	28709252+rs201360660	rs201360660	C>A	C	A
NC_000024.9:g.28818413A>T	28818413	28818413+rs201427130	rs201427130	A>T	A	T
NC_000024.9:g.2759947C>G	2759947	2759947+rs201530966	rs201530966	C>G	C	G
NC_000024.9:g.28819163G>A	28819163	28819163+rs201669548	rs201669548	G>A	G	A
NC_000024.9:g.28819016G>T	28819016	28819016+rs201866326	rs201866326	G>T	G	T
NC_000024.9:g.28813259C>T	28813259	28813259+rs201944593	rs201944593	C>T	C	T
NC_000024.9:g.13515161A>C	13515161	13515161+rs201992391	rs201992391	A>C	A	C
NC_000024.9:g.7461449C>G	7461449	7461449+rs202074983	rs202074983	C>G	C	G
NC_000024.9:g.23476015T>G	23476015	23476015+rs202159348	rs202159348	T>G	T	G
NC_000024.9:g.28814941T>G	28814941	28814941+rs202223012	rs202223012	T>G	T	G
NC_000024.9:g.9952290T>A	9952290	9952290+rs2311640	rs2311640	T>A	T	A
NC_000024.9:g.58971182A>T	58971182	58971182+rs2453027	rs2453027	A>T	A	T
NC_000024.9:g.58971564T>A	58971564	58971564+rs2527489	rs2527489	T>A	T	A
NC_000024.9:g.58968689T>G	58968689	58968689+rs2527490	rs2527490	T>G	T	G
NC_000024.9:g.17313278T>C	17313278	17313278+rs2566505	rs2566505	T>C	T	C
NC_000024.9:g.58978913A>C	58978913	58978913+rs2641196	rs2641196	A>C	A	C
NC_000024.9:g.58978909G>T	58978909	58978909+rs2641197	rs2641197	G>T	G	T
NC_000024.9:g.58968660G>T	58968660	58968660+rs2641205	rs2641205	G>T	G	T
NC_000024.9:g.58968074G>A	58968074	58968074+rs2641209	rs2641209	G>A	G	A
NC_000024.9:g.58968816T>G	58968816	58968816+rs28374335	rs28374335	T>G	T	G
NC_000024.9:g.28815765A>T	28815765	28815765+rs2890624	rs2890624	A>T	A	T
NC_000024.9:g.58967742C>T	58967742	58967742+rs2934363	rs2934363	C>T	C	T
NC_000024.9:g.28810859A>G	28810859	28810859+rs34007408	rs34007408	A>G	A	G
NC_000024.9:g.28810848G>T	28810848	28810848+rs34312364	rs34312364	G>T	G	T
NC_000024.9:g.28810788A>T	28810788	28810788+rs36080814	rs36080814	A>T	A	T
NC_000024.9:g.13448141T>A	13448141	13448141+rs367748247	rs367748247	T>A	T	A
NC_000024.9:g.28817079G>A	28817079	28817079+rs367879091	rs367879091	G>A	G	A
NC_000024.9:g.17254423C>T	17254423	17254423+rs368320359	rs368320359	C>T	C	T
NC_000024.9:g.14063200G>A	14063200	14063200+rs370276016	rs370276016	G>A	G	A
NC_000024.9:g.13448145T>C	13448145	13448145+rs370577546	rs370577546	T>C	T	C
NC_000024.9:g.23225335T>C	23225335	23225335+rs370613549	rs370613549	T>C	T	C
NC_000024.9:g.14049534G>T	14049534	14049534+rs370714884	rs370714884	G>T	G	T
NC_000024.9:g.28804982C>A	28804982	28804982+rs371003537	rs371003537	C>A	C	A
NC_000024.9:g.9952707G>A	9952707	9952707+rs371030722	rs371030722	G>A	G	A

NC_000024.9:g.22517413A>C	22517413	22517413+rs371098625	rs371098625	A>C	A	C
NC_000024.9:g.28817109A>G	28817109	28817109+rs371988622	rs371988622	A>G	A	G
NC_000024.9:g.28817225A>G	28817225	28817225+rs372390542	rs372390542	A>G	A	G
NC_000024.9:g.13514971T>C	13514971	13514971+rs372755361	rs372755361	T>C	T	C
NC_000024.9:g.22977543C>A	22977543	22977543+rs373222788	rs373222788	C>A	C	A
NC_000024.9:g.13448910A>C	13448910	13448910+rs373576356	rs373576356	A>C	A	C
NC_000024.9:g.9850574A>G	9850574	9850574+rs374153433	rs374153433	A>G	A	G
NC_000024.9:g.17274963C>T	17274963	17274963+rs374872247	rs374872247	C>T	C	T
NC_000024.9:g.28817222G>A	28817222	28817222+rs375099177	rs375099177	G>A	G	A
NC_000024.9:g.2759928G>T	2759928	2759928+rs375374253	rs375374253	G>T	G	T
NC_000024.9:g.15422969A>C	15422969	15422969+rs376324049	rs376324049	A>C	A	C
NC_000024.9:g.17857184T>C	17857184	17857184+rs376346152	rs376346152	T>C	T	C
NC_000024.9:g.28791793A>G	28791793	28791793+rs376444891	rs376444891	A>G	A	G
NC_000024.9:g.13448873T>C	13448873	13448873+rs377621225	rs377621225	T>C	T	C
NC_000024.9:g.13908941G>A	13908941	13908941+rs3888953	rs3888953	G>A	G	A
NC_000024.9:g.58969854G>A	58969854	58969854+rs3947483	rs3947483	G>A	G	A
NC_000024.9:g.28582863A>G	28582863	28582863+rs4008408	rs4008408	A>G	A	G
NC_000024.9:g.28582865A>G	28582865	28582865+rs4008409	rs4008409	A>G	A	G
NC_000024.9:g.28582921A>G	28582921	28582921+rs4008410	rs4008410	A>G	A	G
NC_000024.9:g.28582932G>A	28582932	28582932+rs4008411	rs4008411	G>A	G	A
NC_000024.9:g.28583050T>C	28583050	28583050+rs4008414	rs4008414	T>C	T	C
NC_000024.9:g.6670461G>T	6670461	6670461+rs4027562	rs4027562	G>T	G	T
NC_000024.9:g.58967719A>G	58967719	58967719+rs4134481	rs4134481	A>G	A	G
NC_000024.9:g.58970429T>C	58970429	58970429+rs4243386	rs4243386	T>C	T	C
NC_000024.9:g.28793797A>G	28793797	28793797+rs4261066	rs4261066	A>G	A	G
NC_000024.9:g.58969146C>T	58969146	58969146+rs4491015	rs4491015	C>T	C	T
NC_000024.9:g.58968888A>G	58968888	58968888+rs4576608	rs4576608	A>G	A	G
NC_000024.9:g.58969145T>G	58969145	58969145+rs4579697	rs4579697	T>G	T	G
NC_000024.9:g.58969133G>A	58969133	58969133+rs4615785	rs4615785	G>A	G	A
NC_000024.9:g.58968939G>A	58968939	58968939+rs4636392	rs4636392	G>A	G	A
NC_000024.9:g.58969655G>A	58969655	58969655+rs4893701	rs4893701	G>A	G	A
NC_000024.9:g.58969644G>C	58969644	58969644+rs4893702	rs4893702	G>C	G	C
NC_000024.9:g.58969409T>A	58969409	58969409+rs4893703	rs4893703	T>A	T	A
NC_000024.9:g.58982471G>A	58982471	58982471+rs4893792	rs4893792	G>A	G	A
NC_000024.9:g.58968724T>C	58968724	58968724+rs4893814	rs4893814	T>C	T	C
NC_000024.9:g.14027829C>T	14027829	14027829+rs528244489	rs528244489	C>T	C	T
NC_000024.9:g.14091157G>A	14091157	14091157+rs530307480	rs530307480	G>A	G	A
NC_000024.9:g.28606530C>A	28606530	28606530+rs531490187	rs531490187	C>A	C	A
NC_000024.9:g.16735462G>T	16735462	16735462+rs534176122	rs534176122	G>T	G	T
NC_000024.9:g.16993300C>T	16993300	16993300+rs536064140	rs536064140	C>T	C	T
NC_000024.9:g.14607881G>A	14607881	14607881+rs537103108	rs537103108	G>A	G	A
NC_000024.9:g.18149598C>G	18149598	18149598+rs541145761	rs541145761	C>G	C	G
NC_000024.9:g.8815309G>A	8815309	8815309+rs543357253	rs543357253	G>A	G	A
NC_000024.9:g.14264865G>T	14264865	14264865+rs546824712	rs546824712	G>T	G	T
NC_000024.9:g.14555761T>C	14555761	14555761+rs549138332	rs549138332	T>C	T	C
NC_000024.9:g.28635927A>G	28635927	28635927+rs549584628	rs549584628	A>G	A	G
NC_000024.9:g.18857588T>G	18857588	18857588+rs550110590	rs550110590	T>G	T	G
NC_000024.9:g.22893304C>T	22893304	22893304+rs551031888	rs551031888	C>T	C	T
NC_000024.9:g.15202609G>A	15202609	15202609+rs553509804	rs553509804	G>A	G	A
NC_000024.9:g.59004273A>G	59004273	59004273+rs56044312	rs56044312	A>G	A	G
NC_000024.9:g.21475018T>C	21475018	21475018+rs561021114	rs561021114	T>C	T	C
NC_000024.9:g.17511829C>T	17511829	17511829+rs564824811	rs564824811	C>T	C	T
NC_000024.9:g.16802006C>T	16802006	16802006+rs567880612	rs567880612	C>T	C	T
NC_000024.9:g.28610750T>C	28610750	28610750+rs570081878	rs570081878	T>C	T	C
NC_000024.9:g.17073647T>A	17073647	17073647+rs573798417	rs573798417	T>A	T	A
NC_000024.9:g.14155419C>A	14155419	14155419+rs573967133	rs573967133	C>A	C	A
NC_000024.9:g.28787578G>C	28787578	28787578+rs57888452	rs57888452	G>C	G	C
NC_000024.9:g.13591495C>T	13591495	13591495+rs62603883	rs62603883	C>T	C	T
NC_000024.9:g.9952345C>T	9952345	9952345+rs62609926	rs62609926	C>T	C	T
NC_000024.9:g.13140446A>T	13140446	13140446+rs62610031	rs62610031	A>T	A	T
NC_000024.9:g.28549568T>G	28549568	28549568+rs62614526	rs62614526	T>G	T	G
NC_000024.9:g.28549652T>C	28549652	28549652+rs62614527	rs62614527	T>C	T	C
NC_000024.9:g.28583137G>A	28583137	28583137+rs62614538	rs62614538	G>A	G	A
NC_000024.9:g.28586775C>T	28586775	28586775+rs62614539	rs62614539	C>T	C	T
NC_000024.9:g.28586776A>G	28586776	28586776+rs62614541	rs62614541	A>G	A	G
NC_000024.9:g.28586782G>A	28586782	28586782+rs62614542	rs62614542	G>A	G	A
NC_000024.9:g.28587232T>C	28587232	28587232+rs62614543	rs62614543	T>C	T	C
NC_000024.9:g.28797378A>G	28797378	28797378+rs62614559	rs62614559	A>G	A	G
NC_000024.9:g.28805109T>G	28805109	28805109+rs62615402	rs62615402	T>G	T	G
NC_000024.9:g.28805126T>G	28805126	28805126+rs62615403	rs62615403	T>G	T	G
NC_000024.9:g.28805871C>G	28805871	28805871+rs62615406	rs62615406	C>G	C	G
NC_000024.9:g.28790604C>T	28790604	28790604+rs62642015	rs62642015	C>T	C	T
NC_000024.9:g.28780823T>A	28780823	28780823+rs67789210	rs67789210	T>A	T	A
NC_000024.9:g.28816878C>G	28816878	28816878+rs7067451	rs7067451	C>G	C	G
NC_000024.9:g.28817636G>A	28817636	28817636+rs7067479	rs7067479	G>A	G	A
NC_000024.9:g.28813372T>G	28813372	28813372+rs71265351	rs71265351	T>G	T	G
NC_000024.9:g.13909007A>G	13909007	13909007+rs72609608	rs72609608	A>G	A	G
NC_000024.9:g.28587511C>T	28587511	28587511+rs72615078	rs72615078	C>T	C	T
NC_000024.9:g.28776400T>C	28776400	28776400+rs72615086	rs72615086	T>C	T	C
NC_000024.9:g.9850653T>C	9850653	9850653+rs72618773	rs72618773	T>C	T	C
NC_000024.9:g.9952642T>C	9952642	9952642+rs72618787	rs72618787	T>C	T	C
NC_000024.9:g.10014445T>G	10014445	10014445+rs72619843	rs72619843	T>G	T	G
NC_000024.9:g.2829444A>G	2829444	2829444+rs72625387	rs72625387	A>G	A	G
NC_000024.9:g.10014512A>G	10014512	10014512+rs73616552	rs73616552	A>G	A	G
NC_000024.9:g.10014517A>G	10014517	10014517+rs73616553	rs73616553	A>G	A	G
NC_000024.9:g.10022039A>G	10022039	10022039+rs73616577	rs73616577	A>G	A	G
NC_000024.9:g.15226313C>T	15226313	15226313+rs73621770	rs73621770	C>T	C	T
NC_000024.9:g.28587495C>T	28587495	28587495+rs73628936	rs73628936	C>T	C	T
NC_000024.9:g.28803167A>G	28803167	28803167+rs73628950	rs73628950	A>G	A	G
NC_000024.9:g.13909140T>C	13909140	13909140+rs74324683	rs74324683	T>C	T	C
NC_000024.9:g.28587483A>C	28587483	28587483+rs74354264	rs74354264	A>C	A	C
NC_000024.9:g.28584081T>C	28584081	28584081+rs74415902	rs74415902	T>C	T	C
NC_000024.9:g.13448358C>T	13448358	13448358+rs74436109	rs74436109	C>T	C	T
NC_000024.9:g.28587559T>G	28587559	28587559+rs74514528	rs74514528	T>G	T	G
NC_000024.9:g.28588244T>C	28588244	28588244+rs74524856	rs74524856	T>C	T	C
NC_000024.9:g.58969452C>T	58969452	58969452+rs74538489	rs74538489	C>T	C	T
NC_000024.9:g.28585775T>C	28585775	28585775+rs74550271	rs74550271	T>C	T	C
NC_000024.9:g.17198497G>A	17198497	17198497+rs745760184	rs745760184	G>A	G	A
NC_000024.9:g.28791131T>A	28791131	28791131+rs74581547	rs74581547	T>A	T	A
NC_000024.9:g.28588446G>T	28588446	28588446+rs74618536	rs74618536	G>T	G	T
NC_000024.9:g.18149594C>G	18149594	18149594+rs746330388	rs746330388	C>G	C	G
NC_000024.9:g.7110511T>G	7110511	7110511+rs746772590	rs746772590	T>G	T	G
NC_000024.9:g.28587646T>A	28587646	28587646+rs74678927	rs74678927	T>A	T	A
NC_000024.9:g.13945850G>C	13945850	13945850+rs746814892	rs746814892	G>C	G	C

NC_000024.9:g.28814985G>A	28814985	28814985+rs74692304	rs74692304	G>A	G	A
NC_000024.9:g.10014459A>C	10014459	10014459+rs74714660	rs74714660	A>C	A	C
NC_000024.9:g.9962532A>C	9962532	9962532+rs74744282	rs74744282	A>C	A	C
NC_000024.9:g.28587531G>A	28587531	28587531+rs74780525	rs74780525	G>A	G	A
NC_000024.9:g.19256677A>G	19256677	19256677+rs747890065	rs747890065	A>G	A	G
NC_000024.9:g.16818679T>A	16818679	16818679+rs74838198	rs74838198	T>A	T	A
NC_000024.9:g.23149974G>C	23149974	23149974+rs748416375	rs748416375	G>C	G	C
NC_000024.9:g.21722779G>A	21722779	21722779+rs748653011	rs748653011	G>A	G	A
NC_000024.9:g.13591428G>A	13591428	13591428+rs748805049	rs748805049	G>A	G	A
NC_000024.9:g.14548195G>A	14548195	14548195+rs748829263	rs748829263	G>A	G	A
NC_000024.9:g.10022132A>G	10022132	10022132+rs74897129	rs74897129	A>G	A	G
NC_000024.9:g.17592474G>T	17592474	17592474+rs749173189	rs749173189	G>T	G	T
NC_000024.9:g.28587666A>C	28587666	28587666+rs74922965	rs74922965	A>C	A	C
NC_000024.9:g.28612484A>C	28612484	28612484+rs749238301	rs749238301	A>C	A	C
NC_000024.9:g.14215333T>C	14215333	14215333+rs749266412	rs749266412	T>C	T	C
NC_000024.9:g.9962451T>C	9962451	9962451+rs74991867	rs74991867	T>C	T	C
NC_000024.9:g.28585427T>G	28585427	28585427+rs75000644	rs75000644	T>G	T	G
NC_000024.9:g.15447901T>C	15447901	15447901+rs750038689	rs750038689	T>C	T	C
NC_000024.9:g.22133789G>A	22133789	22133789+rs750311496	rs750311496	G>A	G	A
NC_000024.9:g.17376464C>T	17376464	17376464+rs750456977	rs750456977	C>T	C	T
NC_000024.9:g.18198205G>A	18198205	18198205+rs750603017	rs750603017	G>A	G	A
NC_000024.9:g.13451048A>C	13451048	13451048+rs75072515	rs75072515	A>C	A	C
NC_000024.9:g.14053382A>G	14053382	14053382+rs750769154	rs750769154	A>G	A	G
NC_000024.9:g.18829387T>G	18829387	18829387+rs750817587	rs750817587	T>G	T	G
NC_000024.9:g.58969445A>G	58969445	58969445+rs75095670	rs75095670	A>G	A	G
NC_000024.9:g.58977492C>T	58977492	58977492+rs75147993	rs75147993	C>T	C	T
NC_000024.9:g.13452320G>A	13452320	13452320+rs75186491	rs75186491	G>A	G	A
NC_000024.9:g.13514988A>G	13514988	13514988+rs75202512	rs75202512	A>G	A	G
NC_000024.9:g.28780767A>C	28780767	28780767+rs752353499	rs752353499	A>C	A	C
NC_000024.9:g.18048053G>C	18048053	18048053+rs752650829	rs752650829	G>C	G	C
NC_000024.9:g.14316705C>T	14316705	14316705+rs752721678	rs752721678	C>T	C	T
NC_000024.9:g.21869679G>A	21869679	21869679+rs752773051	rs752773051	G>A	G	A
NC_000024.9:g.28807932A>G	28807932	28807932+rs75293327	rs75293327	A>G	A	G
NC_000024.9:g.13591259C>T	13591259	13591259+rs752981079	rs752981079	C>T	C	T
NC_000024.9:g.19213850C>A	19213850	19213850+rs753004348	rs753004348	C>A	C	A
NC_000024.9:g.17313283T>C	17313283	17313283+rs753030204	rs753030204	T>C	T	C
NC_000024.9:g.17216457T>G	17216457	17216457+rs753043475	rs753043475	T>G	T	G
NC_000024.9:g.28585456C>A	28585456	28585456+rs75332064	rs75332064	C>A	C	A
NC_000024.9:g.13450120C>T	13450120	13450120+rs753718142	rs753718142	C>T	C	T
NC_000024.9:g.28810391C>G	28810391	28810391+rs75403276	rs75403276	C>G	C	G
NC_000024.9:g.28796280G>A	28796280	28796280+rs75407776	rs75407776	G>A	G	A
NC_000024.9:g.58982110G>C	58982110	58982110+rs75444722	rs75444722	G>C	G	C
NC_000024.9:g.17560651G>A	17560651	17560651+rs754528696	rs754528696	G>A	G	A
NC_000024.9:g.17274971T>C	17274971	17274971+rs754671427	rs754671427	T>C	T	C
NC_000024.9:g.28587818T>C	28587818	28587818+rs75473243	rs75473243	T>C	T	C
NC_000024.9:g.28709251C>T	28709251	28709251+rs754794741	rs754794741	C>T	C	T
NC_000024.9:g.28583948G>C	28583948	28583948+rs75490000	rs75490000	G>C	G	C
NC_000024.9:g.18064892A>G	18064892	18064892+rs755243881	rs755243881	A>G	A	G
NC_000024.9:g.28583212T>C	28583212	28583212+rs75549417	rs75549417	T>C	T	C
NC_000024.9:g.28803170A>T	28803170	28803170+rs755531201	rs755531201	A>T	A	T
NC_000024.9:g.28709275G>C	28709275	28709275+rs755725209	rs755725209	G>C	G	C
NC_000024.9:g.9950612G>T	9950612	9950612+rs75589121	rs75589121	G>T	G	T
NC_000024.9:g.13591385T>C	13591385	13591385+rs755981208	rs755981208	T>C	T	C
NC_000024.9:g.14151331C>T	14151331	14151331+rs756154149	rs756154149	C>T	C	T
NC_000024.9:g.22948010C>T	22948010	22948010+rs756185452	rs756185452	C>T	C	T
NC_000024.9:g.28582676G>A	28582676	28582676+rs75650300	rs75650300	G>A	G	A
NC_000024.9:g.10014477C>T	10014477	10014477+rs75666100	rs75666100	C>T	C	T
NC_000024.9:g.28585528A>G	28585528	28585528+rs75675580	rs75675580	A>G	A	G
NC_000024.9:g.15519876C>T	15519876	15519876+rs756933051	rs756933051	C>T	C	T
NC_000024.9:g.19061611C>A	19061611	19061611+rs756965366	rs756965366	C>A	C	A
NC_000024.9:g.15852079T>G	15852079	15852079+rs757012884	rs757012884	T>G	T	G
NC_000024.9:g.14000346G>A	14000346	14000346+rs757115426	rs757115426	G>A	G	A
NC_000024.9:g.28585563T>G	28585563	28585563+rs75721037	rs75721037	T>G	T	G
NC_000024.9:g.23254109T>C	23254109	23254109+rs757306095	rs757306095	T>C	T	C
NC_000024.9:g.6665404G>T	6665404	6665404+rs757389554	rs757389554	G>T	G	T
NC_000024.9:g.19232131C>T	19232131	19232131+rs757579581	rs757579581	C>T	C	T
NC_000024.9:g.21297892G>A	21297892	21297892+rs757584342	rs757584342	G>A	G	A
NC_000024.9:g.28679389A>T	28679389	28679389+rs757977695	rs757977695	A>T	A	T
NC_000024.9:g.28807843T>C	28807843	28807843+rs75815722	rs75815722	T>C	T	C
NC_000024.9:g.22926675C>A	22926675	22926675+rs758426389	rs758426389	C>A	C	A
NC_000024.9:g.13451070C>G	13451070	13451070+rs75860833	rs75860833	C>G	C	G
NC_000024.9:g.28582716A>G	28582716	28582716+rs75866991	rs75866991	A>G	A	G
NC_000024.9:g.28803198T>A	28803198	28803198+rs75901595	rs75901595	T>A	T	A
NC_000024.9:g.28810308G>C	28810308	28810308+rs75906628	rs75906628	G>C	G	C
NC_000024.9:g.15492420T>C	15492420	15492420+rs759279119	rs759279119	T>C	T	C
NC_000024.9:g.9962506T>C	9962506	9962506+rs75950849	rs75950849	T>C	T	C
NC_000024.9:g.21848010C>T	21848010	21848010+rs759748640	rs759748640	C>T	C	T
NC_000024.9:g.28549346C>A	28549346	28549346+rs76001868	rs76001868	C>A	C	A
NC_000024.9:g.17866469G>A	17866469	17866469+rs760128565	rs760128565	G>A	G	A
NC_000024.9:g.28520255T>C	28520255	28520255+rs760362236	rs760362236	T>C	T	C
NC_000024.9:g.18118109A>C	18118109	18118109+rs760746109	rs760746109	A>C	A	C
NC_000024.9:g.23031281A>G	23031281	23031281+rs761041678	rs761041678	A>G	A	G
NC_000024.9:g.9962045A>G	9962045	9962045+rs76132179	rs76132179	A>G	A	G
NC_000024.9:g.18924606C>T	18924606	18924606+rs761442177	rs761442177	C>T	C	T
NC_000024.9:g.17732555G>A	17732555	17732555+rs761569559	rs761569559	G>A	G	A
NC_000024.9:g.58969450C>G	58969450	58969450+rs76160065	rs76160065	C>G	C	G
NC_000024.9:g.28607153C>T	28607153	28607153+rs76180116	rs76180116	C>T	C	T
NC_000024.9:g.28588453T>C	28588453	28588453+rs76201937	rs76201937	T>C	T	C
NC_000024.9:g.19042191A>G	19042191	19042191+rs762256905	rs762256905	A>G	A	G
NC_000024.9:g.28585558C>T	28585558	28585558+rs76244474	rs76244474	C>T	C	T
NC_000024.9:g.15869544A>G	15869544	15869544+rs762485020	rs762485020	A>G	A	G
NC_000024.9:g.23358218C>T	23358218	23358218+rs762960330	rs762960330	C>T	C	T
NC_000024.9:g.15987568A>C	15987568	15987568+rs762988006	rs762988006	A>C	A	C
NC_000024.9:g.18177496G>A	18177496	18177496+rs763028846	rs763028846	G>A	G	A
NC_000024.9:g.28818237A>G	28818237	28818237+rs76345297	rs76345297	A>G	A	G
NC_000024.9:g.21366043A>G	21366043	21366043+rs763766340	rs763766340	A>G	A	G
NC_000024.9:g.28709316A>T	28709316	28709316+rs76385067	rs76385067	A>T	A	T
NC_000024.9:g.14506297G>A	14506297	14506297+rs763885715	rs763885715	G>A	G	A
NC_000024.9:g.13489077T>C	13489077	13489077+rs76389422	rs76389422	T>C	T	C
NC_000024.9:g.22065387A>C	22065387	22065387+rs764445676	rs764445676	A>C	A	C
NC_000024.9:g.28809938T>G	28809938	28809938+rs76447142	rs76447142	T>G	T	G
NC_000024.9:g.28796239C>G	28796239	28796239+rs76482714	rs76482714	C>G	C	G
NC_000024.9:g.13591269G>A	13591269	13591269+rs765429796	rs765429796	G>A	G	A
NC_000024.9:g.13514981T>C	13514981	13514981+rs76555728	rs76555728	T>C	T	C

NC_000024.9:g.13591443G>A	13591443	13591443+rs76557083	rs76557083	G>A	G	A
NC_000024.9:g.28587640C>G	28587640	28587640+rs76589313	rs76589313	C>G	C	G
NC_000024.9:g.18622327C>T	18622327	18622327+rs766065848	rs766065848	C>T	C	T
NC_000024.9:g.13449198A>C	13449198	13449198+rs76608312	rs76608312	A>C	A	C
NC_000024.9:g.28585667C>G	28585667	28585667+rs76616963	rs76616963	C>G	C	G
NC_000024.9:g.18952185T>C	18952185	18952185+rs766181344	rs766181344	T>C	T	C
NC_000024.9:g.28587895C>T	28587895	28587895+rs76618880	rs76618880	C>T	C	T
NC_000024.9:g.13909032G>A	13909032	13909032+rs76624439	rs76624439	G>A	G	A
NC_000024.9:g.15599239G>A	15599239	15599239+rs766295907	rs766295907	G>A	G	A
NC_000024.9:g.17839946G>A	17839946	17839946+rs766792966	rs766792966	G>A	G	A
NC_000024.9:g.23261805G>C	23261805	23261805+rs766840015	rs766840015	G>C	G	C
NC_000024.9:g.13450578G>C	13450578	13450578+rs76690468	rs76690468	G>C	G	C
NC_000024.9:g.10014478A>G	10014478	10014478+rs76723369	rs76723369	A>G	A	G
NC_000024.9:g.28583500C>T	28583500	28583500+rs76728162	rs76728162	C>T	C	T
NC_000024.9:g.22713505G>A	22713505	22713505+rs76736067	rs76736067	G>A	G	A
NC_000024.9:g.13969160C>T	13969160	13969160+rs767717232	rs767717232	C>T	C	T
NC_000024.9:g.21461205G>A	21461205	21461205+rs767826907	rs767826907	G>A	G	A
NC_000024.9:g.21929459A>T	21929459	21929459+rs767902840	rs767902840	A>T	A	T
NC_000024.9:g.28690476A>G	28690476	28690476+rs768062536	rs768062536	A>G	A	G
NC_000024.9:g.13591415T>A	13591415	13591415+rs768319368	rs768319368	T>A	T	A
NC_000024.9:g.21651671A>C	21651671	21651671+rs768466501	rs768466501	A>C	A	C
NC_000024.9:g.21557235G>C	21557235	21557235+rs768533213	rs768533213	G>C	G	C
NC_000024.9:g.28549364G>T	28549364	28549364+rs76875841	rs76875841	G>T	G	T
NC_000024.9:g.16890047G>T	16890047	16890047+rs768798582	rs768798582	G>T	G	T
NC_000024.9:g.16750394G>A	16750394	16750394+rs769051230	rs769051230	G>A	G	A
NC_000024.9:g.16731464G>A	16731464	16731464+rs769608038	rs769608038	G>A	G	A
NC_000024.9:g.28585703G>A	28585703	28585703+rs76998257	rs76998257	G>A	G	A
NC_000024.9:g.9962028T>C	9962028	9962028+rs77002497	rs77002497	T>C	T	C
NC_000024.9:g.9952698T>C	9952698	9952698+rs77043363	rs77043363	T>C	T	C
NC_000024.9:g.28582685C>A	28582685	28582685+rs77050427	rs77050427	C>A	C	A
NC_000024.9:g.14928106C>T	14928106	14928106+rs770725321	rs770725321	C>T	C	T
NC_000024.9:g.9962570A>G	9962570	9962570+rs77081858	rs77081858	A>G	A	G
NC_000024.9:g.13448405G>A	13448405	13448405+rs77086143	rs77086143	G>A	G	A
NC_000024.9:g.14560733G>A	14560733	14560733+rs770870106	rs770870106	G>A	G	A
NC_000024.9:g.18979824G>A	18979824	18979824+rs771390204	rs771390204	G>A	G	A
NC_000024.9:g.28797308T>A	28797308	28797308+rs77144534	rs77144534	T>A	T	A
NC_000024.9:g.16723439A>G	16723439	16723439+rs771511738	rs771511738	A>G	A	G
NC_000024.9:g.17918711G>T	17918711	17918711+rs771595171	rs771595171	G>T	G	T
NC_000024.9:g.22945465C>T	22945465	22945465+rs771628136	rs771628136	C>T	C	T
NC_000024.9:g.13137115T>C	13137115	13137115+rs77169027	rs77169027	T>C	T	C
NC_000024.9:g.6977559C>T	6977559	6977559+rs771835191	rs771835191	C>T	C	T
NC_000024.9:g.18135346G>A	18135346	18135346+rs771964787	rs771964787	G>A	G	A
NC_000024.9:g.13449018A>C	13449018	13449018+rs77198017	rs77198017	A>C	A	C
NC_000024.9:g.15770655A>G	15770655	15770655+rs772054131	rs772054131	A>G	A	G
NC_000024.9:g.7332619G>A	7332619	7332619+rs772251340	rs772251340	G>A	G	A
NC_000024.9:g.28809954C>G	28809954	28809954+rs77242366	rs77242366	C>G	C	G
NC_000024.9:g.7368780G>A	7368780	7368780+rs772740434	rs772740434	G>A	G	A
NC_000024.9:g.7160590T>C	7160590	7160590+rs772782500	rs772782500	T>C	T	C
NC_000024.9:g.28791231A>T	28791231	28791231+rs77284363	rs77284363	A>T	A	T
NC_000024.9:g.9952558C>A	9952558	9952558+rs77290863	rs77290863	C>A	C	A
NC_000024.9:g.28557222C>A	28557222	28557222+rs773204935	rs773204935	C>A	C	A
NC_000024.9:g.17537276A>T	17537276	17537276+rs773312711	rs773312711	A>T	A	T
NC_000024.9:g.28582721T>C	28582721	28582721+rs77364246	rs77364246	T>C	T	C
NC_000024.9:g.28588477G>A	28588477	28588477+rs773746380	rs773746380	G>A	G	A
NC_000024.9:g.7307494T>G	7307494	7307494+rs77380062	rs77380062	T>G	T	G
NC_000024.9:g.6932096G>A	6932096	6932096+rs774423916	rs774423916	G>A	G	A
NC_000024.9:g.15146124A>T	15146124	15146124+rs774749212	rs774749212	A>T	A	T
NC_000024.9:g.15773448C>T	15773448	15773448+rs774828764	rs774828764	C>T	C	T
NC_000024.9:g.18555339G>A	18555339	18555339+rs774942606	rs774942606	G>A	G	A
NC_000024.9:g.28587124C>T	28587124	28587124+rs77495203	rs77495203	C>T	C	T
NC_000024.9:g.28587496A>G	28587496	28587496+rs77526804	rs77526804	A>G	A	G
NC_000024.9:g.28776493G>A	28776493	28776493+rs775537703	rs775537703	G>A	G	A
NC_000024.9:g.18588420A>T	18588420	18588420+rs775610410	rs775610410	A>T	A	T
NC_000024.9:g.17022540A>T	17022540	17022540+rs775626588	rs775626588	A>T	A	T
NC_000024.9:g.7437899T>G	7437899	7437899+rs776021870	rs776021870	T>G	T	G
NC_000024.9:g.15932433C>T	15932433	15932433+rs776340763	rs776340763	C>T	C	T
NC_000024.9:g.28480867C>A	28480867	28480867+rs776345368	rs776345368	C>A	C	A
NC_000024.9:g.19032464C>A	19032464	19032464+rs776598925	rs776598925	C>A	C	A
NC_000024.9:g.9952303G>A	9952303	9952303+rs77667374	rs77667374	G>A	G	A
NC_000024.9:g.28583163T>C	28583163	28583163+rs77717842	rs77717842	T>C	T	C
NC_000024.9:g.14789849G>A	14789849	14789849+rs777190332	rs777190332	G>A	G	A
NC_000024.9:g.13137093G>A	13137093	13137093+rs77747950	rs77747950	G>A	G	A
NC_000024.9:g.13909050G>A	13909050	13909050+rs77755164	rs77755164	G>A	G	A
NC_000024.9:g.14242972C>A	14242972	14242972+rs777579690	rs777579690	C>A	C	A
NC_000024.9:g.7422444T>A	7422444	7422444+rs777738765	rs777738765	T>A	T	A
NC_000024.9:g.14689958G>A	14689958	14689958+rs777976552	rs777976552	G>A	G	A
NC_000024.9:g.28549443T>C	28549443	28549443+rs77806111	rs77806111	T>C	T	C
NC_000024.9:g.28586874G>A	28586874	28586874+rs77807600	rs77807600	G>A	G	A
NC_000024.9:g.13137055T>G	13137055	13137055+rs77831461	rs77831461	T>G	T	G
NC_000024.9:g.15912128C>T	15912128	15912128+rs778433823	rs778433823	C>T	C	T
NC_000024.9:g.28588346G>A	28588346	28588346+rs77871662	rs77871662	G>A	G	A
NC_000024.9:g.6634258C>T	6634258	6634258+rs778777051	rs778777051	C>T	C	T
NC_000024.9:g.17426753G>A	17426753	17426753+rs778872605	rs778872605	G>A	G	A
NC_000024.9:g.22825462C>T	22825462	22825462+rs779104699	rs779104699	C>T	C	T
NC_000024.9:g.28807141G>C	28807141	28807141+rs77924037	rs77924037	G>C	G	C
NC_000024.9:g.23079397G>A	23079397	23079397+rs779586891	rs779586891	G>A	G	A
NC_000024.9:g.13136942G>A	13136942	13136942+rs77960862	rs77960862	G>A	G	A
NC_000024.9:g.28709271C>A	28709271	28709271+rs779810719	rs779810719	C>A	C	A
NC_000024.9:g.28810768A>C	28810768	28810768+rs779901819	rs779901819	A>C	A	C
NC_000024.9:g.14468205G>A	14468205	14468205+rs780968109	rs780968109	G>A	G	A
NC_000024.9:g.16345620A>G	16345620	16345620+rs781248245	rs781248245	A>G	A	G
NC_000024.9:g.18765784A>G	18765784	18765784+rs781252793	rs781252793	A>G	A	G
NC_000024.9:g.6979855C>A	6979855	6979855+rs781257443	rs781257443	C>A	C	A
NC_000024.9:g.9962580T>G	9962580	9962580+rs78127299	rs78127299	T>G	T	G
NC_000024.9:g.22037518G>T	22037518	22037518+rs781282637	rs781282637	G>T	G	T
NC_000024.9:g.28587749G>T	28587749	28587749+rs78132007	rs78132007	G>T	G	T
NC_000024.9:g.28587685A>C	28587685	28587685+rs78162835	rs78162835	A>C	A	C
NC_000024.9:g.28587418A>C	28587418	28587418+rs78177339	rs78177339	A>C	A	C
NC_000024.9:g.13195712T>A	13195712	13195712+rs78177491	rs78177491	T>A	T	A
NC_000024.9:g.28588218A>G	28588218	28588218+rs78203686	rs78203686	A>G	A	G
NC_000024.9:g.28549471C>A	28549471	28549471+rs78220102	rs78220102	C>A	C	A
NC_000024.9:g.19226196G>A	19226196	19226196+rs78310168	rs78310168	G>A	G	A
NC_000024.9:g.10022014G>A	10022014	10022014+rs78335276	rs78335276	G>A	G	A
NC_000024.9:g.13908787T>G	13908787	13908787+rs78348311	rs78348311	T>G	T	G

NC_000024.9:g.19071411T>C	19071411	19071411+rs78349167	rs78349167	T>C	T	C
NC_000024.9:g.28709228A>C	28709228	28709228+rs78419228	rs78419228	A>C	A	C
NC_000024.9:g.14102798A>G	14102798	14102798+rs78464143	rs78464143	A>G	A	G
NC_000024.9:g.9950584T>C	9950584	9950584+rs78489304	rs78489304	T>C	T	C
NC_000024.9:g.28583892T>C	28583892	28583892+rs78611711	rs78611711	T>C	T	C
NC_000024.9:g.9950542G>A	9950542	9950542+rs78616962	rs78616962	G>A	G	A
NC_000024.9:g.28808558C>T	28808558	28808558+rs78688155	rs78688155	C>T	C	T
NC_000024.9:g.9962397A>G	9962397	9962397+rs78689559	rs78689559	A>G	A	G
NC_000024.9:g.28584003T>C	28584003	28584003+rs78697151	rs78697151	T>C	T	C
NC_000024.9:g.9952565A>G	9952565	9952565+rs78715501	rs78715501	A>G	A	G
NC_000024.9:g.10022033A>G	10022033	10022033+rs78717746	rs78717746	A>G	A	G
NC_000024.9:g.28585444G>C	28585444	28585444+rs78826092	rs78826092	G>C	G	C
NC_000024.9:g.28583903G>A	28583903	28583903+rs78829982	rs78829982	G>A	G	A
NC_000024.9:g.10022059A>C	10022059	10022059+rs78871333	rs78871333	A>C	A	C
NC_000024.9:g.28583727G>A	28583727	28583727+rs78904837	rs78904837	G>A	G	A
NC_000024.9:g.14926420G>A	14926420	14926420+rs7892898	rs7892898	G>A	G	A
NC_000024.9:g.58969735G>A	58969735	58969735+rs7892968	rs7892968	G>A	G	A
NC_000024.9:g.58968784T>C	58968784	58968784+rs7893049	rs7893049	T>C	T	C
NC_000024.9:g.14585324C>T	14585324	14585324+rs7893102	rs7893102	C>T	C	T
NC_000024.9:g.28585594T>C	28585594	28585594+rs78952397	rs78952397	T>C	T	C
NC_000024.9:g.28587420C>T	28587420	28587420+rs79086004	rs79086004	C>T	C	T
NC_000024.9:g.10014561A>G	10014561	10014561+rs79103418	rs79103418	A>G	A	G
NC_000024.9:g.28791300G>A	28791300	28791300+rs79104491	rs79104491	G>A	G	A
NC_000024.9:g.28549475A>T	28549475	28549475+rs79129440	rs79129440	A>T	A	T
NC_000024.9:g.28587820T>C	28587820	28587820+rs79154417	rs79154417	T>C	T	C
NC_000024.9:g.13137079G>C	13137079	13137079+rs79205937	rs79205937	G>C	G	C
NC_000024.9:g.9950703C>T	9950703	9950703+rs79243195	rs79243195	C>T	C	T
NC_000024.9:g.17780336G>T	17780336	17780336+rs79274875	rs79274875	G>T	G	T
NC_000024.9:g.9950504A>G	9950504	9950504+rs79488679	rs79488679	A>G	A	G
NC_000024.9:g.28587697C>T	28587697	28587697+rs79534436	rs79534436	C>T	C	T
NC_000024.9:g.28807900T>A	28807900	28807900+rs79555435	rs79555435	T>A	T	A
NC_000024.9:g.13137100A>C	13137100	13137100+rs79567470	rs79567470	A>C	A	C
NC_000024.9:g.13982835C>T	13982835	13982835+rs796265632	rs796265632	C>T	C	T
NC_000024.9:g.28586959T>C	28586959	28586959+rs79654653	rs79654653	T>C	T	C
NC_000024.9:g.9950503C>T	9950503	9950503+rs79734930	rs79734930	C>T	C	T
NC_000024.9:g.28804197T>G	28804197	28804197+rs79797149	rs79797149	T>G	T	G
NC_000024.9:g.13449189G>A	13449189	13449189+rs79823856	rs79823856	G>A	G	A
NC_000024.9:g.13447381T>C	13447381	13447381+rs79918872	rs79918872	T>C	T	C
NC_000024.9:g.9952407T>A	9952407	9952407+rs79993451	rs79993451	T>A	T	A
NC_000024.9:g.13451033A>T	13451033	13451033+rs80075857	rs80075857	A>T	A	T
NC_000024.9:g.28587530C>T	28587530	28587530+rs80100012	rs80100012	C>T	C	T
NC_000024.9:g.28583850G>A	28583850	28583850+rs80101284	rs80101284	G>A	G	A
NC_000024.9:g.28585669G>A	28585669	28585669+rs80126603	rs80126603	G>A	G	A
NC_000024.9:g.9962531A>T	9962531	9962531+rs80128657	rs80128657	A>T	A	T
NC_000024.9:g.28797612A>T	28797612	28797612+rs80180048	rs80180048	A>T	A	T
NC_000024.9:g.10014508G>T	10014508	10014508+rs80190784	rs80190784	G>T	G	T
NC_000024.9:g.9952617T>C	9952617	9952617+rs80225156	rs80225156	T>C	T	C
NC_000024.9:g.28588206T>C	28588206	28588206+rs80246349	rs80246349	T>C	T	C
NC_000024.9:g.21853778T>A	21853778	21853778+rs865869481	rs865869481	T>A	T	A
NC_000024.9:g.7038691C>T	7038691	7038691+rs866722098	rs866722098	C>T	C	T
NC_000024.9:g.7428075G>C	7428075	7428075+rs867146356	rs867146356	G>C	G	C
NC_000024.9:g.14107438C>T	14107438	14107438+rs867343413	rs867343413	C>T	C	T
NC_000024.9:g.7428072A>G	7428072	7428072+rs868428726	rs868428726	A>G	A	G
NC_000024.9:g.58980525T>C	58980525	58980525+rs878925277	rs878925277	T>C	T	C
NC_000024.9:g.58982081A>C	58982081	58982081+rs878996945	rs878996945	A>C	A	C
NC_000024.9:g.14107314T>C	14107314	14107314+rs879016544	rs879016544	T>C	T	C
NC_000024.9:g.58979276C>T	58979276	58979276+rs879095517	rs879095517	C>T	C	T
NC_000024.9:g.23383856A>T	23383856	23383856+rs889121016	rs889121016	A>T	A	T
NC_000024.9:g.18176035G>T	18176035	18176035+rs889400501	rs889400501	G>T	G	T
NC_000024.9:g.21386617G>A	21386617	21386617+rs890463802	rs890463802	G>A	G	A
NC_000024.9:g.17639306G>A	17639306	17639306+rs901009635	rs901009635	G>A	G	A
NC_000024.9:g.15763445C>T	15763445	15763445+rs901422444	rs901422444	C>T	C	T
NC_000024.9:g.16455096C>G	16455096	16455096+rs902195825	rs902195825	C>G	C	G
NC_000024.9:g.17421219C>T	17421219	17421219+rs902427588	rs902427588	C>T	C	T
NC_000024.9:g.2728206C>T	2728206	2728206+rs904289982	rs904289982	C>T	C	T
NC_000024.9:g.2714628G>A	2714628	2714628+rs904841705	rs904841705	G>A	G	A
NC_000024.9:g.13489166C>A	13489166	13489166+rs906552741	rs906552741	C>A	C	A
NC_000024.9:g.22188172T>A	22188172	22188172+rs908935865	rs908935865	T>A	T	A
NC_000024.9:g.15921430C>T	15921430	15921430+rs909507726	rs909507726	C>T	C	T
NC_000024.9:g.17415778T>C	17415778	17415778+rs913635377	rs913635377	T>C	T	C
NC_000024.9:g.22977547C>A	22977547	22977547+rs914229123	rs914229123	C>A	C	A
NC_000024.9:g.19055971T>C	19055971	19055971+rs921044611	rs921044611	T>C	T	C
NC_000024.9:g.22020019A>G	22020019	22020019+rs922304047	rs922304047	A>G	A	G
NC_000024.9:g.16973052G>A	16973052	16973052+rs925676630	rs925676630	G>A	G	A
NC_000024.9:g.21074609G>A	21074609	21074609+rs925840105	rs925840105	G>A	G	A
NC_000024.9:g.6824369G>T	6824369	6824369+rs928686081	rs928686081	G>T	G	T
NC_000024.9:g.21804153C>T	21804153	21804153+rs932809570	rs932809570	C>T	C	T
NC_000024.9:g.22738079C>G	22738079	22738079+rs9341311	rs9341311	C>G	C	G
NC_000024.9:g.21436981C>T	21436981	21436981+rs935160198	rs935160198	C>T	C	T
NC_000024.9:g.22718987G>T	22718987	22718987+rs935760402	rs935760402	G>T	G	T
NC_000024.9:g.22850721C>G	22850721	22850721+rs936881087	rs936881087	C>G	C	G
NC_000024.9:g.22865763G>A	22865763	22865763+rs938899553	rs938899553	G>A	G	A
NC_000024.9:g.16372149A>G	16372149	16372149+rs939225918	rs939225918	A>G	A	G
NC_000024.9:g.21478364G>A	21478364	21478364+rs940301475	rs940301475	G>A	G	A
NC_000024.9:g.17143036A>G	17143036	17143036+rs941955256	rs941955256	A>G	A	G
NC_000024.9:g.21105988T>C	21105988	21105988+rs945837494	rs945837494	T>C	T	C
NC_000024.9:g.7058472G>T	7058472	7058472+rs945959575	rs945959575	G>T	G	T
NC_000024.9:g.22168501G>T	22168501	22168501+rs948017847	rs948017847	G>T	G	T
NC_000024.9:g.17286473C>T	17286473	17286473+rs949592958	rs949592958	C>T	C	T
NC_000024.9:g.16422586G>A	16422586	16422586+rs953294795	rs953294795	G>A	G	A
NC_000024.9:g.18177626C>T	18177626	18177626+rs953674897	rs953674897	C>T	C	T
NC_000024.9:g.21032877A>G	21032877	21032877+rs954094692	rs954094692	A>G	A	G
NC_000024.9:g.19245831G>A	19245831	19245831+rs954223960	rs954223960	G>A	G	A
NC_000024.9:g.28588261G>A	28588261	28588261+rs954231116	rs954231116	G>A	G	A
NC_000024.9:g.6931586T>C	6931586	6931586+rs954711297	rs954711297	T>C	T	C
NC_000024.9:g.21477220C>A	21477220	21477220+rs954962846	rs954962846	C>A	C	A
NC_000024.9:g.21665747A>G	21665747	21665747+rs955053996	rs955053996	A>G	A	G
NC_000024.9:g.19123988C>T	19123988	19123988+rs964048816	rs964048816	C>T	C	T
NC_000024.9:g.16029626T>C	16029626	16029626+rs9645172	rs9645172	T>C	T	C
NC_000024.9:g.14107068A>C	14107068	14107068+rs9650861	rs9650861	A>C	A	C
NC_000024.9:g.14433405T>C	14433405	14433405+rs966375764	rs966375764	T>C	T	C
NC_000024.9:g.19282005A>G	19282005	19282005+rs967510332	rs967510332	A>G	A	G
NC_000024.9:g.16985712C>T	16985712	16985712+rs967817295	rs967817295	C>T	C	T
NC_000024.9:g.4502746A>G	4502746	4502746+rs9699387	rs9699387	A>G	A	G

NC_000024.9:g.19161644T>C	19161644	19161644+rs969966954	rs969966954	T>C	T	C
NC_000024.9:g.6931580A>G	6931580	6931580+rs974882411	rs974882411	A>G	A	G
NC_000024.9:g.21694722C>G	21694722	21694722+rs975253068	rs975253068	C>G	C	G
NC_000024.9:g.17577781T>C	17577781	17577781+rs975757081	rs975757081	T>C	T	C
NC_000024.9:g.17087047T>G	17087047	17087047+rs9785656	rs9785656	T>G	T	G
NC_000024.9:g.3967342C>T	3967342	3967342+rs9785679	rs9785679	C>T	C	T
NC_000024.9:g.28680422C>T	28680422	28680422+rs9785798	rs9785798	C>T	C	T
NC_000024.9:g.28792268T>C	28792268	28792268+rs9785925	rs9785925	T>C	T	C
NC_000024.9:g.28470600A>C	28470600	28470600+rs9786004	rs9786004	A>C	A	C
NC_000024.9:g.21499869T>G	21499869	21499869+rs9786031	rs9786031	T>G	T	G
NC_000024.9:g.17534389A>T	17534389	17534389+rs9786213	rs9786213	A>T	A	T
NC_000024.9:g.14415575A>T	14415575	14415575+rs9786343	rs9786343	A>T	A	T
NC_000024.9:g.19397759G>A	19397759	19397759+rs978638896	rs978638896	G>A	G	A
NC_000024.9:g.28818131A>G	28818131	28818131+rs9786440	rs9786440	A>G	A	G
NC_000024.9:g.28797251A>G	28797251	28797251+rs9786549	rs9786549	A>G	A	G
NC_000024.9:g.28815864C>T	28815864	28815864+rs9786623	rs9786623	C>T	C	T
NC_000024.9:g.28816831C>T	28816831	28816831+rs9786648	rs9786648	C>T	C	T
NC_000024.9:g.28792487T>C	28792487	28792487+rs9786665	rs9786665	T>C	T	C
NC_000024.9:g.14199507C>A	14199507	14199507+rs979911690	rs979911690	C>A	C	A
NC_000024.9:g.18619784G>T	18619784	18619784+rs986207190	rs986207190	G>T	G	T
NC_000024.9:g.15286246C>T	15286246	15286246+rs986392329	rs986392329	C>T	C	T
NC_000024.9:g.28784130C>T	28784130	28784130+rs989121074	rs989121074	C>T	C	T
NC_000024.9:g.17156134A>C	17156134	17156134+rs991353219	rs991353219	A>C	A	C
NC_000024.9:g.15136985A>G	15136985	15136985+rs993452092	rs993452092	A>G	A	G
NC_000024.9:g.18943472C>G	18943472	18943472+rs995312219	rs995312219	C>G	C	G
NC_000024.9:g.2889805G>C	2889805	2889805+rs997109981	rs997109981	G>C	G	C
NC_000024.9:g.6826834T>C	6826834	6826834+S3028^^,R1b1a1b1a1a2c1a5a2a1a1	S3028^^,R1b1a1b1a1a2c1a5a2a1a1	T>C	T	C
NC_000024.9:g.7391202T>C	7391202	7391202+S5025,Z2174,R1b1b	S5025,Z2174,R1b1b	T>C	T	C
NC_000024.9:g.2892835G>A	2892835	2892835+S8460,I2a1b1a2a1~	S8460,I2a1b1a2a1~	G>A	G	A
NC_000024.9:g.8148836A>G	8148836	8148836+SA05	SA05	A>G	A	G
NC_000024.9:g.6931891C>T	6931891	6931891+SA29	SA29	C>T	C	T
NC_000024.9:g.15508183G>A	15508183	15508183+Y1_15508183	Y1_15508183	G>A	G	A
NC_000024.9:g.17922077A>G	17922077	17922077+Y1_17922077	Y1_17922077	A>G	A	G
NC_000024.9:g.19179327G>A	19179327	19179327+Y1_19179327	Y1_19179327	G>A	G	A
NC_000024.9:g.21609949A>G	21609949	21609949+Y1_21609949	Y1_21609949	A>G	A	G
NC_000024.9:g.7192964T>C	7192964	7192964+Y1_7192964	Y1_7192964	T>C	T	C
NC_000024.9:g.6894185C>G	6894185	6894185+Y1_rs1603040369	Y1_rs1603040369	C>G	C	G
NC_000024.9:g.17657595G>T	17657595	17657595+Y1_rs541403360	Y1_rs541403360	G>T	G	T
NC_000024.9:g.17657596A>T	17657596	17657596+Y1_rs564763483	Y1_rs564763483	A>T	A	T
NC_000024.9:g.21716226C>T	21716226	21716226+Y1_rs779238512	Y1_rs779238512	C>T	C	T
NC_000024.9:g.18052819G>A	18052819	18052819+Y101104	Y101104	G>A	G	A
NC_000024.9:g.6619374C>T	6619374	6619374+Y1036,Q2	Y1036,Q2	C>T	C	T
NC_000024.9:g.19170794C>T	19170794	19170794+Y103889	Y103889	C>T	C	T
NC_000024.9:g.7265818A>G	7265818	7265818+Y1041,Q2	Y1041,Q2	A>G	A	G
NC_000024.9:g.13885426G>T	13885426	13885426+Y1064,Q2	Y1064,Q2	G>T	G	T
NC_000024.9:g.14079002T>C	14079002	14079002+Y1067,Q2	Y1067,Q2	T>C	T	C
NC_000024.9:g.14792841G>A	14792841	14792841+Y1071,Q2	Y1071,Q2	G>A	G	A
NC_000024.9:g.21675854C>T	21675854	21675854+Y107197	Y107197	C>T	C	T
NC_000024.9:g.15379441G>T	15379441	15379441+Y1073,Q2	Y1073,Q2	G>T	G	T
NC_000024.9:g.15715397G>C	15715397	15715397+Y1074,Q2	Y1074,Q2	G>C	G	C
NC_000024.9:g.15825332G>A	15825332	15825332+Y1077,Q2	Y1077,Q2	G>A	G	A
NC_000024.9:g.7994080G>A	7994080	7994080+Y10781	Y10781	G>A	G	A
NC_000024.9:g.16656126C>T	16656126	16656126+Y10783	Y10783	C>T	C	T
NC_000024.9:g.16276215G>T	16276215	16276215+Y1080,Q2	Y1080,Q2	G>T	G	T
NC_000024.9:g.17796644A>C	17796644	17796644+Y1093,Q2	Y1093,Q2	A>C	A	C
NC_000024.9:g.28772907C>T	28772907	28772907+Y10981	Y10981	C>T	C	T
NC_000024.9:g.18958311T>C	18958311	18958311+Y1100,Q2	Y1100,Q2	T>C	T	C
NC_000024.9:g.21722478A>T	21722478	21722478+Y1108,Q2	Y1108,Q2	A>T	A	T
NC_000024.9:g.21807829G>A	21807829	21807829+Y1110,Q2	Y1110,Q2	G>A	G	A
NC_000024.9:g.21880542G>C	21880542	21880542+Y1113,Q2	Y1113,Q2	G>C	G	C
NC_000024.9:g.22026705A>G	22026705	22026705+Y1115,Q2	Y1115,Q2	A>G	A	G
NC_000024.9:g.22123352G>T	22123352	22123352+Y1116,Q2	Y1116,Q2	G>T	G	T
NC_000024.9:g.23136457C>G	23136457	23136457+Y1122,Q2	Y1122,Q2	C>G	C	G
NC_000024.9:g.23253927G>A	23253927	23253927+Y1124,Q2	Y1124,Q2	G>A	G	A
NC_000024.9:g.23387192A>G	23387192	23387192+Y1125,Q2	Y1125,Q2	A>G	A	G
NC_000024.9:g.23418531G>A	23418531	23418531+Y1126,Q2	Y1126,Q2	G>A	G	A
NC_000024.9:g.28782579C>A	28782579	28782579+Y1136,Q2	Y1136,Q2	C>A	C	A
NC_000024.9:g.7703250G>A	7703250	7703250+Y11655	Y11655	G>A	G	A
NC_000024.9:g.22601953C>T	22601953	22601953+Y11659	Y11659	C>T	C	T
NC_000024.9:g.6693564C>T	6693564	6693564+Y12422^,Z19568^,Q1b1a1a1e2	Y12422^,Z19568^,Q1b1a1a1e2	C>T	C	T
NC_000024.9:g.6759419G>A	6759419	6759419+Y12423,Z19571,Q1b1a1a1e2	Y12423,Z19571,Q1b1a1a1e2	G>A	G	A
NC_000024.9:g.15144082T>G	15144082	15144082+Y12430,Z19617,Q1b1a1a1e2	Y12430,Z19617,Q1b1a1a1e2	T>G	T	G
NC_000024.9:g.15426792A>G	15426792	15426792+Y12432,Z19621,Q1b1a1a1e2	Y12432,Z19621,Q1b1a1a1e2	A>G	A	G
NC_000024.9:g.15632852A>G	15632852	15632852+Y12433,Z19624,Q1b1a1a1e2	Y12433,Z19624,Q1b1a1a1e2	A>G	A	G
NC_000024.9:g.19122369C>T	19122369	19122369+Y12436,Z19645,Q1b1a1a1e2	Y12436,Z19645,Q1b1a1a1e2	C>T	C	T
NC_000024.9:g.19477755T>C	19477755	19477755+Y12439	Y12439	T>C	T	C
NC_000024.9:g.22748802G>A	22748802	22748802+Y12440,Z19658,Q1b1a1a1e2	Y12440,Z19658,Q1b1a1a1e2	G>A	G	A
NC_000024.9:g.22817295C>A	22817295	22817295+Y12441,Z19659,Q1b1a1a1e2	Y12441,Z19659,Q1b1a1a1e2	C>A	C	A
NC_000024.9:g.23314666C>T	23314666	23314666+Y12442,Z19666,Q1b1a1a1e2	Y12442,Z19666,Q1b1a1a1e2	C>T	C	T
NC_000024.9:g.22062036C>T	22062036	22062036	Y126607	C>T	C	T
NC_000024.9:g.15835884C>T	15835884	15835884+Y126611	Y126611	C>T	C	T
NC_000024.9:g.13622899A>G	13622899	13622899+Y126613	Y126613	A>G	A	G
NC_000024.9:g.8157693T>C	8157693	8157693+Y126614	Y126614	T>C	T	C
NC_000024.9:g.22440500A>C	22440500	22440500+Y126688	Y126688	A>C	A	C
NC_000024.9:g.21996649T>A	21996649	21996649+Y126708	Y126708	T>A	T	A
NC_000024.9:g.22471020A>T	22471020	22471020+Y126908	Y126908	A>T	A	T
NC_000024.9:g.23546798C>T	23546798	23546798+Y128058	Y128058	C>T	C	T
NC_000024.9:g.2795875G>A	2795875	2795875+Y128306,G2a2b2a1b2	Y128306,G2a2b2a1b2	G>A	G	A
NC_000024.9:g.9040440C>T	9040440	9040440+Y12874	Y12874	C>T	C	T
NC_000024.9:g.6372937G>T	6372937	6372937+Y12875	Y12875	G>T	G	T
NC_000024.9:g.23139197G>A	23139197	23139197+Y12876,Z19663,Q1b1a1a1e2	Y12876,Z19663,Q1b1a1a1e2	G>A	G	A
NC_000024.9:g.6740971A>C	6740971	6740971+Y12877,Z19570,Q1b1a1a1e2	Y12877,Z19570,Q1b1a1a1e2	A>C	A	C
NC_000024.9:g.21112760T>C	21112760	21112760+Y12878,Z19648,Q1b1a1a1e2	Y12878,Z19648,Q1b1a1a1e2	T>C	T	C
NC_000024.9:g.18843866G>T	18843866	18843866+Y12881,Z19640,Q1b1a1a1e2	Y12881,Z19640,Q1b1a1a1e2	G>T	G	T
NC_000024.9:g.14396812C>T	14396812	14396812+Y12882,Z19608,Q1b1a1a1e2	Y12882,Z19608,Q1b1a1a1e2	C>T	C	T
NC_000024.9:g.8202085A>T	8202085	8202085+Y129666	Y129666	A>T	A	T
NC_000024.9:g.23617849C>T	23617849	23617849+Y129719	Y129719	C>T	C	T
NC_000024.9:g.9463688C>T	9463688	9463688+Y129743	Y129743	C>T	C	T
NC_000024.9:g.9879006G>A	9879006	9879006+Y129963	Y129963	G>A	G	A
NC_000024.9:g.28769764C>T	28769764	28769764+Y139737	Y139737	C>T	C	T
NC_000024.9:g.23808866G>T	23808866	23808866+Y152500	Y152500	G>T	G	T
NC_000024.9:g.8474328C>A	8474328	8474328+Y152512	Y152512	C>A	C	A
NC_000024.9:g.7821499T>C	7821499	7821499+Y152529	Y152529	T>C	T	C
NC_000024.9:g.15701678A>T	15701678	15701678+Y152535	Y152535	A>T	A	T

NC_000024.9:g.3901680A>T	3901680	3901680+Y159338	Y159338	A>T	A	T
NC_000024.9:g.17179204G>A	17179204	17179204+Y16253	Y16253	G>A	G	A
NC_000024.9:g.17711949G>A	17711949	17711949+Y16270,E1b1b1a1b1a10b2b1~	Y16270,E1b1b1a1b1a10b2b1~	G>A	G	A
NC_000024.9:g.21346052C>T	21346052	21346052+Y16336	Y16336	C>T	C	T
NC_000024.9:g.8060702A>G	8060702	8060702+Y16337	Y16337	A>G	A	G
NC_000024.9:g.15235515A>C	15235515	15235515+Y16338	Y16338	A>C	A	C
NC_000024.9:g.2767940T>C	2767940	2767940+Y165184	Y165184	T>C	T	C
NC_000024.9:g.7781746C>T	7781746	7781746+Y165185	Y165185	C>T	C	T
NC_000024.9:g.19227228C>T	19227228	19227228+Y165187	Y165187	C>T	C	T
NC_000024.9:g.8002012G>T	8002012	8002012+Y165188	Y165188	G>T	G	T
NC_000024.9:g.19150027G>A	19150027	19150027+Y165189	Y165189	G>A	G	A
NC_000024.9:g.7591385G>T	7591385	7591385+Y165190	Y165190	G>T	G	T
NC_000024.9:g.8449247G>A	8449247	8449247+Y165191	Y165191	G>A	G	A
NC_000024.9:g.8569711T>A	8569711	8569711+Y165192	Y165192	T>A	T	A
NC_000024.9:g.15850813G>A	15850813	15850813+Y165193	Y165193	G>A	G	A
NC_000024.9:g.23480327G>A	23480327	23480327+Y165194	Y165194	G>A	G	A
NC_000024.9:g.23523805C>T	23523805	23523805+Y165195	Y165195	C>T	C	T
NC_000024.9:g.18641163A>C	18641163	18641163+Y165196	Y165196	A>C	A	C
NC_000024.9:g.14877353A>G	14877353	14877353+Y165197	Y165197	A>G	A	G
NC_000024.9:g.7814518T>A	7814518	7814518+Y165198	Y165198	T>A	T	A
NC_000024.9:g.8267877A>G	8267877	8267877+Y165199	Y165199	A>G	A	G
NC_000024.9:g.24497510C>A	24497510	24497510+Y165200	Y165200	C>A	C	A
NC_000024.9:g.8474837G>T	8474837	8474837+Y165201	Y165201	G>T	G	T
NC_000024.9:g.14967916T>C	14967916	14967916+Y165202	Y165202	T>C	T	C
NC_000024.9:g.8533214C>G	8533214	8533214+Y165205	Y165205	C>G	C	G
NC_000024.9:g.7841584A>G	7841584	7841584+Y165206	Y165206	A>G	A	G
NC_000024.9:g.9896919G>T	9896919	9896919+Y165207	Y165207	G>T	G	T
NC_000024.9:g.8145108G>A	8145108	8145108+Y165208	Y165208	G>A	G	A
NC_000024.9:g.8369477C>T	8369477	8369477+Y165210	Y165210	C>T	C	T
NC_000024.9:g.23766935A>G	23766935	23766935+Y165211	Y165211	A>G	A	G
NC_000024.9:g.7768399G>T	7768399	7768399+Y166136	Y166136	G>T	G	T
NC_000024.9:g.8211776C>T	8211776	8211776+Y166140	Y166140	C>T	C	T
NC_000024.9:g.19547652A>G	19547652	19547652+Y166229	Y166229	A>G	A	G
NC_000024.9:g.15537082T>C	15537082	15537082+Y166310	Y166310	T>C	T	C
NC_000024.9:g.8680843C>T	8680843	8680843+Y166446	Y166446	C>T	C	T
NC_000024.9:g.18837422G>T	18837422	18837422+Y166698	Y166698	G>T	G	T
NC_000024.9:g.14429344A>T	14429344	14429344+Y166699	Y166699	A>T	A	T
NC_000024.9:g.22161039T>C	22161039	22161039+Y166700	Y166700	T>C	T	C
NC_000024.9:g.13686267T>C	13686267	13686267+Y166701	Y166701	T>C	T	C
NC_000024.9:g.14136897T>C	14136897	14136897+Y166702	Y166702	T>C	T	C
NC_000024.9:g.13231561A>G	13231561	13231561+Y166703	Y166703	A>G	A	G
NC_000024.9:g.14522208C>A	14522208	14522208+Y166704	Y166704	C>A	C	A
NC_000024.9:g.22066123T>C	22066123	22066123+Y166705	Y166705	T>C	T	C
NC_000024.9:g.14800135G>A	14800135	14800135+Y166706	Y166706	G>A	G	A
NC_000024.9:g.17914435A>G	17914435	17914435+Y166707	Y166707	A>G	A	G
NC_000024.9:g.8835537G>A	8835537	8835537+Y17425	Y17425	G>A	G	A
NC_000024.9:g.4119445A>G	4119445	4119445+Y17850	Y17850	A>G	A	G
NC_000024.9:g.13897865G>T	13897865	13897865+Y17851	Y17851	G>T	G	T
NC_000024.9:g.21035805T>C	21035805	21035805+Y17852	Y17852	T>C	T	C
NC_000024.9:g.22222120T>G	22222120	22222120+Y17853	Y17853	T>G	T	G
NC_000024.9:g.19564909G>A	19564909	19564909+Y179986	Y179986	G>A	G	A
NC_000024.9:g.21967585C>G	21967585	21967585+Y182311	Y182311	C>G	C	G
NC_000024.9:g.13608881C>T	13608881	13608881+Y197029	Y197029	C>T	C	T
NC_000024.9:g.14366724G>A	14366724	14366724+Y2_MG11	Y2_MG11	G>A	G	A
NC_000024.9:g.14432230G>A	14432230	14432230+Y2_MG13	Y2_MG13	G>A	G	A
NC_000024.9:g.15399584G>C	15399584	15399584+Y2_MG15	Y2_MG15	G>C	G	C
NC_000024.9:g.19554722A>C	19554722	19554722+Y202433	Y202433	A>C	A	C
NC_000024.9:g.6854226T>C	6854226	6854226+Y202434	Y202434	T>C	T	C
NC_000024.9:g.23423931A>G	23423931	23423931+Y202435	Y202435	A>G	A	G
NC_000024.9:g.21523544C>A	21523544	21523544+Y202436	Y202436	C>A	C	A
NC_000024.9:g.21275494G>A	21275494	21275494+Y202437	Y202437	G>A	G	A
NC_000024.9:g.17738783T>C	17738783	17738783+Y202438	Y202438	T>C	T	C
NC_000024.9:g.15580627T>C	15580627	15580627+Y202439	Y202439	T>C	T	C
NC_000024.9:g.14189693T>C	14189693	14189693+Y202440	Y202440	T>C	T	C
NC_000024.9:g.8674555A>G	8674555	8674555+Y202441	Y202441	A>G	A	G
NC_000024.9:g.6813886T>C	6813886	6813886+Y202442	Y202442	T>C	T	C
NC_000024.9:g.6428382T>G	6428382	6428382+Y202443	Y202443	T>G	T	G
NC_000024.9:g.18201774C>G	18201774	18201774+Y202444	Y202444	C>G	C	G
NC_000024.9:g.17105746T>C	17105746	17105746+Y202445	Y202445	T>C	T	C
NC_000024.9:g.15766616A>G	15766616	15766616+Y202446	Y202446	A>G	A	G
NC_000024.9:g.22107850T>C	22107850	22107850+Y202447	Y202447	T>C	T	C
NC_000024.9:g.19443427G>A	19443427	19443427+Y202448	Y202448	G>A	G	A
NC_000024.9:g.18214398G>T	18214398	18214398+Y202449	Y202449	G>T	G	T
NC_000024.9:g.17005428A>T	17005428	17005428+Y202450	Y202450	A>T	A	T
NC_000024.9:g.16748109A>G	16748109	16748109+Y202451	Y202451	A>G	A	G
NC_000024.9:g.15710652C>T	15710652	15710652+Y202452	Y202452	C>T	C	T
NC_000024.9:g.15304037C>A	15304037	15304037+Y202453	Y202453	C>A	C	A
NC_000024.9:g.15272072G>A	15272072	15272072+Y202454	Y202454	G>A	G	A
NC_000024.9:g.14726056T>C	14726056	14726056+Y202455	Y202455	T>C	T	C
NC_000024.9:g.10014487G>C	10014487	10014487+Y202456	Y202456	G>C	G	C
NC_000024.9:g.21700304A>C	21700304	21700304+Y202457	Y202457	A>C	A	C
NC_000024.9:g.17677935C>T	17677935	17677935+Y202458	Y202458	C>T	C	T
NC_000024.9:g.14200666G>A	14200666	14200666+Y202459	Y202459	G>A	G	A
NC_000024.9:g.28508575G>A	28508575	28508575+Y202460	Y202460	G>A	G	A
NC_000024.9:g.22007099G>A	22007099	22007099+Y202461	Y202461	G>A	G	A
NC_000024.9:g.14284498C>T	14284498	14284498+Y202462	Y202462	C>T	C	T
NC_000024.9:g.24451706A>G	24451706	24451706+Y202463	Y202463	A>G	A	G
NC_000024.9:g.22207745G>A	22207745	22207745+Y202464	Y202464	G>A	G	A
NC_000024.9:g.19005889C>A	19005889	19005889+Y202465	Y202465	C>A	C	A
NC_000024.9:g.10050769C>T	10050769	10050769+Y202466	Y202466	C>T	C	T
NC_000024.9:g.9125019T>C	9125019	9125019+Y202467	Y202467	T>C	T	C
NC_000024.9:g.5885951C>T	5885951	5885951+Y202468	Y202468	C>T	C	T
NC_000024.9:g.21497306C>G	21497306	21497306+Y202469	Y202469	C>G	C	G
NC_000024.9:g.21240284G>A	21240284	21240284+Y202470	Y202470	G>A	G	A
NC_000024.9:g.18422480G>A	18422480	18422480+Y202471	Y202471	G>A	G	A
NC_000024.9:g.18215018A>T	18215018	18215018+Y202472	Y202472	A>T	A	T
NC_000024.9:g.17124465G>A	17124465	17124465+Y202473	Y202473	G>A	G	A
NC_000024.9:g.16758741T>G	16758741	16758741+Y202474	Y202474	T>G	T	G
NC_000024.9:g.23197512G>A	23197512	23197512+Y202475	Y202475	G>A	G	A
NC_000024.9:g.22858848G>C	22858848	22858848+Y202476	Y202476	G>C	G	C
NC_000024.9:g.21485762G>A	21485762	21485762+Y202477	Y202477	G>A	G	A
NC_000024.9:g.15842755T>A	15842755	15842755+Y202478	Y202478	T>A	T	A
NC_000024.9:g.15822824T>C	15822824	15822824+Y202479	Y202479	T>C	T	C
NC_000024.9:g.15804892C>A	15804892	15804892+Y202480	Y202480	C>A	C	A

NC_000024.9:g.14296251G>A	14296251	14296251+Y202481	Y202481	G>A	G	A
NC_000024.9:g.9819858G>A	9819858	9819858+Y202482	Y202482	G>A	G	A
NC_000024.9:g.21998186T>A	21998186	21998186+Y202483	Y202483	T>A	T	A
NC_000024.9:g.17092976G>A	17092976	17092976+Y202484	Y202484	G>A	G	A
NC_000024.9:g.9813378T>C	9813378	9813378+Y202485	Y202485	T>C	T	C
NC_000024.9:g.23532768G>A	23532768	23532768+Y202486	Y202486	G>A	G	A
NC_000024.9:g.23455456G>A	23455456	23455456+Y202487	Y202487	G>A	G	A
NC_000024.9:g.18190085A>G	18190085	18190085+Y202488	Y202488	A>G	A	G
NC_000024.9:g.23575562G>T	23575562	23575562+Y202489	Y202489	G>T	G	T
NC_000024.9:g.22570053G>A	22570053	22570053+Y202490	Y202490	G>A	G	A
NC_000024.9:g.19262126G>T	19262126	19262126+Y202491	Y202491	G>T	G	T
NC_000024.9:g.18929471A>G	18929471	18929471+Y202492	Y202492	A>G	A	G
NC_000024.9:g.17900548C>T	17900548	17900548+Y202493	Y202493	C>T	C	T
NC_000024.9:g.17745107G>A	17745107	17745107+Y202494	Y202494	G>A	G	A
NC_000024.9:g.15173923T>G	15173923	15173923+Y202495	Y202495	T>G	T	G
NC_000024.9:g.9820506C>A	9820506	9820506+Y202496	Y202496	C>A	C	A
NC_000024.9:g.14430467A>G	14430467	14430467+Y202497	Y202497	A>G	A	G
NC_000024.9:g.4442904C>T	4442904	4442904+Y202498	Y202498	C>T	C	T
NC_000024.9:g.23456168T>C	23456168	23456168+Y202499	Y202499	T>C	T	C
NC_000024.9:g.8498871T>G	8498871	8498871+Y202500	Y202500	T>G	T	G
NC_000024.9:g.6767482A>T	6767482	6767482+Y202501	Y202501	A>T	A	T
NC_000024.9:g.21309307T>C	21309307	21309307+Y202502	Y202502	T>C	T	C
NC_000024.9:g.18988639C>T	18988639	18988639+Y202503	Y202503	C>T	C	T
NC_000024.9:g.16839206G>A	16839206	16839206+Y202504	Y202504	G>A	G	A
NC_000024.9:g.16561904T>G	16561904	16561904+Y202505	Y202505	T>G	T	G
NC_000024.9:g.14287289A>G	14287289	14287289+Y202506	Y202506	A>G	A	G
NC_000024.9:g.6914845A>T	6914845	6914845+Y202507	Y202507	A>T	A	T
NC_000024.9:g.23951762T>G	23951762	23951762+Y202508	Y202508	T>G	T	G
NC_000024.9:g.21471902T>C	21471902	21471902+Y202509	Y202509	T>C	T	C
NC_000024.9:g.19160961T>C	19160961	19160961+Y202510	Y202510	T>C	T	C
NC_000024.9:g.4532715A>G	4532715	4532715+Y202511	Y202511	A>G	A	G
NC_000024.9:g.2803169G>C	2803169	2803169+Y202512	Y202512	G>C	G	C
NC_000024.9:g.9445406A>G	9445406	9445406+Y202513	Y202513	A>G	A	G
NC_000024.9:g.13878632A>G	13878632	13878632+Y202514	Y202514	A>G	A	G
NC_000024.9:g.7965330G>A	7965330	7965330+Y202515	Y202515	G>A	G	A
NC_000024.9:g.7809354C>A	7809354	7809354+Y202516	Y202516	C>A	C	A
NC_000024.9:g.6405525C>T	6405525	6405525+Y202517	Y202517	C>T	C	T
NC_000024.9:g.8111697A>G	8111697	8111697+Y202518	Y202518	A>G	A	G
NC_000024.9:g.7576068G>A	7576068	7576068+Y202519	Y202519	G>A	G	A
NC_000024.9:g.28745476T>C	28745476	28745476+Y202520	Y202520	T>C	T	C
NC_000024.9:g.9393106T>G	9393106	9393106+Y202521	Y202521	T>G	T	G
NC_000024.9:g.28578513T>C	28578513	28578513+Y202523	Y202523	T>C	T	C
NC_000024.9:g.13895932A>G	13895932	13895932+Y202524	Y202524	A>G	A	G
NC_000024.9:g.9934065T>C	9934065	9934065+Y203610	Y203610	T>C	T	C
NC_000024.9:g.13592685C>T	13592685	13592685+Y203612	Y203612	C>T	C	T
NC_000024.9:g.22767396A>C	22767396	22767396+Y203613	Y203613	A>C	A	C
NC_000024.9:g.16981456A>G	16981456	16981456+Y203614	Y203614	A>G	A	G
NC_000024.9:g.7924101A>T	7924101	7924101+Y203615	Y203615	A>T	A	T
NC_000024.9:g.23786473G>T	23786473	23786473+Y203616	Y203616	G>T	G	T
NC_000024.9:g.23333989T>C	23333989	23333989+Y203617	Y203617	T>C	T	C
NC_000024.9:g.14012614G>A	14012614	14012614+Y203618	Y203618	G>A	G	A
NC_000024.9:g.9947530C>T	9947530	9947530+Y203619	Y203619	C>T	C	T
NC_000024.9:g.18159414G>A	18159414	18159414+Y203620	Y203620	G>A	G	A
NC_000024.9:g.10064345C>G	10064345	10064345+Y203621	Y203621	C>G	C	G
NC_000024.9:g.21369801G>T	21369801	21369801+Y203622	Y203622	G>T	G	T
NC_000024.9:g.23647174A>G	23647174	23647174+Y203623	Y203623	A>G	A	G
NC_000024.9:g.15794809A>G	15794809	15794809+Y203624	Y203624	A>G	A	G
NC_000024.9:g.6746390T>A	6746390	6746390+Y203625	Y203625	T>A	T	A
NC_000024.9:g.21869621G>A	21869621	21869621+Y203626	Y203626	G>A	G	A
NC_000024.9:g.18424637C>A	18424637	18424637+Y203627	Y203627	C>A	C	A
NC_000024.9:g.21474064C>T	21474064	21474064+Y203628	Y203628	C>T	C	T
NC_000024.9:g.17433411T>A	17433411	17433411+Y203629	Y203629	T>A	T	A
NC_000024.9:g.7431272A>G	7431272	7431272+Y203630	Y203630	A>G	A	G
NC_000024.9:g.14032007G>T	14032007	14032007+Y203631	Y203631	G>T	G	T
NC_000024.9:g.22193508G>A	22193508	22193508+Y203632	Y203632	G>A	G	A
NC_000024.9:g.17620603G>A	17620603	17620603+Y203633	Y203633	G>A	G	A
NC_000024.9:g.8167345C>T	8167345	8167345+Y203634	Y203634	C>T	C	T
NC_000024.9:g.28660167G>A	28660167	28660167+Y203635	Y203635	G>A	G	A
NC_000024.9:g.14795454G>A	14795454	14795454+Y203636	Y203636	G>A	G	A
NC_000024.9:g.18138670C>T	18138670	18138670+Y203637	Y203637	C>T	C	T
NC_000024.9:g.18226733C>A	18226733	18226733+Y203638	Y203638	C>A	C	A
NC_000024.9:g.15857903T>G	15857903	15857903+Y203639	Y203639	T>G	T	G
NC_000024.9:g.23439876C>T	23439876	23439876+Y204792	Y204792	C>T	C	T
NC_000024.9:g.17163854C>G	17163854	17163854+Y204794	Y204794	C>G	C	G
NC_000024.9:g.17219357C>T	17219357	17219357+Y204795	Y204795	C>T	C	T
NC_000024.9:g.17260566A>G	17260566	17260566+Y209287	Y209287	A>G	A	G
NC_000024.9:g.13683828G>A	13683828	13683828+Y210674	Y210674	G>A	G	A
NC_000024.9:g.13212452A>C	13212452	13212452+Y210683	Y210683	A>C	A	C
NC_000024.9:g.13798769A>C	13798769	13798769+Y210684	Y210684	A>C	A	C
NC_000024.9:g.13575385A>T	13575385	13575385+Y210685	Y210685	A>T	A	T
NC_000024.9:g.10009022G>C	10009022	10009022+Y210686	Y210686	G>C	G	C
NC_000024.9:g.9955711T>C	9955711	9955711+Y210687	Y210687	T>C	T	C
NC_000024.9:g.8015889A>T	8015889	8015889+Y210688	Y210688	A>T	A	T
NC_000024.9:g.13577467T>G	13577467	13577467+Y222797	Y222797	T>G	T	G
NC_000024.9:g.6534771C>G	6534771	6534771+Y222798	Y222798	C>G	C	G
NC_000024.9:g.5577041T>C	5577041	5577041+Y222799	Y222799	T>C	T	C
NC_000024.9:g.4256175T>C	4256175	4256175+Y222800	Y222800	T>C	T	C
NC_000024.9:g.4759028T>C	4759028	4759028+Y222801	Y222801	T>C	T	C
NC_000024.9:g.15671238G>A	15671238	15671238+Y225388	Y225388	G>A	G	A
NC_000024.9:g.17900865A>T	17900865	17900865+Y225397	Y225397	A>T	A	T
NC_000024.9:g.13624664G>T	13624664	13624664+Y225955	Y225955	G>T	G	T
NC_000024.9:g.15854636G>A	15854636	15854636+Y225956	Y225956	G>A	G	A
NC_000024.9:g.5019513A>G	5019513	5019513+Y226348	Y226348	A>G	A	G
NC_000024.9:g.21809360G>T	21809360	21809360+Y23183,N1a1a1a2a2~	Y23183,N1a1a1a2a2~	G>T	G	T
NC_000024.9:g.3788778G>T	3788778	3788778+Y2463	Y2463	G>T	G	T
NC_000024.9:g.19672965C>T	19672965	19672965+Y2470_1	Y2470_1	C>T	C	T
NC_000024.9:g.22535839A>G	22535839	22535839+Y2471	Y2471	A>G	A	G
NC_000024.9:g.9492526A>G	9492526	9492526+Y262075	Y262075	A>G	A	G
NC_000024.9:g.14391683T>C	14391683	14391683+Y26466	Y26466	T>C	T	C
NC_000024.9:g.6693411A>G	6693411	6693411+Y26468	Y26468	A>G	A	G
NC_000024.9:g.6805454A>G	6805454	6805454+Y26469	Y26469	A>G	A	G
NC_000024.9:g.7064934C>T	7064934	7064934+Y26470	Y26470	C>T	C	T
NC_000024.9:g.7161780T>A	7161780	7161780+Y26471	Y26471	T>A	T	A
NC_000024.9:g.7425221T>C	7425221	7425221+Y26472	Y26472	T>C	T	C

NC_000024.9:g.7585563T>G	7585563	7585563+Y26473	Y26473	T>G	T	G
NC_000024.9:g.7587352A>G	7587352	7587352+Y26474	Y26474	A>G	A	G
NC_000024.9:g.7829187A>G	7829187	7829187+Y26475	Y26475	A>G	A	G
NC_000024.9:g.7834899G>A	7834899	7834899+Y26476	Y26476	G>A	G	A
NC_000024.9:g.8000496G>A	8000496	8000496+Y26477	Y26477	G>A	G	A
NC_000024.9:g.8037435A>G	8037435	8037435+Y26478	Y26478	A>G	A	G
NC_000024.9:g.8057072C>T	8057072	8057072+Y26479	Y26479	C>T	C	T
NC_000024.9:g.8204517G>C	8204517	8204517+Y26481	Y26481	G>C	G	C
NC_000024.9:g.8434880G>A	8434880	8434880+Y26482	Y26482	G>A	G	A
NC_000024.9:g.8783364G>A	8783364	8783364+Y26483	Y26483	G>A	G	A
NC_000024.9:g.9052803T>G	9052803	9052803+Y26484	Y26484	T>G	T	G
NC_000024.9:g.14006408A>G	14006408	14006408+Y26486	Y26486	A>G	A	G
NC_000024.9:g.14121228A>T	14121228	14121228+Y26487	Y26487	A>T	A	T
NC_000024.9:g.14184570A>T	14184570	14184570+Y26488	Y26488	A>T	A	T
NC_000024.9:g.14248738A>T	14248738	14248738+Y26489	Y26489	A>T	A	T
NC_000024.9:g.14479349C>T	14479349	14479349+Y26490	Y26490	C>T	C	T
NC_000024.9:g.14668160C>G	14668160	14668160+Y26491	Y26491	C>G	C	G
NC_000024.9:g.14758607T>C	14758607	14758607+Y26492	Y26492	T>C	T	C
NC_000024.9:g.14832035T>G	14832035	14832035+Y26493	Y26493	T>G	T	G
NC_000024.9:g.14935273C>A	14935273	14935273+Y26494	Y26494	C>A	C	A
NC_000024.9:g.14935559A>C	14935559	14935559+Y26495	Y26495	A>C	A	C
NC_000024.9:g.14954148T>C	14954148	14954148+Y26496	Y26496	T>C	T	C
NC_000024.9:g.14987418T>G	14987418	14987418+Y26497	Y26497	T>G	T	G
NC_000024.9:g.15151142T>A	15151142	15151142+Y26498	Y26498	T>A	T	A
NC_000024.9:g.15279649C>G	15279649	15279649+Y26499	Y26499	C>G	C	G
NC_000024.9:g.15589897C>A	15589897	15589897+Y26500	Y26500	C>A	C	A
NC_000024.9:g.15979492C>A	15979492	15979492+Y26501	Y26501	C>A	C	A
NC_000024.9:g.16089883T>G	16089883	16089883+Y26502	Y26502	T>G	T	G
NC_000024.9:g.16360320G>A	16360320	16360320+Y26503	Y26503	G>A	G	A
NC_000024.9:g.16422801G>A	16422801	16422801+Y26504	Y26504	G>A	G	A
NC_000024.9:g.16710401A>G	16710401	16710401+Y26505	Y26505	A>G	A	G
NC_000024.9:g.16760808C>T	16760808	16760808+Y26506	Y26506	C>T	C	T
NC_000024.9:g.17488000G>T	17488000	17488000+Y26509	Y26509	G>T	G	T
NC_000024.9:g.17537941G>A	17537941	17537941+Y26510	Y26510	G>A	G	A
NC_000024.9:g.17640062C>T	17640062	17640062+Y26511	Y26511	C>T	C	T
NC_000024.9:g.17800540T>C	17800540	17800540+Y26512	Y26512	T>C	T	C
NC_000024.9:g.17893926G>A	17893926	17893926+Y26513	Y26513	G>A	G	A
NC_000024.9:g.17947856T>G	17947856	17947856+Y26514	Y26514	T>G	T	G
NC_000024.9:g.17947857G>A	17947857	17947857+Y26515	Y26515	G>A	G	A
NC_000024.9:g.18020803G>A	18020803	18020803+Y26516	Y26516	G>A	G	A
NC_000024.9:g.18024321C>T	18024321	18024321+Y26517	Y26517	C>T	C	T
NC_000024.9:g.18649776C>T	18649776	18649776+Y26518	Y26518	C>T	C	T
NC_000024.9:g.18682425G>A	18682425	18682425+Y26519	Y26519	G>A	G	A
NC_000024.9:g.18692055A>G	18692055	18692055+Y26520	Y26520	A>G	A	G
NC_000024.9:g.18758037C>T	18758037	18758037+Y26521	Y26521	C>T	C	T
NC_000024.9:g.18774285T>C	18774285	18774285+Y26523	Y26523	T>C	T	C
NC_000024.9:g.18920333G>C	18920333	18920333+Y26524	Y26524	G>C	G	C
NC_000024.9:g.18960635A>C	18960635	18960635+Y26525	Y26525	A>C	A	C
NC_000024.9:g.19014509C>A	19014509	19014509+Y26526	Y26526	C>A	C	A
NC_000024.9:g.19123301T>C	19123301	19123301+Y26527	Y26527	T>C	T	C
NC_000024.9:g.19208657C>A	19208657	19208657+Y26528	Y26528	C>A	C	A
NC_000024.9:g.19335597A>G	19335597	19335597+Y26529	Y26529	A>G	A	G
NC_000024.9:g.19386428A>G	19386428	19386428+Y26530	Y26530	A>G	A	G
NC_000024.9:g.21230272T>G	21230272	21230272+Y26532	Y26532	T>G	T	G
NC_000024.9:g.21496048C>T	21496048	21496048+Y26533	Y26533	C>T	C	T
NC_000024.9:g.21650104C>T	21650104	21650104+Y26534	Y26534	C>T	C	T
NC_000024.9:g.21882279C>T	21882279	21882279+Y26535	Y26535	C>T	C	T
NC_000024.9:g.22574907C>A	22574907	22574907+Y26536	Y26536	C>A	C	A
NC_000024.9:g.23019788T>C	23019788	23019788+Y26537	Y26537	T>C	T	C
NC_000024.9:g.23084102A>C	23084102	23084102+Y26538	Y26538	A>C	A	C
NC_000024.9:g.23153096A>G	23153096	23153096+Y26539	Y26539	A>G	A	G
NC_000024.9:g.23189337G>T	23189337	23189337+Y26540	Y26540	G>T	G	T
NC_000024.9:g.23189338A>T	23189338	23189338+Y26541	Y26541	A>T	A	T
NC_000024.9:g.23271102A>G	23271102	23271102+Y26542	Y26542	A>G	A	G
NC_000024.9:g.23954485G>T	23954485	23954485+Y26543	Y26543	G>T	G	T
NC_000024.9:g.23954486C>T	23954486	23954486+Y26544	Y26544	C>T	C	T
NC_000024.9:g.28777937C>G	28777937	28777937+Y26546	Y26546	C>G	C	G
NC_000024.9:g.6698908G>C	6698908	6698908+Y26547	Y26547	G>C	G	C
NC_000024.9:g.6835354A>G	6835354	6835354+Y26548	Y26548	A>G	A	G
NC_000024.9:g.6845623C>T	6845623	6845623+Y26549	Y26549	C>T	C	T
NC_000024.9:g.6847451G>A	6847451	6847451+Y26550	Y26550	G>A	G	A
NC_000024.9:g.7672315A>G	7672315	7672315+Y26552	Y26552	A>G	A	G
NC_000024.9:g.7676068A>T	7676068	7676068+Y26553	Y26553	A>T	A	T
NC_000024.9:g.7843564T>C	7843564	7843564+Y26554	Y26554	T>C	T	C
NC_000024.9:g.7929840G>A	7929840	7929840+Y26555	Y26555	G>A	G	A
NC_000024.9:g.7934704T>G	7934704	7934704+Y26556	Y26556	T>G	T	G
NC_000024.9:g.8008399T>G	8008399	8008399+Y26557	Y26557	T>G	T	G
NC_000024.9:g.8047054T>G	8047054	8047054+Y26558	Y26558	T>G	T	G
NC_000024.9:g.8086114G>T	8086114	8086114+Y26559	Y26559	G>T	G	T
NC_000024.9:g.8105751A>T	8105751	8105751+Y26560	Y26560	A>T	A	T
NC_000024.9:g.8306124A>G	8306124	8306124+Y26561	Y26561	A>G	A	G
NC_000024.9:g.8353233G>A	8353233	8353233+Y26562	Y26562	G>A	G	A
NC_000024.9:g.8445638G>C	8445638	8445638+Y26563	Y26563	G>C	G	C
NC_000024.9:g.8581071T>C	8581071	8581071+Y26564	Y26564	T>C	T	C
NC_000024.9:g.8601165C>T	8601165	8601165+Y26565	Y26565	C>T	C	T
NC_000024.9:g.8704106C>G	8704106	8704106+Y26566	Y26566	C>G	C	G
NC_000024.9:g.9012775A>C	9012775	9012775+Y26567	Y26567	A>C	A	C
NC_000024.9:g.9168921A>G	9168921	9168921+Y26568	Y26568	A>G	A	G
NC_000024.9:g.9440234A>G	9440234	9440234+Y26569	Y26569	A>G	A	G
NC_000024.9:g.9464721T>C	9464721	9464721+Y26570	Y26570	T>C	T	C
NC_000024.9:g.9898699A>G	9898699	9898699+Y26572	Y26572	A>G	A	G
NC_000024.9:g.13880013T>C	13880013	13880013+Y26573	Y26573	T>C	T	C
NC_000024.9:g.14003426A>G	14003426	14003426+Y26574	Y26574	A>G	A	G
NC_000024.9:g.14054523A>G	14054523	14054523+Y26575	Y26575	A>G	A	G
NC_000024.9:g.14079211G>A	14079211	14079211+Y26576	Y26576	G>A	G	A
NC_000024.9:g.14169818A>T	14169818	14169818+Y26577	Y26577	A>T	A	T
NC_000024.9:g.14258873C>T	14258873	14258873+Y26578	Y26578	C>T	C	T
NC_000024.9:g.14587383C>T	14587383	14587383+Y26580	Y26580	C>T	C	T
NC_000024.9:g.15330838A>G	15330838	15330838+Y26581	Y26581	A>G	A	G
NC_000024.9:g.15345215C>G	15345215	15345215+Y26582	Y26582	C>G	C	G
NC_000024.9:g.15391576G>C	15391576	15391576+Y26583	Y26583	G>C	G	C
NC_000024.9:g.15746337C>T	15746337	15746337+Y26584	Y26584	C>T	C	T
NC_000024.9:g.15798888T>C	15798888	15798888+Y26585	Y26585	T>C	T	C
NC_000024.9:g.15869065T>A	15869065	15869065+Y26586	Y26586	T>A	T	A

NC_000024.9:g.16054765A>G	16054765	16054765+Y26587	Y26587	A>G	A	G
NC_000024.9:g.16680268G>A	16680268	16680268+Y26588	Y26588	G>A	G	A
NC_000024.9:g.16696452C>A	16696452	16696452+Y26589	Y26589	C>A	C	A
NC_000024.9:g.17104392G>A	17104392	17104392+Y26590	Y26590	G>A	G	A
NC_000024.9:g.17800104C>T	17800104	17800104+Y26592	Y26592	C>T	C	T
NC_000024.9:g.17866611T>A	17866611	17866611+Y26593	Y26593	T>A	T	A
NC_000024.9:g.17909830A>T	17909830	17909830+Y26594	Y26594	A>T	A	T
NC_000024.9:g.18122131C>T	18122131	18122131+Y26595	Y26595	C>T	C	T
NC_000024.9:g.18149024C>G	18149024	18149024+Y26596	Y26596	C>G	C	G
NC_000024.9:g.18715192G>T	18715192	18715192+Y26597	Y26597	G>T	G	T
NC_000024.9:g.19127299A>C	19127299	19127299+Y26598	Y26598	A>C	A	C
NC_000024.9:g.19452928T>C	19452928	19452928+Y26599	Y26599	T>C	T	C
NC_000024.9:g.22123960C>G	22123960	22123960+Y26601	Y26601	C>G	C	G
NC_000024.9:g.22208111A>G	22208111	22208111+Y26602	Y26602	A>G	A	G
NC_000024.9:g.22649468A>C	22649468	22649468+Y26603	Y26603	A>C	A	C
NC_000024.9:g.22649933C>T	22649933	22649933+Y26604	Y26604	C>T	C	T
NC_000024.9:g.22914954G>C	22914954	22914954+Y26605	Y26605	G>C	G	C
NC_000024.9:g.23207629G>A	23207629	23207629+Y26606	Y26606	G>A	G	A
NC_000024.9:g.23857512G>A	23857512	23857512+Y26607	Y26607	G>A	G	A
NC_000024.9:g.23985823G>T	23985823	23985823+Y26608	Y26608	G>T	G	T
NC_000024.9:g.22217066G>A	22217066	22217066+Y26611	Y26611	G>A	G	A
NC_000024.9:g.2803666C>G	2803666	2803666+Y26612	Y26612	C>G	C	G
NC_000024.9:g.8485713A>G	8485713	8485713+Y26613	Y26613	A>G	A	G
NC_000024.9:g.14811037C>G	14811037	14811037+Y26669	Y26669	C>G	C	G
NC_000024.9:g.28543275A>G	28543275	28543275+Y2710	Y2710	A>G	A	G
NC_000024.9:g.21966926T>C	21966926	21966926+Y272141	Y272141	T>C	T	C
NC_000024.9:g.21966924T>C	21966924	21966924+Y272142	Y272142	T>C	T	C
NC_000024.9:g.4229024G>A	4229024	4229024+Y275157	Y275157	G>A	G	A
NC_000024.9:g.14344884A>C	14344884	14344884+Y27994,Q1b1a1a1n~	Y27994,Q1b1a1a1n~	A>C	A	C
NC_000024.9:g.22089472T>C	22089472	22089472+Y27997	Y27997	T>C	T	C
NC_000024.9:g.22173485G>A	22173485	22173485+Y27998	Y27998	G>A	G	A
NC_000024.9:g.3318511T>A	3318511	3318511+Y27999	Y27999	T>A	T	A
NC_000024.9:g.7769790C>T	7769790	7769790+Y28003	Y28003	C>T	C	T
NC_000024.9:g.7949507C>A	7949507	7949507+Y28004	Y28004	C>A	C	A
NC_000024.9:g.17966277G>C	17966277	17966277+Y28005	Y28005	G>C	G	C
NC_000024.9:g.8238657C>T	8238657	8238657+Y28006	Y28006	C>T	C	T
NC_000024.9:g.7317737C>A	7317737	7317737+Y28007	Y28007	C>A	C	A
NC_000024.9:g.14219521G>C	14219521	14219521+Y28008	Y28008	G>C	G	C
NC_000024.9:g.14485300G>A	14485300	14485300+Y28009	Y28009	G>A	G	A
NC_000024.9:g.18095549G>T	18095549	18095549+Y28010	Y28010	G>T	G	T
NC_000024.9:g.18762612G>A	18762612	18762612+Y28011	Y28011	G>A	G	A
NC_000024.9:g.18906014A>C	18906014	18906014+Y28012	Y28012	A>C	A	C
NC_000024.9:g.14991028G>A	14991028	14991028+Y28013	Y28013	G>A	G	A
NC_000024.9:g.17945760C>T	17945760	17945760+Y28014	Y28014	C>T	C	T
NC_000024.9:g.18715085A>C	18715085	18715085+Y28015	Y28015	A>C	A	C
NC_000024.9:g.17096544G>T	17096544	17096544+Y28018	Y28018	G>T	G	T
NC_000024.9:g.16673936A>G	16673936	16673936+Y28019	Y28019	A>G	A	G
NC_000024.9:g.16552828C>T	16552828	16552828+Y28020	Y28020	C>T	C	T
NC_000024.9:g.5897821T>C	5897821	5897821+Y28021	Y28021	T>C	T	C
NC_000024.9:g.6883704A>C	6883704	6883704+Y28022	Y28022	A>C	A	C
NC_000024.9:g.9401763C>G	9401763	9401763+Y28023	Y28023	C>G	C	G
NC_000024.9:g.8345999G>C	8345999	8345999+Y28024	Y28024	G>C	G	C
NC_000024.9:g.9832570G>A	9832570	9832570+Y28025	Y28025	G>A	G	A
NC_000024.9:g.13835435G>T	13835435	13835435+Y28026	Y28026	G>T	G	T
NC_000024.9:g.15733103C>A	15733103	15733103+Y28027	Y28027	C>A	C	A
NC_000024.9:g.14479346G>C	14479346	14479346+Y28028	Y28028	G>C	G	C
NC_000024.9:g.17632065A>G	17632065	17632065+Y28029	Y28029	A>G	A	G
NC_000024.9:g.17466911A>G	17466911	17466911+Y28030	Y28030	A>G	A	G
NC_000024.9:g.18553538G>T	18553538	18553538+Y28031	Y28031	G>T	G	T
NC_000024.9:g.18648328T>C	18648328	18648328+Y28032	Y28032	T>C	T	C
NC_000024.9:g.18626481T>C	18626481	18626481+Y28033	Y28033	T>C	T	C
NC_000024.9:g.21750521A>C	21750521	21750521+Y28034	Y28034	A>C	A	C
NC_000024.9:g.19441882A>C	19441882	19441882+Y28035	Y28035	A>C	A	C
NC_000024.9:g.21823260G>A	21823260	21823260+Y28036	Y28036	G>A	G	A
NC_000024.9:g.21227492A>G	21227492	21227492+Y28037	Y28037	A>G	A	G
NC_000024.9:g.23883540T>A	23883540	23883540+Y28039	Y28039	T>A	T	A
NC_000024.9:g.28651124C>A	28651124	28651124+Y28040	Y28040	C>A	C	A
NC_000024.9:g.4431029A>G	4431029	4431029+Y28042	Y28042	A>G	A	G
NC_000024.9:g.9385266C>G	9385266	9385266+Y2805	Y2805	C>G	C	G
NC_000024.9:g.22480356C>T	22480356	22480356+Y2827	Y2827	C>T	C	T
NC_000024.9:g.23730136G>C	23730136	23730136+Y2829	Y2829	G>C	G	C
NC_000024.9:g.14820439A>G	14820439	14820439+Y3_SA02	Y3_SA02	A>G	A	G
NC_000024.9:g.15019808C>G	15019808	15019808+Y3_SA03.1	Y3_SA03.1	C>G	C	G
NC_000024.9:g.15019822A>G	15019822	15019822+Y3_SA03.2	Y3_SA03.2	A>G	A	G
NC_000024.9:g.15974563A>T	15974563	15974563+Y3_SA04	Y3_SA04	A>T	A	T
NC_000024.9:g.28487244A>G	28487244	28487244+Y300457	Y300457	A>G	A	G
NC_000024.9:g.4989202T>C	4989202	4989202+Y300544	Y300544	T>C	T	C
NC_000024.9:g.16685543T>G	16685543	16685543+Y300722	Y300722	T>G	T	G
NC_000024.9:g.13549191G>T	13549191	13549191+Y300723	Y300723	G>T	G	T
NC_000024.9:g.19445823C>T	19445823	19445823+Y30594,I1a1b1a1e2d2~	Y30594,I1a1b1a1e2d2~	C>T	C	T
NC_000024.9:g.22690907G>C	22690907	22690907+Y32752	Y32752	G>C	G	C
NC_000024.9:g.21626336G>A	21626336	21626336+Y34226,R1a1a1b2a1a2c2b1~	Y34226,R1a1a1b2a1a2c2b1~	G>A	G	A
NC_000024.9:g.5179125A>C	5179125	5179125+Y3438	Y3438	A>C	A	C
NC_000024.9:g.6082633G>A	6082633	6082633+Y3439	Y3439	G>A	G	A
NC_000024.9:g.20802686T>A	20802686	20802686+Y3440	Y3440	T>A	T	A
NC_000024.9:g.9401357T>C	9401357	9401357+Y38133	Y38133	T>C	T	C
NC_000024.9:g.16533386C>A	16533386	16533386+Y38271	Y38271	C>A	C	A
NC_000024.9:g.9640222G>A	9640222	9640222+Y38274	Y38274	G>A	G	A
NC_000024.9:g.23018415A>T	23018415	23018415+Y38275	Y38275	A>T	A	T
NC_000024.9:g.16642510G>C	16642510	16642510+Y3889	Y3889	G>C	G	C
NC_000024.9:g.22942417C>T	22942417	22942417+Y3907	Y3907	C>T	C	T
NC_000024.9:g.8479549C>T	8479549	8479549+Y4291	Y4291	C>T	C	T
NC_000024.9:g.6884771G>A	6884771	6884771+Y45410	Y45410	G>A	G	A
NC_000024.9:g.13961083C>T	13961083	13961083+Y5_CO02	Y5_CO02	C>T	C	T
NC_000024.9:g.21924485C>T	21924485	21924485+Y5_CO03	Y5_CO03	C>T	C	T
NC_000024.9:g.8032185C>G	8032185	8032185+Y5_CO11	Y5_CO11	C>G	C	G
NC_000024.9:g.13963082C>G	13963082	13963082+Y5_CO12	Y5_CO12	C>G	C	G
NC_000024.9:g.5699467C>T	5699467	5699467+Y569	Y569	C>T	C	T
NC_000024.9:g.9140911G>T	9140911	9140911+Y570	Y570	G>T	G	T
NC_000024.9:g.17270147G>T	17270147	17270147+Y573	Y573	G>T	G	T
NC_000024.9:g.3335075T>C	3335075	3335075+Y579	Y579	T>C	T	C
NC_000024.9:g.3436379G>A	3436379	3436379+Y581	Y581	G>A	G	A
NC_000024.9:g.3502887C>T	3502887	3502887+Y582	Y582	C>T	C	T
NC_000024.9:g.3907849A>G	3907849	3907849+Y583	Y583	A>G	A	G

NC_000024.9:g.4084499C>A	4084499	4084499+Y584	Y584	C>A	C	A
NC_000024.9:g.4093024A>G	4093024	4093024+Y585	Y585	A>G	A	G
NC_000024.9:g.4282118A>G	4282118	4282118+Y588	Y588	A>G	A	G
NC_000024.9:g.4287008C>T	4287008	4287008+Y589	Y589	C>T	C	T
NC_000024.9:g.4301142G>T	4301142	4301142+Y590	Y590	G>T	G	T
NC_000024.9:g.4310262T>G	4310262	4310262+Y591	Y591	T>G	T	G
NC_000024.9:g.4455708G>T	4455708	4455708+Y592	Y592	G>T	G	T
NC_000024.9:g.4731050G>A	4731050	4731050+Y594	Y594	G>A	G	A
NC_000024.9:g.4755531G>A	4755531	4755531+Y595	Y595	G>A	G	A
NC_000024.9:g.4843075G>A	4843075	4843075+Y596	Y596	G>A	G	A
NC_000024.9:g.23857963C>T	23857963	23857963+Y59605	Y59605	C>T	C	T
NC_000024.9:g.4918155T>C	4918155	4918155+Y597	Y597	T>C	T	C
NC_000024.9:g.4935693G>A	4935693	4935693+Y598	Y598	G>A	G	A
NC_000024.9:g.5054560C>A	5054560	5054560+Y599	Y599	C>A	C	A
NC_000024.9:g.28589665C>T	28589665	28589665+Y59934	Y59934	C>T	C	T
NC_000024.9:g.5068708G>A	5068708	5068708+Y600	Y600	G>A	G	A
NC_000024.9:g.5709986T>C	5709986	5709986+Y602	Y602	T>C	T	C
NC_000024.9:g.2778186T>C	2778186	2778186+Y60256	Y60256	T>C	T	C
NC_000024.9:g.6419468T>A	6419468	6419468+Y606	Y606	T>A	T	A
NC_000024.9:g.6484686C>T	6484686	6484686+Y607	Y607	C>T	C	T
NC_000024.9:g.6495129C>T	6495129	6495129+Y608	Y608	C>T	C	T
NC_000024.9:g.7895292A>G	7895292	7895292+Y612	Y612	A>G	A	G
NC_000024.9:g.19500653A>C	19500653	19500653+Y6164	Y6164	A>C	A	C
NC_000024.9:g.7354925C>T	7354925	7354925+Y61936	Y61936	C>T	C	T
NC_000024.9:g.13907291T>C	13907291	13907291+Y623	Y623	T>C	T	C
NC_000024.9:g.13996143G>A	13996143	13996143+Y625	Y625	G>A	G	A
NC_000024.9:g.14114634A>T	14114634	14114634+Y626	Y626	A>T	A	T
NC_000024.9:g.14423729T>C	14423729	14423729+Y630	Y630	T>C	T	C
NC_000024.9:g.16235031G>A	16235031	16235031+Y640	Y640	G>A	G	A
NC_000024.9:g.22462369T>G	22462369	22462369+Y649	Y649	T>G	T	G
NC_000024.9:g.22517174G>T	22517174	22517174+Y651	Y651	G>T	G	T
NC_000024.9:g.22542768A>G	22542768	22542768+Y652	Y652	A>G	A	G
NC_000024.9:g.9850809A>C	9850809	9850809+Y65300	Y65300	A>C	A	C
NC_000024.9:g.23212590C>G	23212590	23212590+Y655	Y655	C>G	C	G
NC_000024.9:g.23655048A>T	23655048	23655048+Y657	Y657	A>T	A	T
NC_000024.9:g.28206881C>T	28206881	28206881+Y660	Y660	C>T	C	T
NC_000024.9:g.28518893T>C	28518893	28518893+Y661	Y661	T>C	T	C
NC_000024.9:g.19033874T>C	19033874	19033874+Y666	Y666	T>C	T	C
NC_000024.9:g.28625839C>T	28625839	28625839+Y669	Y669	C>T	C	T
NC_000024.9:g.24427412T>A	24427412	24427412+Y670	Y670	T>A	T	A
NC_000024.9:g.5850288T>C	5850288	5850288+Y672	Y672	T>C	T	C
NC_000024.9:g.5951290T>G	5951290	5951290+Y673	Y673	T>G	T	G
NC_000024.9:g.14112192C>T	14112192	14112192+Y675	Y675	C>T	C	T
NC_000024.9:g.9023670A>T	9023670	9023670+Y6776	Y6776	A>T	A	T
NC_000024.9:g.21830160A>T	21830160	21830160+Y679	Y679	A>T	A	T
NC_000024.9:g.5207581G>A	5207581	5207581+Y684	Y684	G>A	G	A
NC_000024.9:g.23655075G>T	23655075	23655075+Y6847	Y6847	G>T	G	T
NC_000024.9:g.7162185G>A	7162185	7162185+Y685	Y685	G>A	G	A
NC_000024.9:g.15240761G>A	15240761	15240761+Y687	Y687	G>A	G	A
NC_000024.9:g.22444625G>T	22444625	22444625+Y688	Y688	G>T	G	T
NC_000024.9:g.28556640A>G	28556640	28556640+Y689	Y689	A>G	A	G
NC_000024.9:g.17229332A>G	17229332	17229332+Y690	Y690	A>G	A	G
NC_000024.9:g.28801154G>A	28801154	28801154+Y707	Y707	G>A	G	A
NC_000024.9:g.16960342C>A	16960342	16960342+Y70800	Y70800	C>A	C	A
NC_000024.9:g.3550871A>T	3550871	3550871+Y715	Y715	A>T	A	T
NC_000024.9:g.3847063A>T	3847063	3847063+Y716	Y716	A>T	A	T
NC_000024.9:g.4060167T>C	4060167	4060167+Y717	Y717	T>C	T	C
NC_000024.9:g.4114714G>A	4114714	4114714+Y718	Y718	G>A	G	A
NC_000024.9:g.17514794T>A	17514794	17514794+Y71815	Y71815	T>A	T	A
NC_000024.9:g.4249577T>C	4249577	4249577+Y719	Y719	T>C	T	C
NC_000024.9:g.4901135C>T	4901135	4901135+Y721	Y721	C>T	C	T
NC_000024.9:g.5525434A>G	5525434	5525434+Y724	Y724	A>G	A	G
NC_000024.9:g.5793300C>T	5793300	5793300+Y725	Y725	C>T	C	T
NC_000024.9:g.5823439G>A	5823439	5823439+Y726	Y726	G>A	G	A
NC_000024.9:g.6046598G>A	6046598	6046598+Y727	Y727	G>A	G	A
NC_000024.9:g.8967189C>G	8967189	8967189+Y730	Y730	C>G	C	G
NC_000024.9:g.9502942G>A	9502942	9502942+Y731	Y731	G>A	G	A
NC_000024.9:g.13604783G>A	13604783	13604783+Y736	Y736	G>A	G	A
NC_000024.9:g.4717246G>C	4717246	4717246+Y738	Y738	G>C	G	C
NC_000024.9:g.4756387G>T	4756387	4756387+Y740	Y740	G>T	G	T
NC_000024.9:g.5504134G>T	5504134	5504134+Y741	Y741	G>T	G	T
NC_000024.9:g.5677171T>C	5677171	5677171+Y743	Y743	T>C	T	C
NC_000024.9:g.22262730T>A	22262730	22262730+Y748	Y748	T>A	T	A
NC_000024.9:g.22460495G>T	22460495	22460495+Y751	Y751	G>T	G	T
NC_000024.9:g.22478077T>A	22478077	22478077+Y752	Y752	T>A	T	A
NC_000024.9:g.6075983A>G	6075983	6075983+Y756	Y756	A>G	A	G
NC_000024.9:g.5983319A>G	5983319	5983319+Y757	Y757	A>G	A	G
NC_000024.9:g.6013204A>G	6013204	6013204+Y758	Y758	A>G	A	G
NC_000024.9:g.6013234C>T	6013234	6013234+Y759	Y759	C>T	C	T
NC_000024.9:g.3105982C>T	3105982	3105982+Y764	Y764	C>T	C	T
NC_000024.9:g.6601746G>A	6601746	6601746+Y765	Y765	G>A	G	A
NC_000024.9:g.13305369G>T	13305369	13305369+Y766	Y766	G>T	G	T
NC_000024.9:g.4129985C>A	4129985	4129985+Y770	Y770	C>A	C	A
NC_000024.9:g.4590011A>G	4590011	4590011+Y771	Y771	A>G	A	G
NC_000024.9:g.13583132C>T	13583132	13583132+Y772	Y772	C>T	C	T
NC_000024.9:g.22613361C>A	22613361	22613361+Y773,Q1b1a1a	Y773,Q1b1a1a	C>A	C	A
NC_000024.9:g.3123979G>C	3123979	3123979+Y774	Y774	G>C	G	C
NC_000024.9:g.21546493G>A	21546493	21546493+Y775,Q1b1~	Y775,Q1b1~	G>A	G	A
NC_000024.9:g.5810134C>A	5810134	5810134+Y780	Y780	C>A	C	A
NC_000024.9:g.3781049G>A	3781049	3781049+Y784	Y784	G>A	G	A
NC_000024.9:g.17641227A>C	17641227	17641227+Y787,Q1b1a1a1j	Y787,Q1b1a1a1j	A>C	A	C
NC_000024.9:g.4952911T>A	4952911	4952911+Y790	Y790	T>A	T	A
NC_000024.9:g.13909667G>A	13909667	13909667+Y791	Y791	G>A	G	A
NC_000024.9:g.3093145C>G	3093145	3093145+Y796	Y796	C>G	C	G
NC_000024.9:g.3631028C>T	3631028	3631028+Y797	Y797	C>T	C	T
NC_000024.9:g.3716083G>A	3716083	3716083+Y799	Y799	G>A	G	A
NC_000024.9:g.3833916A>C	3833916	3833916+Y801	Y801	A>C	A	C
NC_000024.9:g.5224100G>A	5224100	5224100+Y802	Y802	G>A	G	A
NC_000024.9:g.6473294G>C	6473294	6473294+Y803	Y803	G>C	G	C
NC_000024.9:g.6593634G>A	6593634	6593634+Y804	Y804	G>A	G	A
NC_000024.9:g.6618898A>G	6618898	6618898+Y805	Y805	A>G	A	G
NC_000024.9:g.9433619A>C	9433619	9433619+Y810	Y810	A>C	A	C
NC_000024.9:g.13589800G>C	13589800	13589800+Y811	Y811	G>C	G	C
NC_000024.9:g.13603541T>C	13603541	13603541+Y812	Y812	T>C	T	C

NC_000024.9:g.13604724G>A	13604724	13604724+Y813	Y813	G>A	G	A
NC_000024.9:g.13892062A>C	13892062	13892062+Y814	Y814	A>C	A	C
NC_000024.9:g.13914345C>T	13914345	13914345+Y815	Y815	C>T	C	T
NC_000024.9:g.22209898A>C	22209898	22209898+Y837	Y837	A>C	A	C
NC_000024.9:g.22458094G>A	22458094	22458094+Y839	Y839	G>A	G	A
NC_000024.9:g.22464511T>A	22464511	22464511+Y840	Y840	T>A	T	A
NC_000024.9:g.22464532A>T	22464532	22464532+Y841	Y841	A>T	A	T
NC_000024.9:g.23229428A>G	23229428	23229428+Y848	Y848	A>G	A	G
NC_000024.9:g.28541932C>T	28541932	28541932+Y850	Y850	C>T	C	T
NC_000024.9:g.4530011T>A	4530011	4530011+Y852	Y852	T>A	T	A
NC_000024.9:g.4584826G>A	4584826	4584826+Y853	Y853	G>A	G	A
NC_000024.9:g.3428815T>C	3428815	3428815+Y854	Y854	T>C	T	C
NC_000024.9:g.22522865G>A	22522865	22522865+Y857	Y857	G>A	G	A
NC_000024.9:g.3912113A>C	3912113	3912113+Y859	Y859	A>C	A	C
NC_000024.9:g.6460938A>G	6460938	6460938+Y860	Y860	A>G	A	G
NC_000024.9:g.4490558A>G	4490558	4490558+Y863	Y863	A>G	A	G
NC_000024.9:g.16172112G>T	16172112	16172112+Y864	Y864	G>T	G	T
NC_000024.9:g.28638284G>T	28638284	28638284+Y871	Y871	G>T	G	T
NC_000024.9:g.17039219G>T	17039219	17039219+Y98020	Y98020	G>T	G	T
NC_000024.9:g.17297035G>A	17297035	17297035+Y98705,O2a2b1a1a1a1a1b1a1a2	Y98705,O2a2b1a1a1a1a1b1a1a2	G>A	G	A
NC_000024.9:g.15073918G>A	15073918	15073918+YFS402625.1^,Z16183.1^,Z19615.1^,E1b1a1a1a1c2c3a3	YFS402625.1^,Z16183.1^,Z19615.1^,E1b1a1a1a1c2c3a3	G>A	G	A
NC_000024.9:g.7535352C>G	7535352	7535352+YP4673	YP4673	C>G	C	G
NC_000024.9:g.7772807G>A	7772807	7772807+YP4674	YP4674	G>A	G	A
NC_000024.9:g.8395921C>T	8395921	8395921+YP4675	YP4675	C>T	C	T
NC_000024.9:g.8509132T>C	8509132	8509132+YP4676	YP4676	T>C	T	C
NC_000024.9:g.9648650T>C	9648650	9648650+YP4677	YP4677	T>C	T	C
NC_000024.9:g.9085222C>T	9085222	9085222+YP4678	YP4678	C>T	C	T
NC_000024.9:g.13662258G>A	13662258	13662258+YP4679	YP4679	G>A	G	A
NC_000024.9:g.14403746A>G	14403746	14403746+YP4680	YP4680	A>G	A	G
NC_000024.9:g.18611197C>A	18611197	18611197+YP4681	YP4681	C>A	C	A
NC_000024.9:g.21230407G>T	21230407	21230407+YP4682	YP4682	G>T	G	T
NC_000024.9:g.21416122G>C	21416122	21416122+YP4683	YP4683	G>C	G	C
NC_000024.9:g.21771726A>G	21771726	21771726+YP4684	YP4684	A>G	A	G
NC_000024.9:g.22540105T>C	22540105	22540105+YP4686	YP4686	T>C	T	C
NC_000024.9:g.23451359A>G	23451359	23451359+YP4687	YP4687	A>G	A	G
NC_000024.9:g.23767128T>C	23767128	23767128+YP4688	YP4688	T>C	T	C
NC_000024.9:g.23854854C>T	23854854	23854854+YP4689	YP4689	C>T	C	T
NC_000024.9:g.8657351G>A	8657351	8657351+YP4716	YP4716	G>A	G	A
NC_000024.9:g.21238983G>C	21238983	21238983+YP4719	YP4719	G>C	G	C
NC_000024.9:g.23568943G>T	23568943	23568943+YP4720	YP4720	G>T	G	T
NC_000024.9:g.24437890G>C	24437890	24437890+YP4721	YP4721	G>C	G	C
NC_000024.9:g.16324994A>G	16324994	16324994+YP4722	YP4722	A>G	A	G
NC_000024.9:g.16960587A>G	16960587	16960587+YP4723	YP4723	A>G	A	G
NC_000024.9:g.18128727G>A	18128727	18128727+YP4724	YP4724	G>A	G	A
NC_000024.9:g.4712672A>G	4712672	4712672+YP5735	YP5735	A>G	A	G
NC_000024.9:g.5907508T>G	5907508	5907508+YP5736	YP5736	T>G	T	G
NC_000024.9:g.10075489T>C	10075489	10075489+YP5737	YP5737	T>C	T	C
NC_000024.9:g.14447092G>A	14447092	14447092+YP5739	YP5739	G>A	G	A
NC_000024.9:g.14555799T>G	14555799	14555799+YP5740	YP5740	T>G	T	G
NC_000024.9:g.13814742C>A	13814742	13814742+YP5741	YP5741	C>A	C	A
NC_000024.9:g.14813764A>C	14813764	14813764+YP5742	YP5742	A>C	A	C
NC_000024.9:g.15282361G>T	15282361	15282361+YP5743	YP5743	G>T	G	T
NC_000024.9:g.15517155C>T	15517155	15517155+YP5744	YP5744	C>T	C	T
NC_000024.9:g.16253334G>A	16253334	16253334+YP5745	YP5745	G>A	G	A
NC_000024.9:g.16519247T>G	16519247	16519247+YP5746	YP5746	T>G	T	G
NC_000024.9:g.18256335C>T	18256335	18256335+YP5747	YP5747	C>T	C	T
NC_000024.9:g.19247021C>T	19247021	19247021+YP5748	YP5748	C>T	C	T
NC_000024.9:g.21632648T>C	21632648	21632648+YP5749	YP5749	T>C	T	C
NC_000024.9:g.21639619C>T	21639619	21639619+YP5750	YP5750	C>T	C	T
NC_000024.9:g.22713089T>C	22713089	22713089+YP5751	YP5751	T>C	T	C
NC_000024.9:g.23049558G>A	23049558	23049558+YP5753	YP5753	G>A	G	A
NC_000024.9:g.16273119C>G	16273119	16273119+YP5754	YP5754	C>G	C	G
NC_000024.9:g.16622405C>A	16622405	16622405+YP5755	YP5755	C>A	C	A
NC_000024.9:g.18395083T>C	18395083	18395083+YP5756	YP5756	T>C	T	C
NC_000024.9:g.19057775A>G	19057775	19057775+YP5757	YP5757	A>G	A	G
NC_000024.9:g.22018844T>G	22018844	22018844+YP5758	YP5758	T>G	T	G
NC_000024.9:g.22106084C>T	22106084	22106084+YP5759	YP5759	C>T	C	T
NC_000024.9:g.23775134G>A	23775134	23775134+YP5760	YP5760	G>A	G	A
NC_000024.9:g.8704117C>T	8704117	8704117+YP5762	YP5762	C>T	C	T
NC_000024.9:g.8789011G>A	8789011	8789011+YP5763	YP5763	G>A	G	A
NC_000024.9:g.16738262C>T	16738262	16738262+YP5764	YP5764	C>T	C	T
NC_000024.9:g.18685720T>A	18685720	18685720+YP5765	YP5765	T>A	T	A
NC_000024.9:g.18427359C>G	18427359	18427359+YP5766	YP5766	C>G	C	G
NC_000024.9:g.6422924C>T	6422924	6422924+YP5767	YP5767	C>T	C	T
NC_000024.9:g.6702974G>A	6702974	6702974+YP5768	YP5768	G>A	G	A
NC_000024.9:g.28543031C>T	28543031	28543031+YP5769	YP5769	C>T	C	T
NC_000024.9:g.28667072G>A	28667072	28667072+YP5770	YP5770	G>A	G	A
NC_000024.9:g.9895907C>A	9895907	9895907+YP5772	YP5772	C>A	C	A
NC_000024.9:g.13833116G>A	13833116	13833116+YP5773	YP5773	G>A	G	A
NC_000024.9:g.6724062C>T	6724062	6724062+YP6104	YP6104	C>T	C	T
NC_000024.9:g.7530321C>T	7530321	7530321+YP6114	YP6114	C>T	C	T
NC_000024.9:g.8975394T>A	8975394	8975394+YP6127	YP6127	T>A	T	A
NC_000024.9:g.16526605T>A	16526605	16526605+YP6154	YP6154	T>A	T	A
NC_000024.9:g.18767074G>A	18767074	18767074+YP6161	YP6161	G>A	G	A
NC_000024.9:g.13649339G>A	13649339	13649339+YP924	YP924	G>A	G	A
NC_000024.9:g.17754902C>T	17754902	17754902+Z12675.2,C1b1a1a1a1a1a1a1a2~	Z12675.2,C1b1a1a1a1a1a1a1a2~	C>T	C	T
NC_000024.9:g.21383955G>A	21383955	21383955+Z15311,E1a2a1b1	Z15311,E1a2a1b1	G>A	G	A
NC_000024.9:g.22074644G>A	22074644	22074644+Z15526,E1a	Z15526,E1a	G>A	G	A
NC_000024.9:g.17940792T>C	17940792	17940792+Z17157,D1	Z17157,D1	T>C	T	C
NC_000024.9:g.16542978G>A	16542978	16542978+Z18896,H2a	Z18896,H2a	G>A	G	A
NC_000024.9:g.14155658A>G	14155658	14155658+Z19301,Q1b1a1a1i	Z19301,Q1b1a1a1i	A>G	A	G
NC_000024.9:g.15266638T>C	15266638	15266638+Z19302,Q1b1a1a1i	Z19302,Q1b1a1a1i	T>C	T	C
NC_000024.9:g.15490857C>T	15490857	15490857+Z19304^,Q1b1a1a1i	Z19304^,Q1b1a1a1i	C>T	C	T
NC_000024.9:g.17494745A>T	17494745	17494745+Z19306^,Q1b1a1a1i	Z19306^,Q1b1a1a1i	A>T	A	T
NC_000024.9:g.17779948C>T	17779948	17779948+Z19307,Q1b1a1a1i	Z19307,Q1b1a1a1i	C>T	C	T
NC_000024.9:g.17941194T>C	17941194	17941194+Z19308,Q1b1a1a1i	Z19308,Q1b1a1a1i	T>C	T	C
NC_000024.9:g.18788786C>T	18788786	18788786+Z19311,Q1b1a1a1i	Z19311,Q1b1a1a1i	C>T	C	T
NC_000024.9:g.21864909A>G	21864909	21864909+Z19312,Q1b1a1a1i	Z19312,Q1b1a1a1i	A>G	A	G
NC_000024.9:g.23097167A>C	23097167	23097167+Z19315,Q1b1a1a1i	Z19315,Q1b1a1a1i	A>C	A	C
NC_000024.9:g.23372539G>C	23372539	23372539+Z19316,Q1b1a1a1i	Z19316,Q1b1a1a1i	G>C	G	C
NC_000024.9:g.16750091T>C	16750091	16750091+Z19318,Q1b1a1a1i1	Z19318,Q1b1a1a1i1	T>C	T	C
NC_000024.9:g.23028397C>T	23028397	23028397+Z19319,Q1b1a1a1i1	Z19319,Q1b1a1a1i1	C>T	C	T
NC_000024.9:g.17287969C>T	17287969	17287969+Z19329,Q1b1a1a1i1a1	Z19329,Q1b1a1a1i1a1	C>T	C	T
NC_000024.9:g.19403841A>G	19403841	19403841+Z19330,Q1b1a1a1i1a1	Z19330,Q1b1a1a1i1a1	A>G	A	G

NC_000024.9:g.21617186G>A	21617186	21617186+Z19331,Q1b1a1a1i1a1	Z19331,Q1b1a1a1i1a1	G>A	G	A
NC_000024.9:g.23162545C>T	23162545	23162545+Z19333,Q1b1a1a1i1a1	Z19333,Q1b1a1a1i1a1	C>T	C	T
NC_000024.9:g.16659052C>G	16659052	16659052+Z19343,Q1b1a1a1i1a1a	Z19343,Q1b1a1a1i1a1a	C>G	C	G
NC_000024.9:g.17448026G>A	17448026	17448026+Z19344,Q1b1a1a1i1a1a	Z19344,Q1b1a1a1i1a1a	G>A	G	A
NC_000024.9:g.18094109A>G	18094109	18094109+Z19345^,Q1b1a1a1i1a1a	Z19345^,Q1b1a1a1i1a1a	A>G	A	G
NC_000024.9:g.21704517G>C	21704517	21704517+Z19348,Q1b1a1a1i1a1a	Z19348,Q1b1a1a1i1a1a	G>C	G	C
NC_000024.9:g.23486494A>G	23486494	23486494+Z19350,Q1b1a1a1i1a1a	Z19350,Q1b1a1a1i1a1a	A>G	A	G
NC_000024.9:g.15977005G>A	15977005	15977005+Z19367,Q1b1a1a1j	Z19367,Q1b1a1a1j	G>A	G	A
NC_000024.9:g.16559961A>G	16559961	16559961+Z19432,Q1b1a1a1k	Z19432,Q1b1a1a1k	A>G	A	G
NC_000024.9:g.6759798G>A	6759798	6759798+Z19458,Q1b1a1a1h1	Z19458,Q1b1a1a1h1	G>A	G	A
NC_000024.9:g.6850436C>T	6850436	6850436+Z19460,Q1b1a1a1h1	Z19460,Q1b1a1a1h1	C>T	C	T
NC_000024.9:g.7274144G>T	7274144	7274144+Z19462,Q1b1a1a1h1	Z19462,Q1b1a1a1h1	G>T	G	T
NC_000024.9:g.14030866C>G	14030866	14030866+Z19476^,Q1b1a1a1h1	Z19476^,Q1b1a1a1h1	C>G	C	G
NC_000024.9:g.14497093T>C	14497093	14497093+Z19478^,Q1b1a1a1h1	Z19478^,Q1b1a1a1h1	T>C	T	C
NC_000024.9:g.14932787A>G	14932787	14932787+Z19479^,Q1b1a1a1h1	Z19479^,Q1b1a1a1h1	A>G	A	G
NC_000024.9:g.15050646T>G	15050646	15050646+Z19480,Q1b1a1a1h1	Z19480,Q1b1a1a1h1	T>G	T	G
NC_000024.9:g.15486922T>C	15486922	15486922+Z19481^,Q1b1a1a1h1	Z19481^,Q1b1a1a1h1	T>C	T	C
NC_000024.9:g.15517699G>A	15517699	15517699+Z19482,Q1b1a1a1h1	Z19482,Q1b1a1a1h1	G>A	G	A
NC_000024.9:g.15809817A>G	15809817	15809817+Z19483,Q1b1a1a1h1	Z19483,Q1b1a1a1h1	A>G	A	G
NC_000024.9:g.16170815T>C	16170815	16170815+Z19484,Q1b1a1a1h1	Z19484,Q1b1a1a1h1	T>C	T	C
NC_000024.9:g.16345569G>A	16345569	16345569+Z19485^,Q1b1a1a1h1	Z19485^,Q1b1a1a1h1	G>A	G	A
NC_000024.9:g.16442390G>C	16442390	16442390+Z19486,Q1b1a1a1h1	Z19486,Q1b1a1a1h1	G>C	G	C
NC_000024.9:g.16489245G>C	16489245	16489245+Z19487,Q1b1a1a1h1	Z19487,Q1b1a1a1h1	G>C	G	C
NC_000024.9:g.16623973G>A	16623973	16623973+Z19488,Q1b1a1a1h1	Z19488,Q1b1a1a1h1	G>A	G	A
NC_000024.9:g.16742289G>T	16742289	16742289+Z19489,Q1b1a1a1h1	Z19489,Q1b1a1a1h1	G>T	G	T
NC_000024.9:g.17205081A>G	17205081	17205081+Z19491,Q1b1a1a1h1	Z19491,Q1b1a1a1h1	A>G	A	G
NC_000024.9:g.17241270C>A	17241270	17241270+Z19492^,Q1b1a1a1h1	Z19492^,Q1b1a1a1h1	C>A	C	A
NC_000024.9:g.17618999A>G	17618999	17618999+Z19493,Q1b1a1a1h1	Z19493,Q1b1a1a1h1	A>G	A	G
NC_000024.9:g.17619765C>T	17619765	17619765+Z19494,Q1b1a1a1h1	Z19494,Q1b1a1a1h1	C>T	C	T
NC_000024.9:g.18587425C>A	18587425	18587425+Z19496^,Q1b1a1a1h1	Z19496^,Q1b1a1a1h1	C>A	C	A
NC_000024.9:g.18702565C>A	18702565	18702565+Z19497,Q1b1a1a1h1	Z19497,Q1b1a1a1h1	C>A	C	A
NC_000024.9:g.18803311G>A	18803311	18803311+Z19498^,Q1b1a1a1h1	Z19498^,Q1b1a1a1h1	G>A	G	A
NC_000024.9:g.21248701G>A	21248701	21248701+Z19499^,Q1b1a1a1h1	Z19499^,Q1b1a1a1h1	G>A	G	A
NC_000024.9:g.21442440G>A	21442440	21442440+Z19500^,Q1b1a1a1h1	Z19500^,Q1b1a1a1h1	G>A	G	A
NC_000024.9:g.21818186T>C	21818186	21818186+Z19502,Q1b1a1a1h1	Z19502,Q1b1a1a1h1	T>C	T	C
NC_000024.9:g.21958634A>G	21958634	21958634+Z19503,Q1b1a1a1h1	Z19503,Q1b1a1a1h1	A>G	A	G
NC_000024.9:g.21983408A>T	21983408	21983408+Z19504,Q1b1a1a1h1	Z19504,Q1b1a1a1h1	A>T	A	T
NC_000024.9:g.22074092A>G	22074092	22074092+Z19505^,Q1b1a1a1h1	Z19505^,Q1b1a1a1h1	A>G	A	G
NC_000024.9:g.22966265T>A	22966265	22966265+Z19511,Q1b1a1a1h1	Z19511,Q1b1a1a1h1	T>A	T	A
NC_000024.9:g.23453379A>T	23453379	23453379+Z19512,Q1b1a1a1h1	Z19512,Q1b1a1a1h1	A>T	A	T
NC_000024.9:g.23476184C>A	23476184	23476184+Z19513^,Q1b1a1a1h1	Z19513^,Q1b1a1a1h1	C>A	C	A
NC_000024.9:g.21954471G>A	21954471	21954471+Z19521^,Q1b1a1a1h1	Z19521^,Q1b1a1a1h1	G>A	G	A
NC_000024.9:g.7374678G>A	7374678	7374678+Z19523,Q1b1a1a1h1a	Z19523,Q1b1a1a1h1a	G>A	G	A
NC_000024.9:g.14192548A>T	14192548	14192548+Z19526,Q1b1a1a1h1a	Z19526,Q1b1a1a1h1a	A>T	A	T
NC_000024.9:g.14595006C>T	14595006	14595006+Z19527,Q1b1a1a1h1a	Z19527,Q1b1a1a1h1a	C>T	C	T
NC_000024.9:g.15707941C>G	15707941	15707941+Z19528,Q1b1a1a1h1a	Z19528,Q1b1a1a1h1a	C>G	C	G
NC_000024.9:g.16214674T>C	16214674	16214674+Z19529,Q1b1a1a1h1a	Z19529,Q1b1a1a1h1a	T>C	T	C
NC_000024.9:g.17575979C>T	17575979	17575979+Z19530,Q1b1a1a1h1a	Z19530,Q1b1a1a1h1a	C>T	C	T
NC_000024.9:g.18771654C>A	18771654	18771654+Z19531,Q1b1a1a1h1a	Z19531,Q1b1a1a1h1a	C>A	C	A
NC_000024.9:g.23246315C>G	23246315	23246315+Z19533,Q1b1a1a1h1a	Z19533,Q1b1a1a1h1a	C>G	C	G
NC_000024.9:g.23283819T>C	23283819	23283819+Z19534,Q1b1a1a1h1a	Z19534,Q1b1a1a1h1a	T>C	T	C
NC_000024.9:g.6711076A>G	6711076	6711076+Z19569,Q1b1a1a1e2	Z19569,Q1b1a1a1e2	A>G	A	G
NC_000024.9:g.6994528C>T	6994528	6994528+Z19575,Q1b1a1a1e2	Z19575,Q1b1a1a1e2	C>T	C	T
NC_000024.9:g.7013692G>A	7013692	7013692+Z19577,Q1b1a1a1e2	Z19577,Q1b1a1a1e2	G>A	G	A
NC_000024.9:g.7162460A>G	7162460	7162460+Z19579^,Q1b1a1a1e2	Z19579^,Q1b1a1a1e2	A>G	A	G
NC_000024.9:g.7327853G>T	7327853	7327853+Z19580,Q1b1a1a1e2	Z19580,Q1b1a1a1e2	G>T	G	T
NC_000024.9:g.14094659C>A	14094659	14094659+Z19601,Q1b1a1a1e2	Z19601,Q1b1a1a1e2	C>A	C	A
NC_000024.9:g.14284290G>T	14284290	14284290+Z19605,Q1b1a1a1e2	Z19605,Q1b1a1a1e2	G>T	G	T
NC_000024.9:g.14297478G>A	14297478	14297478+Z19606^,Q1b1a1a1e2	Z19606^,Q1b1a1a1e2	G>A	G	A
NC_000024.9:g.14325889G>T	14325889	14325889+Z19607,Q1b1a1a1e2	Z19607,Q1b1a1a1e2	G>T	G	T
NC_000024.9:g.14412783T>C	14412783	14412783+Z19609,Q1b1a1a1e2	Z19609,Q1b1a1a1e2	T>C	T	C
NC_000024.9:g.14458138T>G	14458138	14458138+Z19610,Q1b1a1a1e2	Z19610,Q1b1a1a1e2	T>G	T	G
NC_000024.9:g.14537137C>T	14537137	14537137+Z19613,Q1b1a1a1e2	Z19613,Q1b1a1a1e2	C>T	C	T
NC_000024.9:g.14714934C>T	14714934	14714934+Z19614,Q1b1a1a1e2	Z19614,Q1b1a1a1e2	C>T	C	T
NC_000024.9:g.15126912T>C	15126912	15126912+Z19616,Q1b1a1a1e2	Z19616,Q1b1a1a1e2	T>C	T	C
NC_000024.9:g.15217056A>T	15217056	15217056+Z19618,Q1b1a1a1e2	Z19618,Q1b1a1a1e2	A>T	A	T
NC_000024.9:g.15517982G>A	15517982	15517982+Z19622^,Q1b1a1a1e2	Z19622^,Q1b1a1a1e2	G>A	G	A
NC_000024.9:g.15608126C>T	15608126	15608126+Z19623,Q1b1a1a1e2	Z19623,Q1b1a1a1e2	C>T	C	T
NC_000024.9:g.15910856T>C	15910856	15910856+Z19625^,Q1b1a1a1e2	Z19625^,Q1b1a1a1e2	T>C	T	C
NC_000024.9:g.15928297C>T	15928297	15928297+Z19626^,Q1b1a1a1e2	Z19626^,Q1b1a1a1e2	C>T	C	T
NC_000024.9:g.16691166A>G	16691166	16691166+Z19630,Q1b1a1a1e2	Z19630,Q1b1a1a1e2	A>G	A	G
NC_000024.9:g.16987383A>T	16987383	16987383+Z19631,Q1b1a1a1e2	Z19631,Q1b1a1a1e2	A>T	A	T
NC_000024.9:g.17497843C>T	17497843	17497843+Z19632,Q1b1a1a1e2	Z19632,Q1b1a1a1e2	C>T	C	T
NC_000024.9:g.17511877A>T	17511877	17511877+Z19633,Q1b1a1a1e2	Z19633,Q1b1a1a1e2	A>T	A	T
NC_000024.9:g.17752548T>A	17752548	17752548+Z19634,Q1b1a1a1e2	Z19634,Q1b1a1a1e2	T>A	T	A
NC_000024.9:g.18045725T>C	18045725	18045725+Z19636,Q1b1a1a1e2	Z19636,Q1b1a1a1e2	T>C	T	C
NC_000024.9:g.18121695C>T	18121695	18121695+Z19638,Q1b1a1a1e2	Z19638,Q1b1a1a1e2	C>T	C	T
NC_000024.9:g.19071943C>A	19071943	19071943+Z19642,Q1b1a1a1e2	Z19642,Q1b1a1a1e2	C>A	C	A
NC_000024.9:g.19246840T>C	19246840	19246840+Z19646,Q1b1a1a1e2	Z19646,Q1b1a1a1e2	T>C	T	C
NC_000024.9:g.19358046C>A	19358046	19358046+Z19647,Q1b1a1a1e2	Z19647,Q1b1a1a1e2	C>A	C	A
NC_000024.9:g.21169028C>G	21169028	21169028+Z19649,Q1b1a1a1e2	Z19649,Q1b1a1a1e2	C>G	C	G
NC_000024.9:g.21945450G>A	21945450	21945450+Z19651,Q1b1a1a1e2	Z19651,Q1b1a1a1e2	G>A	G	A
NC_000024.9:g.22119845A>G	22119845	22119845+Z19653,Q1b1a1a1e2	Z19653,Q1b1a1a1e2	A>G	A	G
NC_000024.9:g.22999565T>C	22999565	22999565+Z19660,Q1b1a1a1e2	Z19660,Q1b1a1a1e2	T>C	T	C
NC_000024.9:g.23087404C>A	23087404	23087404+Z19661,Q1b1a1a1e2	Z19661,Q1b1a1a1e2	C>A	C	A
NC_000024.9:g.23202179G>A	23202179	23202179+Z19665,Q1b1a1a1e2	Z19665,Q1b1a1a1e2	G>A	G	A
NC_000024.9:g.17931277C>T	17931277	17931277+Z20410,L1a1	Z20410,L1a1	C>T	C	T
NC_000024.9:g.15392961A>G	15392961	15392961+Z35458,Q1b1a1a1h1	Z35458,Q1b1a1a1h1	A>G	A	G
NC_000024.9:g.19438600G>T	19438600	19438600+Z35464,Q1b1a1a1h1	Z35464,Q1b1a1a1h1	G>T	G	T
NC_000024.9:g.15195474A>G	15195474	15195474+Z35471,Q1b1a1a1h1a3~	Z35471,Q1b1a1a1h1a3~	A>G	A	G
NC_000024.9:g.6662951C>T	6662951	6662951+Z35487,Q1b1a1a1h1a4b1~	Z35487,Q1b1a1a1h1a4b1~	C>T	C	T
NC_000024.9:g.14035236A>C	14035236	14035236+Z35513,Q1b1a1a1p	Z35513,Q1b1a1a1p	A>C	A	C
NC_000024.9:g.14209686C>T	14209686	14209686+Z35514,Q1b1a1a1p	Z35514,Q1b1a1a1p	C>T	C	T
NC_000024.9:g.15758325A>G	15758325	15758325+Z35523,Q1b1a1a1p	Z35523,Q1b1a1a1p	A>G	A	G
NC_000024.9:g.16198670T>A	16198670	16198670+Z35527,Q1b1a1a1p	Z35527,Q1b1a1a1p	T>A	T	A
NC_000024.9:g.16760359G>A	16760359	16760359+Z35531,Q1b1a1a1p	Z35531,Q1b1a1a1p	G>A	G	A
NC_000024.9:g.17182519A>G	17182519	17182519+Z35535,Q1b1a1a1p	Z35535,Q1b1a1a1p	A>G	A	G
NC_000024.9:g.17471877T>C	17471877	17471877+Z35539,Q1b1a1a1p	Z35539,Q1b1a1a1p	T>C	T	C
NC_000024.9:g.18963252A>G	18963252	18963252+Z35549,Q1b1a1a1p	Z35549,Q1b1a1a1p	A>G	A	G
NC_000024.9:g.19112760A>G	19112760	19112760+Z35551,Q1b1a1a1p	Z35551,Q1b1a1a1p	A>G	A	G
NC_000024.9:g.19142712T>A	19142712	19142712+Z35552,Q1b1a1a1p	Z35552,Q1b1a1a1p	T>A	T	A
NC_000024.9:g.19405271G>A	19405271	19405271+Z35555,Q1b1a1a1p	Z35555,Q1b1a1a1p	G>A	G	A
NC_000024.9:g.19519108T>C	19519108	19519108+Z35559,Q1b1a1a1p	Z35559,Q1b1a1a1p	T>C	T	C
NC_000024.9:g.19543559A>G	19543559	19543559+Z35561,Q1b1a1a1p	Z35561,Q1b1a1a1p	A>G	A	G

NC_000024.9:g.21239610T>A	21239610	21239610+Z35563,Q1b1a1a1p	Z35563,Q1b1a1a1p	T>A	T	A
NC_000024.9:g.21569196G>A	21569196	21569196+Z35569,Q1b1a1a1p	Z35569,Q1b1a1a1p	G>A	G	A
NC_000024.9:g.21572507T>C	21572507	21572507+Z35570,Q1b1a1a1p	Z35570,Q1b1a1a1p	T>C	T	C
NC_000024.9:g.23007685T>C	23007685	23007685+Z35583,Q1b1a1a1p	Z35583,Q1b1a1a1p	T>C	T	C
NC_000024.9:g.23144492G>A	23144492	23144492+Z35585,Q1b1a1a1p	Z35585,Q1b1a1a1p	G>A	G	A
NC_000024.9:g.23432352T>C	23432352	23432352+Z35587,Q1b1a1a1p	Z35587,Q1b1a1a1p	T>C	T	C
NC_000024.9:g.28502680A>G	28502680	28502680	Z35597	A>G	A	G
NC_000024.9:g.2663470C>T	2663470	2663470+Z35598,Q1b1a1a1k2~	Z35598,Q1b1a1a1k2~	C>T	C	T
NC_000024.9:g.2764120G>A	2764120	2764120+Z35599,Q1b1a1a1k2~	Z35599,Q1b1a1a1k2~	G>A	G	A
NC_000024.9:g.2754187C>T	2754187	2754187+Z35715,Q1b1a1a1e2b~	Z35715,Q1b1a1a1e2b~	C>T	C	T
NC_000024.9:g.6758908A>G	6758908	6758908+Z35742,Q1b1a1a1t~	Z35742,Q1b1a1a1t~	A>G	A	G
NC_000024.9:g.7015110A>T	7015110	7015110+Z35743,Q1b1a1a1t~	Z35743,Q1b1a1a1t~	A>T	A	T
NC_000024.9:g.15083967C>T	15083967	15083967+Z35840,Q1b1a1a1f	Z35840,Q1b1a1a1f	C>T	C	T
NC_000024.9:g.21718405T>G	21718405	21718405+Z35841,Q1b1a1a1f	Z35841,Q1b1a1a1f	T>G	T	G
NC_000024.9:g.2851463C>G	2851463	2851463+Z35896,Q1b1a1a1i1a1a1~	Z35896,Q1b1a1a1i1a1a1~	C>G	C	G
NC_000024.9:g.7579211C>T	7579211	7579211+Z35900,Q1b1a1a1i1a1a1~	Z35900,Q1b1a1a1i1a1a1~	C>T	C	T
NC_000024.9:g.28773518C>G	28773518	28773518+Z35922,Q1b1a1a1i1a2	Z35922,Q1b1a1a1i1a2	C>G	C	G
NC_000024.9:g.2796214G>C	2796214	2796214+Z36358	Z36358	G>C	G	C
NC_000024.9:g.14143224G>A	14143224	14143224+Z41402,S1a1b1d	Z41402,S1a1b1d	G>A	G	A
NC_000024.9:g.17415838C>T	17415838	17415838+Z44971,G2a2b2a2	Z44971,G2a2b2a2	C>T	C	T
NC_000024.9:g.24441779C>T	24441779	24441779+Z5829	Z5829	C>T	C	T
NC_000024.9:g.2723295A>C	2723295	2723295+Z5906,Z32422,C1b1a2a	Z5906,Z32422,C1b1a2a	A>C	A	C
NC_000024.9:g.6844661G>A	6844661	6844661+Z5907,Q1b1a1a1h1a	Z5907,Q1b1a1a1h1a	G>A	G	A
NC_000024.9:g.6675536T>C	6675536	6675536+Z5908,Q1b1a1a1i	Z5908,Q1b1a1a1i	T>C	T	C
NC_000024.9:g.7047706T>C	7047706	7047706+Z5910,Q1b1a1a1i1a	Z5910,Q1b1a1a1i1a	T>C	T	C
NC_000024.9:g.14318862G>A	14318862	14318862+Z5911,Q1b1a1a1i1a1	Z5911,Q1b1a1a1i1a1	G>A	G	A
NC_000024.9:g.16390052C>A	16390052	16390052+Z5912,Q1b1a1a1i1a1a	Z5912,Q1b1a1a1i1a1a	C>A	C	A
NC_000024.9:g.7038321C>T	7038321	7038321+Z5919,L1a2a	Z5919,L1a2a	C>T	C	T
NC_000024.9:g.7461466T>A	7461466	7461466+Z6132,G2a2a1a2a1	Z6132,G2a2a1a2a1	T>A	T	A
NC_000024.9:g.14534937G>A	14534937	14534937+Z6340,G2a2b1c~	Z6340,G2a2b1c~	G>A	G	A
NC_000024.9:g.28595831C>T	28595831	28595831+Z6917,G2a2b2a1a1a1b4~	Z6917,G2a2b2a1a1a1b4~	C>T	C	T
NC_000024.9:g.14181495G>A	14181495	14181495+Z8028,Q1b1a1a1h	Z8028,Q1b1a1a1h	G>A	G	A
NC_000024.9:g.7152988G>A	7152988	7152988+Z8297,J2b2a2b1a~	Z8297,J2b2a2b1a~	G>A	G	A
NC_000024.9:g.2658297G>T	2658297	2658297	ZK1	G>T	G	T
NC_000024.9:g.2725427G>C	2725427	2725427	ZK2	G>C	G	C
NC_000024.9:g.2736256A>C	2736256	2736256	ZK3	A>C	A	C
NC_000024.9:g.2739629G>C	2739629	2739629	ZK4	G>C	G	C
NC_000024.9:g.2749436T>G	2749436	2749436	ZK5	T>G	T	G
NC_000024.9:g.2754983C>G	2754983	2754983	ZK6	C>G	C	G
NC_000024.9:g.2755674G>C	2755674	2755674	ZK7	G>C	G	C
NC_000024.9:g.2777243C>G	2777243	2777243	ZK8	C>G	C	G
NC_000024.9:g.2778491A>C	2778491	2778491	ZK9	A>C	A	C
NC_000024.9:g.2797939G>A	2797939	2797939	ZK10	G>A	G	A
NC_000024.9:g.2818627G>C	2818627	2818627	ZK11	G>C	G	C
NC_000024.9:g.2823201A>G	2823201	2823201	ZK12	A>G	A	G
NC_000024.9:g.2837010C>G	2837010	2837010	ZK13	C>G	C	G
NC_000024.9:g.2845426T>C	2845426	2845426	ZK14	T>C	T	C
NC_000024.9:g.2865000G>A	2865000	2865000	ZK15	G>A	G	A
NC_000024.9:g.2871750T>C	2871750	2871750	ZK16	T>C	T	C
NC_000024.9:g.2871757C>T	2871757	2871757	ZK17	C>T	C	T
NC_000024.9:g.2878050C>A	2878050	2878050	ZK18	C>A	C	A
NC_000024.9:g.2878432G>A	2878432	2878432	ZK19	G>A	G	A
NC_000024.9:g.2882209C>T	2882209	2882209	ZK20	C>T	C	T
NC_000024.9:g.2890586C>G	2890586	2890586	ZK21	C>G	C	G
NC_000024.9:g.2894086G>A	2894086	2894086	ZK22	G>A	G	A
NC_000024.9:g.2906535C>A	2906535	2906535	ZK23	C>A	C	A
NC_000024.9:g.6628740A>G	6628740	6628740	ZK24	A>G	A	G
NC_000024.9:g.6629232T>C	6629232	6629232	ZK25	T>C	T	C
NC_000024.9:g.6633449T>C	6633449	6633449	ZK26	T>C	T	C
NC_000024.9:g.6657862T>C	6657862	6657862	ZK27	T>C	T	C
NC_000024.9:g.6659537C>T	6659537	6659537	ZK28	C>T	C	T
NC_000024.9:g.6664760T>C	6664760	6664760	ZK29	T>C	T	C
NC_000024.9:g.6690357G>A	6690357	6690357	ZK30	G>A	G	A
NC_000024.9:g.6703485C>T	6703485	6703485	ZK31	C>T	C	T
NC_000024.9:g.6716389G>T	6716389	6716389	ZK32	G>T	G	T
NC_000024.9:g.6754680A>G	6754680	6754680	ZK33	A>G	A	G
NC_000024.9:g.6760279A>T	6760279	6760279	ZK34	A>T	A	T
NC_000024.9:g.6770044C>T	6770044	6770044	ZK35	C>T	C	T
NC_000024.9:g.6773291C>A	6773291	6773291	ZK36	C>A	C	A
NC_000024.9:g.6811645C>G	6811645	6811645	ZK37	C>G	C	G
NC_000024.9:g.6822986G>A	6822986	6822986	ZK38	G>A	G	A
NC_000024.9:g.6825867A>T	6825867	6825867	ZK39	A>T	A	T
NC_000024.9:g.6830783G>C	6830783	6830783	ZK40	G>C	G	C
NC_000024.9:g.6837506A>G	6837506	6837506	ZK41	A>G	A	G
NC_000024.9:g.6845063C>T	6845063	6845063	ZK42	C>T	C	T
NC_000024.9:g.6848493A>G	6848493	6848493	ZK43	A>G	A	G
NC_000024.9:g.6875655C>T	6875655	6875655	ZK44	C>T	C	T
NC_000024.9:g.6878588G>A	6878588	6878588	ZK45	G>A	G	A
NC_000024.9:g.6886966T>A	6886966	6886966	ZK46	T>A	T	A
NC_000024.9:g.6887165A>G	6887165	6887165	ZK47	A>G	A	G
NC_000024.9:g.6894680G>C	6894680	6894680	ZK48	G>C	G	C
NC_000024.9:g.6931583G>C	6931583	6931583	ZK49	G>C	G	C
NC_000024.9:g.7011267T>C	7011267	7011267	ZK50	T>C	T	C
NC_000024.9:g.7029140A>C	7029140	7029140	ZK51	A>C	A	C
NC_000024.9:g.7039192G>A	7039192	7039192	ZK52	G>A	G	A
NC_000024.9:g.7045357A>C	7045357	7045357	ZK53	A>C	A	C
NC_000024.9:g.7049012A>T	7049012	7049012	ZK54	A>T	A	T
NC_000024.9:g.7049608T>C	7049608	7049608	ZK55	T>C	T	C
NC_000024.9:g.7078775G>T	7078775	7078775	ZK56	G>T	G	T
NC_000024.9:g.7085346C>G	7085346	7085346	ZK57	C>G	C	G
NC_000024.9:g.7112649C>T	7112649	7112649	ZK58	C>T	C	T
NC_000024.9:g.7135943C>T	7135943	7135943	ZK59	C>T	C	T
NC_000024.9:g.7140017T>C	7140017	7140017	ZK60	T>C	T	C
NC_000024.9:g.7145745C>G	7145745	7145745	ZK61	C>G	C	G
NC_000024.9:g.7174231C>T	7174231	7174231	ZK62	C>T	C	T
NC_000024.9:g.7177701G>C	7177701	7177701	ZK63	G>C	G	C
NC_000024.9:g.7187335A>G	7187335	7187335	ZK64	A>G	A	G
NC_000024.9:g.7202529C>T	7202529	7202529	ZK65	C>T	C	T
NC_000024.9:g.7202986T>C	7202986	7202986	ZK66	T>C	T	C
NC_000024.9:g.7206113G>C	7206113	7206113	ZK67	G>C	G	C
NC_000024.9:g.7242952G>A	7242952	7242952	ZK68	G>A	G	A
NC_000024.9:g.7255213G>T	7255213	7255213	ZK69	G>T	G	T
NC_000024.9:g.7259274C>T	7259274	7259274	ZK70	C>T	C	T
NC_000024.9:g.7301898A>C	7301898	7301898	ZK71	A>C	A	C

NC_000024.9:g.7316940A>G	7316940	7316940	ZK72	A>G	A	G
NC_000024.9:g.7344729T>G	7344729	7344729	ZK73	T>G	T	G
NC_000024.9:g.7350228C>T	7350228	7350228	ZK74	C>T	C	T
NC_000024.9:g.7355921T>C	7355921	7355921	ZK75	T>C	T	C
NC_000024.9:g.7356120G>A	7356120	7356120	ZK76	G>A	G	A
NC_000024.9:g.7380886G>C	7380886	7380886	ZK77	G>C	G	C
NC_000024.9:g.7397532C>G	7397532	7397532	ZK78	C>G	C	G
NC_000024.9:g.7402303T>C	7402303	7402303	ZK79	T>C	T	C
NC_000024.9:g.7415547T>C	7415547	7415547	ZK80	T>C	T	C
NC_000024.9:g.7420284T>C	7420284	7420284	ZK81	T>C	T	C
NC_000024.9:g.7432761A>G	7432761	7432761	ZK82	A>G	A	G
NC_000024.9:g.7465455A>G	7465455	7465455	ZK83	A>G	A	G
NC_000024.9:g.13137783C>A	13137783	13137783	ZK84	C>A	C	A
NC_000024.9:g.13871383C>T	13871383	13871383	ZK85	C>T	C	T
NC_000024.9:g.13893942A>C	13893942	13893942	ZK86	A>C	A	C
NC_000024.9:g.13900316C>A	13900316	13900316	ZK87	C>A	C	A
NC_000024.9:g.13904223A>G	13904223	13904223	ZK88	A>G	A	G
NC_000024.9:g.13936882C>T	13936882	13936882	ZK89	C>T	C	T
NC_000024.9:g.13945571G>A	13945571	13945571	ZK90	G>A	G	A
NC_000024.9:g.13960050G>A	13960050	13960050	ZK91	G>A	G	A
NC_000024.9:g.13971032T>G	13971032	13971032	ZK92	T>G	T	G
NC_000024.9:g.13975721G>A	13975721	13975721	ZK93	G>A	G	A
NC_000024.9:g.13990022C>T	13990022	13990022	ZK94	C>T	C	T
NC_000024.9:g.13991776T>A	13991776	13991776	ZK95	T>A	T	A
NC_000024.9:g.13992400T>A	13992400	13992400	ZK96	T>A	T	A
NC_000024.9:g.14003457T>G	14003457	14003457	ZK97	T>G	T	G
NC_000024.9:g.14004737C>T	14004737	14004737	ZK98	C>T	C	T
NC_000024.9:g.14010670G>T	14010670	14010670	ZK99	G>T	G	T
NC_000024.9:g.14015369T>G	14015369	14015369	ZK100	T>G	T	G
NC_000024.9:g.14020875G>A	14020875	14020875	ZK101	G>A	G	A
NC_000024.9:g.14024712A>G	14024712	14024712	ZK102	A>G	A	G
NC_000024.9:g.14028098C>A	14028098	14028098	ZK103	C>A	C	A
NC_000024.9:g.14038787G>A	14038787	14038787	ZK104	G>A	G	A
NC_000024.9:g.14039954G>A	14039954	14039954	ZK105	G>A	G	A
NC_000024.9:g.14049777T>C	14049777	14049777	ZK106	T>C	T	C
NC_000024.9:g.14064034C>A	14064034	14064034	ZK107	C>A	C	A
NC_000024.9:g.14066665G>A	14066665	14066665	ZK108	G>A	G	A
NC_000024.9:g.14078246G>T	14078246	14078246	ZK109	G>T	G	T
NC_000024.9:g.14081814C>G	14081814	14081814	ZK110	C>G	C	G
NC_000024.9:g.14099995G>A	14099995	14099995	ZK111	G>A	G	A
NC_000024.9:g.14102817T>C	14102817	14102817	ZK112	T>C	T	C
NC_000024.9:g.14107952C>A	14107952	14107952	ZK113	C>A	C	A
NC_000024.9:g.14115272G>T	14115272	14115272	ZK114	G>T	G	T
NC_000024.9:g.14116347T>C	14116347	14116347	ZK115	T>C	T	C
NC_000024.9:g.14126812T>A	14126812	14126812	ZK116	T>A	T	A
NC_000024.9:g.14139392T>G	14139392	14139392	ZK117	T>G	T	G
NC_000024.9:g.14150483C>G	14150483	14150483	ZK118	C>G	C	G
NC_000024.9:g.14151953G>T	14151953	14151953	ZK119	G>T	G	T
NC_000024.9:g.14169072C>T	14169072	14169072	ZK120	C>T	C	T
NC_000024.9:g.14174650G>A	14174650	14174650	ZK121	G>A	G	A
NC_000024.9:g.14185936A>T	14185936	14185936	ZK122	A>T	A	T
NC_000024.9:g.14207974C>T	14207974	14207974	ZK123	C>T	C	T
NC_000024.9:g.14209814T>C	14209814	14209814	ZK124	T>C	T	C
NC_000024.9:g.14210382A>G	14210382	14210382	ZK125	A>G	A	G
NC_000024.9:g.14215329T>C	14215329	14215329	ZK126	T>C	T	C
NC_000024.9:g.14227003G>A	14227003	14227003	ZK127	G>A	G	A
NC_000024.9:g.14236890G>A	14236890	14236890	ZK128	G>A	G	A
NC_000024.9:g.14241037G>A	14241037	14241037	ZK129	G>A	G	A
NC_000024.9:g.14244675A>T	14244675	14244675	ZK130	A>T	A	T
NC_000024.9:g.14244757T>C	14244757	14244757	ZK131	T>C	T	C
NC_000024.9:g.14249639C>A	14249639	14249639	ZK132	C>A	C	A
NC_000024.9:g.14254493G>C	14254493	14254493	ZK133	G>C	G	C
NC_000024.9:g.14263095G>C	14263095	14263095	ZK134	G>C	G	C
NC_000024.9:g.14264277G>A	14264277	14264277	ZK135	G>A	G	A
NC_000024.9:g.14267091A>G	14267091	14267091	ZK136	A>G	A	G
NC_000024.9:g.14275124G>A	14275124	14275124	ZK137	G>A	G	A
NC_000024.9:g.14275167A>T	14275167	14275167	ZK138	A>T	A	T
NC_000024.9:g.14276909A>C	14276909	14276909	ZK139	A>C	A	C
NC_000024.9:g.14277390C>A	14277390	14277390	ZK140	C>A	C	A
NC_000024.9:g.14301603G>A	14301603	14301603	ZK141	G>A	G	A
NC_000024.9:g.14311081C>A	14311081	14311081	ZK142	C>A	C	A
NC_000024.9:g.14311744G>A	14311744	14311744	ZK143	G>A	G	A
NC_000024.9:g.14312005G>T	14312005	14312005	ZK144	G>T	G	T
NC_000024.9:g.14321695C>A	14321695	14321695	ZK145	C>A	C	A
NC_000024.9:g.14323889T>C	14323889	14323889	ZK146	T>C	T	C
NC_000024.9:g.14327778C>T	14327778	14327778	ZK147	C>T	C	T
NC_000024.9:g.14335801T>A	14335801	14335801	ZK148	T>A	T	A
NC_000024.9:g.14339962C>T	14339962	14339962	ZK149	C>T	C	T
NC_000024.9:g.14349562C>T	14349562	14349562	ZK150	C>T	C	T
NC_000024.9:g.14365115G>C	14365115	14365115	ZK151	G>C	G	C
NC_000024.9:g.14368553T>C	14368553	14368553	ZK152	T>C	T	C
NC_000024.9:g.14370702T>A	14370702	14370702	ZK153	T>A	T	A
NC_000024.9:g.14380079G>A	14380079	14380079	ZK154	G>A	G	A
NC_000024.9:g.14385124C>G	14385124	14385124	ZK155	C>G	C	G
NC_000024.9:g.14387009G>C	14387009	14387009	ZK156	G>C	G	C
NC_000024.9:g.14388103G>A	14388103	14388103	ZK157	G>A	G	A
NC_000024.9:g.14396029G>A	14396029	14396029	ZK158	G>A	G	A
NC_000024.9:g.14442658G>T	14442658	14442658	ZK159	G>T	G	T
NC_000024.9:g.14453501G>A	14453501	14453501	ZK160	G>A	G	A
NC_000024.9:g.14457822A>T	14457822	14457822	ZK161	A>T	A	T
NC_000024.9:g.14458794A>G	14458794	14458794	ZK162	A>G	A	G
NC_000024.9:g.14483215C>T	14483215	14483215	ZK163	C>T	C	T
NC_000024.9:g.14487522A>T	14487522	14487522	ZK164	A>T	A	T
NC_000024.9:g.14520832T>A	14520832	14520832	ZK165	T>A	T	A
NC_000024.9:g.14534669A>G	14534669	14534669	ZK166	A>G	A	G
NC_000024.9:g.14548221G>A	14548221	14548221	ZK167	G>A	G	A
NC_000024.9:g.14563735T>C	14563735	14563735	ZK168	T>C	T	C
NC_000024.9:g.14580089G>C	14580089	14580089	ZK169	G>C	G	C
NC_000024.9:g.14580361G>A	14580361	14580361	ZK170	G>A	G	A
NC_000024.9:g.14581508T>C	14581508	14581508	ZK171	T>C	T	C
NC_000024.9:g.14581899C>G	14581899	14581899	ZK172	C>G	C	G
NC_000024.9:g.14584784G>A	14584784	14584784	ZK173	G>A	G	A
NC_000024.9:g.14586934A>T	14586934	14586934	ZK174	A>T	A	T
NC_000024.9:g.14589639A>G	14589639	14589639	ZK175	A>G	A	G

NC_000024.9:g.14593023G>A	14593023	14593023	ZK176	G>A	G	A
NC_000024.9:g.14600911C>T	14600911	14600911	ZK177	C>T	C	T
NC_000024.9:g.14607093C>T	14607093	14607093	ZK178	C>T	C	T
NC_000024.9:g.14623254C>T	14623254	14623254	ZK179	C>T	C	T
NC_000024.9:g.14629424G>T	14629424	14629424	ZK180	G>T	G	T
NC_000024.9:g.14653497C>T	14653497	14653497	ZK181	C>T	C	T
NC_000024.9:g.14656757C>G	14656757	14656757	ZK182	C>G	C	G
NC_000024.9:g.14671191G>T	14671191	14671191	ZK183	G>T	G	T
NC_000024.9:g.14696342T>C	14696342	14696342	ZK184	T>C	T	C
NC_000024.9:g.14724756A>C	14724756	14724756	ZK185	A>C	A	C
NC_000024.9:g.14800761C>T	14800761	14800761	ZK186	C>T	C	T
NC_000024.9:g.14803695C>T	14803695	14803695	ZK187	C>T	C	T
NC_000024.9:g.14812516G>A	14812516	14812516	ZK188	G>A	G	A
NC_000024.9:g.14838075A>T	14838075	14838075	ZK189	A>T	A	T
NC_000024.9:g.14846451G>C	14846451	14846451	ZK190	G>C	G	C
NC_000024.9:g.14878050T>G	14878050	14878050	ZK191	T>G	T	G
NC_000024.9:g.14895457A>G	14895457	14895457	ZK192	A>G	A	G
NC_000024.9:g.14904215A>G	14904215	14904215	ZK193	A>G	A	G
NC_000024.9:g.14913190G>T	14913190	14913190	ZK194	G>T	G	T
NC_000024.9:g.14941773A>T	14941773	14941773	ZK195	A>T	A	T
NC_000024.9:g.14969881C>G	14969881	14969881	ZK196	C>G	C	G
NC_000024.9:g.14975491T>A	14975491	14975491	ZK197	T>A	T	A
NC_000024.9:g.14989304T>A	14989304	14989304	ZK198	T>A	T	A
NC_000024.9:g.14993204T>A	14993204	14993204	ZK199	T>A	T	A
NC_000024.9:g.15021992A>G	15021992	15021992	ZK200	A>G	A	G
NC_000024.9:g.15069452C>T	15069452	15069452	ZK201	C>T	C	T
NC_000024.9:g.15071532A>G	15071532	15071532	ZK202	A>G	A	G
NC_000024.9:g.15071937A>G	15071937	15071937	ZK203	A>G	A	G
NC_000024.9:g.15085330C>A	15085330	15085330	ZK204	C>A	C	A
NC_000024.9:g.15095790A>G	15095790	15095790	ZK205	A>G	A	G
NC_000024.9:g.15110545T>A	15110545	15110545	ZK206	T>A	T	A
NC_000024.9:g.15128986A>T	15128986	15128986	ZK207	A>T	A	T
NC_000024.9:g.15129421G>A	15129421	15129421	ZK208	G>A	G	A
NC_000024.9:g.15134247A>T	15134247	15134247	ZK209	A>T	A	T
NC_000024.9:g.15161138A>G	15161138	15161138	ZK210	A>G	A	G
NC_000024.9:g.15193188C>T	15193188	15193188	ZK211	C>T	C	T
NC_000024.9:g.15236600G>T	15236600	15236600	ZK212	G>T	G	T
NC_000024.9:g.15257061T>C	15257061	15257061	ZK213	T>C	T	C
NC_000024.9:g.15279799G>A	15279799	15279799	ZK214	G>A	G	A
NC_000024.9:g.15289683A>T	15289683	15289683	ZK215	A>T	A	T
NC_000024.9:g.15298356G>C	15298356	15298356	ZK216	G>C	G	C
NC_000024.9:g.15314410T>C	15314410	15314410	ZK217	T>C	T	C
NC_000024.9:g.15331265A>T	15331265	15331265	ZK218	A>T	A	T
NC_000024.9:g.15337575C>T	15337575	15337575	ZK219	C>T	C	T
NC_000024.9:g.15344317G>C	15344317	15344317	ZK220	G>C	G	C
NC_000024.9:g.15349821G>A	15349821	15349821	ZK221	G>A	G	A
NC_000024.9:g.15364354A>T	15364354	15364354	ZK222	A>T	A	T
NC_000024.9:g.15405050C>A	15405050	15405050	ZK223	C>A	C	A
NC_000024.9:g.15418709G>A	15418709	15418709	ZK224	G>A	G	A
NC_000024.9:g.15427788C>A	15427788	15427788	ZK225	C>A	C	A
NC_000024.9:g.15461432T>G	15461432	15461432	ZK226	T>G	T	G
NC_000024.9:g.15471756T>C	15471756	15471756	ZK227	T>C	T	C
NC_000024.9:g.15493480C>T	15493480	15493480	ZK228	C>T	C	T
NC_000024.9:g.15518155T>C	15518155	15518155	ZK229	T>C	T	C
NC_000024.9:g.15527559C>T	15527559	15527559	ZK230	C>T	C	T
NC_000024.9:g.15537720A>T	15537720	15537720	ZK231	A>T	A	T
NC_000024.9:g.15539991T>C	15539991	15539991	ZK232	T>C	T	C
NC_000024.9:g.15556390A>T	15556390	15556390	ZK233	A>T	A	T
NC_000024.9:g.15564954A>T	15564954	15564954	ZK234	A>T	A	T
NC_000024.9:g.15580919C>A	15580919	15580919	ZK235	C>A	C	A
NC_000024.9:g.15592252C>G	15592252	15592252	ZK236	C>G	C	G
NC_000024.9:g.15594481T>C	15594481	15594481	ZK237	T>C	T	C
NC_000024.9:g.15597375T>C	15597375	15597375	ZK238	T>C	T	C
NC_000024.9:g.15607224A>T	15607224	15607224	ZK239	A>T	A	T
NC_000024.9:g.15655118C>G	15655118	15655118	ZK240	C>G	C	G
NC_000024.9:g.15692563G>A	15692563	15692563	ZK241	G>A	G	A
NC_000024.9:g.15697600G>A	15697600	15697600	ZK242	G>A	G	A
NC_000024.9:g.15705240T>A	15705240	15705240	ZK243	T>A	T	A
NC_000024.9:g.15712836G>T	15712836	15712836	ZK244	G>T	G	T
NC_000024.9:g.15724939C>T	15724939	15724939	ZK245	C>T	C	T
NC_000024.9:g.15737323C>G	15737323	15737323	ZK246	C>G	C	G
NC_000024.9:g.15749412G>C	15749412	15749412	ZK247	G>C	G	C
NC_000024.9:g.15752140C>A	15752140	15752140	ZK248	C>A	C	A
NC_000024.9:g.15752252C>T	15752252	15752252	ZK249	C>T	C	T
NC_000024.9:g.15765495G>A	15765495	15765495	ZK250	G>A	G	A
NC_000024.9:g.15786602G>T	15786602	15786602	ZK251	G>T	G	T
NC_000024.9:g.15798047G>A	15798047	15798047	ZK252	G>A	G	A
NC_000024.9:g.15800206T>G	15800206	15800206	ZK253	T>G	T	G
NC_000024.9:g.15813874C>T	15813874	15813874	ZK254	C>T	C	T
NC_000024.9:g.15814551G>A	15814551	15814551	ZK255	G>A	G	A
NC_000024.9:g.15820186G>C	15820186	15820186	ZK256	G>C	G	C
NC_000024.9:g.15825552G>A	15825552	15825552	ZK257	G>A	G	A
NC_000024.9:g.15828194A>G	15828194	15828194	ZK258	A>G	A	G
NC_000024.9:g.15829841C>T	15829841	15829841	ZK259	C>T	C	T
NC_000024.9:g.15830183C>T	15830183	15830183	ZK260	C>T	C	T
NC_000024.9:g.15861608C>T	15861608	15861608	ZK261	C>T	C	T
NC_000024.9:g.15872207A>T	15872207	15872207	ZK262	A>T	A	T
NC_000024.9:g.15920796T>C	15920796	15920796	ZK263	T>C	T	C
NC_000024.9:g.15931442C>A	15931442	15931442	ZK264	C>A	C	A
NC_000024.9:g.15936905G>A	15936905	15936905	ZK265	G>A	G	A
NC_000024.9:g.15944858G>C	15944858	15944858	ZK266	G>C	G	C
NC_000024.9:g.15954804C>T	15954804	15954804	ZK267	C>T	C	T
NC_000024.9:g.15965638A>G	15965638	15965638	ZK268	A>G	A	G
NC_000024.9:g.15969023G>C	15969023	15969023	ZK269	G>C	G	C
NC_000024.9:g.15969590T>C	15969590	15969590	ZK270	T>C	T	C
NC_000024.9:g.15990295G>T	15990295	15990295	ZK271	G>T	G	T
NC_000024.9:g.16015261A>C	16015261	16015261	ZK272	A>C	A	C
NC_000024.9:g.16017604A>G	16017604	16017604	ZK273	A>G	A	G
NC_000024.9:g.16042582T>A	16042582	16042582	ZK274	T>A	T	A
NC_000024.9:g.16057211G>T	16057211	16057211	ZK275	G>T	G	T
NC_000024.9:g.16065273C>A	16065273	16065273	ZK276	C>A	C	A
NC_000024.9:g.16070115C>T	16070115	16070115	ZK277	C>T	C	T
NC_000024.9:g.16070137C>A	16070137	16070137	ZK278	C>A	C	A
NC_000024.9:g.16074328C>T	16074328	16074328	ZK279	C>T	C	T

NC_000024.9:g.16190410G>C	16190410	16190410	ZK280	G>C	G	C
NC_000024.9:g.16197884A>C	16197884	16197884	ZK281	A>C	A	C
NC_000024.9:g.16211844T>G	16211844	16211844	ZK282	T>G	T	G
NC_000024.9:g.16226818C>A	16226818	16226818	ZK283	C>A	C	A
NC_000024.9:g.16244296G>C	16244296	16244296	ZK284	G>C	G	C
NC_000024.9:g.16247649C>T	16247649	16247649	ZK285	C>T	C	T
NC_000024.9:g.16259902G>A	16259902	16259902	ZK286	G>A	G	A
NC_000024.9:g.16281535T>C	16281535	16281535	ZK287	T>C	T	C
NC_000024.9:g.16288798T>A	16288798	16288798	ZK288	T>A	T	A
NC_000024.9:g.16307914T>C	16307914	16307914	ZK289	T>C	T	C
NC_000024.9:g.16308297G>C	16308297	16308297	ZK290	G>C	G	C
NC_000024.9:g.16310040C>A	16310040	16310040	ZK291	C>A	C	A
NC_000024.9:g.16332123A>C	16332123	16332123	ZK292	A>C	A	C
NC_000024.9:g.16332710C>T	16332710	16332710	ZK293	C>T	C	T
NC_000024.9:g.16344060A>G	16344060	16344060	ZK294	A>G	A	G
NC_000024.9:g.16346268C>T	16346268	16346268	ZK295	C>T	C	T
NC_000024.9:g.16347471G>A	16347471	16347471	ZK296	G>A	G	A
NC_000024.9:g.16373350G>C	16373350	16373350	ZK297	G>C	G	C
NC_000024.9:g.16377037A>G	16377037	16377037	ZK298	A>G	A	G
NC_000024.9:g.16386984T>A	16386984	16386984	ZK299	T>A	T	A
NC_000024.9:g.16415125C>G	16415125	16415125	ZK300	C>G	C	G
NC_000024.9:g.16418105C>T	16418105	16418105	ZK301	C>T	C	T
NC_000024.9:g.16424640A>G	16424640	16424640	ZK302	A>G	A	G
NC_000024.9:g.16439497A>G	16439497	16439497	ZK303	A>G	A	G
NC_000024.9:g.16441130C>T	16441130	16441130	ZK304	C>T	C	T
NC_000024.9:g.16441651C>T	16441651	16441651	ZK305	C>T	C	T
NC_000024.9:g.16444341A>G	16444341	16444341	ZK306	A>G	A	G
NC_000024.9:g.16445262C>T	16445262	16445262	ZK307	C>T	C	T
NC_000024.9:g.16448839T>C	16448839	16448839	ZK308	T>C	T	C
NC_000024.9:g.16452676G>A	16452676	16452676	ZK309	G>A	G	A
NC_000024.9:g.16464998G>A	16464998	16464998	ZK310	G>A	G	A
NC_000024.9:g.16465174G>A	16465174	16465174	ZK311	G>A	G	A
NC_000024.9:g.16497444T>A	16497444	16497444	ZK312	T>A	T	A
NC_000024.9:g.16505154A>T	16505154	16505154	ZK313	A>T	A	T
NC_000024.9:g.16525693A>G	16525693	16525693	ZK314	A>G	A	G
NC_000024.9:g.16529941G>C	16529941	16529941	ZK315	G>C	G	C
NC_000024.9:g.16537483C>T	16537483	16537483	ZK316	C>T	C	T
NC_000024.9:g.16543539G>A	16543539	16543539	ZK317	G>A	G	A
NC_000024.9:g.16550412G>A	16550412	16550412	ZK318	G>A	G	A
NC_000024.9:g.16568032C>A	16568032	16568032	ZK319	C>A	C	A
NC_000024.9:g.16574000A>G	16574000	16574000	ZK320	A>G	A	G
NC_000024.9:g.16576388G>C	16576388	16576388	ZK321	G>C	G	C
NC_000024.9:g.16585367G>A	16585367	16585367	ZK322	G>A	G	A
NC_000024.9:g.16587048C>T	16587048	16587048	ZK323	C>T	C	T
NC_000024.9:g.16587132A>G	16587132	16587132	ZK324	A>G	A	G
NC_000024.9:g.16597293T>C	16597293	16597293	ZK325	T>C	T	C
NC_000024.9:g.16615135T>C	16615135	16615135	ZK326	T>C	T	C
NC_000024.9:g.16619486C>T	16619486	16619486	ZK327	C>T	C	T
NC_000024.9:g.16634392G>A	16634392	16634392	ZK328	G>A	G	A
NC_000024.9:g.16688907A>G	16688907	16688907	ZK329	A>G	A	G
NC_000024.9:g.16693967A>G	16693967	16693967	ZK330	A>G	A	G
NC_000024.9:g.16715097G>A	16715097	16715097	ZK331	G>A	G	A
NC_000024.9:g.16731744C>T	16731744	16731744	ZK332	C>T	C	T
NC_000024.9:g.16735590G>A	16735590	16735590	ZK333	G>A	G	A
NC_000024.9:g.16738685A>G	16738685	16738685	ZK334	A>G	A	G
NC_000024.9:g.16756227A>C	16756227	16756227	ZK335	A>C	A	C
NC_000024.9:g.16779662C>A	16779662	16779662	ZK336	C>A	C	A
NC_000024.9:g.16787002T>C	16787002	16787002	ZK337	T>C	T	C
NC_000024.9:g.16791710G>A	16791710	16791710	ZK338	G>A	G	A
NC_000024.9:g.16791882A>G	16791882	16791882	ZK339	A>G	A	G
NC_000024.9:g.16794189A>G	16794189	16794189	ZK340	A>G	A	G
NC_000024.9:g.16797254C>A	16797254	16797254	ZK341	C>A	C	A
NC_000024.9:g.16801825G>T	16801825	16801825	ZK342	G>T	G	T
NC_000024.9:g.16802683C>A	16802683	16802683	ZK343	C>A	C	A
NC_000024.9:g.16810107T>G	16810107	16810107	ZK344	T>G	T	G
NC_000024.9:g.16812287T>C	16812287	16812287	ZK345	T>C	T	C
NC_000024.9:g.16813629G>A	16813629	16813629	ZK346	G>A	G	A
NC_000024.9:g.16820297T>A	16820297	16820297	ZK347	T>A	T	A
NC_000024.9:g.16822776C>A	16822776	16822776	ZK348	C>A	C	A
NC_000024.9:g.16829906G>A	16829906	16829906	ZK349	G>A	G	A
NC_000024.9:g.16838287A>G	16838287	16838287	ZK350	A>G	A	G
NC_000024.9:g.16839994T>C	16839994	16839994	ZK351	T>C	T	C
NC_000024.9:g.16861376T>A	16861376	16861376	ZK352	T>A	T	A
NC_000024.9:g.16861908G>A	16861908	16861908	ZK353	G>A	G	A
NC_000024.9:g.16882969C>T	16882969	16882969	ZK354	C>T	C	T
NC_000024.9:g.16887772T>C	16887772	16887772	ZK355	T>C	T	C
NC_000024.9:g.16893988T>C	16893988	16893988	ZK356	T>C	T	C
NC_000024.9:g.16894515A>C	16894515	16894515	ZK357	A>C	A	C
NC_000024.9:g.16899631T>C	16899631	16899631	ZK358	T>C	T	C
NC_000024.9:g.16921486C>T	16921486	16921486	ZK359	C>T	C	T
NC_000024.9:g.16929588A>G	16929588	16929588	ZK360	A>G	A	G
NC_000024.9:g.16932029T>C	16932029	16932029	ZK361	T>C	T	C
NC_000024.9:g.16950896C>T	16950896	16950896	ZK362	C>T	C	T
NC_000024.9:g.16968496A>G	16968496	16968496	ZK363	A>G	A	G
NC_000024.9:g.16979878C>T	16979878	16979878	ZK364	C>T	C	T
NC_000024.9:g.16984382G>A	16984382	16984382	ZK365	G>A	G	A
NC_000024.9:g.17009932C>T	17009932	17009932	ZK366	C>T	C	T
NC_000024.9:g.17010526A>G	17010526	17010526	ZK367	A>G	A	G
NC_000024.9:g.17015977C>T	17015977	17015977	ZK368	C>T	C	T
NC_000024.9:g.17018431T>C	17018431	17018431	ZK369	T>C	T	C
NC_000024.9:g.17043399A>G	17043399	17043399	ZK370	A>G	A	G
NC_000024.9:g.17051905T>G	17051905	17051905	ZK371	T>G	T	G
NC_000024.9:g.17080496C>G	17080496	17080496	ZK372	C>G	C	G
NC_000024.9:g.17080674T>C	17080674	17080674	ZK373	T>C	T	C
NC_000024.9:g.17090496G>C	17090496	17090496	ZK374	G>C	G	C
NC_000024.9:g.17099982C>A	17099982	17099982	ZK375	C>A	C	A
NC_000024.9:g.17129016C>T	17129016	17129016	ZK376	C>T	C	T
NC_000024.9:g.17137710G>C	17137710	17137710	ZK377	G>C	G	C
NC_000024.9:g.17137831G>C	17137831	17137831	ZK378	G>C	G	C
NC_000024.9:g.17148754G>A	17148754	17148754	ZK379	G>A	G	A
NC_000024.9:g.17153054G>A	17153054	17153054	ZK380	G>A	G	A
NC_000024.9:g.17208223C>T	17208223	17208223	ZK381	C>T	C	T
NC_000024.9:g.17236933C>T	17236933	17236933	ZK382	C>T	C	T
NC_000024.9:g.17253347T>C	17253347	17253347	ZK383	T>C	T	C

NC_000024.9:g.17257060A>G	17257060	17257060	ZK384	A>G	A	G
NC_000024.9:g.17264269G>A	17264269	17264269	ZK385	G>A	G	A
NC_000024.9:g.17272939C>T	17272939	17272939	ZK386	C>T	C	T
NC_000024.9:g.17273175A>G	17273175	17273175	ZK387	A>G	A	G
NC_000024.9:g.17295065T>C	17295065	17295065	ZK388	T>C	T	C
NC_000024.9:g.17309375T>C	17309375	17309375	ZK389	T>C	T	C
NC_000024.9:g.17310632G>A	17310632	17310632	ZK390	G>A	G	A
NC_000024.9:g.17310633C>T	17310633	17310633	ZK391	C>T	C	T
NC_000024.9:g.17311918G>C	17311918	17311918	ZK392	G>C	G	C
NC_000024.9:g.17315854C>T	17315854	17315854	ZK393	C>T	C	T
NC_000024.9:g.17340365A>G	17340365	17340365	ZK394	A>G	A	G
NC_000024.9:g.17343585A>G	17343585	17343585	ZK395	A>G	A	G
NC_000024.9:g.17350998G>A	17350998	17350998	ZK396	G>A	G	A
NC_000024.9:g.17391661G>C	17391661	17391661	ZK397	G>C	G	C
NC_000024.9:g.17421697A>G	17421697	17421697	ZK398	A>G	A	G
NC_000024.9:g.17424182T>G	17424182	17424182	ZK399	T>G	T	G
NC_000024.9:g.17426543G>A	17426543	17426543	ZK400	G>A	G	A
NC_000024.9:g.17444631T>C	17444631	17444631	ZK401	T>C	T	C
NC_000024.9:g.17446097G>A	17446097	17446097	ZK402	G>A	G	A
NC_000024.9:g.17459626C>T	17459626	17459626	ZK403	C>T	C	T
NC_000024.9:g.17465141A>G	17465141	17465141	ZK404	A>G	A	G
NC_000024.9:g.17475861T>C	17475861	17475861	ZK405	T>C	T	C
NC_000024.9:g.17482834G>A	17482834	17482834	ZK406	G>A	G	A
NC_000024.9:g.17486041T>A	17486041	17486041	ZK407	T>A	T	A
NC_000024.9:g.17486051A>G	17486051	17486051	ZK408	A>G	A	G
NC_000024.9:g.17487818C>T	17487818	17487818	ZK409	C>T	C	T
NC_000024.9:g.17491047T>C	17491047	17491047	ZK410	T>C	T	C
NC_000024.9:g.17493638A>C	17493638	17493638	ZK411	A>C	A	C
NC_000024.9:g.17510121A>G	17510121	17510121	ZK412	A>G	A	G
NC_000024.9:g.17510541G>A	17510541	17510541	ZK413	G>A	G	A
NC_000024.9:g.17512647G>T	17512647	17512647	ZK414	G>T	G	T
NC_000024.9:g.17517908T>A	17517908	17517908	ZK415	T>A	T	A
NC_000024.9:g.17525116G>T	17525116	17525116	ZK416	G>T	G	T
NC_000024.9:g.17525178A>G	17525178	17525178	ZK417	A>G	A	G
NC_000024.9:g.17525896A>G	17525896	17525896	ZK418	A>G	A	G
NC_000024.9:g.17544200G>A	17544200	17544200	ZK419	G>A	G	A
NC_000024.9:g.17553260G>T	17553260	17553260	ZK420	G>T	G	T
NC_000024.9:g.17555003C>T	17555003	17555003	ZK421	C>T	C	T
NC_000024.9:g.17561183A>T	17561183	17561183	ZK422	A>T	A	T
NC_000024.9:g.17561751C>T	17561751	17561751	ZK423	C>T	C	T
NC_000024.9:g.17565498C>A	17565498	17565498	ZK424	C>A	C	A
NC_000024.9:g.17568570T>A	17568570	17568570	ZK425	T>A	T	A
NC_000024.9:g.17578964C>G	17578964	17578964	ZK426	C>G	C	G
NC_000024.9:g.17592561G>A	17592561	17592561	ZK427	G>A	G	A
NC_000024.9:g.17592711T>A	17592711	17592711	ZK428	T>A	T	A
NC_000024.9:g.17637386G>A	17637386	17637386	ZK429	G>A	G	A
NC_000024.9:g.17656230G>A	17656230	17656230	ZK430	G>A	G	A
NC_000024.9:g.17657210A>C	17657210	17657210	ZK431	A>C	A	C
NC_000024.9:g.17677899G>A	17677899	17677899	ZK432	G>A	G	A
NC_000024.9:g.17686771C>T	17686771	17686771	ZK433	C>T	C	T
NC_000024.9:g.17703110T>C	17703110	17703110	ZK434	T>C	T	C
NC_000024.9:g.17721853T>C	17721853	17721853	ZK435	T>C	T	C
NC_000024.9:g.17734141C>T	17734141	17734141	ZK436	C>T	C	T
NC_000024.9:g.17737284G>A	17737284	17737284	ZK437	G>A	G	A
NC_000024.9:g.17740068G>A	17740068	17740068	ZK438	G>A	G	A
NC_000024.9:g.17756143C>T	17756143	17756143	ZK439	C>T	C	T
NC_000024.9:g.17758795C>T	17758795	17758795	ZK440	C>T	C	T
NC_000024.9:g.17759788C>T	17759788	17759788	ZK441	C>T	C	T
NC_000024.9:g.17761370G>T	17761370	17761370	ZK442	G>T	G	T
NC_000024.9:g.17769319A>G	17769319	17769319	ZK443	A>G	A	G
NC_000024.9:g.17775767T>A	17775767	17775767	ZK444	T>A	T	A
NC_000024.9:g.17777227G>A	17777227	17777227	ZK445	G>A	G	A
NC_000024.9:g.17778273T>C	17778273	17778273	ZK446	T>C	T	C
NC_000024.9:g.17800961A>G	17800961	17800961	ZK447	A>G	A	G
NC_000024.9:g.17805525T>A	17805525	17805525	ZK448	T>A	T	A
NC_000024.9:g.17818846A>T	17818846	17818846	ZK449	A>T	A	T
NC_000024.9:g.17821413C>A	17821413	17821413	ZK450	C>A	C	A
NC_000024.9:g.17848401C>T	17848401	17848401	ZK451	C>T	C	T
NC_000024.9:g.17854080C>G	17854080	17854080	ZK452	C>G	C	G
NC_000024.9:g.17878416A>G	17878416	17878416	ZK453	A>G	A	G
NC_000024.9:g.17883844G>A	17883844	17883844	ZK454	G>A	G	A
NC_000024.9:g.17895452G>A	17895452	17895452	ZK455	G>A	G	A
NC_000024.9:g.17897299G>C	17897299	17897299	ZK456	G>C	G	C
NC_000024.9:g.17906136A>C	17906136	17906136	ZK457	A>C	A	C
NC_000024.9:g.17919278A>T	17919278	17919278	ZK458	A>T	A	T
NC_000024.9:g.17930410A>G	17930410	17930410	ZK459	A>G	A	G
NC_000024.9:g.17935938A>G	17935938	17935938	ZK460	A>G	A	G
NC_000024.9:g.17936126G>C	17936126	17936126	ZK461	G>C	G	C
NC_000024.9:g.17938371T>C	17938371	17938371	ZK462	T>C	T	C
NC_000024.9:g.17953209T>A	17953209	17953209	ZK463	T>A	T	A
NC_000024.9:g.17956820T>C	17956820	17956820	ZK464	T>C	T	C
NC_000024.9:g.17959134C>T	17959134	17959134	ZK465	C>T	C	T
NC_000024.9:g.17973117C>A	17973117	17973117	ZK466	C>A	C	A
NC_000024.9:g.17981164A>G	17981164	17981164	ZK467	A>G	A	G
NC_000024.9:g.17981939T>C	17981939	17981939	ZK468	T>C	T	C
NC_000024.9:g.18020515G>A	18020515	18020515	ZK469	G>A	G	A
NC_000024.9:g.18020888G>A	18020888	18020888	ZK470	G>A	G	A
NC_000024.9:g.18030739C>T	18030739	18030739	ZK471	C>T	C	T
NC_000024.9:g.18033758C>A	18033758	18033758	ZK472	C>A	C	A
NC_000024.9:g.18038439T>A	18038439	18038439	ZK473	T>A	T	A
NC_000024.9:g.18043979T>G	18043979	18043979	ZK474	T>G	T	G
NC_000024.9:g.18064730T>C	18064730	18064730	ZK475	T>C	T	C
NC_000024.9:g.18066740T>C	18066740	18066740	ZK476	T>C	T	C
NC_000024.9:g.18068210A>C	18068210	18068210	ZK477	A>C	A	C
NC_000024.9:g.18093204C>T	18093204	18093204	ZK478	C>T	C	T
NC_000024.9:g.18102758C>A	18102758	18102758	ZK479	C>A	C	A
NC_000024.9:g.18111738C>G	18111738	18111738	ZK480	C>G	C	G
NC_000024.9:g.18117477C>T	18117477	18117477	ZK481	C>T	C	T
NC_000024.9:g.18123246T>C	18123246	18123246	ZK482	T>C	T	C
NC_000024.9:g.18150866G>C	18150866	18150866	ZK483	G>C	G	C
NC_000024.9:g.18163110A>G	18163110	18163110	ZK484	A>G	A	G
NC_000024.9:g.18205437G>A	18205437	18205437	ZK485	G>A	G	A
NC_000024.9:g.18214199C>A	18214199	18214199	ZK486	C>A	C	A
NC_000024.9:g.18225471A>G	18225471	18225471	ZK487	A>G	A	G

NC_000024.9:g.18248180A>T	18248180	18248180	ZK488	A>T	A	T
NC_000024.9:g.18556839C>T	18556839	18556839	ZK489	C>T	C	T
NC_000024.9:g.18561612T>C	18561612	18561612	ZK490	T>C	T	C
NC_000024.9:g.18585268C>T	18585268	18585268	ZK491	C>T	C	T
NC_000024.9:g.18587230G>T	18587230	18587230	ZK492	G>T	G	T
NC_000024.9:g.18588998G>T	18588998	18588998	ZK493	G>T	G	T
NC_000024.9:g.18601241G>T	18601241	18601241	ZK494	G>T	G	T
NC_000024.9:g.18606456G>A	18606456	18606456	ZK495	G>A	G	A
NC_000024.9:g.18607898G>T	18607898	18607898	ZK496	G>T	G	T
NC_000024.9:g.18611652A>T	18611652	18611652	ZK497	A>T	A	T
NC_000024.9:g.18621058C>T	18621058	18621058	ZK498	C>T	C	T
NC_000024.9:g.18629128C>T	18629128	18629128	ZK499	C>T	C	T
NC_000024.9:g.18637741T>C	18637741	18637741	ZK500	T>C	T	C
NC_000024.9:g.18653813T>C	18653813	18653813	ZK501	T>C	T	C
NC_000024.9:g.18655369A>G	18655369	18655369	ZK502	A>G	A	G
NC_000024.9:g.18663674A>C	18663674	18663674	ZK503	A>C	A	C
NC_000024.9:g.18669683T>A	18669683	18669683	ZK504	T>A	T	A
NC_000024.9:g.18672228C>T	18672228	18672228	ZK505	C>T	C	T
NC_000024.9:g.18694035C>T	18694035	18694035	ZK506	C>T	C	T
NC_000024.9:g.18748671C>T	18748671	18748671	ZK507	C>T	C	T
NC_000024.9:g.18749460C>T	18749460	18749460	ZK508	C>T	C	T
NC_000024.9:g.18755844T>C	18755844	18755844	ZK509	T>C	T	C
NC_000024.9:g.18760897A>C	18760897	18760897	ZK510	A>C	A	C
NC_000024.9:g.18761070A>C	18761070	18761070	ZK511	A>C	A	C
NC_000024.9:g.18764318A>T	18764318	18764318	ZK512	A>T	A	T
NC_000024.9:g.18771094C>T	18771094	18771094	ZK513	C>T	C	T
NC_000024.9:g.18803137T>C	18803137	18803137	ZK514	T>C	T	C
NC_000024.9:g.18803514A>T	18803514	18803514	ZK515	A>T	A	T
NC_000024.9:g.18805540G>A	18805540	18805540	ZK516	G>A	G	A
NC_000024.9:g.18807324C>T	18807324	18807324	ZK517	C>T	C	T
NC_000024.9:g.18813660G>A	18813660	18813660	ZK518	G>A	G	A
NC_000024.9:g.18819815T>C	18819815	18819815	ZK519	T>C	T	C
NC_000024.9:g.18823975G>C	18823975	18823975	ZK520	G>C	G	C
NC_000024.9:g.18834158T>C	18834158	18834158	ZK521	T>C	T	C
NC_000024.9:g.18889864T>C	18889864	18889864	ZK522	T>C	T	C
NC_000024.9:g.18901563T>C	18901563	18901563	ZK523	T>C	T	C
NC_000024.9:g.18904313G>A	18904313	18904313	ZK524	G>A	G	A
NC_000024.9:g.18916960G>A	18916960	18916960	ZK525	G>A	G	A
NC_000024.9:g.18926116G>T	18926116	18926116	ZK526	G>T	G	T
NC_000024.9:g.18931349A>G	18931349	18931349	ZK527	A>G	A	G
NC_000024.9:g.18955581A>C	18955581	18955581	ZK528	A>C	A	C
NC_000024.9:g.18962959T>A	18962959	18962959	ZK529	T>A	T	A
NC_000024.9:g.18963184C>A	18963184	18963184	ZK530	C>A	C	A
NC_000024.9:g.18965038C>T	18965038	18965038	ZK531	C>T	C	T
NC_000024.9:g.18977455C>T	18977455	18977455	ZK532	C>T	C	T
NC_000024.9:g.18999012G>T	18999012	18999012	ZK533	G>T	G	T
NC_000024.9:g.19010175G>A	19010175	19010175	ZK534	G>A	G	A
NC_000024.9:g.19031920C>T	19031920	19031920	ZK535	C>T	C	T
NC_000024.9:g.19035287C>A	19035287	19035287	ZK536	C>A	C	A
NC_000024.9:g.19038682C>T	19038682	19038682	ZK537	C>T	C	T
NC_000024.9:g.19046368C>T	19046368	19046368	ZK538	C>T	C	T
NC_000024.9:g.19056948A>T	19056948	19056948	ZK539	A>T	A	T
NC_000024.9:g.19067285C>A	19067285	19067285	ZK540	C>A	C	A
NC_000024.9:g.19068228C>A	19068228	19068228	ZK541	C>A	C	A
NC_000024.9:g.19076412C>T	19076412	19076412	ZK542	C>T	C	T
NC_000024.9:g.19077515T>C	19077515	19077515	ZK543	T>C	T	C
NC_000024.9:g.19110361G>A	19110361	19110361	ZK544	G>A	G	A
NC_000024.9:g.19111718C>T	19111718	19111718	ZK545	C>T	C	T
NC_000024.9:g.19112785C>A	19112785	19112785	ZK546	C>A	C	A
NC_000024.9:g.19114321C>A	19114321	19114321	ZK547	C>A	C	A
NC_000024.9:g.19115633C>T	19115633	19115633	ZK548	C>T	C	T
NC_000024.9:g.19117231T>C	19117231	19117231	ZK549	T>C	T	C
NC_000024.9:g.19118352C>T	19118352	19118352	ZK550	C>T	C	T
NC_000024.9:g.19134315G>A	19134315	19134315	ZK551	G>A	G	A
NC_000024.9:g.19143833T>C	19143833	19143833	ZK552	T>C	T	C
NC_000024.9:g.19147543A>G	19147543	19147543	ZK553	A>G	A	G
NC_000024.9:g.19149722T>G	19149722	19149722	ZK554	T>G	T	G
NC_000024.9:g.19151393A>G	19151393	19151393	ZK555	A>G	A	G
NC_000024.9:g.19158271A>G	19158271	19158271	ZK556	A>G	A	G
NC_000024.9:g.19162042T>C	19162042	19162042	ZK557	T>C	T	C
NC_000024.9:g.19168560A>T	19168560	19168560	ZK558	A>T	A	T
NC_000024.9:g.19186545A>G	19186545	19186545	ZK559	A>G	A	G
NC_000024.9:g.19202316G>T	19202316	19202316	ZK560	G>T	G	T
NC_000024.9:g.19209158T>C	19209158	19209158	ZK561	T>C	T	C
NC_000024.9:g.19211922A>T	19211922	19211922	ZK562	A>T	A	T
NC_000024.9:g.19213696C>T	19213696	19213696	ZK563	C>T	C	T
NC_000024.9:g.19218761C>G	19218761	19218761	ZK564	C>G	C	G
NC_000024.9:g.19221761C>A	19221761	19221761	ZK565	C>A	C	A
NC_000024.9:g.19222238G>A	19222238	19222238	ZK566	G>A	G	A
NC_000024.9:g.19225368T>A	19225368	19225368	ZK567	T>A	T	A
NC_000024.9:g.19228272G>T	19228272	19228272	ZK568	G>T	G	T
NC_000024.9:g.19231880C>T	19231880	19231880	ZK569	C>T	C	T
NC_000024.9:g.19260629C>T	19260629	19260629	ZK570	C>T	C	T
NC_000024.9:g.19262420A>G	19262420	19262420	ZK571	A>G	A	G
NC_000024.9:g.19265139G>A	19265139	19265139	ZK572	G>A	G	A
NC_000024.9:g.19275913T>C	19275913	19275913	ZK573	T>C	T	C
NC_000024.9:g.19288914C>A	19288914	19288914	ZK574	C>A	C	A
NC_000024.9:g.19300451C>A	19300451	19300451	ZK575	C>A	C	A
NC_000024.9:g.19305914C>A	19305914	19305914	ZK576	C>A	C	A
NC_000024.9:g.19306092C>T	19306092	19306092	ZK577	C>T	C	T
NC_000024.9:g.19311408C>T	19311408	19311408	ZK578	C>T	C	T
NC_000024.9:g.19312033G>A	19312033	19312033	ZK579	G>A	G	A
NC_000024.9:g.19332983G>T	19332983	19332983	ZK580	G>T	G	T
NC_000024.9:g.19347737A>G	19347737	19347737	ZK581	A>G	A	G
NC_000024.9:g.19360066C>T	19360066	19360066	ZK582	C>T	C	T
NC_000024.9:g.19364396T>C	19364396	19364396	ZK583	T>C	T	C
NC_000024.9:g.19368226C>T	19368226	19368226	ZK584	C>T	C	T
NC_000024.9:g.19368791G>A	19368791	19368791	ZK585	G>A	G	A
NC_000024.9:g.19371878A>G	19371878	19371878	ZK586	A>G	A	G
NC_000024.9:g.19374684G>T	19374684	19374684	ZK587	G>T	G	T
NC_000024.9:g.19412199T>C	19412199	19412199	ZK588	T>C	T	C
NC_000024.9:g.19415891C>A	19415891	19415891	ZK589	C>A	C	A
NC_000024.9:g.19418824C>G	19418824	19418824	ZK590	C>G	C	G
NC_000024.9:g.19418876T>C	19418876	19418876	ZK591	T>C	T	C

NC_000024.9:g.19419881A>G	19419881	19419881	ZK592	A>G	A	G
NC_000024.9:g.19430219A>G	19430219	19430219	ZK593	A>G	A	G
NC_000024.9:g.19439332T>A	19439332	19439332	ZK594	T>A	T	A
NC_000024.9:g.19443035T>C	19443035	19443035	ZK595	T>C	T	C
NC_000024.9:g.19444598A>G	19444598	19444598	ZK596	A>G	A	G
NC_000024.9:g.19458477A>G	19458477	19458477	ZK597	A>G	A	G
NC_000024.9:g.19468167T>C	19468167	19468167	ZK598	T>C	T	C
NC_000024.9:g.19475900C>T	19475900	19475900	ZK599	C>T	C	T
NC_000024.9:g.19477223G>C	19477223	19477223	ZK600	G>C	G	C
NC_000024.9:g.19500657C>G	19500657	19500657	ZK601	C>G	C	G
NC_000024.9:g.19503939C>A	19503939	19503939	ZK602	C>A	C	A
NC_000024.9:g.19509831A>G	19509831	19509831	ZK603	A>G	A	G
NC_000024.9:g.19514377G>A	19514377	19514377	ZK604	G>A	G	A
NC_000024.9:g.19515484A>G	19515484	19515484	ZK605	A>G	A	G
NC_000024.9:g.19542973A>C	19542973	19542973	ZK606	A>C	A	C
NC_000024.9:g.19545964C>G	19545964	19545964	ZK607	C>G	C	G
NC_000024.9:g.21034173C>T	21034173	21034173	ZK608	C>T	C	T
NC_000024.9:g.21039034T>C	21039034	21039034	ZK609	T>C	T	C
NC_000024.9:g.21039235C>T	21039235	21039235	ZK610	C>T	C	T
NC_000024.9:g.21067404C>T	21067404	21067404	ZK611	C>T	C	T
NC_000024.9:g.21071425G>A	21071425	21071425	ZK612	G>A	G	A
NC_000024.9:g.21098366T>C	21098366	21098366	ZK613	T>C	T	C
NC_000024.9:g.21124558C>T	21124558	21124558	ZK614	C>T	C	T
NC_000024.9:g.21134779T>G	21134779	21134779	ZK615	T>G	T	G
NC_000024.9:g.21163614A>G	21163614	21163614	ZK616	A>G	A	G
NC_000024.9:g.21194893G>C	21194893	21194893	ZK617	G>C	G	C
NC_000024.9:g.21201620T>G	21201620	21201620	ZK618	T>G	T	G
NC_000024.9:g.21209569G>A	21209569	21209569	ZK619	G>A	G	A
NC_000024.9:g.21217995A>G	21217995	21217995	ZK620	A>G	A	G
NC_000024.9:g.21248406G>A	21248406	21248406	ZK621	G>A	G	A
NC_000024.9:g.21249916G>C	21249916	21249916	ZK622	G>C	G	C
NC_000024.9:g.21251855T>C	21251855	21251855	ZK623	T>C	T	C
NC_000024.9:g.21259334G>C	21259334	21259334	ZK624	G>C	G	C
NC_000024.9:g.21264162G>T	21264162	21264162	ZK625	G>T	G	T
NC_000024.9:g.21276902C>A	21276902	21276902	ZK626	C>A	C	A
NC_000024.9:g.21284549G>A	21284549	21284549	ZK627	G>A	G	A
NC_000024.9:g.21296621A>G	21296621	21296621	ZK628	A>G	A	G
NC_000024.9:g.21301586G>A	21301586	21301586	ZK629	G>A	G	A
NC_000024.9:g.21307812A>G	21307812	21307812	ZK630	A>G	A	G
NC_000024.9:g.21316202T>A	21316202	21316202	ZK631	T>A	T	A
NC_000024.9:g.21341242G>A	21341242	21341242	ZK632	G>A	G	A
NC_000024.9:g.21344275C>T	21344275	21344275	ZK633	C>T	C	T
NC_000024.9:g.21346237C>T	21346237	21346237	ZK634	C>T	C	T
NC_000024.9:g.21386585G>T	21386585	21386585	ZK635	G>T	G	T
NC_000024.9:g.21387673G>T	21387673	21387673	ZK636	G>T	G	T
NC_000024.9:g.21398480A>T	21398480	21398480	ZK637	A>T	A	T
NC_000024.9:g.21399587A>G	21399587	21399587	ZK638	A>G	A	G
NC_000024.9:g.21401174G>A	21401174	21401174	ZK639	G>A	G	A
NC_000024.9:g.21411342A>T	21411342	21411342	ZK640	A>T	A	T
NC_000024.9:g.21458552C>T	21458552	21458552	ZK641	C>T	C	T
NC_000024.9:g.21458851T>C	21458851	21458851	ZK642	T>C	T	C
NC_000024.9:g.21465586T>A	21465586	21465586	ZK643	T>A	T	A
NC_000024.9:g.21470483G>A	21470483	21470483	ZK644	G>A	G	A
NC_000024.9:g.21473117A>G	21473117	21473117	ZK645	A>G	A	G
NC_000024.9:g.21491055A>G	21491055	21491055	ZK646	A>G	A	G
NC_000024.9:g.21533653C>T	21533653	21533653	ZK647	C>T	C	T
NC_000024.9:g.21548080G>C	21548080	21548080	ZK648	G>C	G	C
NC_000024.9:g.21566459G>A	21566459	21566459	ZK649	G>A	G	A
NC_000024.9:g.21569440G>A	21569440	21569440	ZK650	G>A	G	A
NC_000024.9:g.21571017C>A	21571017	21571017	ZK651	C>A	C	A
NC_000024.9:g.21582606A>G	21582606	21582606	ZK652	A>G	A	G
NC_000024.9:g.21588311A>G	21588311	21588311	ZK653	A>G	A	G
NC_000024.9:g.21590650G>T	21590650	21590650	ZK654	G>T	G	T
NC_000024.9:g.21590731T>C	21590731	21590731	ZK655	T>C	T	C
NC_000024.9:g.21595351A>T	21595351	21595351	ZK656	A>T	A	T
NC_000024.9:g.21597940A>G	21597940	21597940	ZK657	A>G	A	G
NC_000024.9:g.21608866G>C	21608866	21608866	ZK658	G>C	G	C
NC_000024.9:g.21611215A>G	21611215	21611215	ZK659	A>G	A	G
NC_000024.9:g.21612068A>G	21612068	21612068	ZK660	A>G	A	G
NC_000024.9:g.21619516G>T	21619516	21619516	ZK661	G>T	G	T
NC_000024.9:g.21620722C>T	21620722	21620722	ZK662	C>T	C	T
NC_000024.9:g.21640599C>T	21640599	21640599	ZK663	C>T	C	T
NC_000024.9:g.21661110T>A	21661110	21661110	ZK664	T>A	T	A
NC_000024.9:g.21672600A>G	21672600	21672600	ZK665	A>G	A	G
NC_000024.9:g.21672970G>A	21672970	21672970	ZK666	G>A	G	A
NC_000024.9:g.21682947T>C	21682947	21682947	ZK667	T>C	T	C
NC_000024.9:g.21685805T>C	21685805	21685805	ZK668	T>C	T	C
NC_000024.9:g.21688515A>G	21688515	21688515	ZK669	A>G	A	G
NC_000024.9:g.21690622G>A	21690622	21690622	ZK670	G>A	G	A
NC_000024.9:g.21696224G>C	21696224	21696224	ZK671	G>C	G	C
NC_000024.9:g.21702445G>T	21702445	21702445	ZK672	G>T	G	T
NC_000024.9:g.21704282A>G	21704282	21704282	ZK673	A>G	A	G
NC_000024.9:g.21707788T>C	21707788	21707788	ZK674	T>C	T	C
NC_000024.9:g.21712077A>G	21712077	21712077	ZK675	A>G	A	G
NC_000024.9:g.21712919A>C	21712919	21712919	ZK676	A>C	A	C
NC_000024.9:g.21729331T>C	21729331	21729331	ZK677	T>C	T	C
NC_000024.9:g.21733598C>T	21733598	21733598	ZK678	C>T	C	T
NC_000024.9:g.21753091G>C	21753091	21753091	ZK679	G>C	G	C
NC_000024.9:g.21755138T>C	21755138	21755138	ZK680	T>C	T	C
NC_000024.9:g.21771406T>C	21771406	21771406	ZK681	T>C	T	C
NC_000024.9:g.21777989C>T	21777989	21777989	ZK682	C>T	C	T
NC_000024.9:g.21779552T>G	21779552	21779552	ZK683	T>G	T	G
NC_000024.9:g.21798573G>T	21798573	21798573	ZK684	G>T	G	T
NC_000024.9:g.21802022A>T	21802022	21802022	ZK685	A>T	A	T
NC_000024.9:g.21813019C>T	21813019	21813019	ZK686	C>T	C	T
NC_000024.9:g.21819840G>A	21819840	21819840	ZK687	G>A	G	A
NC_000024.9:g.21820177G>A	21820177	21820177	ZK688	G>A	G	A
NC_000024.9:g.21822217C>A	21822217	21822217	ZK689	C>A	C	A
NC_000024.9:g.21828185C>T	21828185	21828185	ZK690	C>T	C	T
NC_000024.9:g.21828194A>G	21828194	21828194	ZK691	A>G	A	G
NC_000024.9:g.21828485T>G	21828485	21828485	ZK692	T>G	T	G
NC_000024.9:g.21838439A>C	21838439	21838439	ZK693	A>C	A	C
NC_000024.9:g.21858390T>A	21858390	21858390	ZK694	T>A	T	A
NC_000024.9:g.21859185T>G	21859185	21859185	ZK695	T>G	T	G

NC_000024.9:g.21869495C>T	21869495	21869495	ZK696	C>T	C	T
NC_000024.9:g.21869612C>G	21869612	21869612	ZK697	C>G	C	G
NC_000024.9:g.21870865T>C	21870865	21870865	ZK698	T>C	T	C
NC_000024.9:g.21907282A>G	21907282	21907282	ZK699	A>G	A	G
NC_000024.9:g.21927630A>G	21927630	21927630	ZK700	A>G	A	G
NC_000024.9:g.21927907T>G	21927907	21927907	ZK701	T>G	T	G
NC_000024.9:g.21972020C>A	21972020	21972020	ZK702	C>A	C	A
NC_000024.9:g.21980574T>G	21980574	21980574	ZK703	T>G	T	G
NC_000024.9:g.21993405T>C	21993405	21993405	ZK704	T>C	T	C
NC_000024.9:g.22031045G>T	22031045	22031045	ZK705	G>T	G	T
NC_000024.9:g.22033793C>A	22033793	22033793	ZK706	C>A	C	A
NC_000024.9:g.22071723T>C	22071723	22071723	ZK707	T>C	T	C
NC_000024.9:g.22072502C>T	22072502	22072502	ZK708	C>T	C	T
NC_000024.9:g.22089722T>C	22089722	22089722	ZK709	T>C	T	C
NC_000024.9:g.22112888T>C	22112888	22112888	ZK710	T>C	T	C
NC_000024.9:g.22127143A>G	22127143	22127143	ZK711	A>G	A	G
NC_000024.9:g.22132960A>G	22132960	22132960	ZK712	A>G	A	G
NC_000024.9:g.22141731C>G	22141731	22141731	ZK713	C>G	C	G
NC_000024.9:g.22147525G>A	22147525	22147525	ZK714	G>A	G	A
NC_000024.9:g.22148802C>T	22148802	22148802	ZK715	C>T	C	T
NC_000024.9:g.22165801G>A	22165801	22165801	ZK716	G>A	G	A
NC_000024.9:g.22184202G>A	22184202	22184202	ZK717	G>A	G	A
NC_000024.9:g.22184405C>A	22184405	22184405	ZK718	C>A	C	A
NC_000024.9:g.22188910C>T	22188910	22188910	ZK719	C>T	C	T
NC_000024.9:g.22190491T>C	22190491	22190491	ZK720	T>C	T	C
NC_000024.9:g.22515443T>C	22515443	22515443	ZK721	T>C	T	C
NC_000024.9:g.22517181A>G	22517181	22517181	ZK722	A>G	A	G
NC_000024.9:g.22549899G>C	22549899	22549899	ZK723	G>C	G	C
NC_000024.9:g.22561620C>G	22561620	22561620	ZK724	C>G	C	G
NC_000024.9:g.22565162T>C	22565162	22565162	ZK725	T>C	T	C
NC_000024.9:g.22575689G>A	22575689	22575689	ZK726	G>A	G	A
NC_000024.9:g.22589365T>A	22589365	22589365	ZK727	T>A	T	A
NC_000024.9:g.22592871C>T	22592871	22592871	ZK728	C>T	C	T
NC_000024.9:g.22595652C>T	22595652	22595652	ZK729	C>T	C	T
NC_000024.9:g.22595987G>T	22595987	22595987	ZK730	G>T	G	T
NC_000024.9:g.22599902T>G	22599902	22599902	ZK731	T>G	T	G
NC_000024.9:g.22604097T>C	22604097	22604097	ZK732	T>C	T	C
NC_000024.9:g.22604845T>C	22604845	22604845	ZK733	T>C	T	C
NC_000024.9:g.22607952A>G	22607952	22607952	ZK734	A>G	A	G
NC_000024.9:g.22613374G>A	22613374	22613374	ZK735	G>A	G	A
NC_000024.9:g.22616519C>T	22616519	22616519	ZK736	C>T	C	T
NC_000024.9:g.22629063C>A	22629063	22629063	ZK737	C>A	C	A
NC_000024.9:g.22656784G>T	22656784	22656784	ZK738	G>T	G	T
NC_000024.9:g.22657845C>T	22657845	22657845	ZK739	C>T	C	T
NC_000024.9:g.22669654C>A	22669654	22669654	ZK740	C>A	C	A
NC_000024.9:g.22669895G>A	22669895	22669895	ZK741	G>A	G	A
NC_000024.9:g.22672668G>C	22672668	22672668	ZK742	G>C	G	C
NC_000024.9:g.22679773G>A	22679773	22679773	ZK743	G>A	G	A
NC_000024.9:g.22686911T>C	22686911	22686911	ZK744	T>C	T	C
NC_000024.9:g.22717966C>G	22717966	22717966	ZK745	C>G	C	G
NC_000024.9:g.22720427G>A	22720427	22720427	ZK746	G>A	G	A
NC_000024.9:g.22721243A>G	22721243	22721243	ZK747	A>G	A	G
NC_000024.9:g.22747941G>T	22747941	22747941	ZK748	G>T	G	T
NC_000024.9:g.22748170C>T	22748170	22748170	ZK749	C>T	C	T
NC_000024.9:g.22750912G>C	22750912	22750912	ZK750	G>C	G	C
NC_000024.9:g.22786171C>T	22786171	22786171	ZK751	C>T	C	T
NC_000024.9:g.22797173A>G	22797173	22797173	ZK752	A>G	A	G
NC_000024.9:g.22802061T>C	22802061	22802061	ZK753	T>C	T	C
NC_000024.9:g.22802596G>A	22802596	22802596	ZK754	G>A	G	A
NC_000024.9:g.22807434G>A	22807434	22807434	ZK755	G>A	G	A
NC_000024.9:g.22809769C>T	22809769	22809769	ZK756	C>T	C	T
NC_000024.9:g.22812979G>A	22812979	22812979	ZK757	G>A	G	A
NC_000024.9:g.22816700G>A	22816700	22816700	ZK758	G>A	G	A
NC_000024.9:g.22816852A>T	22816852	22816852	ZK759	A>T	A	T
NC_000024.9:g.22817718C>G	22817718	22817718	ZK760	C>G	C	G
NC_000024.9:g.22829113C>A	22829113	22829113	ZK761	C>A	C	A
NC_000024.9:g.22830034C>A	22830034	22830034	ZK762	C>A	C	A
NC_000024.9:g.22845567T>A	22845567	22845567	ZK763	T>A	T	A
NC_000024.9:g.22846566G>T	22846566	22846566	ZK764	G>T	G	T
NC_000024.9:g.22855199G>T	22855199	22855199	ZK765	G>T	G	T
NC_000024.9:g.22863578C>A	22863578	22863578	ZK766	C>A	C	A
NC_000024.9:g.22879474T>C	22879474	22879474	ZK767	T>C	T	C
NC_000024.9:g.22879545G>A	22879545	22879545	ZK768	G>A	G	A
NC_000024.9:g.22898982T>C	22898982	22898982	ZK769	T>C	T	C
NC_000024.9:g.22902031C>T	22902031	22902031	ZK770	C>T	C	T
NC_000024.9:g.22905905C>T	22905905	22905905	ZK771	C>T	C	T
NC_000024.9:g.22908968A>C	22908968	22908968	ZK772	A>C	A	C
NC_000024.9:g.22911311G>A	22911311	22911311	ZK773	G>A	G	A
NC_000024.9:g.22923682A>G	22923682	22923682	ZK774	A>G	A	G
NC_000024.9:g.22927185C>T	22927185	22927185	ZK775	C>T	C	T
NC_000024.9:g.22928146A>G	22928146	22928146	ZK776	A>G	A	G
NC_000024.9:g.22930230G>A	22930230	22930230	ZK777	G>A	G	A
NC_000024.9:g.22949029T>C	22949029	22949029	ZK778	T>C	T	C
NC_000024.9:g.22957137C>T	22957137	22957137	ZK779	C>T	C	T
NC_000024.9:g.22957161T>C	22957161	22957161	ZK780	T>C	T	C
NC_000024.9:g.22967001A>G	22967001	22967001	ZK781	A>G	A	G
NC_000024.9:g.22991404C>G	22991404	22991404	ZK782	C>G	C	G
NC_000024.9:g.23024010C>T	23024010	23024010	ZK783	C>T	C	T
NC_000024.9:g.23048223A>G	23048223	23048223	ZK784	A>G	A	G
NC_000024.9:g.23054336A>C	23054336	23054336	ZK785	A>C	A	C
NC_000024.9:g.23058372T>C	23058372	23058372	ZK786	T>C	T	C
NC_000024.9:g.23065342G>T	23065342	23065342	ZK787	G>T	G	T
NC_000024.9:g.23072237G>A	23072237	23072237	ZK788	G>A	G	A
NC_000024.9:g.23080296G>A	23080296	23080296	ZK789	G>A	G	A
NC_000024.9:g.23090450G>A	23090450	23090450	ZK790	G>A	G	A
NC_000024.9:g.23093551A>T	23093551	23093551	ZK791	A>T	A	T
NC_000024.9:g.23116554C>T	23116554	23116554	ZK792	C>T	C	T
NC_000024.9:g.23126574A>T	23126574	23126574	ZK793	A>T	A	T
NC_000024.9:g.23134579C>T	23134579	23134579	ZK794	C>T	C	T
NC_000024.9:g.23137984C>T	23137984	23137984	ZK795	C>T	C	T
NC_000024.9:g.23138197G>T	23138197	23138197	ZK796	G>T	G	T
NC_000024.9:g.23141324A>T	23141324	23141324	ZK797	A>T	A	T
NC_000024.9:g.23150536C>T	23150536	23150536	ZK798	C>T	C	T
NC_000024.9:g.23151335C>T	23151335	23151335	ZK799	C>T	C	T

NC_000024.9:g.23162485A>T	23162485	23162485	ZK800	A>T	A	T
NC_000024.9:g.23162820T>C	23162820	23162820	ZK801	T>C	T	C
NC_000024.9:g.23164546T>A	23164546	23164546	ZK802	T>A	T	A
NC_000024.9:g.23165077G>C	23165077	23165077	ZK803	G>C	G	C
NC_000024.9:g.23195012T>C	23195012	23195012	ZK804	T>C	T	C
NC_000024.9:g.23200684T>C	23200684	23200684	ZK805	T>C	T	C
NC_000024.9:g.23201706A>C	23201706	23201706	ZK806	A>C	A	C
NC_000024.9:g.23206756C>T	23206756	23206756	ZK807	C>T	C	T
NC_000024.9:g.23207306A>T	23207306	23207306	ZK808	A>T	A	T
NC_000024.9:g.23234997A>G	23234997	23234997	ZK809	A>G	A	G
NC_000024.9:g.23238783C>T	23238783	23238783	ZK810	C>T	C	T
NC_000024.9:g.23240659T>C	23240659	23240659	ZK811	T>C	T	C
NC_000024.9:g.23243689A>T	23243689	23243689	ZK812	A>T	A	T
NC_000024.9:g.23246764G>A	23246764	23246764	ZK813	G>A	G	A
NC_000024.9:g.23247649T>C	23247649	23247649	ZK814	T>C	T	C
NC_000024.9:g.23252004A>G	23252004	23252004	ZK815	A>G	A	G
NC_000024.9:g.23261806A>C	23261806	23261806	ZK816	A>C	A	C
NC_000024.9:g.23261814C>T	23261814	23261814	ZK817	C>T	C	T
NC_000024.9:g.23266257T>G	23266257	23266257	ZK818	T>G	T	G
NC_000024.9:g.23281112T>C	23281112	23281112	ZK819	T>C	T	C
NC_000024.9:g.23291152A>T	23291152	23291152	ZK820	A>T	A	T
NC_000024.9:g.23296813A>T	23296813	23296813	ZK821	A>T	A	T
NC_000024.9:g.23313814A>T	23313814	23313814	ZK822	A>T	A	T
NC_000024.9:g.23323599C>G	23323599	23323599	ZK823	C>G	C	G
NC_000024.9:g.23328717C>G	23328717	23328717	ZK824	C>G	C	G
NC_000024.9:g.23364005T>G	23364005	23364005	ZK825	T>G	T	G
NC_000024.9:g.23369020C>G	23369020	23369020	ZK826	C>G	C	G
NC_000024.9:g.23371142C>T	23371142	23371142	ZK827	C>T	C	T
NC_000024.9:g.23376532G>A	23376532	23376532	ZK828	G>A	G	A
NC_000024.9:g.23384688G>A	23384688	23384688	ZK829	G>A	G	A
NC_000024.9:g.23406590C>T	23406590	23406590	ZK830	C>T	C	T
NC_000024.9:g.23408259A>G	23408259	23408259	ZK831	A>G	A	G
NC_000024.9:g.23429109G>C	23429109	23429109	ZK832	G>C	G	C
NC_000024.9:g.23450109C>G	23450109	23450109	ZK833	C>G	C	G
NC_000024.9:g.23456187C>T	23456187	23456187	ZK834	C>T	C	T
NC_000024.9:g.23456582T>C	23456582	23456582	ZK835	T>C	T	C
NC_000024.9:g.23471160A>G	23471160	23471160	ZK836	A>G	A	G
NC_000024.9:g.23484298A>G	23484298	23484298	ZK837	A>G	A	G
NC_000024.9:g.23492233C>T	23492233	23492233	ZK838	C>T	C	T
NC_000024.9:g.23493833C>T	23493833	23493833	ZK839	C>T	C	T
NC_000024.9:g.23494094T>C	23494094	23494094	ZK840	T>C	T	C
NC_000024.9:g.28471573G>A	28471573	28471573	ZK841	G>A	G	A
NC_000024.9:g.28492421T>C	28492421	28492421	ZK842	T>C	T	C
NC_000024.9:g.28493788T>C	28493788	28493788	ZK843	T>C	T	C
NC_000024.9:g.28494905C>T	28494905	28494905	ZK844	C>T	C	T
NC_000024.9:g.28504671C>A	28504671	28504671	ZK845	C>A	C	A
NC_000024.9:g.28514783T>C	28514783	28514783	ZK846	T>C	T	C
NC_000024.9:g.28564838C>T	28564838	28564838	ZK847	C>T	C	T
NC_000024.9:g.28573346A>G	28573346	28573346	ZK848	A>G	A	G
NC_000024.9:g.28590944G>A	28590944	28590944	ZK849	G>A	G	A
NC_000024.9:g.28594608G>A	28594608	28594608	ZK850	G>A	G	A
NC_000024.9:g.28597578C>T	28597578	28597578	ZK851	C>T	C	T
NC_000024.9:g.28626592G>T	28626592	28626592	ZK852	G>T	G	T
NC_000024.9:g.28639650G>C	28639650	28639650	ZK853	G>C	G	C
NC_000024.9:g.28645805C>T	28645805	28645805	ZK854	C>T	C	T
NC_000024.9:g.28647373C>T	28647373	28647373	ZK855	C>T	C	T
NC_000024.9:g.28655870A>G	28655870	28655870	ZK856	A>G	A	G
NC_000024.9:g.28656647C>G	28656647	28656647	ZK857	C>G	C	G
NC_000024.9:g.28659637C>A	28659637	28659637	ZK858	C>A	C	A
NC_000024.9:g.28660122A>T	28660122	28660122	ZK859	A>T	A	T
NC_000024.9:g.28660615T>C	28660615	28660615	ZK860	T>C	T	C
NC_000024.9:g.28661287C>T	28661287	28661287	ZK861	C>T	C	T
NC_000024.9:g.28686825T>C	28686825	28686825	ZK862	T>C	T	C
NC_000024.9:g.28701907T>C	28701907	28701907	ZK863	T>C	T	C
NC_000024.9:g.28705794T>G	28705794	28705794	ZK864	T>G	T	G
NC_000024.9:g.28709046C>T	28709046	28709046	ZK865	C>T	C	T
NC_000024.9:g.28709455A>T	28709455	28709455	ZK866	A>T	A	T
NC_000024.9:g.28711344C>A	28711344	28711344	ZK867	C>A	C	A
NC_000024.9:g.28718622G>A	28718622	28718622	ZK868	G>A	G	A
NC_000024.9:g.28732697A>G	28732697	28732697	ZK869	A>G	A	G
NC_000024.9:g.28732794A>G	28732794	28732794	ZK870	A>G	A	G
NC_000024.9:g.28738597A>G	28738597	28738597	ZK871	A>G	A	G
NC_000024.9:g.28747275A>G	28747275	28747275	ZK872	A>G	A	G
NC_000024.9:g.28756242G>A	28756242	28756242	ZK873	G>A	G	A
NC_000024.9:g.28758144C>T	28758144	28758144	ZK874	C>T	C	T
NC_000024.9:g.28764915G>A	28764915	28764915	ZK875	G>A	G	A
NC_000024.9:g.28766686G>A	28766686	28766686	ZK876	G>A	G	A
NC_000024.9:g.28766728C>T	28766728	28766728	ZK877	C>T	C	T
NC_000024.9:g.28774952T>C	28774952	28774952	ZK878	T>C	T	C
NC_000024.9:g.28775764C>T	28775764	28775764	ZK879	C>T	C	T
NC_000024.9:g.28776828T>A	28776828	28776828	ZK880	T>A	T	A
NC_000024.9:g.28777993G>A	28777993	28777993	ZK881	G>A	G	A
NC_000024.9:g.28781455G>C	28781455	28781455	ZK882	G>C	G	C
NC_000024.9:g.28782958T>C	28782958	28782958	ZK883	T>C	T	C
NC_000024.9:g.2794282C>T	2794282	2794282	ZK884	C>T	C	T
NC_000024.9:g.2821312T>G	2821312	2821312	ZK885	T>G	T	G
NC_000024.9:g.2855421A>C	2855421	2855421	ZK886	A>C	A	C
NC_000024.9:g.2866854A>G	2866854	2866854	ZK887	A>G	A	G
NC_000024.9:g.2875178C>A	2875178	2875178	ZK888	C>A	C	A
NC_000024.9:g.4841279C>T	4841279	4841279	ZK889	C>T	C	T
NC_000024.9:g.6618737G>T	6618737	6618737	ZK890	G>T	G	T
NC_000024.9:g.6645937C>T	6645937	6645937	ZK891	C>T	C	T
NC_000024.9:g.6664123T>C	6664123	6664123	ZK892	T>C	T	C
NC_000024.9:g.6668970T>C	6668970	6668970	ZK893	T>C	T	C
NC_000024.9:g.6696013A>G	6696013	6696013	ZK894	A>G	A	G
NC_000024.9:g.6731832C>G	6731832	6731832	ZK895	C>G	C	G
NC_000024.9:g.6774250T>C	6774250	6774250	ZK896	T>C	T	C
NC_000024.9:g.6857685T>G	6857685	6857685	ZK897	T>G	T	G
NC_000024.9:g.6897354C>T	6897354	6897354	ZK898	C>T	C	T
NC_000024.9:g.6900699C>T	6900699	6900699	ZK899	C>T	C	T
NC_000024.9:g.6940369G>T	6940369	6940369	ZK900	G>T	G	T
NC_000024.9:g.6975973G>A	6975973	6975973	ZK901	G>A	G	A
NC_000024.9:g.6996566T>C	6996566	6996566	ZK902	T>C	T	C
NC_000024.9:g.7042954G>A	7042954	7042954	ZK903	G>A	G	A

NC_000024.9:g.7043049G>A	7043049	7043049	ZK904	G>A	G	A
NC_000024.9:g.7048812G>A	7048812	7048812	ZK905	G>A	G	A
NC_000024.9:g.7085248T>A	7085248	7085248	ZK906	T>A	T	A
NC_000024.9:g.7136055G>C	7136055	7136055	ZK907	G>C	G	C
NC_000024.9:g.7179540A>C	7179540	7179540	ZK908	A>C	A	C
NC_000024.9:g.7185794A>G	7185794	7185794	ZK909	A>G	A	G
NC_000024.9:g.7210348T>C	7210348	7210348	ZK910	T>C	T	C
NC_000024.9:g.7246991T>G	7246991	7246991	ZK911	T>G	T	G
NC_000024.9:g.7283622G>A	7283622	7283622	ZK912	G>A	G	A
NC_000024.9:g.7286755G>T	7286755	7286755	ZK913	G>T	G	T
NC_000024.9:g.7295237G>A	7295237	7295237	ZK914	G>A	G	A
NC_000024.9:g.7315872C>G	7315872	7315872	ZK915	C>G	C	G
NC_000024.9:g.7328027G>A	7328027	7328027	ZK916	G>A	G	A
NC_000024.9:g.7350352G>T	7350352	7350352	ZK917	G>T	G	T
NC_000024.9:g.7369355A>C	7369355	7369355	ZK918	A>C	A	C
NC_000024.9:g.7372943C>A	7372943	7372943	ZK919	C>A	C	A
NC_000024.9:g.7379252G>A	7379252	7379252	ZK920	G>A	G	A
NC_000024.9:g.7384185T>G	7384185	7384185	ZK921	T>G	T	G
NC_000024.9:g.7421538G>C	7421538	7421538	ZK922	G>C	G	C
NC_000024.9:g.7422867G>A	7422867	7422867	ZK923	G>A	G	A
NC_000024.9:g.7423393G>A	7423393	7423393	ZK924	G>A	G	A
NC_000024.9:g.7425285A>G	7425285	7425285	ZK925	A>G	A	G
NC_000024.9:g.13592537A>T	13592537	13592537	ZK926	A>T	A	T
NC_000024.9:g.13880811T>C	13880811	13880811	ZK927	T>C	T	C
NC_000024.9:g.13890492C>A	13890492	13890492	ZK928	C>A	C	A
NC_000024.9:g.13899162C>T	13899162	13899162	ZK929	C>T	C	T
NC_000024.9:g.13906388T>C	13906388	13906388	ZK930	T>C	T	C
NC_000024.9:g.13906721T>C	13906721	13906721	ZK931	T>C	T	C
NC_000024.9:g.13911024G>C	13911024	13911024	ZK932	G>C	G	C
NC_000024.9:g.13916673G>A	13916673	13916673	ZK933	G>A	G	A
NC_000024.9:g.13925979G>A	13925979	13925979	ZK934	G>A	G	A
NC_000024.9:g.13930405A>T	13930405	13930405	ZK935	A>T	A	T
NC_000024.9:g.13938301G>A	13938301	13938301	ZK936	G>A	G	A
NC_000024.9:g.13943336C>G	13943336	13943336	ZK937	C>G	C	G
NC_000024.9:g.13951519A>T	13951519	13951519	ZK938	A>T	A	T
NC_000024.9:g.13952526G>A	13952526	13952526	ZK939	G>A	G	A
NC_000024.9:g.13960659G>A	13960659	13960659	ZK940	G>A	G	A
NC_000024.9:g.13960910G>T	13960910	13960910	ZK941	G>T	G	T
NC_000024.9:g.13984391T>C	13984391	13984391	ZK942	T>C	T	C
NC_000024.9:g.13991271A>G	13991271	13991271	ZK943	A>G	A	G
NC_000024.9:g.13993520C>T	13993520	13993520	ZK944	C>T	C	T
NC_000024.9:g.13995608C>G	13995608	13995608	ZK945	C>G	C	G
NC_000024.9:g.14013093G>A	14013093	14013093	ZK946	G>A	G	A
NC_000024.9:g.14050334A>G	14050334	14050334	ZK947	A>G	A	G
NC_000024.9:g.14052765G>A	14052765	14052765	ZK948	G>A	G	A
NC_000024.9:g.14069198G>C	14069198	14069198	ZK949	G>C	G	C
NC_000024.9:g.14106062C>T	14106062	14106062	ZK950	C>T	C	T
NC_000024.9:g.14135205G>A	14135205	14135205	ZK951	G>A	G	A
NC_000024.9:g.14137376G>A	14137376	14137376	ZK952	G>A	G	A
NC_000024.9:g.14140359T>C	14140359	14140359	ZK953	T>C	T	C
NC_000024.9:g.14155852A>T	14155852	14155852	ZK954	A>T	A	T
NC_000024.9:g.14158128T>C	14158128	14158128	ZK955	T>C	T	C
NC_000024.9:g.14173887T>C	14173887	14173887	ZK956	T>C	T	C
NC_000024.9:g.14182981G>C	14182981	14182981	ZK957	G>C	G	C
NC_000024.9:g.14204559A>G	14204559	14204559	ZK958	A>G	A	G
NC_000024.9:g.14214581C>T	14214581	14214581	ZK959	C>T	C	T
NC_000024.9:g.14240542G>T	14240542	14240542	ZK960	G>T	G	T
NC_000024.9:g.14294612C>G	14294612	14294612	ZK961	C>G	C	G
NC_000024.9:g.14298657G>T	14298657	14298657	ZK962	G>T	G	T
NC_000024.9:g.14300951G>A	14300951	14300951	ZK963	G>A	G	A
NC_000024.9:g.14320520A>G	14320520	14320520	ZK964	A>G	A	G
NC_000024.9:g.14335531C>T	14335531	14335531	ZK965	C>T	C	T
NC_000024.9:g.14366620G>A	14366620	14366620	ZK966	G>A	G	A
NC_000024.9:g.14374137C>G	14374137	14374137	ZK967	C>G	C	G
NC_000024.9:g.14388988G>A	14388988	14388988	ZK968	G>A	G	A
NC_000024.9:g.14408172G>A	14408172	14408172	ZK969	G>A	G	A
NC_000024.9:g.14427882A>G	14427882	14427882	ZK970	A>G	A	G
NC_000024.9:g.14516462C>T	14516462	14516462	ZK971	C>T	C	T
NC_000024.9:g.14668242G>C	14668242	14668242	ZK972	G>C	G	C
NC_000024.9:g.14680624C>A	14680624	14680624	ZK973	C>A	C	A
NC_000024.9:g.14707716A>G	14707716	14707716	ZK974	A>G	A	G
NC_000024.9:g.14771566A>C	14771566	14771566	ZK975	A>C	A	C
NC_000024.9:g.14779266T>C	14779266	14779266	ZK976	T>C	T	C
NC_000024.9:g.14797669G>A	14797669	14797669	ZK977	G>A	G	A
NC_000024.9:g.14846275C>T	14846275	14846275	ZK978	C>T	C	T
NC_000024.9:g.14858091T>C	14858091	14858091	ZK979	T>C	T	C
NC_000024.9:g.14898345A>G	14898345	14898345	ZK980	A>G	A	G
NC_000024.9:g.14902484C>T	14902484	14902484	ZK981	C>T	C	T
NC_000024.9:g.14950430C>T	14950430	14950430	ZK982	C>T	C	T
NC_000024.9:g.14975480C>G	14975480	14975480	ZK983	C>G	C	G
NC_000024.9:g.15024567A>G	15024567	15024567	ZK984	A>G	A	G
NC_000024.9:g.15042720T>C	15042720	15042720	ZK985	T>C	T	C
NC_000024.9:g.15055688G>A	15055688	15055688	ZK986	G>A	G	A
NC_000024.9:g.15113816G>C	15113816	15113816	ZK987	G>C	G	C
NC_000024.9:g.15152601C>A	15152601	15152601	ZK988	C>A	C	A
NC_000024.9:g.15155460T>C	15155460	15155460	ZK989	T>C	T	C
NC_000024.9:g.15224406T>C	15224406	15224406	ZK990	T>C	T	C
NC_000024.9:g.15265652G>T	15265652	15265652	ZK991	G>T	G	T
NC_000024.9:g.15300494C>A	15300494	15300494	ZK992	C>A	C	A
NC_000024.9:g.15378454T>A	15378454	15378454	ZK993	T>A	T	A
NC_000024.9:g.15394799G>A	15394799	15394799	ZK994	G>A	G	A
NC_000024.9:g.15397808C>A	15397808	15397808	ZK995	C>A	C	A
NC_000024.9:g.15398726T>G	15398726	15398726	ZK996	T>G	T	G
NC_000024.9:g.15426120C>T	15426120	15426120	ZK997	C>T	C	T
NC_000024.9:g.15478986T>C	15478986	15478986	ZK998	T>C	T	C
NC_000024.9:g.15490850T>C	15490850	15490850	ZK999	T>C	T	C
NC_000024.9:g.15536871G>A	15536871	15536871	ZK1000	G>A	G	A
NC_000024.9:g.15542191T>C	15542191	15542191	ZK1001	T>C	T	C
NC_000024.9:g.15563591C>T	15563591	15563591	ZK1002	C>T	C	T
NC_000024.9:g.15584524T>C	15584524	15584524	ZK1003	T>C	T	C
NC_000024.9:g.15613845A>C	15613845	15613845	ZK1004	A>C	A	C
NC_000024.9:g.15663481G>A	15663481	15663481	ZK1005	G>A	G	A
NC_000024.9:g.15670302A>T	15670302	15670302	ZK1006	A>T	A	T
NC_000024.9:g.15721072T>A	15721072	15721072	ZK1007	T>A	T	A

NC_000024.9:g.15723444C>T	15723444	15723444	ZK1008	C>T	C	T
NC_000024.9:g.15746210T>A	15746210	15746210	ZK1009	T>A	T	A
NC_000024.9:g.15748934G>T	15748934	15748934	ZK1010	G>T	G	T
NC_000024.9:g.15750009C>A	15750009	15750009	ZK1011	C>A	C	A
NC_000024.9:g.15750785A>G	15750785	15750785	ZK1012	A>G	A	G
NC_000024.9:g.15769185C>T	15769185	15769185	ZK1013	C>T	C	T
NC_000024.9:g.15773850T>C	15773850	15773850	ZK1014	T>C	T	C
NC_000024.9:g.15799846G>C	15799846	15799846	ZK1015	G>C	G	C
NC_000024.9:g.15842376A>T	15842376	15842376	ZK1016	A>T	A	T
NC_000024.9:g.15862766G>A	15862766	15862766	ZK1017	G>A	G	A
NC_000024.9:g.15867625T>C	15867625	15867625	ZK1018	T>C	T	C
NC_000024.9:g.15871549A>G	15871549	15871549	ZK1019	A>G	A	G
NC_000024.9:g.15936713T>A	15936713	15936713	ZK1020	T>A	T	A
NC_000024.9:g.15966529C>T	15966529	15966529	ZK1021	C>T	C	T
NC_000024.9:g.16009043G>A	16009043	16009043	ZK1022	G>A	G	A
NC_000024.9:g.16019271G>C	16019271	16019271	ZK1023	G>C	G	C
NC_000024.9:g.16052672A>T	16052672	16052672	ZK1024	A>T	A	T
NC_000024.9:g.16065183G>A	16065183	16065183	ZK1025	G>A	G	A
NC_000024.9:g.16087457G>A	16087457	16087457	ZK1026	G>A	G	A
NC_000024.9:g.16088641A>G	16088641	16088641	ZK1027	A>G	A	G
NC_000024.9:g.16192239A>T	16192239	16192239	ZK1028	A>T	A	T
NC_000024.9:g.16219506T>C	16219506	16219506	ZK1029	T>C	T	C
NC_000024.9:g.16270870G>A	16270870	16270870	ZK1030	G>A	G	A
NC_000024.9:g.16290067G>T	16290067	16290067	ZK1031	G>T	G	T
NC_000024.9:g.16373516A>T	16373516	16373516	ZK1032	A>T	A	T
NC_000024.9:g.16377077T>C	16377077	16377077	ZK1033	T>C	T	C
NC_000024.9:g.16381866C>T	16381866	16381866	ZK1034	C>T	C	T
NC_000024.9:g.16413618T>C	16413618	16413618	ZK1035	T>C	T	C
NC_000024.9:g.16464477G>A	16464477	16464477	ZK1036	G>A	G	A
NC_000024.9:g.16466289A>G	16466289	16466289	ZK1037	A>G	A	G
NC_000024.9:g.16468047T>G	16468047	16468047	ZK1038	T>G	T	G
NC_000024.9:g.16540110C>G	16540110	16540110	ZK1039	C>G	C	G
NC_000024.9:g.16549241G>A	16549241	16549241	ZK1040	G>A	G	A
NC_000024.9:g.16569074G>A	16569074	16569074	ZK1041	G>A	G	A
NC_000024.9:g.16580545G>A	16580545	16580545	ZK1042	G>A	G	A
NC_000024.9:g.16622325A>G	16622325	16622325	ZK1043	A>G	A	G
NC_000024.9:g.16627954T>A	16627954	16627954	ZK1044	T>A	T	A
NC_000024.9:g.16661126T>C	16661126	16661126	ZK1045	T>C	T	C
NC_000024.9:g.16669005C>T	16669005	16669005	ZK1046	C>T	C	T
NC_000024.9:g.16672802G>C	16672802	16672802	ZK1047	G>C	G	C
NC_000024.9:g.16686181A>G	16686181	16686181	ZK1048	A>G	A	G
NC_000024.9:g.16748896A>G	16748896	16748896	ZK1049	A>G	A	G
NC_000024.9:g.16859160G>T	16859160	16859160	ZK1050	G>T	G	T
NC_000024.9:g.16882612G>A	16882612	16882612	ZK1051	G>A	G	A
NC_000024.9:g.16887697G>C	16887697	16887697	ZK1052	G>C	G	C
NC_000024.9:g.16931011G>A	16931011	16931011	ZK1053	G>A	G	A
NC_000024.9:g.16983886C>A	16983886	16983886	ZK1054	C>A	C	A
NC_000024.9:g.16987349T>C	16987349	16987349	ZK1055	T>C	T	C
NC_000024.9:g.17001879C>T	17001879	17001879	ZK1056	C>T	C	T
NC_000024.9:g.17052947A>G	17052947	17052947	ZK1057	A>G	A	G
NC_000024.9:g.17116055A>G	17116055	17116055	ZK1058	A>G	A	G
NC_000024.9:g.17197729G>A	17197729	17197729	ZK1059	G>A	G	A
NC_000024.9:g.17247632A>T	17247632	17247632	ZK1060	A>T	A	T
NC_000024.9:g.17247765A>G	17247765	17247765	ZK1061	A>G	A	G
NC_000024.9:g.17271252A>C	17271252	17271252	ZK1062	A>C	A	C
NC_000024.9:g.17271826C>T	17271826	17271826	ZK1063	C>T	C	T
NC_000024.9:g.17301002T>A	17301002	17301002	ZK1064	T>A	T	A
NC_000024.9:g.17303267G>A	17303267	17303267	ZK1065	G>A	G	A
NC_000024.9:g.17342978C>T	17342978	17342978	ZK1066	C>T	C	T
NC_000024.9:g.17344437C>A	17344437	17344437	ZK1067	C>A	C	A
NC_000024.9:g.17378362T>C	17378362	17378362	ZK1068	T>C	T	C
NC_000024.9:g.17379614G>C	17379614	17379614	ZK1069	G>C	G	C
NC_000024.9:g.17396801C>T	17396801	17396801	ZK1070	C>T	C	T
NC_000024.9:g.17401317G>T	17401317	17401317	ZK1071	G>T	G	T
NC_000024.9:g.17452845C>T	17452845	17452845	ZK1072	C>T	C	T
NC_000024.9:g.17479748G>A	17479748	17479748	ZK1073	G>A	G	A
NC_000024.9:g.17486885T>C	17486885	17486885	ZK1074	T>C	T	C
NC_000024.9:g.17511501C>A	17511501	17511501	ZK1075	C>A	C	A
NC_000024.9:g.17517224G>A	17517224	17517224	ZK1076	G>A	G	A
NC_000024.9:g.17537592C>A	17537592	17537592	ZK1077	C>A	C	A
NC_000024.9:g.17547854A>G	17547854	17547854	ZK1078	A>G	A	G
NC_000024.9:g.17550024G>T	17550024	17550024	ZK1079	G>T	G	T
NC_000024.9:g.17580808G>C	17580808	17580808	ZK1080	G>C	G	C
NC_000024.9:g.17581023T>C	17581023	17581023	ZK1081	T>C	T	C
NC_000024.9:g.17600776A>G	17600776	17600776	ZK1082	A>G	A	G
NC_000024.9:g.17606105T>A	17606105	17606105	ZK1083	T>A	T	A
NC_000024.9:g.17628409G>T	17628409	17628409	ZK1084	G>T	G	T
NC_000024.9:g.17651995T>C	17651995	17651995	ZK1085	T>C	T	C
NC_000024.9:g.17661360G>A	17661360	17661360	ZK1086	G>A	G	A
NC_000024.9:g.17716295T>C	17716295	17716295	ZK1087	T>C	T	C
NC_000024.9:g.17779395G>T	17779395	17779395	ZK1088	G>T	G	T
NC_000024.9:g.17825102C>G	17825102	17825102	ZK1089	C>G	C	G
NC_000024.9:g.17856190T>C	17856190	17856190	ZK1090	T>C	T	C
NC_000024.9:g.17871541G>A	17871541	17871541	ZK1091	G>A	G	A
NC_000024.9:g.17872359G>A	17872359	17872359	ZK1092	G>A	G	A
NC_000024.9:g.17888137G>A	17888137	17888137	ZK1093	G>A	G	A
NC_000024.9:g.17933992G>T	17933992	17933992	ZK1094	G>T	G	T
NC_000024.9:g.17936275T>C	17936275	17936275	ZK1095	T>C	T	C
NC_000024.9:g.17951614G>A	17951614	17951614	ZK1096	G>A	G	A
NC_000024.9:g.17964279C>A	17964279	17964279	ZK1097	C>A	C	A
NC_000024.9:g.17964841A>T	17964841	17964841	ZK1098	A>T	A	T
NC_000024.9:g.17966839A>C	17966839	17966839	ZK1099	A>C	A	C
NC_000024.9:g.17975935T>C	17975935	17975935	ZK1100	T>C	T	C
NC_000024.9:g.17985097T>G	17985097	17985097	ZK1101	T>G	T	G
NC_000024.9:g.18038008T>C	18038008	18038008	ZK1102	T>C	T	C
NC_000024.9:g.18059637C>T	18059637	18059637	ZK1103	C>T	C	T
NC_000024.9:g.18107729C>G	18107729	18107729	ZK1104	C>G	C	G
NC_000024.9:g.18128995T>A	18128995	18128995	ZK1105	T>A	T	A
NC_000024.9:g.18145419C>A	18145419	18145419	ZK1106	C>A	C	A
NC_000024.9:g.18221611G>C	18221611	18221611	ZK1107	G>C	G	C
NC_000024.9:g.18550160T>A	18550160	18550160	ZK1108	T>A	T	A
NC_000024.9:g.18558256A>T	18558256	18558256	ZK1109	A>T	A	T
NC_000024.9:g.18587204G>A	18587204	18587204	ZK1110	G>A	G	A
NC_000024.9:g.18650986T>C	18650986	18650986	ZK1111	T>C	T	C

NC_000024.9:g.18673656A>C	18673656	18673656	ZK1112	A>C	A	C
NC_000024.9:g.18682125A>G	18682125	18682125	ZK1113	A>G	A	G
NC_000024.9:g.18713474T>C	18713474	18713474	ZK1114	T>C	T	C
NC_000024.9:g.18724337G>A	18724337	18724337	ZK1115	G>A	G	A
NC_000024.9:g.18801299A>G	18801299	18801299	ZK1116	A>G	A	G
NC_000024.9:g.18819362A>T	18819362	18819362	ZK1117	A>T	A	T
NC_000024.9:g.18822710T>A	18822710	18822710	ZK1118	T>A	T	A
NC_000024.9:g.18848846C>A	18848846	18848846	ZK1119	C>A	C	A
NC_000024.9:g.18870802A>T	18870802	18870802	ZK1120	A>T	A	T
NC_000024.9:g.18873640T>C	18873640	18873640	ZK1121	T>C	T	C
NC_000024.9:g.18893118C>T	18893118	18893118	ZK1122	C>T	C	T
NC_000024.9:g.18897478A>G	18897478	18897478	ZK1123	A>G	A	G
NC_000024.9:g.18983574T>A	18983574	18983574	ZK1124	T>A	T	A
NC_000024.9:g.19000291T>C	19000291	19000291	ZK1125	T>C	T	C
NC_000024.9:g.19021569T>G	19021569	19021569	ZK1126	T>G	T	G
NC_000024.9:g.19053995T>A	19053995	19053995	ZK1127	T>A	T	A
NC_000024.9:g.19055602C>A	19055602	19055602	ZK1128	C>A	C	A
NC_000024.9:g.19073691G>A	19073691	19073691	ZK1129	G>A	G	A
NC_000024.9:g.19088270G>A	19088270	19088270	ZK1130	G>A	G	A
NC_000024.9:g.19181135T>C	19181135	19181135	ZK1131	T>C	T	C
NC_000024.9:g.19193538C>T	19193538	19193538	ZK1132	C>T	C	T
NC_000024.9:g.19216250C>A	19216250	19216250	ZK1133	C>A	C	A
NC_000024.9:g.19242728G>A	19242728	19242728	ZK1134	G>A	G	A
NC_000024.9:g.19251329G>T	19251329	19251329	ZK1135	G>T	G	T
NC_000024.9:g.19253796G>T	19253796	19253796	ZK1136	G>T	G	T
NC_000024.9:g.19269208G>T	19269208	19269208	ZK1137	G>T	G	T
NC_000024.9:g.19300875A>G	19300875	19300875	ZK1138	A>G	A	G
NC_000024.9:g.19301001G>T	19301001	19301001	ZK1139	G>T	G	T
NC_000024.9:g.19333716G>A	19333716	19333716	ZK1140	G>A	G	A
NC_000024.9:g.19336700A>G	19336700	19336700	ZK1141	A>G	A	G
NC_000024.9:g.19372329T>C	19372329	19372329	ZK1142	T>C	T	C
NC_000024.9:g.19396120A>G	19396120	19396120	ZK1143	A>G	A	G
NC_000024.9:g.19411230T>C	19411230	19411230	ZK1144	T>C	T	C
NC_000024.9:g.19432418T>C	19432418	19432418	ZK1145	T>C	T	C
NC_000024.9:g.19438847T>C	19438847	19438847	ZK1146	T>C	T	C
NC_000024.9:g.19477372G>C	19477372	19477372	ZK1147	G>C	G	C
NC_000024.9:g.19485337G>A	19485337	19485337	ZK1148	G>A	G	A
NC_000024.9:g.19502325G>A	19502325	19502325	ZK1149	G>A	G	A
NC_000024.9:g.19515581A>G	19515581	19515581	ZK1150	A>G	A	G
NC_000024.9:g.19521373G>A	19521373	19521373	ZK1151	G>A	G	A
NC_000024.9:g.19553978T>C	19553978	19553978	ZK1152	T>C	T	C
NC_000024.9:g.21093210C>A	21093210	21093210	ZK1153	C>A	C	A
NC_000024.9:g.21098567G>C	21098567	21098567	ZK1154	G>C	G	C
NC_000024.9:g.21201621G>T	21201621	21201621	ZK1155	G>T	G	T
NC_000024.9:g.21212045A>T	21212045	21212045	ZK1156	A>T	A	T
NC_000024.9:g.21245281A>G	21245281	21245281	ZK1157	A>G	A	G
NC_000024.9:g.21253975A>G	21253975	21253975	ZK1158	A>G	A	G
NC_000024.9:g.21268890T>C	21268890	21268890	ZK1159	T>C	T	C
NC_000024.9:g.21276723C>T	21276723	21276723	ZK1160	C>T	C	T
NC_000024.9:g.21287134G>C	21287134	21287134	ZK1161	G>C	G	C
NC_000024.9:g.21288805T>C	21288805	21288805	ZK1162	T>C	T	C
NC_000024.9:g.21298553T>A	21298553	21298553	ZK1163	T>A	T	A
NC_000024.9:g.21299667G>T	21299667	21299667	ZK1164	G>T	G	T
NC_000024.9:g.21303776T>C	21303776	21303776	ZK1165	T>C	T	C
NC_000024.9:g.21329636C>T	21329636	21329636	ZK1166	C>T	C	T
NC_000024.9:g.21345271A>G	21345271	21345271	ZK1167	A>G	A	G
NC_000024.9:g.21368213T>A	21368213	21368213	ZK1168	T>A	T	A
NC_000024.9:g.21404924T>G	21404924	21404924	ZK1169	T>G	T	G
NC_000024.9:g.21413711A>T	21413711	21413711	ZK1170	A>T	A	T
NC_000024.9:g.21461803G>A	21461803	21461803	ZK1171	G>A	G	A
NC_000024.9:g.21462067G>A	21462067	21462067	ZK1172	G>A	G	A
NC_000024.9:g.21473205G>A	21473205	21473205	ZK1173	G>A	G	A
NC_000024.9:g.21488877C>T	21488877	21488877	ZK1174	C>T	C	T
NC_000024.9:g.21512685C>A	21512685	21512685	ZK1175	C>A	C	A
NC_000024.9:g.21530604T>G	21530604	21530604	ZK1176	T>G	T	G
NC_000024.9:g.21545198G>A	21545198	21545198	ZK1177	G>A	G	A
NC_000024.9:g.21554994G>A	21554994	21554994	ZK1178	G>A	G	A
NC_000024.9:g.21558096T>A	21558096	21558096	ZK1179	T>A	T	A
NC_000024.9:g.21558581C>A	21558581	21558581	ZK1180	C>A	C	A
NC_000024.9:g.21580657G>A	21580657	21580657	ZK1181	G>A	G	A
NC_000024.9:g.21729206T>C	21729206	21729206	ZK1182	T>C	T	C
NC_000024.9:g.21761548T>C	21761548	21761548	ZK1183	T>C	T	C
NC_000024.9:g.21795946T>G	21795946	21795946	ZK1184	T>G	T	G
NC_000024.9:g.21819512C>A	21819512	21819512	ZK1185	C>A	C	A
NC_000024.9:g.21835842T>G	21835842	21835842	ZK1186	T>G	T	G
NC_000024.9:g.21847573T>G	21847573	21847573	ZK1187	T>G	T	G
NC_000024.9:g.21863391A>G	21863391	21863391	ZK1188	A>G	A	G
NC_000024.9:g.21866987A>G	21866987	21866987	ZK1189	A>G	A	G
NC_000024.9:g.21915792C>T	21915792	21915792	ZK1190	C>T	C	T
NC_000024.9:g.21979426A>G	21979426	21979426	ZK1191	A>G	A	G
NC_000024.9:g.21993626G>C	21993626	21993626	ZK1192	G>C	G	C
NC_000024.9:g.22036788G>A	22036788	22036788	ZK1193	G>A	G	A
NC_000024.9:g.22052483G>A	22052483	22052483	ZK1194	G>A	G	A
NC_000024.9:g.22059379A>G	22059379	22059379	ZK1195	A>G	A	G
NC_000024.9:g.22118893C>T	22118893	22118893	ZK1196	C>T	C	T
NC_000024.9:g.22121699G>C	22121699	22121699	ZK1197	G>C	G	C
NC_000024.9:g.22126480C>T	22126480	22126480	ZK1198	C>T	C	T
NC_000024.9:g.22132283G>T	22132283	22132283	ZK1199	G>T	G	T
NC_000024.9:g.22137081T>A	22137081	22137081	ZK1200	T>A	T	A
NC_000024.9:g.22138342T>A	22138342	22138342	ZK1201	T>A	T	A
NC_000024.9:g.22140913G>T	22140913	22140913	ZK1202	G>T	G	T
NC_000024.9:g.22172395G>C	22172395	22172395	ZK1203	G>C	G	C
NC_000024.9:g.22173519T>C	22173519	22173519	ZK1204	T>C	T	C
NC_000024.9:g.22212686G>A	22212686	22212686	ZK1205	G>A	G	A
NC_000024.9:g.22513704C>T	22513704	22513704	ZK1206	C>T	C	T
NC_000024.9:g.22518278A>G	22518278	22518278	ZK1207	A>G	A	G
NC_000024.9:g.22521067G>A	22521067	22521067	ZK1208	G>A	G	A
NC_000024.9:g.22530233G>T	22530233	22530233	ZK1209	G>T	G	T
NC_000024.9:g.22544324G>T	22544324	22544324	ZK1210	G>T	G	T
NC_000024.9:g.22589542A>C	22589542	22589542	ZK1211	A>C	A	C
NC_000024.9:g.22590454T>C	22590454	22590454	ZK1212	T>C	T	C
NC_000024.9:g.22598523G>A	22598523	22598523	ZK1213	G>A	G	A
NC_000024.9:g.22620880C>T	22620880	22620880	ZK1214	C>T	C	T
NC_000024.9:g.22679155G>A	22679155	22679155	ZK1215	G>A	G	A

NC_000024.9:g.22732023G>A	22732023	22732023	ZK1216	G>A	G	A
NC_000024.9:g.22766801G>A	22766801	22766801	ZK1217	G>A	G	A
NC_000024.9:g.22820587G>A	22820587	22820587	ZK1218	G>A	G	A
NC_000024.9:g.22861978G>C	22861978	22861978	ZK1219	G>C	G	C
NC_000024.9:g.22876593T>C	22876593	22876593	ZK1220	T>C	T	C
NC_000024.9:g.22903313C>G	22903313	22903313	ZK1221	C>G	C	G
NC_000024.9:g.22929953G>C	22929953	22929953	ZK1222	G>C	G	C
NC_000024.9:g.22946466G>A	22946466	22946466	ZK1223	G>A	G	A
NC_000024.9:g.22952780G>A	22952780	22952780	ZK1224	G>A	G	A
NC_000024.9:g.22952833G>A	22952833	22952833	ZK1225	G>A	G	A
NC_000024.9:g.22959478T>A	22959478	22959478	ZK1226	T>A	T	A
NC_000024.9:g.22965080G>A	22965080	22965080	ZK1227	G>A	G	A
NC_000024.9:g.22980464C>A	22980464	22980464	ZK1228	C>A	C	A
NC_000024.9:g.23055562G>A	23055562	23055562	ZK1229	G>A	G	A
NC_000024.9:g.23067337T>C	23067337	23067337	ZK1230	T>C	T	C
NC_000024.9:g.23072226C>T	23072226	23072226	ZK1231	C>T	C	T
NC_000024.9:g.23088484G>T	23088484	23088484	ZK1232	G>T	G	T
NC_000024.9:g.23094613C>T	23094613	23094613	ZK1233	C>T	C	T
NC_000024.9:g.23097703C>T	23097703	23097703	ZK1234	C>T	C	T
NC_000024.9:g.23098112G>T	23098112	23098112	ZK1235	G>T	G	T
NC_000024.9:g.23105081C>T	23105081	23105081	ZK1236	C>T	C	T
NC_000024.9:g.23111844G>A	23111844	23111844	ZK1237	G>A	G	A
NC_000024.9:g.23140460A>T	23140460	23140460	ZK1238	A>T	A	T
NC_000024.9:g.23145553T>C	23145553	23145553	ZK1239	T>C	T	C
NC_000024.9:g.23156840G>T	23156840	23156840	ZK1240	G>T	G	T
NC_000024.9:g.23186722C>T	23186722	23186722	ZK1241	C>T	C	T
NC_000024.9:g.23197399A>T	23197399	23197399	ZK1242	A>T	A	T
NC_000024.9:g.23197814G>T	23197814	23197814	ZK1243	G>T	G	T
NC_000024.9:g.23202252C>T	23202252	23202252	ZK1244	C>T	C	T
NC_000024.9:g.23203670T>G	23203670	23203670	ZK1245	T>G	T	G
NC_000024.9:g.23207053G>A	23207053	23207053	ZK1246	G>A	G	A
NC_000024.9:g.23280315C>T	23280315	23280315	ZK1247	C>T	C	T
NC_000024.9:g.23302847T>C	23302847	23302847	ZK1248	T>C	T	C
NC_000024.9:g.23345519A>T	23345519	23345519	ZK1249	A>T	A	T
NC_000024.9:g.23351733A>T	23351733	23351733	ZK1250	A>T	A	T
NC_000024.9:g.23398547G>A	23398547	23398547	ZK1251	G>A	G	A
NC_000024.9:g.23418037G>C	23418037	23418037	ZK1252	G>C	G	C
NC_000024.9:g.23424098G>A	23424098	23424098	ZK1253	G>A	G	A
NC_000024.9:g.23440383G>A	23440383	23440383	ZK1254	G>A	G	A
NC_000024.9:g.23456777C>A	23456777	23456777	ZK1255	C>A	C	A
NC_000024.9:g.23492402A>T	23492402	23492402	ZK1256	A>T	A	T
NC_000024.9:g.23492862T>C	23492862	23492862	ZK1257	T>C	T	C
NC_000024.9:g.26722065C>A	26722065	26722065	ZK1258	C>A	C	A
NC_000024.9:g.28497250A>G	28497250	28497250	ZK1259	A>G	A	G
NC_000024.9:g.28511195C>T	28511195	28511195	ZK1260	C>T	C	T
NC_000024.9:g.28512450T>C	28512450	28512450	ZK1261	T>C	T	C
NC_000024.9:g.28512519T>C	28512519	28512519	ZK1262	T>C	T	C
NC_000024.9:g.28514172T>A	28514172	28514172	ZK1263	T>A	T	A
NC_000024.9:g.28551811C>T	28551811	28551811	ZK1264	C>T	C	T
NC_000024.9:g.28552545A>G	28552545	28552545	ZK1265	A>G	A	G
NC_000024.9:g.28561438T>C	28561438	28561438	ZK1266	T>C	T	C
NC_000024.9:g.28586632G>A	28586632	28586632	ZK1267	G>A	G	A
NC_000024.9:g.28598174T>C	28598174	28598174	ZK1268	T>C	T	C
NC_000024.9:g.28603262A>T	28603262	28603262	ZK1269	A>T	A	T
NC_000024.9:g.28625255C>T	28625255	28625255	ZK1270	C>T	C	T
NC_000024.9:g.28630092C>A	28630092	28630092	ZK1271	C>A	C	A
NC_000024.9:g.28638279A>T	28638279	28638279	ZK1272	A>T	A	T
NC_000024.9:g.28648554T>C	28648554	28648554	ZK1273	T>C	T	C
NC_000024.9:g.28654236G>A	28654236	28654236	ZK1274	G>A	G	A
NC_000024.9:g.28678214C>G	28678214	28678214	ZK1275	C>G	C	G
NC_000024.9:g.28681291C>T	28681291	28681291	ZK1276	C>T	C	T
NC_000024.9:g.28688609C>A	28688609	28688609	ZK1277	C>A	C	A
NC_000024.9:g.28699595C>T	28699595	28699595	ZK1278	C>T	C	T
NC_000024.9:g.28713093A>G	28713093	28713093	ZK1279	A>G	A	G
NC_000024.9:g.28759844A>G	28759844	28759844	ZK1280	A>G	A	G
NC_000024.9:g.28762421T>C	28762421	28762421	ZK1281	T>C	T	C
NC_000024.9:g.28764277C>T	28764277	28764277	ZK1282	C>T	C	T
NC_000024.9:g.28787021T>G	28787021	28787021	ZK1283	T>G	T	G
NC_000024.9:g.28799004G>A	28799004	28799004	ZK1284	G>A	G	A
NC_000024.9:g.28800412A>G	28800412	28800412	ZK1285	A>G	A	G
NC_000024.9:g.28803786A>G	28803786	28803786	ZK1286	A>G	A	G
NC_000024.9:g.28805050C>T	28805050	28805050	ZK1287	C>T	C	T
NC_000024.9:g.28808555A>G	28808555	28808555	ZK1288	A>G	A	G
NC_000024.9:g.28808637A>G	28808637	28808637	ZK1289	A>G	A	G
NC_000024.9:g.28808647G>C	28808647	28808647	ZK1290	G>C	G	C
NC_000024.9:g.28811505A>T	28811505	28811505	ZK1291	A>T	A	T
NC_000024.9:g.19145335T>A	19145335	19145335+ZS5410,J1a2a1a2c2~	ZS5410,J1a2a1a2c2~	T>A	T	A
