# Supplementary material for: Application of Targeted Y-Chromosomal Capture Enrichment to Increase the Resolution of Native American Haplogroup Q
Source: Hum Mutat. 2024 Jul 29;2024:3046495. doi: 10.1155/2024/3046495 (PMC11918922; doi:10.1155/2024/3046495)
Supplement: Supporting Information — Additional supporting information can be found online in the Supporting Information section. Figure S1. Map of the origin of all 277 haplogroup Q samples from Central and South America included in the study. Modern admixed (ModAdmix, black), ancient indigenous (AncNAM, red), and modern indigenous (ModNAM, blue) samples are presented at the place of their origin. The point size is proportional to the number of samples. Square markers indicate 59 samples genotyped in this study; round markers indicate 218 samples from public databases. Figure S2. Linux command-based bioinformatic pipeline for retrieval, preparation, and variant calling of publicly available sequencing data in the CRAM (or BAM) or FASTQ format. Figure S3. (a) Nine unique regions within the Y chromosome [6]; (b) targeted regions in this work using RNA baits. Figure S4. Bioinformatic pipeline for targeted capture data from SureSelect XT HS2 with the commands on the left side and visual representation of the commands' effects of the respective file on the right side. Figure S5. Median and cumulative read depth of the 59 samples within the nine targeted regions (orange bins). Table S1. References of the 277 samples of ModAdmix, AncNAM, or ModNAM origin. Table S2. Nine unique regions within the Y chromosome targeted in this work. Table S3. Region sizes, number of probes, and total probe size of the two designed probe groups. Table S4. Detailed information on the 4128 variants reported and targeted in this study. Table S5. Phylogenetic hierarchy of Y-chromosomal variants from the Native American haplogroup Q linages. [file 3046495.f1.zip › Supplementary_TableS5_Phylogenetic_tree_Q_Revised.pdf]

Supplementary Table 5: Phylogenetic hierarchy of Y-chromosomal variants from the Native American haplogroup Q lineages[A,B,C]

*: only found in ancient samples

Geographic location[a]	ancient + indigenous	modern + indigenous	modern + admixed	Reported samples

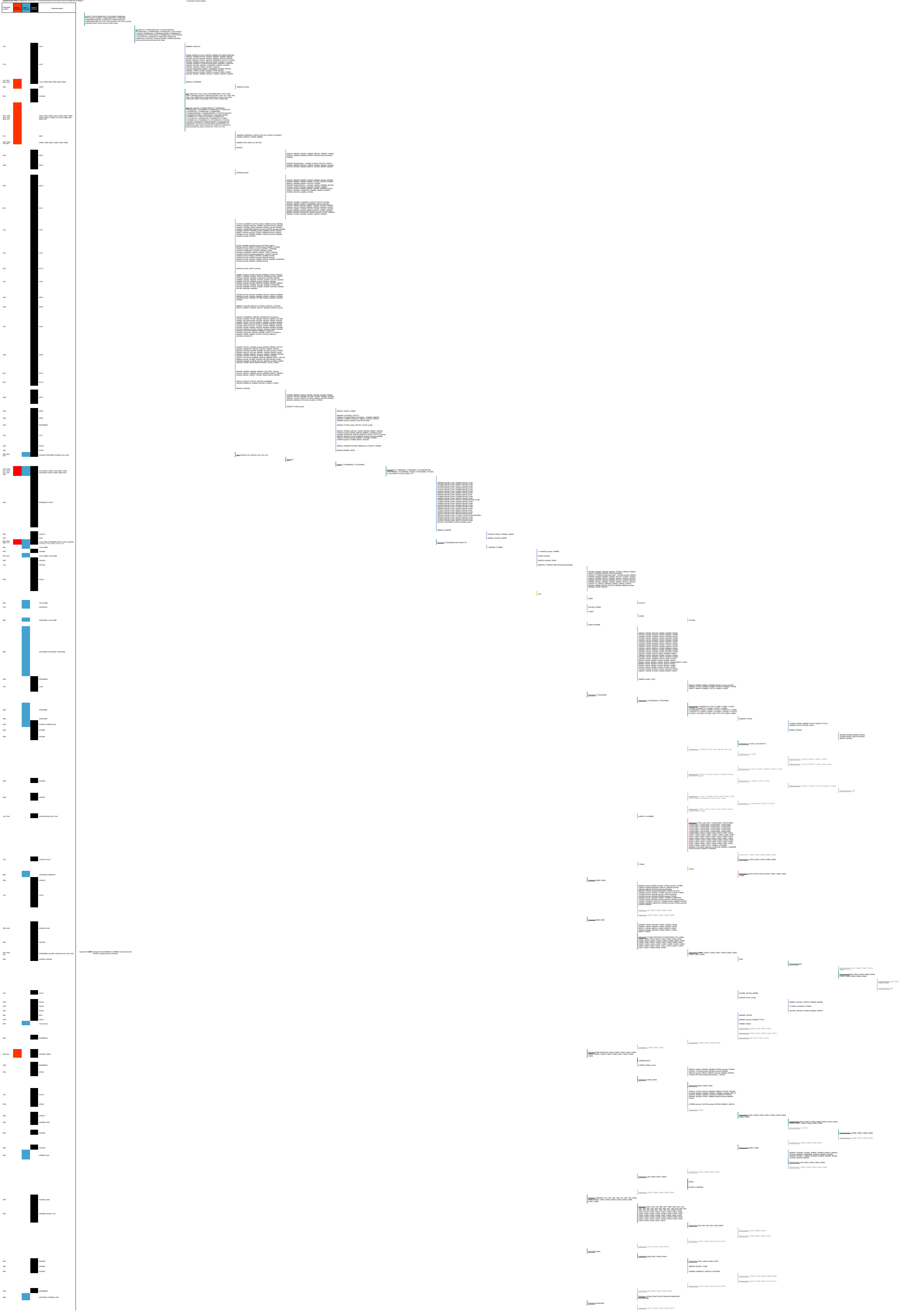

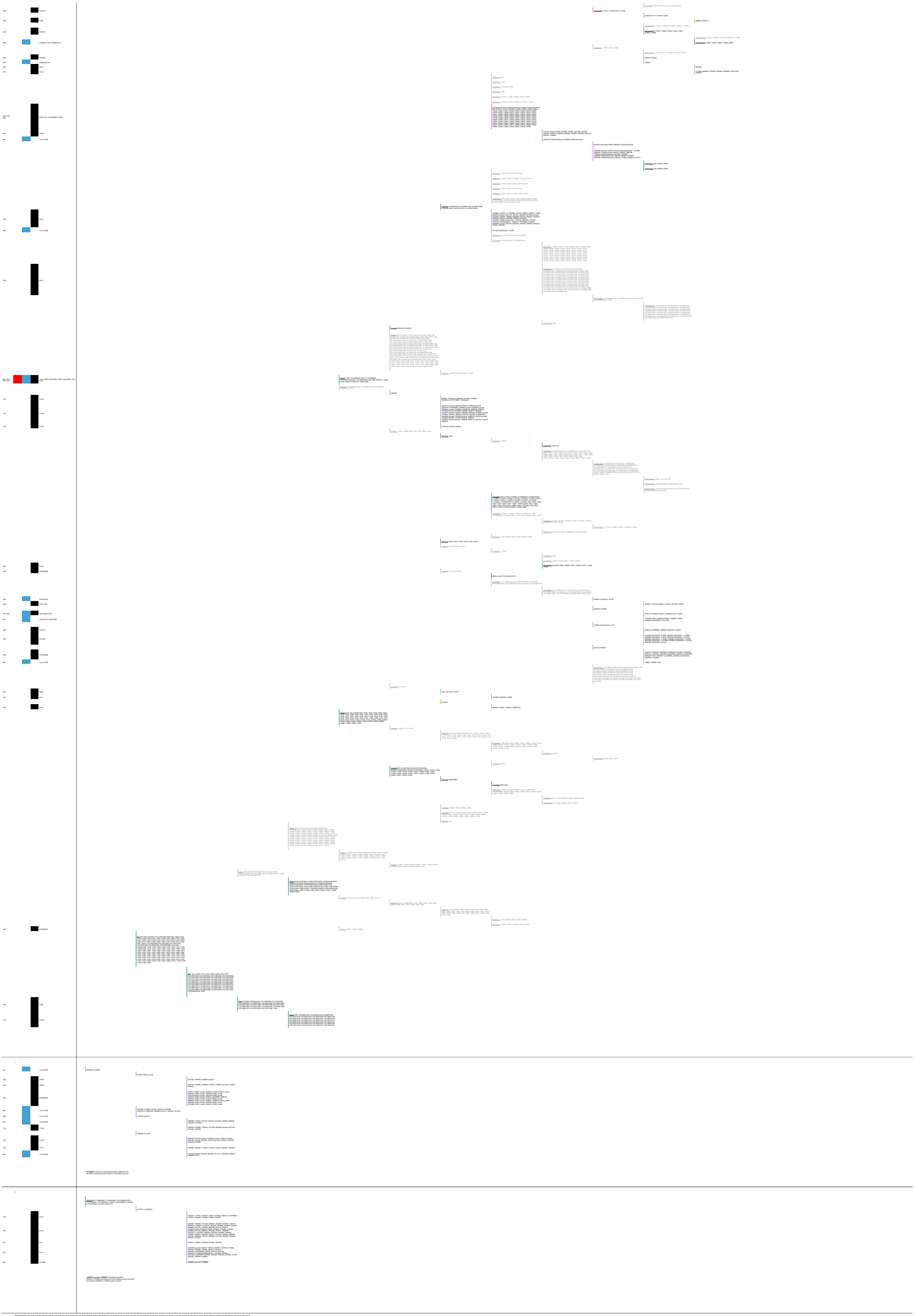

*Reported branches were found in the 281 sequences analyzed in this study and current phylogenetic trees (Y-SNP databases ISOGG Y-DNA Haplogroup Tree 2019-2020 and YFull Ytree v10.07.00). Not distinguishable variants were reported in one branch or tree layer including the SNP name or variant location (GRCh37) combined with information on the source of the variant.

*Each branch is colored as follows: 1) Branches taken from the ISOGG database that remain unchanged are colored green (or grey for the ISOGG branches with approximate locations). The standard haplogroup nomenclature (e.g. Q1, Q1a) is adopted for branches that remain unchanged. 2) The branches inherited from the ISOGG tree marked in pink are further resolved due to the combined information obtained in the tree generation using SNPtotree or/and from the Yfull database. This affects six original branches that are further resolved into 13 branches. 3) The ten branches in orange contain SNPs exclusively found in the Yfull database and neither in ISOGG nor this study. 4) Branches colored in dark purple are resolved due to sequencing data from other studies and the variant names are taken from the respective publications [11], [47]. 5) Branches colored in blue are reported for the first time in this study and are resolved based on the sequencing data from our study and other sources (see individual variants' names). These include 112 entirely newly resolved branches, that also include previously reported SNPs that were not inserted into a phylogenetic tree with this accuracy.

[a] The Variant labels consist of the variant position (relative to NC_000024.9) and (if available) a variant annotation.

[b] Geographic location information follows three letter codes for: ARG (Argentina), BHS (Bahamas), BOL (Bolivia), BRA (Brazil), CHI (Chile), COL (Colombia), DOM (Dominican Republic), ECU (Ecuador), MEX (Mexico), PAN (Panama), PER (Peru), PRI (Puerto Rico), VEN (Venezuela)
