# Supplementary material for: Application of Targeted Y-Chromosomal Capture Enrichment to Increase the Resolution of Native American Haplogroup Q
Source: Hum Mutat. 2024 Jul 29;2024:3046495. doi: 10.1155/2024/3046495 (PMC11918922; doi:10.1155/2024/3046495)
Supplement: Supporting Information — Additional supporting information can be found online in the Supporting Information section. Figure S1. Map of the origin of all 277 haplogroup Q samples from Central and South America included in the study. Modern admixed (ModAdmix, black), ancient indigenous (AncNAM, red), and modern indigenous (ModNAM, blue) samples are presented at the place of their origin. The point size is proportional to the number of samples. Square markers indicate 59 samples genotyped in this study; round markers indicate 218 samples from public databases. Figure S2. Linux command-based bioinformatic pipeline for retrieval, preparation, and variant calling of publicly available sequencing data in the CRAM (or BAM) or FASTQ format. Figure S3. (a) Nine unique regions within the Y chromosome [6]; (b) targeted regions in this work using RNA baits. Figure S4. Bioinformatic pipeline for targeted capture data from SureSelect XT HS2 with the commands on the left side and visual representation of the commands' effects of the respective file on the right side. Figure S5. Median and cumulative read depth of the 59 samples within the nine targeted regions (orange bins). Table S1. References of the 277 samples of ModAdmix, AncNAM, or ModNAM origin. Table S2. Nine unique regions within the Y chromosome targeted in this work. Table S3. Region sizes, number of probes, and total probe size of the two designed probe groups. Table S4. Detailed information on the 4128 variants reported and targeted in this study. Table S5. Phylogenetic hierarchy of Y-chromosomal variants from the Native American haplogroup Q linages. [file 3046495.f1.zip › Supporting_information_Revised2.pdf]

Supporting information

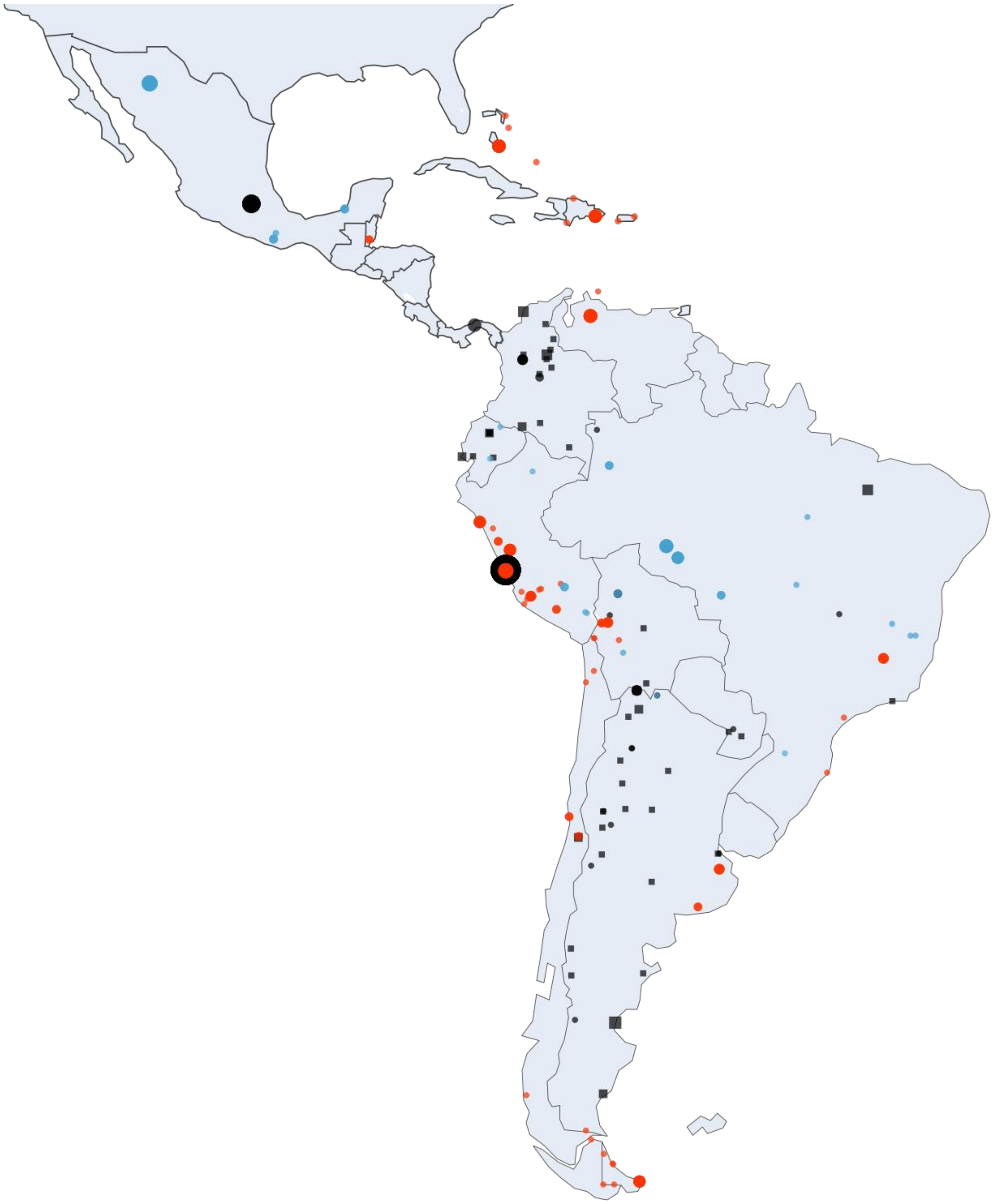

Supplementary Figure S1: Map of the origin of all 277 haplogroup Q samples from Central and South America included in the study. Modern admixed (ModAdmix, black), ancient indigenous (AncNAM, red) and modern indigenous (ModNAM, blue) samples are presented at the place of their origin. The point size is proportional to the number of samples. Square markers indicate 59 samples genotyped in this study, round markers indicate 218 samples from public databases.

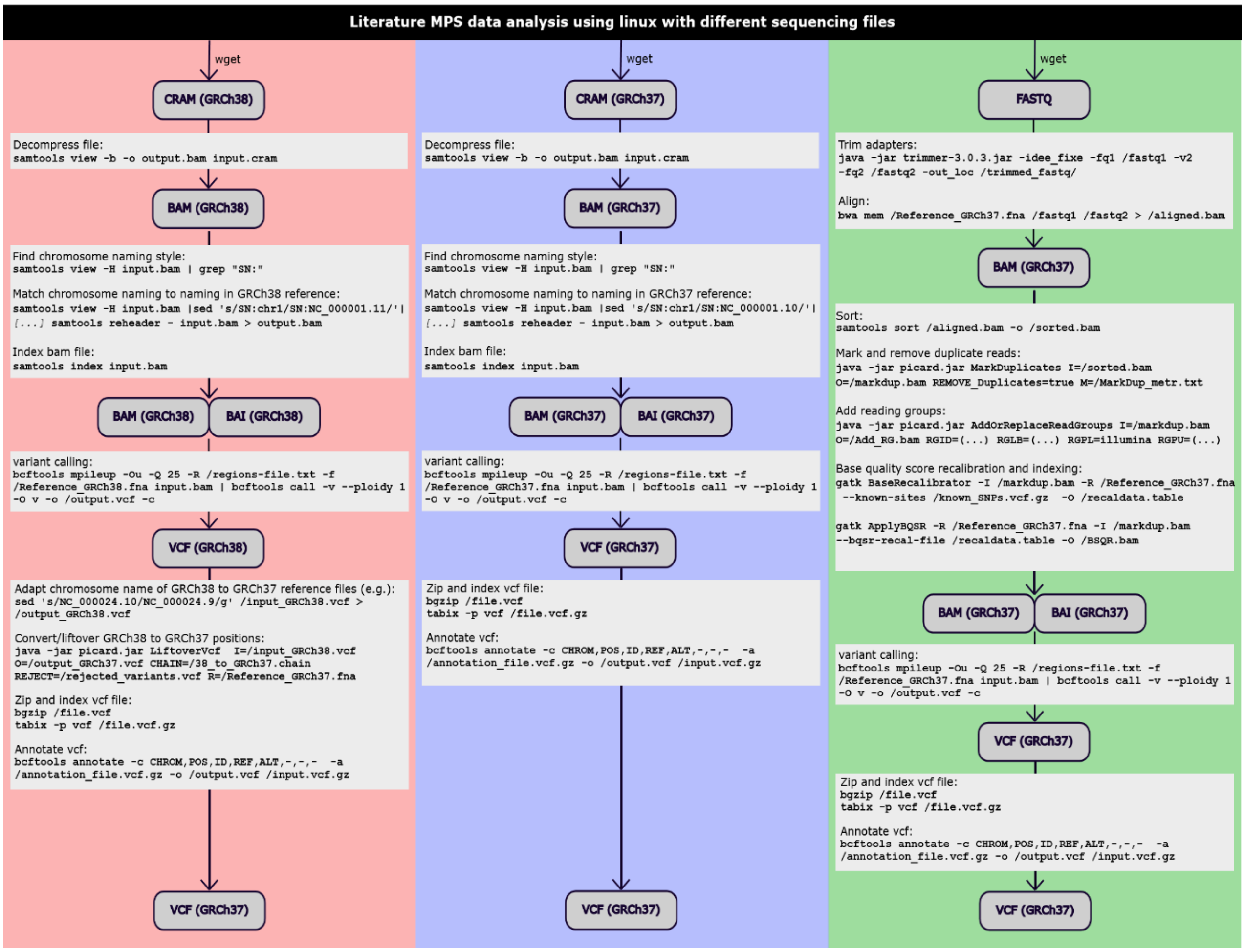

Supplementary Figure S2: Linux command based bioinformatic pipeline for retrieval, preparation and variant calling of publicly available sequencing data in the CRAM (or BAM) or FASTQ format.

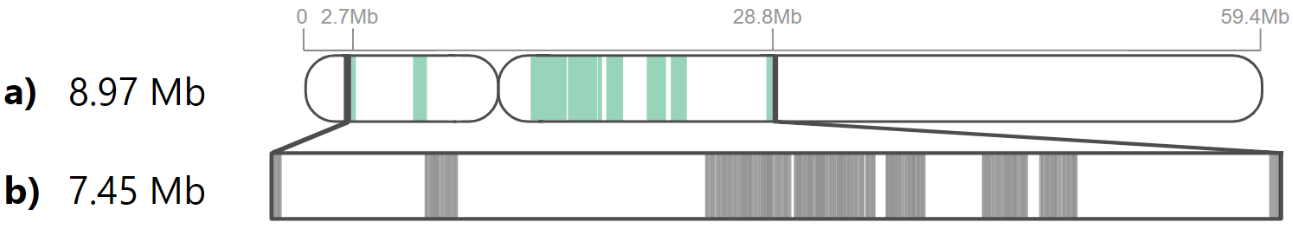

Supplementary Figure S3: (a) Nine unique regions within the Y chromosome [6], (b) targeted regions in this work using RNA baits.

Supplementary Figure S4: Bioinformatic pipeline for targeted capture data from SureSelect XT HS2 with the commands on the left side and visual representation of the commands' effects of the respective file on the right side.

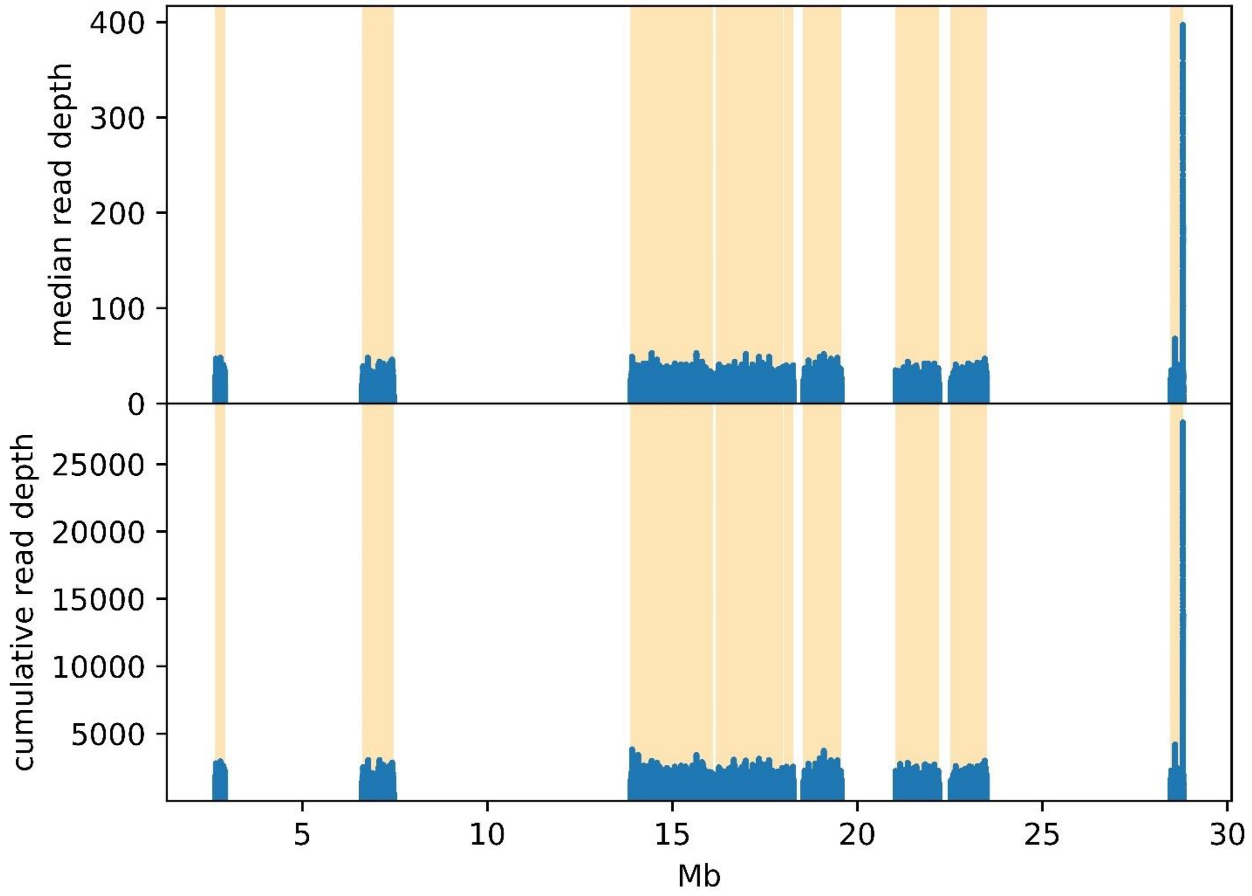

Supplementary Figure S5: Median and cumulative read depth of the 59 samples within the nine targeted regions (orange bins).

Supplementary Table S1: References of the 277 samples of ModAdmix, AncNAM or ModNAM origin.

Reference	File format	Number of samples	Modern/Ancient	Indigenous/Admixed
This study	BCL	59	Modern	Admixed
The 1000 Genomes Project Consortium, 2015 [58]	CRAM	37	Modern	Admixed
Mallick et al., 2016 [59]	BAM	8	Modern	Indigenous
Popović et al., 2021 [48]	BAM	1	Ancient	Indigenous
Pinotti et al., 2019 [28]	CRAM	19	Modern	Indigenous
Bergström et al., 2020 [49]	CRAM	16	Modern	Indigenous
Nakatsuka et al., 2020a,b [50,51]	BAM	40	Ancient	Indigenous
Moreno-Mayar et al., 2018 [52]	BAM	2	Ancient	Indigenous
Paz Sepúlveda et al., 2022 [47]	FASTQ	9	Modern	Admixed
Fernandes et al., 2020 [53]	BAM	33	Ancient	Indigenous
Posth et al., 2018 [54]	BAM	28	Ancient	Indigenous
Grugni et al., 2019 [27]	BAM	25	Modern	Admixed

Supplementary Table S2: Nine unique regions within the Y chromosome targeted in this work.

Region number	Y chr location	Start Position (GRCh37.p13/hg19)	End Position (GRCh37.p13/hg19)	Size (bps)
1	Yp11.2	2,649,807	2,917,723	267,916
2	Yp11.2	6,616,752	7,472,224	855,472
3	Yq11.221	13,870,438	16,095,786	2,225,348
4	Yq11.221, Yq11.222	16,170,614	17,986,473	1,815,859
5	Yq11.222	18,017,095	18,271,273	254,178
6	Yq11.222	18,537,846	19,567,356	1,029,510
7	Yq11.223	21,032,221	22,216,158	1,183,937
8	Yq11.223	22,513,120	23,497,661	984,541
9	Yq12	28,457,993	28,806,758	348,765

Supplementary Table S3: Region sizes, number of probes and total probe size of the two designed probe groups.

	probe group 1	probe group 2	total
**Region Size**	8.965 Mbp	1.507 Mbp	10.472 Mbp
**Total Probes**	315,186	44,768	359,954
**Total Probes Size**	6.999 Mbp	845,674	7.844 Mbp

Supplementary Table S4: Detailed information on the 4,128 variants reported and targeted in this study.

Supplementary Table S5: Phylogenetic hierarchy of Y-chromosomal variants from the Native American haplogroup Q lineages.
