# Supplementary material for: Application of Targeted Y-Chromosomal Capture Enrichment to Increase the Resolution of Native American Haplogroup Q
Source: Hum Mutat. 2024 Jul 29;2024:3046495. doi: 10.1155/2024/3046495 (PMC11918922; doi:10.1155/2024/3046495)

SureSelect XT HS2 MPS pipeline for data analysis using linux

The effects of each command on the corresponding file

toolkit	source code & user guides	command	Troubleshooting & FAQs	comments

red: *add your individual path to a specific file (e.g. input file). Output file is usually the input file for the following step.*
purple: *add your path to this publically available data that was adjusted for the purpose of the analysis as described below in the purple boxes.*
blue: *add adjustable parameter (e.g. number of used threads)*

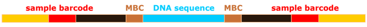

Convert bcl output file from Sequencer to FASTQ format

bcl2fastq2

https://support.illumina.com/sequencing/sequencing_software/bcl2fastq-conversion-software.html

```
nice bcl2fastq2-2.20 -R <input\> -o <output\> -l WARNING -p 48
```

FASTQ:

@A01019:30:HVK5WDRXY:1:2101:7419:1000 1:N:0:**NTACAATG+NTCAAGTC**
NCTGTCCTCACTTCCCAGTAGGGGCGGCCGGGCAGAGGCGCCCCTCACCTCCCAGACGGGAGGCCGTTA*[...]*
+
#FFFFFFFFFFFFFFFFFFFFFFFFFFFFFFFFFFFFFFFFFFFFFFFFFFFFFFFFFFFFFFFFFFFFFFFFFFFFFFF*[...]*

Molecular barcode (MBC) removal, annotation of MBC in FASTQ headers and adapter removal

AGeNT

https://explore.agilent.com/AGeNT-Software-Download-Form-TY ;
https://www.agilent.com/en/product/next-generation-sequencing/hybridization-based-next-generation-sequencing-ngs/ngs-software/agent-232879

```
java -jar /trimmer-3.0.3.jar -fq1 <fastq1_per_sample_per_lane.fastq.gz> -v2 -fq2
<fastq2_per_sample_per_lane.fastq.gz> -out_loc <output>
```

https://support.illumina.com/sequencing/sequencing_software/bcl2fastq-conversion-software.html

demands larger memory space: alternatively you can sub-divide your fastq file into smaller ones

FASTQ:

@A01019:30:HVK5WDRXY:1:2101:7419:1000 BC:Z:**NTACAATG+NTCAAGTC** *[...]* RX:Z:**NCT-NAA** *[...]*
CCTCACTTCCCAGTAGGGGCGGCCGGGCAGAGGCGCCCCTCACCTCCCAGACGGGGCGGCTGGCCGG*[...]*
+
FFFFFFFFFFFFFFFFFFFFFFFFFFFFFFFFFFFFFFFFFFFFFFFFFFFFFFFFFFFFFFFFFFFFFFFFFFFFFF

Alignment to reference genome

BWA

http://bio-bwa.sourceforge.net/

```
bwa mem -C -t 48 <reference.fna> <trimmed_read1_per_sample_per_lane.fastq.gz>
<trimmed_read1_per_sample_per_lane.fastq.gz> > <output.bam>
```

-C: necessary to transfer information (MBC) in the header of the FASTQ to the SAM/BAM file

BAM:

@SQ SN:NC_000001.10 LN:249250621
[...]
A01019:30:HVK5WDRXY:1:2101:7419:1000 83 **NC_000003.11** 178838415 076M = 178838415 -76
GCCCTCCGTCCGGCCAGCCGCCCCGTCTGGGAGGTGAGGGGCGCCTCTGCCCGGCCGCCCCTACTGGGAAGTGAGG
FFFFFFFFFFFFFFFFFFFFFFFFFFFFFFF*[...]* BC:Z:**NTACAATG+NTCAAGTC** *[...]* RX:Z:**NCT-NAA** QX:Z:#FF #:F

**reverse complemented sequence*

<reference.fna>

1. Download fasta file of human reference genome GRCh37.p13: https://www.ncbi.nlm.nih.gov/assembly/GCF_000001405.25/
2. Unpack downloaded .tar file:

```
tar -xvf <GRCh37.p13_genome_assemblies_genome_fasta.tar>
```

3. Unzip zipped file:

```
gunzip <GCF_000001405.25_GRCh37.p13_genomic.fna.gz>
```

4. Index fasta file to get fna.fai file using both samtools and bwa commands:

```
samtools faidx <GCF_000001405.25_GRCh37.p13_genomic.fna>
bwa index <GCF_000001405.25_GRCh37.p13_genomic.fna>
```

5. Generate dictionary file for fasta file:

```
java -jar <picard.jar> CreateSequenceDictionary R=<GCF_000001405.25_GRCh37.p13_genomic.fna> O=<GCF_000001405.25_GRCh37.p13_genomic.dict>
```

Sort reads in bam file by coordinate

samtools

http://www.htslib.org/ ; http://www.htslib.org/doc/samtools-sort.html

```
samtools sort <input.bam> -o <output.bam>
```

BAM:

[...] A01019:30:HVK5WDRXY:1:2230:27046:23375 99 **NC_000001.10** 10003 0 108M38S = 10003 68
ACCCTAACCCTAACCCTAACCCTAACCCTAACCCTAACCCTAACCCTAACCCTAACCCTAACCCTAACCCTAA*[...]*
FFFFFFFFFFFFFFFFFFFFFF NM:i:0 MD:Z:108 MC:Z:77S57M2I11M AS:i:108 XS:i:106 BC:Z:**CTACAATG+GTCAAGTC**
ZA:Z:GGTGT ZB:Z:ATGT RX:Z:**GGT-ATG** QX:Z:FFF F:F

first entry is on chromosome 1 (NC_000001.10) instead of 3 (NC_000003.11)

Add Read group

picard

https://broadinstitute.github.io/picard/index.html ;
https://broadinstitute.github.io/picard/command-line-overview.html#AddOrReplaceReadGroups

```
java -jar picard.jar AddOrReplaceReadGroups I=<input.bam> O=<output.bam>
RGID=Read_group_ID:1 RGLB=sample_name RGPL=illumina
RGPU=Read_group_ID:1:Read_Group_platform_unit(run_barcode) RGSM=sample_name
```

https://gatk.broadinstitute.org/hc/en-us/articles/360035890671?id=6472

Read group ID and platform unit are for characterisation purposes

BAM:

A01019:30:HVK5WDRXY:1:2230:27046:23375 99 NC_000001.10 10003 0 108M38S = 10003 68
ACCCTAACCCTAACCCTAACCCTAACCCTAACCCTAACCCTAACCCTAACCCTAACCCTAACCCTAACCCTAA*[...]*
F:F:FF,FF,:FFFF,:F:FFF,F,:F,FF,,,F,F,,,,,,F,,F,,:FF,,:,:,,,,FF,,,:F,F ZA:Z:GGTGT ZB:Z:ATGT BC:Z:**CTACAATG+GTCAAGTC**
MC:Z:77S57M2I11M MD:Z:108 **RG:Z:HVK5WDRXY:1** NM:i:0 AS:i:108 XS:i:106 QX:Z:FFF F:F RX:Z:**GGT-ATG**

Merge bam files of lane 1 and 2 per sample

samtools

http://www.htslib.org/ ; http://www.htslib.org/doc/samtools-merge.html

```
samtools merge -@48 -r <output.bam> <input_lane1.bam> <input_lane2.bam>
```

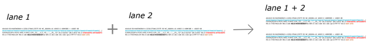

Deduplication of PCR duplicates

AGeNT

https://explore.agilent.com/AGeNT-Software-Download-Form-TY ;
https://www.agilent.com/en/product/next-generation-sequencing/hybridization-based-next-generation-sequencing-ngs/ngs-software/agent-232879

```
java -Xmx48G -jar locatit-2.0.5.jar -v2Duplex -i -S -C -IB -OB -R -m 2 -Q 25 -l
<Covered_regions.bed> -X <Temporary_folder> -o <output.bam> <input.bam>
```

https://support.illumina.com/sequencing/sequencing_software/bcl2fastq-conversion-software.html

-v2Duplex: Duplex consensus mode option
-m 2 : At least 2 read pairs need to be associated with a barcode
-Q 25 : Reads with a base quality score below 25 are filtered out

BAM:

A01019:30:HVK5WDRXY:1:2121:2031:31908 99 NC_000001.10 377263 0 147M = 377392 275
AGGCTGGAGAACAGCAAAGATTGCTGCCTGTTTCTTCCTCTGGAAGTTTTGTCCCAGAGGGGCACCCACCAGATGCCAG*[...]*
FFFFFFFFFFFFFFFFFFFFFFFFFFFFFFFFFFFFFFFFFFFFFFFFFFFF ZA:Z:ATGT ZB:Z:GATCT BC:Z:**CTACAATG+GTCAAGTC**
MC:Z:146M MD:Z:54A92 XF:Z:NC_000001.10:377262:P:=:377537:P RG:Z:S001_cut.sorted.RG XI:i:2 XJ:i:1 NM:i:1
XM:Z:ATG-GAT XQ:Z:FFF FFF AS:i:142 XS:i:147 QX:Z:FFF FFF RX:Z:**ATG-GAT**
rd:Z:[A01019:30:HVK5WDRXY:1:2121:2031:31908,A01019:30:HVK5WDRXY:2:2242:32579:31548]
mg:Z:A01019:30:HVK5WDRXY:1:2252:9588:28432 bq:Z:FFF FFF rq:Z:FFFFFFFFFFFFFFFFFFFFFFFFFFFFFFFFFFFFFFFFF*[...]*

<Covered_regions.bed>

tab-separated table from Agilent SureDesign's "Covered regions" bed file.

Fix Information of the mate read

picard

https://broadinstitute.github.io/picard/index.html ;
https://broadinstitute.github.io/picard/command-line-overview.html#FixMateInformation

```
java -jar picard.jar FixMateInformation I=<input.bam> O=<output.bam> ADD_MATE_CIGAR=true
```

locatit introduces an "CIGAR and query sequence length differ" in some reads. This can be observed using ValidateSamFile on bam files before and after locatit. This error is fixed in the next step.

Index genome sorted BAM file

samtools

http://www.htslib.org/ ; http://www.htslib.org/doc/samtools-index.html

```
samtools index <input.bam>
```

Indexing allows the quick extraction of specific genomic regions from an alignment. It is also a requirement for genome viewers like IGV.

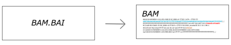

Base quality score calculation Apply machine learning to model systematic base quality errors and re-calculate the quality scores accordingly and output these in a recalibration table

gatk

https://gatk.broadinstitute.org/hc/en-us/articles/360036898312-BaseRecalibrator ;
https://github.com/broadinstitute/gatk-docs ;
https://gatk.broadinstitute.org/hc/en-us/articles/360035890531-Base-Quality-Score-Recalibration-BQSR-

```
gatk BaseRecalibrator -I <input.bam> -R <reference.fna> --known-sites <dbSNP_YSNPs.gz>
-O <BR_output.table>
```

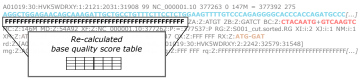

<dbSNP_YSNPs.gz>

1. Download vcf file **00-All.vcf.gz** on https://ftp.ncbi.nlm.nih.gov/snp/organisms/human_9606_b151_GRCh37p13/VCF/
2. filter Y-chromomal SNPs:

```
vcftools --gzvcf <00-All.vcf.gz> --chr Y --recode --out <00-All-SNPs_Yfiltered.vcf>
```

3. zip the filtered vcf file:

```
bgzip <00-All-SNPs_Yfiltered.recode.vcf>
```

4. index vcf file:

```
tabix -p vcf <00-All-SNPs_Yfiltered.recode.vcf.gz>
```

5. Change chromosome names in vcf file, because chromosome names in vcf file need to match chromosome names from the reference sequence used (for Reference sequence GRCh37.p13, the annotated chromosome names are given in the "Assembly unit" table in column "RefSeq sequence" on https://www.ncbi.nlm.nih.gov/assembly/GCF_000001405.25/):
5.1 Generate tab-separated file "Chr_name_translation.txt" with column 1 (=chromosome accession you have in your vcf data) and column 2 (=target chromosome name for that accession):

1	*NC_000001.10*
	...
Y	*NC_000024.9*

```
bcftools annotate --rename-chrs <Chr_name_translation.txt> <00-All-SNPs_Yfiltered.recode.vcf.gz> --threads 48 bgzip -o <00-All-SNPs_Yfiltered.recode.chrnames.vcf>
```

6. zip the resulting vcf file:

```
bgzip <00-All-SNPs_Yfiltered.recode.chrnames.vcf>
```

7. header of the zipped vcf file can be viewed:

```
zcat <00-All-SNPs_Yfiltered.recode.chrnames.vcf.gz> |head -n 100
```

8. index final vcf file:

```
tabix -p vcf <00-All-SNPs_Yfiltered.recode.chrnames.vcf.gz>
```

Create re-calibrated BAM file Correct the base quality values based on the recalibration table

gatk

https://gatk.broadinstitute.org/hc/en-us/articles/360037055712-ApplyBQSR ;
https://github.com/broadinstitute/gatk-docs

```
gatk ApplyBQSR -R <reference.fna>
-I <input.bam> --bqsr-recal-file <BR_output.table> -O <output.BSQR.bam>
```

BAM:

A01019:30:HVK5WDRXY:1:2121:2031:31908 99 NC_000001.10 377263 0 147M = 377392 275
AGGCTGGAGAACAGCAAAGATTGCTGCCTGTTTCTTCCTCTGGAAGTTTTGTCCCAGAGGGGCACCCACCAGATG*[...]*
BECBDECEEEFBBEBBFFEEDGFBDEBBDEDGFDDGD*[...]* ZA:Z:ATGT ZB:Z:GATCT BC:Z:**CTACAATG+GTCAAGTC**
MC:Z:146M MD:Z:54A92 XF:Z:NC_000001.10:377262:P:=:377537:P RG:Z:S001_cut.sorted.RG XI:i:2 XJ:i:1 NM:i:1
XM:Z:ATG-GATMQ:i:0 XQ:Z:FFF FFF AS:i:142 XS:i:147 QX:Z:FFF FFF RX:Z:**ATG-GAT**
rd:Z:[A01019:30:HVK5WDRXY:1:2121:2031:31908, A01019:30:HVK5WDRXY:2:2242:32579:31548]
mg:Z:A01019:30:HVK5WDRXY:1:2252:9588:28432 bq:Z:FFF FFF rq:Z:FFFFFFFFFFFFFFFFFFFFFFFFFFFFFFFFF*[...]*

Index genome sorted BAM file

samtools

http://www.htslib.org/ ; http://www.htslib.org/doc/samtools-index.html

```
samtools index <input.bam>
```

software tools that present parameters of the bam files require indexed bam.bai files.

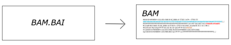

Variant calling mpileup generates genotype likelihoods at each genomic position with coverage, these are then called

bcftools

http://www.htslib.org/doc/bcftools.html#mpileup ; https://samtools.github.io/bcftools/howtos/variant-calling.html

```
bcftools mpileup -Ou -Q 25 -R <regions_of_interest.txt> -f <reference.fna>
<input.bam> | bcftools call -v --ploidy 1 -O v -o <output.vcf> -c
```

mpileup:
-Q: Minimum base quality for a base accepted
-R: Regions file to be considered

call:
-v: output variant sites only
-O v: uncompressed vcf file to be output
-c: consensus caller, since we expect only one variant for haploid data

VCF:

#CHROM	POS	ID	REF	ALT	QUAL	FILTER	INFO	FORMAT	S001
NC_000024.9	2668456	.	T	C	225.007	.	DP=40;*[...]*	GT:PL	1:255,0
NC_000024.9	2679415	.	GC	GCC *[...]*	74.4669	.	INDEL;*[...]*	GT:PL	1:112,5,0

<regions_of_interest.txt>

use whole Y chromosome (like in this study) or targeted regions only by creating tab-separated file taken from SureDesign's "Covered regions" file with 3 columns: **1)** chromosome name (NC_000024.9), **2)** start and **3)** stop position of covered region

Zip and index vcf files this is needed for vcf file annotation

samtools

http://www.htslib.org/ ; http://www.htslib.org/doc/bgzip.html; http://www.htslib.org/doc/tabix.html

```
bgzip <input.vcf>
tabix -p vcf <input.vcf.gz>
```

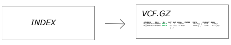

Annotation of vcf file: dbSNP, gnomAD, ISOGG databases detected variants that are already known SNPs can be annotated

bcftools

https://samtools.github.io/bcftools/bcftools.html#annotate

```
bcftools annotate -c CHROM,POS,ID,REF,ALT,-,-,-  -a <dbSNP_YSNPs.gz> -o <output.vcf>
<sample_variants.vcf.gz>
bcftools annotate -c CHROM,POS,ID,REF,ALT,-,-,-  -a <gnomAD3.1_YSNPs.gz> -o <output.vcf>
<sample_variants.vcf.gz>
bcftools annotate -c CHROM,POS,ID,REF,ALT,-,-,-  -a <ISOGG_YSNPs.gz> -o <output.vcf>
<sample_variants.vcf.gz>
```

VCF:

#CHROM	POS	ID	REF	ALT	QUAL	FILTER	INFO	FORMAT	S001
NC_000024.9	2668456	**rs123**	T	C	225.007	.	DP=40;*[...]*	GT:PL	1:255,0
NC_000024.9	2679415	.	GC *[...]*	GCC	74.4669	.	INDEL;*[...]*	GT:PL	1:112,5,0

<gnomAD3.1_YSNPs.gz>

1. Download gnomad variants: https://gnomad.broadinstitute.org/downloads (chrY, mapped to GRCh37)
2. Adapt chromosome naming ("chrY") to naming used here (NC_000024.9) by using a dictionary <Chr_name_translation.txt> (*see for comparison 5.1 in purple box <dbSNP_YSNPs.gz>*)

```
bcftools annotate --rename-chrs <Chr_name_translation.txt> <gnomad_variants.chrY.vcf.bgz> --threads 48 bgzip -o <gnomad_variants.NC_000024.9.vcf.bgz>
```

3. turn bgz into gz:

```
gzip <gnomad_variants.NC_000024.9.vcf.bgz> -c > <gnomad_variants.NC_000024.9.vcf.gz>
```

4. Index zipped vcf file:

```
tabix -p vcf <gnomad_variants.NC_000024.9.vcf.gz>
```

<ISOGG_YSNPs.gz>

compiled file of YSNPs of all haplogroups from ISOGG: https://isogg.org/tree/

Zip and index vcf files this is needed for vcf file annotation

samtools

http://www.htslib.org/ ; http://www.htslib.org/doc/bgzip.html; http://www.htslib.org/doc/tabix.html

```
bgzip <input.vcf>
tabix -p vcf <input.vcf.gz>
```

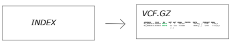

Supplement: Supporting Information — Additional supporting information can be found online in the Supporting Information section. Figure S1. Map of the origin of all 277 haplogroup Q samples from Central and South America included in the study. Modern admixed (ModAdmix, black), ancient indigenous (AncNAM, red), and modern indigenous (ModNAM, blue) samples are presented at the place of their origin. The point size is proportional to the number of samples. Square markers indicate 59 samples genotyped in this study; round markers indicate 218 samples from public databases. Figure S2. Linux command-based bioinformatic pipeline for retrieval, preparation, and variant calling of publicly available sequencing data in the CRAM (or BAM) or FASTQ format. Figure S3. (a) Nine unique regions within the Y chromosome [6]; (b) targeted regions in this work using RNA baits. Figure S4. Bioinformatic pipeline for targeted capture data from SureSelect XT HS2 with the commands on the left side and visual representation of the commands' effects of the respective file on the right side. Figure S5. Median and cumulative read depth of the 59 samples within the nine targeted regions (orange bins). Table S1. References of the 277 samples of ModAdmix, AncNAM, or ModNAM origin. Table S2. Nine unique regions within the Y chromosome targeted in this work. Table S3. Region sizes, number of probes, and total probe size of the two designed probe groups. Table S4. Detailed information on the 4128 variants reported and targeted in this study. Table S5. Phylogenetic hierarchy of Y-chromosomal variants from the Native American haplogroup Q linages. [file Supplementary_FigureS4_Computational_processing.pdf › Supplementary_FigureS4_Computational_processing.pdf]
